# Predicted effect of regionalised delivery care on neonatal mortality, utilisation, financial risk, and patient utility in Malawi: an agent-based modelling analysis

**DOI:** 10.1016/S2214-109X(19)30170-6

**Published:** 2019-06-11

**Authors:** Mark G Shrime, Katherine R Iverson, Rachel Yorlets, Sanam Roder-DeWan, Anna D Gage, Hannah Leslie, Address Malata

**Affiliations:** a Center for Global Surgery Evaluation and Department of Otolaryngology, Massachusetts Eye and Ear Infirmary, Harvard Medical School, Boston, MA, USA; b Program in Global Surgery and Social Change, Harvard Medical School, Boston, MA, USA; c Department of Plastic and Oral Surgery, Boston Children's Hospital, Harvard Medical School, Boston, MA, USA; d Department of Surgery, University of California Davis Medical Center, Davis, CA, USA; e Department Global Health and Population, Harvard TH Chan School of Public Health, Boston, MA, USA; f Department of Nursing, Michigan State University, East Lansing, MI, USA; g Malawi University of Science and Technology, Limbe, Malawi

## Abstract

**Background:**

Health-care regionalisation, in which selected services are concentrated in higher-level facilities, has successfully improved the quality of complex medical care. However, the effectiveness of this strategy in routine maternal care is unknown. Malawi has established a national goal of halving its neonatal mortality by 2030. In this study, we aimed to assess the effect of obstetric service regionalisation in pregnant women and their newborn babies in Malawi.

**Methods:**

In this analysis, we assessed regionalisation through the use of an agent-based simulation model. We used a previously estimated utilisation function, incorporating both patient-specific and health-facility-specific characteristics, to inform patient choice. The model was validated against known utilisation patterns in Malawi. Four regionalisation scenarios were compared with the status quo: scenario 1 restricted deliveries to facilities currently capable of providing caesarean sections; scenario 2 had the same restrictions as scenario 1, but with selected facilities upgraded to provide caesarean sections; scenario 3 restricted delivery to facilities that provided five or more basic emergency obstetric and neonatal care services in the preceding 3 months; and scenario 4 had the same restrictions as scenario 3, but with selected facilities upgraded to provide at least five basic emergency obstetric and neonatal care services. We assessed neonatal mortality, utilisation, travel distance, median out-of-pocket expenditure, and proportion of women facing catastrophic expenditure. The effects of upgrading the obstetric readiness of all facilities, of removing all user fees, and of upgrading without restriction were considered in scenario analyses. Heterogeneity and parameter uncertainty were incorporated to create 95% posterior credible intervals (PCIs).

**Findings:**

Scenarios restricting women to give birth in facilities with caesarean section capabilities reduced neonatal mortality by 11·4 deaths per 1000 livebirths (scenario 1; 95% PCI 9·8–13·1) and 11·6 deaths per 1000 livebirths (scenario 2; 10·2–13·1), whereas scenarios restricting women to facilities that provided five or more basic emergency obstetric and neonatal care services did not affect neonatal mortality. Similarly, the caesarean section rate in Malawi, which is 4·6% under the status quo, was predicted to rise significantly in scenario 1 (14·7%, 95% PCI 14·5–14·9; p<0·0001) and scenario 2 (10·4%, 10·2–10·6; p<0·0001), but not in scenarios 3 and 4. Women were required to travel longer distances in scenario 1 (increase of 7·2 km, 95% PCI 4·5–9·9) and in scenario 2 (4·4 km, 1·5–7·2) than in the status quo (p<0·0001). Out-of-pocket costs tripled (p<0·0001; status quo *vs* scenario 1 and scenario 2), and the risk of catastrophic expenditure significantly increased from a baseline of 6·4% (95% PCI 6·1–6·6) to 14·7% (14·5–14·9) in scenario 1 and 11·3% (11·0–11·5) in scenario 2. This increase was especially pronounced among the poor (p<0·0001; status quo *vs* scenario 1 and scenario 2).

**Interpretation:**

Policies restricting women to give birth in facilities with caesarean section capabilities is likely to result in significant decreases in neonatal mortality and might allow Malawi to meet its goal of halving its neonatal mortality by 2030. However, this improvement comes at the cost of increased distances to care and worsening financial risks among women.

**Funding:**

Bill & Melinda Gates Foundation, Damon Runyon Cancer Research Foundation.

## Introduction

Malawi has one of the highest maternal mortality rates in the world.^1^ In response to this, the Malawian Ministry of Health has organised maternal services into facilities that can provide basic emergency obstetric and neonatal care and those that can provide more comprehensive maternal care, including caesarean sections. However, many of these facilities remain understaffed and underutilised.^1^ Malawi has set a national goal of reducing neonatal mortality to 12 deaths per 1000 livebirths by 2030, down from its current level of 23 deaths per 1000 livebirths.

Improvements in clinical outcomes have been associated with increases in patient and procedure volume in specialties such as orthopaedics,^2^, ^3^ head and neck cancer,^4^ hepatic surgery,^5^ and spinal surgery.^6^ In the USA, mortality after peripartum hysterectomy for obstetric haemorrhage is significantly decreased in women who have their surgery at hospitals with high volume of procedures.^7^ These findings have led to recommendations for the regionalisation of care.

**Research in context**

**Evidence before this study**

In September 2018, *The Lancet Global Health* published the report of its Commission on high quality health systems in the Sustainable Development Goals era. Its foundational recommendation—that high-quality health systems are needed in low-income and middle-income countries to “optimise health care in each given context”—has engendered discussion around the best way to achieve these systems. Evidence exists that regionalisation of care, at least in high-income countries, might result in improved outcomes, leading to recommendations for similar policies in resource-constrained settings. We searched PubMed and Google Scholar for articles published in English up to December, 2018, using the following search terms: “regionalization”[All Fields] OR “regionalisation”[All Fields] OR “decentralization”[All Fields] OR “decentralisation”[All Fields] OR “centralization”[All Fields] OR “centralisation”[All Fields]) AND “health”[MeSH Terms]. Few studies have assessed policies of regionalisation in lower-income settings, and none have done so holistically—that is, including other outcomes of interest to patients besides their medical outcomes. Previously published extended cost-effectiveness analyses have taken a holistic approach to the evaluation of other health laws and policies, but none have done so for the regionalisation of care.

**Added value of this study**

Our study approached the question of regionalisation of care to increase access to high-quality services for pregnant women in Malawi through a holistic, patient-centred lens. We balanced the possible health outcomes gained from regionalisation with other patient-centred and health-planning outcomes (eg, distance travelled, utility, and hospital volume changes). We did so by use of an agent-based modelling technique, which allowed the inclusion of geographic information systems data, patient-level utility, and road networks into health policy analysis. We found that, although regionalisation of delivery care for pregnant Malawian women is likely to decrease the neonatal mortality rate in the country, it does so with potential detrimental consequences in other domains.

**Implications of all the available evidence**

Regionalisation strategies are likely to increase the distance women have to travel for care, the costs they have to pay for care, and their disutility. These strategies also are predicted to have sizeable effects on the staffing needs for delivery centres—staffing needs that, if they cannot be met by the Ministry of Health, will probably decrease any potential health benefits achieved by regionalisation while still exposing pregnant women to its potential negative effects.

Regionalisation has potential benefits, in addition to increasing procedure volume. It concentrates multidisciplinary specialists^8^ and might decrease access disparities and improve outcomes for patients with high-prevalence conditions such as cardiovascular disease.^9^ However, regionalisation also has its downsides: an increase in travel distance and times, with its consequent increase in costs, might impose financial burdens on patients and families and increase delays in care, potentially overcoming some of the strategy's potential benefits. In fact, although cancer care is regionalised in Ontario, Canada, this change has not led to generalised decreases in lung cancer mortality.^6^

Although few published studies of regionalisation of care in low-income and middle-income countries exist, recommendations for the regionalisation of maternal and delivery care have been made.^10^ With the publication of the report of *The Lancet Global Health*'s Commission on high-quality health systems in the Sustainable Development Goals era,^11^ increased attention is being paid to the reorganisation of health services, with a view towards maximising health outcomes rather than geographical access alone. Specifically, this Commission suggests decentralising services that require continuous and coordinated care to the primary level, while regionalising episodic services, such as delivery care, to high-quality health centres. However, evidence for or against regionalisation for more common procedures, such as obstetric delivery, is sparse, especially in low-resource settings. Additionally, a latent class analysis^12^ of obstetric care in Malawi suggested that policies to connect women to high-quality delivery services should consider the strong preference of most women for care that is close and free.

In this study, we used an agent-based model to examine the potential effects of the regionalisation of delivery care in Malawi, with specific attention paid to neonatal mortality, the redistribution of delivery services, the distance patients travel for delivery, and the effects on individual utility (we use the word utility throughout the paper in the classical economic sense; the higher a person's utility is for one choice over another reflects how much they prefer the choice with the highest utility). Our study follows a simulated closed cohort of pregnant Malawian women over one pregnancy, modelling the decision for where they choose to give birth under the status quo and under four regionalisation policies.

## Methods

### Study setting and datasets

Malawi is a country with 18·6 million people, located in southeastern Africa. Its total fertility rate of 5·5 translates to 639 000 births per year. Malawi has a life expectancy at birth of 54·8 years, an adult literacy prevalence of 61·3%, and a neonatal mortality rate of 23 deaths per 1000 livebirths. 61·6% of the Malawian population lives below the international extreme poverty line of US$1·25 per day.^13^

We constructed an agent-based model on the basis of a combined dataset of births in 2013–14 and delivery facilities; this dataset drew from the 2013–14 Malawi Millennium Development Goal Endline Survey and the 2013 Service Provision Assessment and is described in full elsewhere.^14^ Facilities where women gave birth in the dataset were modelled, including details such as global positioning system (GPS) location, facility type, facility management (private, non-governmental organisation [NGO], or public), whether they charged fees for delivery, and their basic obstetric readiness score. Readiness scores reflect the availability of equipment and resources required for obstetric care, as defined by WHO (appendix).^15^

Under the status quo, women in Malawi give birth at all levels of the health system: in central hospitals, district hospitals, rural and community hospitals, and other hospitals (primarily consisting of private and NGO hospitals), as well as in health centres, clinics, and maternities.

### Policy interventions

We assessed four regionalisation strategies in comparison with the status quo. In scenario 1, deliveries were restricted to facilities capable of providing caesarean sections. Scenario 2 is scenario 1 plus the upgrade of selected facilities without caesarean section capability to provide caesarean sections; delivery was restricted to facilities from scenario 1 plus the newly-upgraded facilities, with upgraded facilities chosen to maximise population coverage (appendix). In scenario 3, deliveries were restricted to facilities that reported doing five or more basic emergency obstetric and neonatal care procedures in the preceding 3 months. Scenario 4 is scenario 3 plus selected facilities that did not report basic emergency obstetric and neonatal care upgraded to provide such care; delivery was restricted to facilities from scenario 3 plus the newly-upgraded facilities, with upgraded facilities chosen to maximise population coverage (appendix).

Official Malawian policy was designed to promote delivery in facilities. Traditional birth attendants are barred from practice^16^ and, in reality, fewer than 10% of deliveries occur at home.^1^ As a result, home delivery was not considered in this model.

### Model design

Agent-based models are stochastic models in which each individual actor—patients and health facilities, in this case—responds to its own internal rules. Because of their stochastic nature, these models allow the incorporation of both individual-level heterogeneity and parameter uncertainty (eg, surrounding decision-related factors, such as how important distance or quality is to an individual woman). Agent-based models also allow the easy incorporation of data related to geographical information systems (GIS) such as GPS coordinates, population density, and the road network of a country. Although agent-based models were first developed for transportation, shipping, and other operations-research applications, they have been applied to modelling policies in low-income and middle-income countries in the past decade.^17^

We modelled a synthetic closed cohort of 20 000 women with GPS locations, wealth, parity, age, literacy, education, marital status, urbanicity, number of antenatal visits, multiple gestations, and predicted risky deliveries calibrated to represent a random sample of pregnant Malawian women. Wealth, GPS location, and urbanicity were modelled as a joint distribution, whereas the remaining variables were assumed to be independent of each other and of the three joint variables. Population density and poverty distribution were derived from the WorldPop project.^18^ Parity, age, literacy, education, marital status, antenatal visits, risky delivery, and multiple gestation probabilities were derived from the 2013 Millennium Development Goal Endline Survey in Malawi (National Statistical Office, Zomba, Malawi).^19^ Urbanicity was defined by residence in Blantyre or Lilongwe. Travel routes and distances were calculated with use of the A* search algorithm.^20^

Each woman's choice was modelled as a two-step process, on the basis of the choice function published by Yorlets and colleagues.^12^ The choice function was based on discrete-choice methodology applied to the women in the Millennium Development Goal Endline Survey and the 540 possible delivery facilities where they could give birth. Wealth, literacy, parity, antenatal care, predicted risk of delivery, and predicted need for caesarean section served as individual characteristics, whereas distance, indicators of facility quality, fees, and facility type served as choice-specific characteristics. Conditional logistic regression determined the relative importance of these characteristics and the latent classes that underlay individual preferences.

Each woman in our study was first probabilistically assigned to a latent preference class, on the basis of her wealth, literacy, parity, receipt of antenatal care, predicted risk of delivery, and predicted caesarean section. Conditional on membership in each latent class, the woman was matched to her most likely delivery facility. Average incomes and out-of-pocket costs for delivery were taken from previously published literature.^21^, ^22^, ^23^, ^24^ When multiple estimates were encountered, the most conservative one was used. An expense was defined as catastrophic when it was greater than 10% of a woman's yearly expenditure.^25^, ^26^

We obtained geographical information of all health facilities from the 2013–14 Service Provision Assessment. Facilities offering routine delivery services (n=540) were selected for this analysis. All delivery facilities within 100 km of a woman were included in her choice set. Factors influencing each woman's facility selection were the following: facility type, basic obstetric readiness score, road distance, and whether the facility charged fees for delivery.^12^ We calculated the probability of delivery at each facility and the woman was then assigned, probabilistically, to deliver at a single facility. The woman then travelled through Malawi's road network to her assigned facility, and neonatal mortality was then probabilistically determined (appendix). Distance to the facility and travel time to reach it, its obstetric readiness score, its type, and whether fees were charged were recorded.

### Outcomes and model validation

The status quo for maternal delivery in Malawi was modelled first to serve as validation. Results for each regionalisation strategy were then modelled and compared with the status quo. The primary outcomes of our study were neonatal mortality, the location of delivery, and distance travelled for delivery. The secondary outcome was the assessment of individual utility. Outputs of the status quo scenario were compared with the known joint distribution of wealth and location in Malawi, the distribution of maternal delivery across facility types, and the estimated preference class breakdown from Yorlets and colleagues.^12^

Because utility is not quantifiable and because valid cost data were not available, a so-called willingness to travel calculation was undertaken to make utility more concrete. Utility differences between the status quo and each regionalisation strategy were converted to the number of km that a woman would have to travel to overcome this utility difference.

Client costs were estimated on the basis of a white paper published by Partnerships or Health Reform.^21^ A woman was considered to have faced catastrophic expenses if her out-of-pocket costs equalled more than 10% of her yearly expenditures.^26^

### Scenario analyses

Because active restriction of maternal delivery to any one type of facility might be difficult to implement, we did a scenario analysis in which the selected facilities in scenarios 2 and 4 were still upgraded, but women were free to choose among all available facilities for delivery. Additionally, because obstetric readiness and user fees have previously been identified as drivers of decision making among women in Malawi,^12^ we assessed the effect on utility decrement of policies that removed direct user fees, equipped all facilities with full obstetric readiness, or did both. The goal was to assess whether these compensatory policies could overcome the predicted utility decrement that would result from requiring women to travel further for their delivery care.

To incorporate parameter uncertainty and heterogeneity, the model was run 200 times for each cohort and scenario combination, leading to 1000 runs of the model (200 for the status quo, and 200 each for the regionalisation scenarios), for a total of 20 000 000 individual patients. Modelling was done in AnyLogic (version 8.1), with analysis done in R (version 3.4.0). Results are presented as mean (95% posterior credible interval [PCI]).

### Role of the funding source

The funder of the study had no role in study design, data collection, data analysis, data interpretation, or writing of the report. The corresponding author had full access to all the data in the study and had final responsibility for the decision to submit for publication.

## Results

The actual and modelled joint distribution of wealth and location in Malawi were almost identical (p>0·99; appendix). The number and types of facilities under each regionalisation scenario are given in the appendix. Women were predicted to fall into two classes of preference, with 65·7% (95% PCI 63·8–67·6) of women falling into a class whose decision making was significantly driven by distance and user fees, and the remaining falling into a class showing a preference for delivery at a central hospital and for increased obstetric readiness among facilities. These findings are in keeping with previously published literature.^12^

Under the status quo, the model's predictions mimicked those of the available data. 65·0% (95% PCI 63·6–66·4) of women would give birth at a health centre, 18·7% (18·1–19·3) at a district hospital, 7·2% (6·7–7·6) at rural or community hospitals, 4·4% (4·0–4·8) at central hospitals, and the remaining at the other levels of facilities. Actual data show that 59·6% (95% CI 58·5–60·8) gave birth in health centres, 24·2% (23·2–25·2) in district hospitals, 8·6% (7·9–9·2) in rural and community hospitals, and 2·7% (2·3–3·1) in central hospitals. The dataset^14^ predicted that approximately 2·5% of women travelled more than 60 km for their delivery, whereas our model predicted that 3·2% of women would travel the same distance for their delivery. Finally, our model predicted a caesarean section rate of 4·1% (95% PCI 3·9–4·4), whereas Malawi's reported caesarean section rate was 4·6% in 2010.^13^

Regionalisation scenarios focused on caesarean sections (scenarios 1 and 2) significantly decreased neonatal mortality (p<0·0001 in both scenarios; table). We found no significant differences in neonatal mortality between the status quo and scenarios 3 or 4, which focused on basic emergency obstetric and neonatal care capabilities (table).

**Table:** Summary of the predicted results of each of the four regionalisation scenarios, compared with the status quo

	**Description**	**Neonatal mortality decrease, per 1000 livebirths (95% PCI)**	**Women travelling further for care, % (95% PCI)**	**Mean increased travel distance, km (95% PCI)**	Utility decrement (km required to overcome)*	**Risk of catastrophic expenditure, % chance (95% PCI)**	**Mean per-capita out-of-pocket expenditures, US$ (95% PCI)**	**Deliveries by caesarean section, % (95% PCI)**
Scenario 1	Current caesarean section hospitals	11·4 (9·8–13·1)	65·4% (64·6–66·2)	7·2 (4·5–9·9)	15·8	14·7% (14·5–14·9)	$19·59 (19·31–19·87)	14·7 (14·5–14·9)
Scenario 2	Expanded caesarean section hospitals	11·6 (10·2–13·1)	57·7% (57·1–58·3)	4·4 (1·5–7·2)	9·5	11·3% (11·0–11·5)	$14·07 (13·77–14·37)	10·4 (10·2–10·6)
Scenario 3	Current BEmONC hospitals	−1·4 (−3·0–0·2)	42·6% (42·3–42·9)	1·7 (−1·2–4·5)	3·9	7·8% (7·5–8·1)	$7·68 (7·39–7·96)	5·2 (5·0–5·4)
Scenario 4	Expanded BEmONC hospitals	−1·1 (−2·7–0·6)	37·3% (37·0–37·7)	1·1 (−1·8–4·0)	2·6	7·5% (7·2–7·8)	$7·33 (7·03–7·62)	5·0 (4·8–5·2)

PCI=posterior credible interval. BEmONC=basic emergency obstetric and neonatal care.

* The utility decrement is presented as the number of km that a woman would have to be moved closer to her delivery facility to overcome the utility difference.

**Figure 1:**
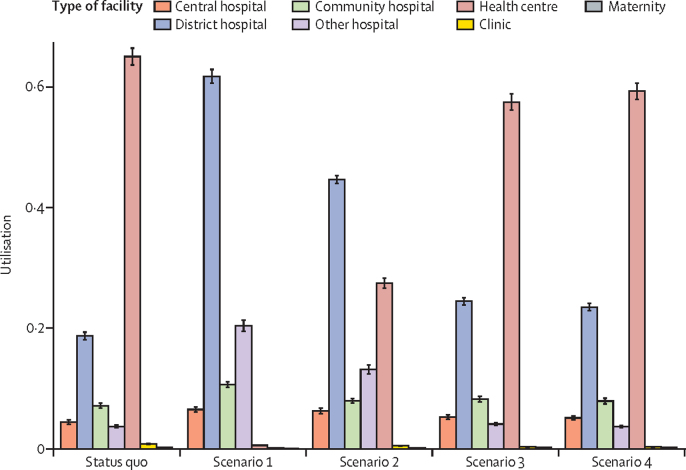
Use of delivery services under each scenario Scenario 1: restricting deliveries to facilities currently capable of providing caesarean sections. Scenario 2: restricting deliveries to current facilities capable of providing caesarean sections and to selectively upgraded facilities that previously did not have caesarean section capabilities, chosen to maximise population coverage. Scenario 3: restricting deliveries to facilities currently providing five or more basic emergency obstetric and neonatal care services. Scenario 4: restricting deliveries to facilities currently providing five or more basic emergency obstetric and neonatal care and to selectively upgraded facilities that previously did not provide five or more basic emergency obstetric and neonatal care, chosen to maximise population coverage. The y axis represents the proportion of deliveries in each scenario done in each facility type. “Other” hospitals are private and non-governmental organisation (NGO) hospitals. Error bars indicate 95% PCI.

**Figure 2:**
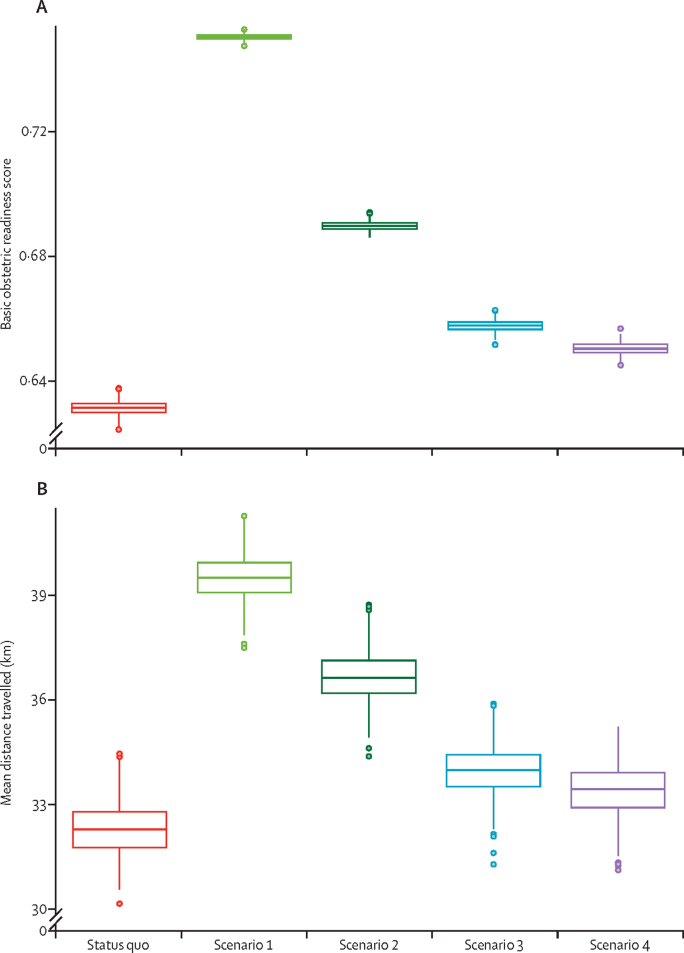
Predicted increases in the calculated obstetric readiness score of accessed facilities (A) and in travel distance (B) with each regionalisation scenario Predicted increases in the calculated obstetric readiness score of accessed facilities was weighed by the number of facilities (appendix; maximum value is 1). Scenario 1: restricting deliveries to facilities currently capable of providing caesarean sections. Scenario 2: restricting deliveries to current facilities capable of providing caesarean sections and to selectively upgraded facilities that previously did not have caesarean section capabilities, chosen to maximise population coverage. Scenario 3: restricting deliveries to facilities currently providing five or more basic emergency obstetric and neonatal care services. Scenario 4: restricting deliveries to facilities currently providing five or more basic emergency obstetric and neonatal care and to selectively upgraded facilities that previously did not provide five or more basic emergency obstetric and neonatal care, chosen to maximise population coverage. The middle line of the box represents the median, with the upper and lower lines representing the IQR; whiskers represent the entire range and dots represent outliers (less than quartile 1 or more than quartile 3 by greater than 1·5 times the IQR).

The majority of women give birth at health centres under the status quo. Scenario 1 would shift the burden of delivery care away from health centres to district and private hospitals. Under this scenario, the majority of deliveries (61·7%, 95% PCI 60·6–62·9) were predicted to occur in district hospitals (figure 1), representing an increase of approximately 275 000 deliveries in district hospitals every year. The largest relative utilisation difference was expected to occur in private and non-governmental hospitals, which would see their delivery numbers increase 5·5 times (95% PCI 5·0–6·1), or about 106 600 births. All regionalisation scenarios saw women choosing facilities with higher obstetric readiness scores (p<0·0001, figure 2A). The rate of caesarean section in Malawi was significantly increased in scenario 1 and scenario 2 (p<0·0001 for both scenarios), whereas scenarios 3 and 4 were not expected to change the caesarean section rate significantly (table). Under all regionalisation scenarios, women were expected to travel further for delivery than they would have under the status quo, although these changes were not significant in scenarios 3 and 4 (table; figure 2B). No significant difference was predicted in the proportion of women travelling more than 60 km for delivery (data not shown).

When compared with the status quo, regionalisation scenarios were predicted to decrease women's utility, despite using facilities with better obstetric readiness (table). This implies that, if they were allowed a choice, they would still prefer a setting of decentralised delivery care. In all scenarios, this utility difference was significantly greater among the poorest and least literate segment of the population.

**Figure 3:**
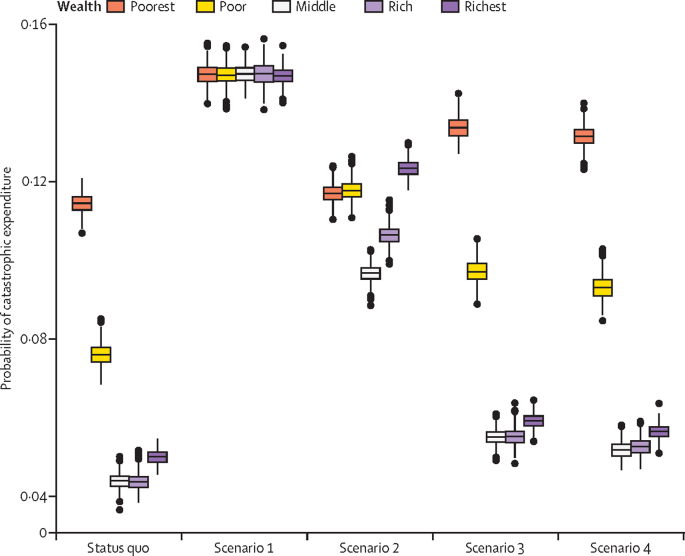
Predicted risk of catastrophic expenditure under each regionalisation scenario Scenario 1: restricting deliveries to facilities currently capable of providing caesarean sections. Scenario 2: restricting deliveries to current facilities capable of providing caesarean sections and to selectively upgraded facilities that previously did not have caesarean section capabilities, chosen to maximise population coverage. Scenario 3: restricting deliveries to facilities currently providing five or more basic emergency obstetric and neonatal care services. Scenario 4: restricting deliveries to facilities currently providing five or more basic emergency obstetric and neonatal care and to selectively upgraded facilities that previously did not provide five or more basic emergency obstetric and neonatal care, chosen to maximise population coverage. The middle line of the box represents the median, with the upper and lower lines representing the IQR; whiskers represent the entire range and dots represent outliers (less than quartile 1 or more than quartile 3 by greater than 1·5 times the IQR).

Under the status quo, approximately 6·4% (95% PCI 6·1–6·6) of women face catastrophic expense for giving birth, in line with other published estimates.^24^ This number was predicted to rise significantly under scenarios 1 and 2 (table; p<0·0001). Scenarios 1 and 2 worsened financial risk for the entire population, whereas scenarios 3 and 4 worsened financial risk for the poorest 40% of the population (figure 3). Mean per-capita out-of-pocket expenditure tripled under scenario 1, from a baseline of US$6·19 (95% PCI 5·91–6·48) to $19·59 (19·31–19·87) and doubled under scenario 2 (table). Such increases are substantial, given that the average yearly consumption expenditure in Malawi is $137.^24^

We examined scenarios in which selected facilities were upgraded to have caesarean section capabilities or basic emergency obstetric and neonatal care capabilities (as in scenarios 2 and 4), but did not restrict women to give birth in these facilities. The beneficial effects on neonatal mortality disappeared in these scenarios. We also examined potential policies to overcome the utility decrement predicted to occur in regionalisation strategies, such as the removal of direct user fees, the equipping of all facilities with full obstetric readiness, or both. We found that no policy was sufficient to overcome the population-level utility decrement predicted to follow regionalisation strategies.

## Discussion

In our study, we assessed the predicted effect of regionalisation of delivery care in Malawi. We found that focusing on basic emergency obstetric and neonatal care capabilities is insufficient to improve neonatal mortality significantly. Mortality was reduced significantly and the caesarean section rate was brought closer to published recommendations^27^ when women were restricted to giving birth in facilities with caesarean section capabilities; upgrading facilities without restricting where women could give birth negated these improvements.

Up to two-thirds of women in Malawi would travel further for delivery under regionalisation scenarios, with a significant increase in the mean distance travelled in scenario 1. For this increased distance, women would access facilities of substantially higher structural quality. However, because of the increased distance, regionalisation was associated with a predicted increase in disutility among pregnant women, primarily among the poor and less educated, who make up two-thirds of the population in Malawi.^12^ This utility decrement was not predicted to be overcome by making delivery free or by further increasing the obstetric readiness at the regionalised centres. Because women's utility is strongly dependent on distance, policies aimed at decreasing the transportation barrier that women face might improve utility.

Workforce implications of regionalisation strategies are also likely to exist. Strategies providing the greatest reductions in neonatal mortality would cause a substantial shift in maternal delivery away from health centres and towards district hospitals and the private and non-governmental sectors. Although these sectors are often contracted by the Malawian Ministry of Health, their workforce and infrastructure might be less well regulated by the ministry. Additionally, the increase in predicted caesarean sections is likely to require a bolstering of the workforce capable of doing these procedures.

Previous evidence has shown that most Malawian women choose where to give birth on the basis of the distance and whether fees are charged.^12^ Access to transportation in Malawi is difficult, and many women do not have help from their partner.^28^ In a study^29^ published in 2015, mothers of deceased newborn babies reported distance and lack of access to transportation to be the most important barriers to seeking care. In Malawi, the poorest women live the furthest from large population centres (appendix), suggesting that pregnant women who need the most resources to access a far away, high-quality facility have the least capacity to do so. Additionally, an association exists between better facilities and large population centres, with the highest structural readiness found in facilities around Lilongwe and Blantyre, where the richest women live (appendix).

Existing studies have shown that women who live further away from health facilities are less likely to give birth assisted by health professionals or in a facility.^30^, ^31^, ^32^ Increased travel distance for delivery care might result in worse maternal outcomes, although data are inconsistent. In rural Tanzania, increased distance from a hospital was associated with increased maternal mortality from direct, delivery-associated causes.^33^ A similar trend was found in Bangladesh and Indonesia for deliveries assisted by health professionals, with the odds of maternal death increasing with increasing distance from a health centre.^32^ However, this pattern was not evident for deliveries in hospitals or not assisted by a health professional. In both studies, an increase in the odds ratio of maternal mortality was associated with increased distance.^32^, ^33^ However, In Burkina Faso, no association was found between increased distance to a health facility and maternal death.^31^ Overall, results are more consistent in the neonatal mortality literature, showing an increase in neonatal mortality with increased distance from health facilities.^34^ However, not all studies,^35^, ^36^ including one done in Malawi, show this relationship.

Although transportation improvements are likely to yield greater gains in access to quality care than facility expansion or caesarean section capability upgrades, the current Malawian national health policy does not discuss transportation in its intersectoral plan.

As with all models, our study has some limitations. Because the Malawian road network was incorporated into this model, distance travelled for most women is road-based. However, for some women (particularly those who live on the Likoma and Chizumulu islands), routing algorithms do not exist and Euclidean, straight-line distances had to be substituted. This substitution is likely to have the effect of lowering the calculated distance travelled to care, implying that the distances calculated in our study are likely to be underestimated. Although we used a global reference measure of obstetric service readiness, its relationship to perceived quality among women is unknown. As such, our conclusion that improved readiness could not overcome the utility decrement produced by regionalisation scenarios should be interpreted with caution, especially because evidence from other settings has shown an increase in satisfaction among patients who bypassed lower-level facilities.^37^ The actual amount of money charged at any of the delivery centres was not available in the dataset; all that was available was a binary indication of whether fees were charged. As such, the only policy lever that could be simulated was the complete elimination of user fees for delivery. The utility function used comes from previously published research.^12^ Importantly, the function does not take the disutility of neonatal mortality into account, which means that our estimates of disutility might be inflated. Finally, to account for differential risk, neonatal mortality was predicted on the basis of an instrumental variable regression (appendix); therefore, mortality results should be interpreted with caution.

Because of an absence of data to predict the changes in facilities that result from policy interventions, the time horizon for this study was limited to one delivery. This limit introduces an assumption that the current mortality estimates, derived from the published literature,^38^ will remain consistent in the face of increased demand. Unless facility capacity, in both physical infrastructure and human capital, increases to meet this demand, neonatal mortality benefits might not reach the levels predicted in this paper.

Mathematical models are valuable for large-scale, population-based policy research, given the limited feasibility of randomised controlled trials in these settings. Agent-based models such as this one have been increasingly used to answer policy-relevant questions in global health.^17^, ^39^, ^40^ Our model specifically allows for the predicted effects—both beneficial and detrimental—of regionalisation for pregnant women, their children, and the health system itself to be made explicit before the implementation of such a policy. Regarding regionalisation of maternal delivery services, the predicted increase in demand at district hospitals, along with the predicted outsourcing of demand to the private and non-governmental sectors, might aid policy makers in targeting the scale-up and distribution of the workforce. Additionally, although delivery at home in Malawi is discouraged, the increased disutility due to regionalisation might result in an increase in home deliveries, for which the ministry should be prepared. Finally, policies that improve access to higher-quality services, such as travel vouchers and an improved pre-hospital system, should be considered, especially for the poor and less-educated members of the population.

Although the regionalisation of services has been used successfully to improve the quality of complex care, its implementation for more routine care is not without its downsides. Restricting women to give birth in hospitals with caesarean section capability could improve neonatal mortality and caesarean section rate in the country, but is expected to cause a three-times higher use of delivery services in district hospitals, with nearly six-times higher use for private and non-governmental hospitals. In doing so, the policy is expected to increase the distance travelled for delivery and the risk of financial catastrophe due to the delivery. This policy is also likely to decrease patient utility, especially among the poor and less educated, an effect that could not be overcome by removing user fees for delivery or by maximising hospital readiness. Regionalisation strategies focusing only on basic obstetric care are likely to result in minimal reductions in mortality, while still requiring women to travel further for their care. The expected benefits of regionalisation should be weighed against these potential staff-related and patient-related consequences, and regionalisation policies should incorporate measures such as improved transportation and increased protection against financial risk to mitigate the potential inequity that they generate.
