# Supplementary material for: Predicted effect of regionalised delivery care on neonatal mortality, utilisation, financial risk, and patient utility in Malawi: an agent-based modelling analysis
Source: Lancet Glob Health. 2019 Jun 11;7(7):e932–9. doi: 10.1016/S2214-109X(19)30170-6 (PMC6581692; doi:10.1016/S2214-109X(19)30170-6)
Supplement: Supplementary appendix [file mmc1.pdf]

THE LANCET
Global Health

Supplementary appendix

This appendix formed part of the original submission and has been peer reviewed. We post it as supplied by the authors.

Supplement to: Shrime MG, Iverson KR, Yorlets R, et al. Predicted effect of regionalised delivery care on neonatal mortality, utilisation, financial risk, and patient utility in Malawi: an agent-based modelling analysis. *Lancet Glob Health* 2019; **7:** e932–39.

Section 1: Obstetric readiness score

The obstetric readiness score was calculated as an average score across three domains, each item in a domain assigned 1 if observed and functional during 2013 facility assessment

**Domain 1: Guidelines**
Guidelines for Integrated management of pregnancy and childbirth (IMPAC)
Staff trained in IMPAC
**Domain 2: Infrastructure**
Emergency transport
Sterilization equipment
Examination light
Delivery pack
Suction apparatus (mucus extractor)
Manual vacuum extractor
Vacuum aspirator or D&C kit
Neonatal bag and mask
Delivery bed
Partograph
Gloves
**Domain 3: Medications**
Antibiotic Eye Ointment for newborn
Injectable uterotonic
Injectable antibiotic
Injectable magnesium sulphate
Skin disinfectant
IV solution with infusion set

Section 2: BeMONC Procedures

Facilities were considered BeMONC capable if they provided five or more of: Assisted vaginal delivery, removal of retained products of conception, parenteral oxytocin for hemorrhage, parenteral magnesium sulfate for (pre-)eclampsia, manual removal of placenta, antibiotics for maternal infection, and newborn resuscitation

Section 3: Population coverage of delivery services

Adapted from Gage et al(1)

We developed a cost surface model to estimate the travel cost (i.e. the travel time in seconds) per pixel using digital road network, elevation, and land cover data. We created two surfaces for driving and walking, then overlaid them with the assumption that women would walk to the nearest road and then take motorized transport. For the driving surface, speeds were classified based on the road type; unknown road types were classified to match the closest known road type. The walking surface was created from slope and land cover coefficients with a baseline walking speed of 4 km/hour. The cost surface was created using ArcGIS 10.4.1 software (ESRI,

Redlands, CA). Pregnancy distribution data is from WorldPop, which estimates the number of annual pregnancies per square kilometer grid cell, with national totals adjusted to match estimates from the Guttmacher Institute.(2, 3) Road network data were downloaded in Spring 2017 from Geofabrik's free download server (https://www.geofabrik.de/), which is updated daily based on modifications to the OpenStreetMap project. The SRTM Digital Elevation Dataset, with a resolution of three arc seconds (approx. 90m), was used to generate slope angles. Lastly, GlobCover 2009 Land Cover data was used to estimate the travel cost to roads.

We estimated the cumulative travel time from each pixel to the nearest facility offering delivery services under each scenario using the Cost Distance Tool in ArcGIS. Due to inconsistencies in the geocodes, all facilities less than one kilometer from the nearest road were re-located to be on the nearest road. We generated a facility catchment area within one or two hours of the facilities using the Zonal Statistics tool and summed the pregnant women within the catchment area. We divided the percent of pregnant women living in the catchment area by the total pregnant women to estimate the percent living within one or two hours.

Pregnancies within one or two hours of delivery services under regionalization (N= 1,007,931)

	Pregnancies within 1 hour		Pregnancies within 2 hours	
	N	%	N	%
0. Status Quo	925,334	92%	959,468	95%
1. Only CS	849,384	84%	952,428	95%
2. Upgrade CS	897,686	89%	957,761	95%
3. Only BEMC	910,574	90%	958,531	95%
4. Upgrade BEMC	919,836	91%	959,187	95%

Section 4: Distribution of facilities under model strategies

	Current delivery facilities (540)		Current c-section (69)		Expanded c-section (125)		Current BEmONC capable (272)		Expanded BEmONC capable (321)	
	N	%	N	%	N	%	N	%	N	%
Central hospital	4	0.74	4	5.8	4	3.2	4	1.47	4	1.25
District hospital	24	4.44	23	33.33	23	18.4	24	8.82	24	7.48
Other hospital	29	5.37	25	36.23	25	20	20	7.35	20	6.23
Rural / community hospital	41	7.59	13	18.84	16	12.8	35	12.87	36	11.21
Health center	420	77.78	1	1.45	52	41.6	184	67.65	232	72.27
Clinic	18	3.33	3	4.35	5	4	4	1.47	4	1.25
Maternity	4	0.74	0	0	0	0	1	0.37	1	0.31

Section 5: Predicted neonatal mortality

We used a previously published instrumental variable model(4) to estimate neonatal mortality based on delivery facility type incorporating selection of risky deliveries into higher quality facilities. The previous analysis estimated neonatal mortality based on a quality index combining readiness to provide care with reported BEmONC procedures; we calculated average performance on this quality index for each tier of facilities considered in the current analysis and predicted mortality setting all facilities to this level of quality. Average quality, predicted mortality, and number of facilities used in the prediction for each type of facility considered in the current analysis are shown below. Because the instrumental variable model used a linear form for mortality, some tiers of facility resulted in negative mortality; expected mortality was set to 0 for these tiers.

Predicted neonatal mortality by facility type and c-section / BEmONC capacity

	Facilities (N=540)	Quality score (standardized)	Estimated 28-day mortality	95% CI (for estimate)	
With c section					
Central hospital	4	1.8901	-0.0065	-0.0304	0.0174
District hospital	23	1.7177	-0.0019	-0.0212	0.0174
Other hospital	25	1.5160	0.0036	-0.0104	0.0176
community hospital	13	1.4953	0.0041	-0.0093	0.0176
Health center	1	1.8465	-0.0054	-0.0281	0.0174
Clinic	3	1.2708	0.0102	0.0025	0.0179
Maternity	0				
Without c section					
Central hospital	0				
District hospital	1	1.8465	-0.0054	-0.0281	0.0174
Other hospital	4	1.1496	0.0135	0.0085	0.0185
community hospital	28	1.1971	0.0122	0.0062	0.0182
Health center	419	0.5447	0.0298	0.0167	0.0429
Clinic	15	0.2646	0.0374	0.0168	0.0579
Maternity	4	0.2946	0.0366	0.0168	0.0563
BEmONC capable (5+ SF)					
Central hospital	4	1.8901	-0.0065	-0.0304	0.0174
District hospital	24	1.7231	-0.0020	-0.0215	0.0174
Other hospital	20	1.7341	-0.0023	-0.0221	0.0174
community hospital	35	1.3519	0.0080	-0.0017	0.0178
Health center	184	0.8963	0.0203	0.0157	0.0250

Clinic	4	0.9086	0.0200	0.0155	0.0244
Maternity	1	0.9754	0.0182	0.0146	0.0217
Not BEmONC capable (<5 SF)					
Central hospital	0				
District hospital	0				
Other hospital	9	0.8702	0.0210	0.0158	0.0262
community hospital	6	0.9404	0.0191	0.0152	0.0231
Health center	236	0.2762	0.0371	0.0168	0.0573
Clinic	14	0.2917	0.0366	0.0168	0.0565
Maternity	3	0.1243	0.0412	0.0169	0.0654

Section 6: Java code

```
package malawi_recentralization_model_2017_06_04;

import java.io.Serializable;
import java.sql.Connection;
import java.sql.SQLException;
import java.util.ArrayDeque;
import java.util.ArrayList;
import java.util.Arrays;
import java.util.Calendar;
import java.util.Collection;
import java.util.Collections;
import java.util.Comparator;
import java.util.Currency;
import java.util.Date;
import java.util.Enumeration;
import java.util.HashMap;
import java.util.HashSet;
import java.util.Hashtable;
import java.util.Iterator;
import java.util.LinkedHashMap;
import java.util.LinkedHashSet;
import java.util.LinkedList;
import java.util.List;
import java.util.ListIterator;
import java.util.Locale;
import java.util.Map;
import java.util.PriorityQueue;
import java.util.Random;
import java.util.Set;
import java.util.SortedMap;
import java.util.SortedSet;
import java.util.Stack;
import java.util.Timer;
import java.util.TreeMap;
import java.util.TreeSet;
import java.util.Vector;
import java.awt.Color;
import java.awt.Font;
import java.awt.Graphics2D;
import java.awt.geom.AffineTransform;
import com.anylogic.engine.connectivity.ResultSet;
```

```
import com.anylogic.engine.connectivity.Statement;
import com.anylogic.engine.elements.*;
import com.anylogic.engine.markup.Network;
import com.anylogic.engine.Position;
import com.anylogic.engine.markup.PedFlowStatistics;
import com.anylogic.engine.markup.DensityMap;


import static java.lang.Math.*;
import static com.anylogic.engine.UtilitiesArray.*;
import static com.anylogic.engine.UtilitiesCollection.*;
import static com.anylogic.engine.presentation.UtilitiesColor.*;
import static com.anylogic.engine.HyperArray.*;

import com.anylogic.engine.*;
import com.anylogic.engine.analysis.*;
import com.anylogic.engine.connectivity.*;
import com.anylogic.engine.database.*;
import com.anylogic.engine.gis.*;
import com.anylogic.engine.markup.*;
import com.anylogic.engine.presentation.*;

import com.mysema.query.Tuple;
import com.mysema.query.sql.SQLBindings;
import static malawi_recentralization_model_2017_06_04.DBDescriptor.*;

import java.awt.geom.Arc2D;

import java.sql.Time;
import org.uncommons.maths.random.MersenneTwisterRNG;
import org.uncommons.maths.random.GaussianGenerator;

public class Main extends Agent
{
  // Excel Files
  public ExcelFile distanceWTP = new ExcelFile( Main.this, 
"/malawi_recentralization_model_2017_06_04/", "/Users/Mark/Dropbox/@Papers/HQSS/Model 
output/Distance WTP calculations.xlsx", false );
  public ExcelFile newClassAssignment = new ExcelFile( Main.this, 
"/malawi_recentralization_model_2017_06_04/", "/Users/Mark/Dropbox/@Papers/HQSS/Model 
output/New Class Assignment.xlsx", false );

  // Parameters

  public 
boolean  allowFeesParam;

  /**
   * Returns default value for parameter <code>allowFeesParam</code>.
   * <i>This method should not be called by user</i>
   */
  @AnyLogicInternalCodegenAPI
  public boolean _allowFeesParam_DefaultValue_xjal() {
    final Main self = this;
    return 
true
;
  }

  public void set_allowFeesParam( boolean allowFeesParam ) {
    if (allowFeesParam == this.allowFeesParam) {
      return;
    }
```

```
    boolean _oldValue_xjal = this.allowFeesParam;
    this.allowFeesParam = allowFeesParam;
    onChange_allowFeesParam_xjal( _oldValue_xjal );
    onChange();
  }

  /**
   * Calls "On change" action for parameter allowFeesParam.<br>
   * Note that 'oldValue' in that action will be unavailable if this method is called
by user
   * (current parameter value will be passed as 'oldValue').<br>
   * Please call <code>set_allowFeesParam()</code> method instead.
   */
  protected void onChange_allowFeesParam() {
    onChange_allowFeesParam_xjal( allowFeesParam );
  }

  @AnyLogicInternalCodegenAPI
  protected void onChange_allowFeesParam_xjal( boolean oldValue ) {
  }


  public
double  centralHosp1;

  /**
   * Returns default value for parameter <code>centralHosp1</code>.
   * <i>This method should not be called by user</i>
   */
  @AnyLogicInternalCodegenAPI
  public double _centralHosp1_DefaultValue_xjal() {
    final Main self = this;
    return
0
;
  }

  public void set_centralHosp1( double centralHosp1 ) {
    if (centralHosp1 == this.centralHosp1) {
      return;
    }
    double _oldValue_xjal = this.centralHosp1;
    this.centralHosp1 = centralHosp1;
    onChange_centralHosp1_xjal( _oldValue_xjal );
    onChange();
  }

  /**
   * Calls "On change" action for parameter centralHosp1.<br>
   * Note that 'oldValue' in that action will be unavailable if this method is called
by user
   * (current parameter value will be passed as 'oldValue').<br>
   * Please call <code>set_centralHosp1()</code> method instead.
   */
  protected void onChange_centralHosp1() {
    onChange_centralHosp1_xjal( centralHosp1 );
  }

  @AnyLogicInternalCodegenAPI
  protected void onChange_centralHosp1_xjal( double oldValue ) {
  }
```

```
  public
double  distance1;

  /**
   * Returns default value for parameter <code>distance1</code>.
   * <i>This method should not be called by user</i>
   */
  @AnyLogicInternalCodegenAPI
  public double _distance1_DefaultValue_xjal() {
    final Main self = this;
    return
(double) selectFrom(parameters)
       .where(parameters.parameter.eq("class.1.dist"))
       .uniqueResult(parameters.starting_value)
;
  }

  public void set_distance1( double distance1 ) {
    if (distance1 == this.distance1) {
      return;
    }
    double _oldValue_xjal = this.distance1;
    this.distance1 = distance1;
    onChange_distance1_xjal( _oldValue_xjal );
    onChange();
  }

  /**
   * Calls "On change" action for parameter distance1.<br>
   * Note that 'oldValue' in that action will be unavailable if this method is called
by user
   * (current parameter value will be passed as 'oldValue').<br>
   * Please call <code>set_distance1()</code> method instead.
   */
  protected void onChange_distance1() {
    onChange_distance1_xjal( distance1 );
  }

  @AnyLogicInternalCodegenAPI
  protected void onChange_distance1_xjal( double oldValue ) {
  }


  public
double  clinic1;

  /**
   * Returns default value for parameter <code>clinic1</code>.
   * <i>This method should not be called by user</i>
   */
  @AnyLogicInternalCodegenAPI
  public double _clinic1_DefaultValue_xjal() {
    final Main self = this;
    return
(double) selectFrom(parameters)
       .where(parameters.parameter.eq("class.1.spaClinic"))
       .uniqueResult(parameters.starting_value)
;
  }

  public void set_clinic1( double clinic1 ) {
    if (clinic1 == this.clinic1) {
      return;
```

```
    }
    double _oldValue_xjal = this.clinic1;
    this.clinic1 = clinic1;
    onChange_clinic1_xjal( _oldValue_xjal );
    onChange();
  }

  /**
   * Calls "On change" action for parameter clinic1.<br>
   * Note that 'oldValue' in that action will be unavailable if this method is called
by user
   * (current parameter value will be passed as 'oldValue').<br>
   * Please call <code>set_clinic1()</code> method instead.
   */
  protected void onChange_clinic1() {
    onChange_clinic1_xjal( clinic1 );
  }

  @AnyLogicInternalCodegenAPI
  protected void onChange_clinic1_xjal( double oldValue ) {
  }


  public
double  maternity1;

  /**
   * Returns default value for parameter <code>maternity1</code>.
   * <i>This method should not be called by user</i>
   */
  @AnyLogicInternalCodegenAPI
  public double _maternity1_DefaultValue_xjal() {
    final Main self = this;
    return
(double) selectFrom(parameters)
      .where(parameters.parameter.eq("class.1.spaMaternity"))
      .uniqueResult(parameters.starting_value)
;
  }

  public void set_maternity1( double maternity1 ) {
    if (maternity1 == this.maternity1) {
      return;
    }
    double _oldValue_xjal = this.maternity1;
    this.maternity1 = maternity1;
    onChange_maternity1_xjal( _oldValue_xjal );
    onChange();
  }

  /**
   * Calls "On change" action for parameter maternity1.<br>
   * Note that 'oldValue' in that action will be unavailable if this method is called
by user
   * (current parameter value will be passed as 'oldValue').<br>
   * Please call <code>set_maternity1()</code> method instead.
   */
  protected void onChange_maternity1() {
    onChange_maternity1_xjal( maternity1 );
  }

  @AnyLogicInternalCodegenAPI
  protected void onChange_maternity1_xjal( double oldValue ) {
```

```
  }


  public
double  healthCenter1;

  /**
   * Returns default value for parameter <code>healthCenter1</code>.
   * <i>This method should not be called by user</i>
   */
  @AnyLogicInternalCodegenAPI
  public double _healthCenter1_DefaultValue_xjal() {
    final Main self = this;
    return
(double) selectFrom(parameters)
       .where(parameters.parameter.eq("class.1.spaHeh center"))
       .uniqueResult(parameters.starting_value)
;
  }

  public void set_healthCenter1( double healthCenter1 ) {
    if (healthCenter1 == this.healthCenter1) {
      return;
    }
    double _oldValue_xjal = this.healthCenter1;
    this.healthCenter1 = healthCenter1;
    onChange_healthCenter1_xjal( _oldValue_xjal );
    onChange();
  }

  /**
   * Calls "On change" action for parameter healthCenter1.<br>
   * Note that 'oldValue' in that action will be unavailable if this method is called
by user
   * (current parameter value will be passed as 'oldValue').<br>
   * Please call <code>set_healthCenter1()</code> method instead.
   */
  protected void onChange_healthCenter1() {
    onChange_healthCenter1_xjal( healthCenter1 );
  }

  @AnyLogicInternalCodegenAPI
  protected void onChange_healthCenter1_xjal( double oldValue ) {
  }


  public
double  otherHosp1;

  /**
   * Returns default value for parameter <code>otherHosp1</code>.
   * <i>This method should not be called by user</i>
   */
  @AnyLogicInternalCodegenAPI
  public double _otherHosp1_DefaultValue_xjal() {
    final Main self = this;
    return
(double) selectFrom(parameters)
       .where(parameters.parameter.eq("class.1.spaOther hospital"))
       .uniqueResult(parameters.starting_value)
;
  }
```

```
  public void set_otherHosp1( double otherHosp1 ) {
    if (otherHosp1 == this.otherHosp1) {
      return;
    }
    double _oldValue_xjal = this.otherHosp1;
    this.otherHosp1 = otherHosp1;
    onChange_otherHosp1_xjal( _oldValue_xjal );
    onChange();
  }

  /**
   * Calls "On change" action for parameter otherHosp1.<br>
   * Note that 'oldValue' in that action will be unavailable if this method is called
by user
   * (current parameter value will be passed as 'oldValue').<br>
   * Please call <code>set_otherHosp1()</code> method instead.
   */
  protected void onChange_otherHosp1() {
    onChange_otherHosp1_xjal( otherHosp1 );
  }

  @AnyLogicInternalCodegenAPI
  protected void onChange_otherHosp1_xjal( double oldValue ) {
  }


  public
double  communityHosp1;

  /**
   * Returns default value for parameter <code>communityHosp1</code>.
   * <i>This method should not be called by user</i>
   */
  @AnyLogicInternalCodegenAPI
  public double _communityHosp1_DefaultValue_xjal() {
    final Main self = this;
    return
(double) selectFrom(parameters)
      .where(parameters.parameter.eq("class.1.spaCommunity hospital"))
      .uniqueResult(parameters.starting_value)
;
  }

  public void set_communityHosp1( double communityHosp1 ) {
    if (communityHosp1 == this.communityHosp1) {
      return;
    }
    double _oldValue_xjal = this.communityHosp1;
    this.communityHosp1 = communityHosp1;
    onChange_communityHosp1_xjal( _oldValue_xjal );
    onChange();
  }

  /**
   * Calls "On change" action for parameter communityHosp1.<br>
   * Note that 'oldValue' in that action will be unavailable if this method is called
by user
   * (current parameter value will be passed as 'oldValue').<br>
   * Please call <code>set_communityHosp1()</code> method instead.
   */
  protected void onChange_communityHosp1() {
    onChange_communityHosp1_xjal( communityHosp1 );
  }
```

```
  @AnyLogicInternalCodegenAPI
  protected void onChange_communityHosp1_xjal( double oldValue ) {
  }


  public
double  districtHosp1;

  /**
   * Returns default value for parameter <code>districtHosp1</code>.
   * <i>This method should not be called by user</i>
   */
  @AnyLogicInternalCodegenAPI
  public double _districtHosp1_DefaultValue_xjal() {
    final Main self = this;
    return
(double) selectFrom(parameters)
      .where(parameters.parameter.eq("class.1.spaDistrict hospital"))
      .uniqueResult(parameters.starting_value)
;
  }

  public void set_districtHosp1( double districtHosp1 ) {
    if (districtHosp1 == this.districtHosp1) {
      return;
    }
    double _oldValue_xjal = this.districtHosp1;
    this.districtHosp1 = districtHosp1;
    onChange_districtHosp1_xjal( _oldValue_xjal );
    onChange();
  }

  /**
   * Calls "On change" action for parameter districtHosp1.<br>
   * Note that 'oldValue' in that action will be unavailable if this method is called
by user
   * (current parameter value will be passed as 'oldValue').<br>
   * Please call <code>set_districtHosp1()</code> method instead.
   */
  protected void onChange_districtHosp1() {
    onChange_districtHosp1_xjal( districtHosp1 );
  }

  @AnyLogicInternalCodegenAPI
  protected void onChange_districtHosp1_xjal( double oldValue ) {
  }


  public
double  bobs1;

  /**
   * Returns default value for parameter <code>bobs1</code>.
   * <i>This method should not be called by user</i>
   */
  @AnyLogicInternalCodegenAPI
  public double _bobs1_DefaultValue_xjal() {
    final Main self = this;
    return
(double) selectFrom(parameters)
      .where(parameters.parameter.eq("class.1.bobs"))
      .uniqueResult(parameters.starting_value)
```

```
;
  }

  public void set_bobs1( double bobs1 ) {
    if (bobs1 == this.bobs1) {
      return;
    }
    double _oldValue_xjal = this.bobs1;
    this.bobs1 = bobs1;
    onChange_bobs1_xjal( _oldValue_xjal );
    onChange();
  }

  /**
   * Calls "On change" action for parameter bobs1.<br>
   * Note that 'oldValue' in that action will be unavailable if this method is called
by user
   * (current parameter value will be passed as 'oldValue').<br>
   * Please call <code>set_bobs1()</code> method instead.
   */
  protected void onChange_bobs1() {
    onChange_bobs1_xjal( bobs1 );
  }

  @AnyLogicInternalCodegenAPI
  protected void onChange_bobs1_xjal( double oldValue ) {
  }


  public
double  fees1;

  /**
   * Returns default value for parameter <code>fees1</code>.
   * <i>This method should not be called by user</i>
   */
  @AnyLogicInternalCodegenAPI
  public double _fees1_DefaultValue_xjal() {
    final Main self = this;
    return
(double) selectFrom(parameters)
       .where(parameters.parameter.eq("class.1.fees"))
       .uniqueResult(parameters.starting_value)
;
  }

  public void set_fees1( double fees1 ) {
    if (fees1 == this.fees1) {
      return;
    }
    double _oldValue_xjal = this.fees1;
    this.fees1 = fees1;
    onChange_fees1_xjal( _oldValue_xjal );
    onChange();
  }

  /**
   * Calls "On change" action for parameter fees1.<br>
   * Note that 'oldValue' in that action will be unavailable if this method is called
by user
   * (current parameter value will be passed as 'oldValue').<br>
   * Please call <code>set_fees1()</code> method instead.
   */
```

```
  protected void onChange_fees1() {
    onChange_fees1_xjal( fees1 );
  }

  @AnyLogicInternalCodegenAPI
  protected void onChange_fees1_xjal( double oldValue ) {
  }


  public
boolean  allowAllParam;

  /**
   * Returns default value for parameter <code>allowAllParam</code>.
   * <i>This method should not be called by user</i>
   */
  @AnyLogicInternalCodegenAPI
  public boolean _allowAllParam_DefaultValue_xjal() {
    final Main self = this;
    return
true
;
  }

  public void set_allowAllParam( boolean allowAllParam ) {
    if (allowAllParam == this.allowAllParam) {
      return;
    }
    boolean _oldValue_xjal = this.allowAllParam;
    this.allowAllParam = allowAllParam;
    onChange_allowAllParam_xjal( _oldValue_xjal );
    onChange();
  }

  /**
   * Calls "On change" action for parameter allowAllParam.<br>
   * Note that 'oldValue' in that action will be unavailable if this method is called
by user
   * (current parameter value will be passed as 'oldValue').<br>
   * Please call <code>set_allowAllParam()</code> method instead.
   */
  protected void onChange_allowAllParam() {
    onChange_allowAllParam_xjal( allowAllParam );
  }

  @AnyLogicInternalCodegenAPI
  protected void onChange_allowAllParam_xjal( boolean oldValue ) {
  }


  public
double  intercept;

  /**
   * Returns default value for parameter <code>intercept</code>.
   * <i>This method should not be called by user</i>
   */
  @AnyLogicInternalCodegenAPI
  public double _intercept_DefaultValue_xjal() {
    final Main self = this;
    return
(double) selectFrom(parameters)
      .where(parameters.parameter.eq("Intercept"))
```

```
      .uniqueResult(parameters.starting_value)
;
  }

  public void set_intercept( double intercept ) {
    if (intercept == this.intercept) {
      return;
    }
    double _oldValue_xjal = this.intercept;
    this.intercept = intercept;
    onChange_intercept_xjal( _oldValue_xjal );
    onChange();
  }

  /**
   * Calls "On change" action for parameter intercept.<br>
   * Note that 'oldValue' in that action will be unavailable if this method is called
by user
   * (current parameter value will be passed as 'oldValue').<br>
   * Please call <code>set_intercept()</code> method instead.
   */
  protected void onChange_intercept() {
    onChange_intercept_xjal( intercept );
  }

  @AnyLogicInternalCodegenAPI
  protected void onChange_intercept_xjal( double oldValue ) {
  }


  public
double  centralHosp2;

  /**
   * Returns default value for parameter <code>centralHosp2</code>.
   * <i>This method should not be called by user</i>
   */
  @AnyLogicInternalCodegenAPI
  public double _centralHosp2_DefaultValue_xjal() {
    final Main self = this;
    return
0
;
  }

  public void set_centralHosp2( double centralHosp2 ) {
    if (centralHosp2 == this.centralHosp2) {
      return;
    }
    double _oldValue_xjal = this.centralHosp2;
    this.centralHosp2 = centralHosp2;
    onChange_centralHosp2_xjal( _oldValue_xjal );
    onChange();
  }

  /**
   * Calls "On change" action for parameter centralHosp2.<br>
   * Note that 'oldValue' in that action will be unavailable if this method is called
by user
   * (current parameter value will be passed as 'oldValue').<br>
   * Please call <code>set_centralHosp2()</code> method instead.
   */
  protected void onChange_centralHosp2() {
```

```
    onChange_centralHosp2_xjal( centralHosp2 );
  }

  @AnyLogicInternalCodegenAPI
  protected void onChange_centralHosp2_xjal( double oldValue ) {
  }


  public
double  distance2;

  /**
   * Returns default value for parameter <code>distance2</code>.
   * <i>This method should not be called by user</i>
   */
  @AnyLogicInternalCodegenAPI
  public double _distance2_DefaultValue_xjal() {
    final Main self = this;
    return
(double) selectFrom(parameters)
       .where(parameters.parameter.eq("class.2.dist"))
       .uniqueResult(parameters.starting_value)

;
  }

  public void set_distance2( double distance2 ) {
    if (distance2 == this.distance2) {
      return;
    }
    double _oldValue_xjal = this.distance2;
    this.distance2 = distance2;
    onChange_distance2_xjal( _oldValue_xjal );
    onChange();
  }

  /**
   * Calls "On change" action for parameter distance2.<br>
   * Note that 'oldValue' in that action will be unavailable if this method is called
by user
   * (current parameter value will be passed as 'oldValue').<br>
   * Please call <code>set_distance2()</code> method instead.
   */
  protected void onChange_distance2() {
    onChange_distance2_xjal( distance2 );
  }

  @AnyLogicInternalCodegenAPI
  protected void onChange_distance2_xjal( double oldValue ) {
  }


  public
double  clinic2;

  /**
   * Returns default value for parameter <code>clinic2</code>.
   * <i>This method should not be called by user</i>
   */
  @AnyLogicInternalCodegenAPI
  public double _clinic2_DefaultValue_xjal() {
    final Main self = this;
    return
```

```
(double) selectFrom(parameters)
       .where(parameters.parameter.eq("class.2.spaClinic"))
       .uniqueResult(parameters.starting_value)
;
  }

  public void set_clinic2( double clinic2 ) {
    if (clinic2 == this.clinic2) {
      return;
    }
    double _oldValue_xjal = this.clinic2;
    this.clinic2 = clinic2;
    onChange_clinic2_xjal( _oldValue_xjal );
    onChange();
  }

  /**
   * Calls "On change" action for parameter clinic2.<br>
   * Note that 'oldValue' in that action will be unavailable if this method is called
by user
   * (current parameter value will be passed as 'oldValue').<br>
   * Please call <code>set_clinic2()</code> method instead.
   */
  protected void onChange_clinic2() {
    onChange_clinic2_xjal( clinic2 );
  }

  @AnyLogicInternalCodegenAPI
  protected void onChange_clinic2_xjal( double oldValue ) {
  }


  public
double  maternity2;

  /**
   * Returns default value for parameter <code>maternity2</code>.
   * <i>This method should not be called by user</i>
   */
  @AnyLogicInternalCodegenAPI
  public double _maternity2_DefaultValue_xjal() {
    final Main self = this;
    return
(double) selectFrom(parameters)
       .where(parameters.parameter.eq("class.2.spaMaternity"))
       .uniqueResult(parameters.starting_value)
;
  }

  public void set_maternity2( double maternity2 ) {
    if (maternity2 == this.maternity2) {
      return;
    }
    double _oldValue_xjal = this.maternity2;
    this.maternity2 = maternity2;
    onChange_maternity2_xjal( _oldValue_xjal );
    onChange();
  }

  /**
   * Calls "On change" action for parameter maternity2.<br>
   * Note that 'oldValue' in that action will be unavailable if this method is called
by user
```

```
   * (current parameter value will be passed as 'oldValue').<br>
   * Please call <code>set_maternity2()</code> method instead.
   */
  protected void onChange_maternity2() {
    onChange_maternity2_xjal( maternity2 );
  }

  @AnyLogicInternalCodegenAPI
  protected void onChange_maternity2_xjal( double oldValue ) {
  }


  public
double  healthCenter2;

  /**
   * Returns default value for parameter <code>healthCenter2</code>.
   * <i>This method should not be called by user</i>
   */
  @AnyLogicInternalCodegenAPI
  public double _healthCenter2_DefaultValue_xjal() {
    final Main self = this;
    return
(double) selectFrom(parameters)
      .where(parameters.parameter.eq("class.2.spaHeh center"))
      .uniqueResult(parameters.starting_value)
;
  }

  public void set_healthCenter2( double healthCenter2 ) {
    if (healthCenter2 == this.healthCenter2) {
      return;
    }
    double _oldValue_xjal = this.healthCenter2;
    this.healthCenter2 = healthCenter2;
    onChange_healthCenter2_xjal( _oldValue_xjal );
    onChange();
  }

  /**
   * Calls "On change" action for parameter healthCenter2.<br>
   * Note that 'oldValue' in that action will be unavailable if this method is called
by user
   * (current parameter value will be passed as 'oldValue').<br>
   * Please call <code>set_healthCenter2()</code> method instead.
   */
  protected void onChange_healthCenter2() {
    onChange_healthCenter2_xjal( healthCenter2 );
  }

  @AnyLogicInternalCodegenAPI
  protected void onChange_healthCenter2_xjal( double oldValue ) {
  }


  public
double  otherHosp2;

  /**
   * Returns default value for parameter <code>otherHosp2</code>.
   * <i>This method should not be called by user</i>
   */
  @AnyLogicInternalCodegenAPI
```

```
  public double _otherHosp2_DefaultValue_xjal() {
    final Main self = this;
    return
(double) selectFrom(parameters)
       .where(parameters.parameter.eq("class.2.spaOther hospital"))
       .uniqueResult(parameters.starting_value)
;
  }

  public void set_otherHosp2( double otherHosp2 ) {
    if (otherHosp2 == this.otherHosp2) {
      return;
    }
    double _oldValue_xjal = this.otherHosp2;
    this.otherHosp2 = otherHosp2;
    onChange_otherHosp2_xjal( _oldValue_xjal );
    onChange();
  }

  /**
   * Calls "On change" action for parameter otherHosp2.<br>
   * Note that 'oldValue' in that action will be unavailable if this method is called
by user
   * (current parameter value will be passed as 'oldValue').<br>
   * Please call <code>set_otherHosp2()</code> method instead.
   */
  protected void onChange_otherHosp2() {
    onChange_otherHosp2_xjal( otherHosp2 );
  }

  @AnyLogicInternalCodegenAPI
  protected void onChange_otherHosp2_xjal( double oldValue ) {
  }


  public
double  communityHosp2;

  /**
   * Returns default value for parameter <code>communityHosp2</code>.
   * <i>This method should not be called by user</i>
   */
  @AnyLogicInternalCodegenAPI
  public double _communityHosp2_DefaultValue_xjal() {
    final Main self = this;
    return
(double) selectFrom(parameters)
       .where(parameters.parameter.eq("class.2.spaCommunity hospital"))
       .uniqueResult(parameters.starting_value)
;
  }

  public void set_communityHosp2( double communityHosp2 ) {
    if (communityHosp2 == this.communityHosp2) {
      return;
    }
    double _oldValue_xjal = this.communityHosp2;
    this.communityHosp2 = communityHosp2;
    onChange_communityHosp2_xjal( _oldValue_xjal );
    onChange();
  }

  /**
```

```
   * Calls "On change" action for parameter communityHosp2.<br>
   * Note that 'oldValue' in that action will be unavailable if this method is called
by user
   * (current parameter value will be passed as 'oldValue').<br>
   * Please call <code>set_communityHosp2()</code> method instead.
   */
  protected void onChange_communityHosp2() {
    onChange_communityHosp2_xjal( communityHosp2 );
  }

  @AnyLogicInternalCodegenAPI
  protected void onChange_communityHosp2_xjal( double oldValue ) {
  }


  public
double  districtHosp2;

  /**
   * Returns default value for parameter <code>districtHosp2</code>.
   * <i>This method should not be called by user</i>
   */
  @AnyLogicInternalCodegenAPI
  public double _districtHosp2_DefaultValue_xjal() {
    final Main self = this;
    return
(double) selectFrom(parameters)
       .where(parameters.parameter.eq("class.2.spaDistrict hospital"))
       .uniqueResult(parameters.starting_value)
;
  }

  public void set_districtHosp2( double districtHosp2 ) {
    if (districtHosp2 == this.districtHosp2) {
      return;
    }
    double _oldValue_xjal = this.districtHosp2;
    this.districtHosp2 = districtHosp2;
    onChange_districtHosp2_xjal( _oldValue_xjal );
    onChange();
  }

  /**
   * Calls "On change" action for parameter districtHosp2.<br>
   * Note that 'oldValue' in that action will be unavailable if this method is called
by user
   * (current parameter value will be passed as 'oldValue').<br>
   * Please call <code>set_districtHosp2()</code> method instead.
   */
  protected void onChange_districtHosp2() {
    onChange_districtHosp2_xjal( districtHosp2 );
  }

  @AnyLogicInternalCodegenAPI
  protected void onChange_districtHosp2_xjal( double oldValue ) {
  }


  public
double  bobs2;

  /**
   * Returns default value for parameter <code>bobs2</code>.
```

```
   * <i>This method should not be called by user</i>
   */
  @AnyLogicInternalCodegenAPI
  public double _bobs2_DefaultValue_xjal() {
    final Main self = this;
    return
(double) selectFrom(parameters)
       .where(parameters.parameter.eq("class.2.bobs"))
       .uniqueResult(parameters.starting_value)
;
  }

  public void set_bobs2( double bobs2 ) {
    if (bobs2 == this.bobs2) {
      return;
    }
    double _oldValue_xjal = this.bobs2;
    this.bobs2 = bobs2;
    onChange_bobs2_xjal( _oldValue_xjal );
    onChange();
  }

  /**
   * Calls "On change" action for parameter bobs2.<br>
   * Note that 'oldValue' in that action will be unavailable if this method is called
by user
   * (current parameter value will be passed as 'oldValue').<br>
   * Please call <code>set_bobs2()</code> method instead.
   */
  protected void onChange_bobs2() {
    onChange_bobs2_xjal( bobs2 );
  }

  @AnyLogicInternalCodegenAPI
  protected void onChange_bobs2_xjal( double oldValue ) {
  }


  public
double  fees2;

  /**
   * Returns default value for parameter <code>fees2</code>.
   * <i>This method should not be called by user</i>
   */
  @AnyLogicInternalCodegenAPI
  public double _fees2_DefaultValue_xjal() {
    final Main self = this;
    return
(double) selectFrom(parameters)
       .where(parameters.parameter.eq("class.2.fees"))
       .uniqueResult(parameters.starting_value)
;
  }

  public void set_fees2( double fees2 ) {
    if (fees2 == this.fees2) {
      return;
    }
    double _oldValue_xjal = this.fees2;
    this.fees2 = fees2;
    onChange_fees2_xjal( _oldValue_xjal );
    onChange();
```

```
  }

  /**
   * Calls "On change" action for parameter fees2.<br>
   * Note that 'oldValue' in that action will be unavailable if this method is called
by user
   * (current parameter value will be passed as 'oldValue').<br>
   * Please call <code>set_fees2()</code> method instead.
   */
  protected void onChange_fees2() {
    onChange_fees2_xjal( fees2 );
  }

  @AnyLogicInternalCodegenAPI
  protected void onChange_fees2_xjal( double oldValue ) {
  }


  public
double  distCenter;

  /**
   * Returns default value for parameter <code>distCenter</code>.
   * <i>This method should not be called by user</i>
   */
  @AnyLogicInternalCodegenAPI
  public double _distCenter_DefaultValue_xjal() {
    final Main self = this;
    return
(double) selectFrom(parameters)
      .where(parameters.parameter.eq("distCenter"))
      .uniqueResult(parameters.starting_value)
;
  }

  public void set_distCenter( double distCenter ) {
    if (distCenter == this.distCenter) {
      return;
    }
    double _oldValue_xjal = this.distCenter;
    this.distCenter = distCenter;
    onChange_distCenter_xjal( _oldValue_xjal );
    onChange();
  }

  /**
   * Calls "On change" action for parameter distCenter.<br>
   * Note that 'oldValue' in that action will be unavailable if this method is called
by user
   * (current parameter value will be passed as 'oldValue').<br>
   * Please call <code>set_distCenter()</code> method instead.
   */
  protected void onChange_distCenter() {
    onChange_distCenter_xjal( distCenter );
  }

  @AnyLogicInternalCodegenAPI
  protected void onChange_distCenter_xjal( double oldValue ) {
  }


  public
double  distRMS;
```

```
  /**
   * Returns default value for parameter <code>distRMS</code>.
   * <i>This method should not be called by user</i>
   */
  @AnyLogicInternalCodegenAPI
  public double _distRMS_DefaultValue_xjal() {
    final Main self = this;
    return
(double) selectFrom(parameters)
       .where(parameters.parameter.eq("distRMS"))
       .uniqueResult(parameters.starting_value)
;
  }

  public void set_distRMS( double distRMS ) {
    if (distRMS == this.distRMS) {
      return;
    }
    double _oldValue_xjal = this.distRMS;
    this.distRMS = distRMS;
    onChange_distRMS_xjal( _oldValue_xjal );
    onChange();
  }

  /**
   * Calls "On change" action for parameter distRMS.<br>
   * Note that 'oldValue' in that action will be unavailable if this method is called
by user
   * (current parameter value will be passed as 'oldValue').<br>
   * Please call <code>set_distRMS()</code> method instead.
   */
  protected void onChange_distRMS() {
    onChange_distRMS_xjal( distRMS );
  }

  @AnyLogicInternalCodegenAPI
  protected void onChange_distRMS_xjal( double oldValue ) {
  }


  public
double  centralHosp1SE;

  /**
   * Returns default value for parameter <code>centralHosp1SE</code>.
   * <i>This method should not be called by user</i>
   */
  @AnyLogicInternalCodegenAPI
  public double _centralHosp1SE_DefaultValue_xjal() {
    final Main self = this;
    return
0
;
  }

  public void set_centralHosp1SE( double centralHosp1SE ) {
    if (centralHosp1SE == this.centralHosp1SE) {
      return;
    }
    double _oldValue_xjal = this.centralHosp1SE;
    this.centralHosp1SE = centralHosp1SE;
    onChange_centralHosp1SE_xjal( _oldValue_xjal );
```

```
    onChange();
  }

  /**
   * Calls "On change" action for parameter centralHosp1SE.<br>
   * Note that 'oldValue' in that action will be unavailable if this method is called
by user
   * (current parameter value will be passed as 'oldValue').<br>
   * Please call <code>set_centralHosp1SE()</code> method instead.
   */
  protected void onChange_centralHosp1SE() {
    onChange_centralHosp1SE_xjal( centralHosp1SE );
  }

  @AnyLogicInternalCodegenAPI
  protected void onChange_centralHosp1SE_xjal( double oldValue ) {
  }


  public
double  distance1SE;

  /**
   * Returns default value for parameter <code>distance1SE</code>.
   * <i>This method should not be called by user</i>
   */
  @AnyLogicInternalCodegenAPI
  public double _distance1SE_DefaultValue_xjal() {
    final Main self = this;
    return
(double) selectFrom(parameters)
      .where(parameters.parameter.eq("class.1.dist"))
      .uniqueResult(parameters.se)
;
  }

  public void set_distance1SE( double distance1SE ) {
    if (distance1SE == this.distance1SE) {
      return;
    }
    double _oldValue_xjal = this.distance1SE;
    this.distance1SE = distance1SE;
    onChange_distance1SE_xjal( _oldValue_xjal );
    onChange();
  }

  /**
   * Calls "On change" action for parameter distance1SE.<br>
   * Note that 'oldValue' in that action will be unavailable if this method is called
by user
   * (current parameter value will be passed as 'oldValue').<br>
   * Please call <code>set_distance1SE()</code> method instead.
   */
  protected void onChange_distance1SE() {
    onChange_distance1SE_xjal( distance1SE );
  }

  @AnyLogicInternalCodegenAPI
  protected void onChange_distance1SE_xjal( double oldValue ) {
  }


  public
```

```
double  clinic1SE;

  /**
   * Returns default value for parameter <code>clinic1SE</code>.
   * <i>This method should not be called by user</i>
   */
  @AnyLogicInternalCodegenAPI
  public double _clinic1SE_DefaultValue_xjal() {
    final Main self = this;
    return
(double) selectFrom(parameters)
       .where(parameters.parameter.eq("class.1.spaClinic"))
       .uniqueResult(parameters.se)
;
  }

  public void set_clinic1SE( double clinic1SE ) {
    if (clinic1SE == this.clinic1SE) {
      return;
    }
    double _oldValue_xjal = this.clinic1SE;
    this.clinic1SE = clinic1SE;
    onChange_clinic1SE_xjal( _oldValue_xjal );
    onChange();
  }

  /**
   * Calls "On change" action for parameter clinic1SE.<br>
   * Note that 'oldValue' in that action will be unavailable if this method is called
by user
   * (current parameter value will be passed as 'oldValue').<br>
   * Please call <code>set_clinic1SE()</code> method instead.
   */
  protected void onChange_clinic1SE() {
    onChange_clinic1SE_xjal( clinic1SE );
  }

  @AnyLogicInternalCodegenAPI
  protected void onChange_clinic1SE_xjal( double oldValue ) {
  }


  public
double  maternity1SE;

  /**
   * Returns default value for parameter <code>maternity1SE</code>.
   * <i>This method should not be called by user</i>
   */
  @AnyLogicInternalCodegenAPI
  public double _maternity1SE_DefaultValue_xjal() {
    final Main self = this;
    return
(double) selectFrom(parameters)
       .where(parameters.parameter.eq("class.1.spaMaternity"))
       .uniqueResult(parameters.se)
;
  }

  public void set_maternity1SE( double maternity1SE ) {
    if (maternity1SE == this.maternity1SE) {
      return;
    }
```

```
    double _oldValue_xjal = this.maternity1SE;
    this.maternity1SE = maternity1SE;
    onChange_maternity1SE_xjal( _oldValue_xjal );
    onChange();
  }

  /**
   * Calls "On change" action for parameter maternity1SE.<br>
   * Note that 'oldValue' in that action will be unavailable if this method is called
by user
   * (current parameter value will be passed as 'oldValue').<br>
   * Please call <code>set_maternity1SE()</code> method instead.
   */
  protected void onChange_maternity1SE() {
    onChange_maternity1SE_xjal( maternity1SE );
  }

  @AnyLogicInternalCodegenAPI
  protected void onChange_maternity1SE_xjal( double oldValue ) {
  }


  public
double  healthCenter1SE;

  /**
   * Returns default value for parameter <code>healthCenter1SE</code>.
   * <i>This method should not be called by user</i>
   */
  @AnyLogicInternalCodegenAPI
  public double _healthCenter1SE_DefaultValue_xjal() {
    final Main self = this;
    return
(double) selectFrom(parameters)
       .where(parameters.parameter.eq("class.1.spaHeh center"))
       .uniqueResult(parameters.se)
;
  }

  public void set_healthCenter1SE( double healthCenter1SE ) {
    if (healthCenter1SE == this.healthCenter1SE) {
      return;
    }
    double _oldValue_xjal = this.healthCenter1SE;
    this.healthCenter1SE = healthCenter1SE;
    onChange_healthCenter1SE_xjal( _oldValue_xjal );
    onChange();
  }

  /**
   * Calls "On change" action for parameter healthCenter1SE.<br>
   * Note that 'oldValue' in that action will be unavailable if this method is called
by user
   * (current parameter value will be passed as 'oldValue').<br>
   * Please call <code>set_healthCenter1SE()</code> method instead.
   */
  protected void onChange_healthCenter1SE() {
    onChange_healthCenter1SE_xjal( healthCenter1SE );
  }

  @AnyLogicInternalCodegenAPI
  protected void onChange_healthCenter1SE_xjal( double oldValue ) {
  }
```

```
  public
double  otherHosp1SE;

  /**
   * Returns default value for parameter <code>otherHosp1SE</code>.
   * <i>This method should not be called by user</i>
   */
  @AnyLogicInternalCodegenAPI
  public double _otherHosp1SE_DefaultValue_xjal() {
    final Main self = this;
    return
(double) selectFrom(parameters)
       .where(parameters.parameter.eq("class.1.spaOther hospital"))
       .uniqueResult(parameters.se)
;
  }

  public void set_otherHosp1SE( double otherHosp1SE ) {
    if (otherHosp1SE == this.otherHosp1SE) {
      return;
    }
    double _oldValue_xjal = this.otherHosp1SE;
    this.otherHosp1SE = otherHosp1SE;
    onChange_otherHosp1SE_xjal( _oldValue_xjal );
    onChange();
  }

  /**
   * Calls "On change" action for parameter otherHosp1SE.<br>
   * Note that 'oldValue' in that action will be unavailable if this method is called
by user
   * (current parameter value will be passed as 'oldValue').<br>
   * Please call <code>set_otherHosp1SE()</code> method instead.
   */
  protected void onChange_otherHosp1SE() {
    onChange_otherHosp1SE_xjal( otherHosp1SE );
  }

  @AnyLogicInternalCodegenAPI
  protected void onChange_otherHosp1SE_xjal( double oldValue ) {
  }


  public
double  communityHosp1SE;

  /**
   * Returns default value for parameter <code>communityHosp1SE</code>.
   * <i>This method should not be called by user</i>
   */
  @AnyLogicInternalCodegenAPI
  public double _communityHosp1SE_DefaultValue_xjal() {
    final Main self = this;
    return
(double) selectFrom(parameters)
       .where(parameters.parameter.eq("class.1.spaCommunity hospital"))
       .uniqueResult(parameters.se)
;
  }

  public void set_communityHosp1SE( double communityHosp1SE ) {
```

```
    if (communityHosp1SE == this.communityHosp1SE) {
      return;
    }
    double _oldValue_xjal = this.communityHosp1SE;
    this.communityHosp1SE = communityHosp1SE;
    onChange_communityHosp1SE_xjal( _oldValue_xjal );
    onChange();
  }

  /**
   * Calls "On change" action for parameter communityHosp1SE.<br>
   * Note that 'oldValue' in that action will be unavailable if this method is called
by user
   * (current parameter value will be passed as 'oldValue').<br>
   * Please call <code>set_communityHosp1SE()</code> method instead.
   */
  protected void onChange_communityHosp1SE() {
    onChange_communityHosp1SE_xjal( communityHosp1SE );
  }

  @AnyLogicInternalCodegenAPI
  protected void onChange_communityHosp1SE_xjal( double oldValue ) {
  }


  public
double  districtHosp1SE;

  /**
   * Returns default value for parameter <code>districtHosp1SE</code>.
   * <i>This method should not be called by user</i>
   */
  @AnyLogicInternalCodegenAPI
  public double _districtHosp1SE_DefaultValue_xjal() {
    final Main self = this;
    return
(double) selectFrom(parameters)
      .where(parameters.parameter.eq("class.1.spaDistrict hospital"))
      .uniqueResult(parameters.se)
;
  }

  public void set_districtHosp1SE( double districtHosp1SE ) {
    if (districtHosp1SE == this.districtHosp1SE) {
      return;
    }
    double _oldValue_xjal = this.districtHosp1SE;
    this.districtHosp1SE = districtHosp1SE;
    onChange_districtHosp1SE_xjal( _oldValue_xjal );
    onChange();
  }

  /**
   * Calls "On change" action for parameter districtHosp1SE.<br>
   * Note that 'oldValue' in that action will be unavailable if this method is called
by user
   * (current parameter value will be passed as 'oldValue').<br>
   * Please call <code>set_districtHosp1SE()</code> method instead.
   */
  protected void onChange_districtHosp1SE() {
    onChange_districtHosp1SE_xjal( districtHosp1SE );
  }
```

```
  @AnyLogicInternalCodegenAPI
  protected void onChange_districtHosp1SE_xjal( double oldValue ) {
  }


  public
double  bobs1SE;

  /**
   * Returns default value for parameter <code>bobs1SE</code>.
   * <i>This method should not be called by user</i>
   */
  @AnyLogicInternalCodegenAPI
  public double _bobs1SE_DefaultValue_xjal() {
    final Main self = this;
    return
(double) selectFrom(parameters)
       .where(parameters.parameter.eq("class.1.bobs"))
       .uniqueResult(parameters.se)
;
  }

  public void set_bobs1SE( double bobs1SE ) {
    if (bobs1SE == this.bobs1SE) {
      return;
    }
    double _oldValue_xjal = this.bobs1SE;
    this.bobs1SE = bobs1SE;
    onChange_bobs1SE_xjal( _oldValue_xjal );
    onChange();
  }

  /**
   * Calls "On change" action for parameter bobs1SE.<br>
   * Note that 'oldValue' in that action will be unavailable if this method is called
by user
   * (current parameter value will be passed as 'oldValue').<br>
   * Please call <code>set_bobs1SE()</code> method instead.
   */
  protected void onChange_bobs1SE() {
    onChange_bobs1SE_xjal( bobs1SE );
  }

  @AnyLogicInternalCodegenAPI
  protected void onChange_bobs1SE_xjal( double oldValue ) {
  }


  public
double  fees1SE;

  /**
   * Returns default value for parameter <code>fees1SE</code>.
   * <i>This method should not be called by user</i>
   */
  @AnyLogicInternalCodegenAPI
  public double _fees1SE_DefaultValue_xjal() {
    final Main self = this;
    return
(double) selectFrom(parameters)
       .where(parameters.parameter.eq("class.1.fees"))
       .uniqueResult(parameters.se)
;
```

```
  }

  public void set_fees1SE( double fees1SE ) {
    if (fees1SE == this.fees1SE) {
      return;
    }
    double _oldValue_xjal = this.fees1SE;
    this.fees1SE = fees1SE;
    onChange_fees1SE_xjal( _oldValue_xjal );
    onChange();
  }

  /**
   * Calls "On change" action for parameter fees1SE.<br>
   * Note that 'oldValue' in that action will be unavailable if this method is called
by user
   * (current parameter value will be passed as 'oldValue').<br>
   * Please call <code>set_fees1SE()</code> method instead.
   */
  protected void onChange_fees1SE() {
    onChange_fees1SE_xjal( fees1SE );
  }

  @AnyLogicInternalCodegenAPI
  protected void onChange_fees1SE_xjal( double oldValue ) {
  }


  public
double  interceptSE;

  /**
   * Returns default value for parameter <code>interceptSE</code>.
   * <i>This method should not be called by user</i>
   */
  @AnyLogicInternalCodegenAPI
  public double _interceptSE_DefaultValue_xjal() {
    final Main self = this;
    return
(double) selectFrom(parameters)
       .where(parameters.parameter.eq("Intercept"))
       .uniqueResult(parameters.se)
;
  }

  public void set_interceptSE( double interceptSE ) {
    if (interceptSE == this.interceptSE) {
      return;
    }
    double _oldValue_xjal = this.interceptSE;
    this.interceptSE = interceptSE;
    onChange_interceptSE_xjal( _oldValue_xjal );
    onChange();
  }

  /**
   * Calls "On change" action for parameter interceptSE.<br>
   * Note that 'oldValue' in that action will be unavailable if this method is called
by user
   * (current parameter value will be passed as 'oldValue').<br>
   * Please call <code>set_interceptSE()</code> method instead.
   */
  protected void onChange_interceptSE() {
```

```
    onChange_interceptSE_xjal( interceptSE );
  }

  @AnyLogicInternalCodegenAPI
  protected void onChange_interceptSE_xjal( double oldValue ) {
  }


  public
double  centralHosp2SE;

  /**
   * Returns default value for parameter <code>centralHosp2SE</code>.
   * <i>This method should not be called by user</i>
   */
  @AnyLogicInternalCodegenAPI
  public double _centralHosp2SE_DefaultValue_xjal() {
    final Main self = this;
    return
0
;
  }

  public void set_centralHosp2SE( double centralHosp2SE ) {
    if (centralHosp2SE == this.centralHosp2SE) {
      return;
    }
    double _oldValue_xjal = this.centralHosp2SE;
    this.centralHosp2SE = centralHosp2SE;
    onChange_centralHosp2SE_xjal( _oldValue_xjal );
    onChange();
  }

  /**
   * Calls "On change" action for parameter centralHosp2SE.<br>
   * Note that 'oldValue' in that action will be unavailable if this method is called
by user
   * (current parameter value will be passed as 'oldValue').<br>
   * Please call <code>set_centralHosp2SE()</code> method instead.
   */
  protected void onChange_centralHosp2SE() {
    onChange_centralHosp2SE_xjal( centralHosp2SE );
  }

  @AnyLogicInternalCodegenAPI
  protected void onChange_centralHosp2SE_xjal( double oldValue ) {
  }


  public
double  distance2SE;

  /**
   * Returns default value for parameter <code>distance2SE</code>.
   * <i>This method should not be called by user</i>
   */
  @AnyLogicInternalCodegenAPI
  public double _distance2SE_DefaultValue_xjal() {
    final Main self = this;
    return
(double) selectFrom(parameters)
      .where(parameters.parameter.eq("class.2.dist"))
      .uniqueResult(parameters.se)
```

```
;
  }

  public void set_distance2SE( double distance2SE ) {
    if (distance2SE == this.distance2SE) {
      return;
    }
    double _oldValue_xjal = this.distance2SE;
    this.distance2SE = distance2SE;
    onChange_distance2SE_xjal( _oldValue_xjal );
    onChange();
  }

  /**
   * Calls "On change" action for parameter distance2SE.<br>
   * Note that 'oldValue' in that action will be unavailable if this method is called
by user
   * (current parameter value will be passed as 'oldValue').<br>
   * Please call <code>set_distance2SE()</code> method instead.
   */
  protected void onChange_distance2SE() {
    onChange_distance2SE_xjal( distance2SE );
  }

  @AnyLogicInternalCodegenAPI
  protected void onChange_distance2SE_xjal( double oldValue ) {
  }


  public
double  clinic2SE;

  /**
   * Returns default value for parameter <code>clinic2SE</code>.
   * <i>This method should not be called by user</i>
   */
  @AnyLogicInternalCodegenAPI
  public double _clinic2SE_DefaultValue_xjal() {
    final Main self = this;
    return
(double) selectFrom(parameters)
      .where(parameters.parameter.eq("class.2.spaClinic"))
      .uniqueResult(parameters.se)
;
  }

  public void set_clinic2SE( double clinic2SE ) {
    if (clinic2SE == this.clinic2SE) {
      return;
    }
    double _oldValue_xjal = this.clinic2SE;
    this.clinic2SE = clinic2SE;
    onChange_clinic2SE_xjal( _oldValue_xjal );
    onChange();
  }

  /**
   * Calls "On change" action for parameter clinic2SE.<br>
   * Note that 'oldValue' in that action will be unavailable if this method is called
by user
   * (current parameter value will be passed as 'oldValue').<br>
   * Please call <code>set_clinic2SE()</code> method instead.
```

```
   */
  protected void onChange_clinic2SE() {
    onChange_clinic2SE_xjal( clinic2SE );
  }

  @AnyLogicInternalCodegenAPI
  protected void onChange_clinic2SE_xjal( double oldValue ) {
  }


  public
double  maternity2SE;

  /**
   * Returns default value for parameter <code>maternity2SE</code>.
   * <i>This method should not be called by user</i>
   */
  @AnyLogicInternalCodegenAPI
  public double _maternity2SE_DefaultValue_xjal() {
    final Main self = this;
    return
(double) selectFrom(parameters)
       .where(parameters.parameter.eq("class.2.spaMaternity"))
       .uniqueResult(parameters.se)
;
  }

  public void set_maternity2SE( double maternity2SE ) {
    if (maternity2SE == this.maternity2SE) {
      return;
    }
    double _oldValue_xjal = this.maternity2SE;
    this.maternity2SE = maternity2SE;
    onChange_maternity2SE_xjal( _oldValue_xjal );
    onChange();
  }

  /**
   * Calls "On change" action for parameter maternity2SE.<br>
   * Note that 'oldValue' in that action will be unavailable if this method is called
by user
   * (current parameter value will be passed as 'oldValue').<br>
   * Please call <code>set_maternity2SE()</code> method instead.
   */
  protected void onChange_maternity2SE() {
    onChange_maternity2SE_xjal( maternity2SE );
  }

  @AnyLogicInternalCodegenAPI
  protected void onChange_maternity2SE_xjal( double oldValue ) {
  }


  public
double  healthCenter2SE;

  /**
   * Returns default value for parameter <code>healthCenter2SE</code>.
   * <i>This method should not be called by user</i>
   */
  @AnyLogicInternalCodegenAPI
  public double _healthCenter2SE_DefaultValue_xjal() {
    final Main self = this;
```

```
    return
(double) selectFrom(parameters)
       .where(parameters.parameter.eq("class.2.spaHeh center"))
       .uniqueResult(parameters.se)
;
  }

  public void set_healthCenter2SE( double healthCenter2SE ) {
    if (healthCenter2SE == this.healthCenter2SE) {
      return;
    }
    double _oldValue_xjal = this.healthCenter2SE;
    this.healthCenter2SE = healthCenter2SE;
    onChange_healthCenter2SE_xjal( _oldValue_xjal );
    onChange();
  }

  /**
   * Calls "On change" action for parameter healthCenter2SE.<br>
   * Note that 'oldValue' in that action will be unavailable if this method is called
by user
   * (current parameter value will be passed as 'oldValue').<br>
   * Please call <code>set_healthCenter2SE()</code> method instead.
   */
  protected void onChange_healthCenter2SE() {
    onChange_healthCenter2SE_xjal( healthCenter2SE );
  }

  @AnyLogicInternalCodegenAPI
  protected void onChange_healthCenter2SE_xjal( double oldValue ) {
  }


  public
double  otherHosp2SE;

  /**
   * Returns default value for parameter <code>otherHosp2SE</code>.
   * <i>This method should not be called by user</i>
   */
  @AnyLogicInternalCodegenAPI
  public double _otherHosp2SE_DefaultValue_xjal() {
    final Main self = this;
    return
(double) selectFrom(parameters)
       .where(parameters.parameter.eq("class.2.spaOther hospital"))
       .uniqueResult(parameters.se)
;
  }

  public void set_otherHosp2SE( double otherHosp2SE ) {
    if (otherHosp2SE == this.otherHosp2SE) {
      return;
    }
    double _oldValue_xjal = this.otherHosp2SE;
    this.otherHosp2SE = otherHosp2SE;
    onChange_otherHosp2SE_xjal( _oldValue_xjal );
    onChange();
  }

  /**
   * Calls "On change" action for parameter otherHosp2SE.<br>
```

```
   * Note that 'oldValue' in that action will be unavailable if this method is called
by user
   * (current parameter value will be passed as 'oldValue').<br>
   * Please call <code>set_otherHosp2SE()</code> method instead.
   */
  protected void onChange_otherHosp2SE() {
    onChange_otherHosp2SE_xjal( otherHosp2SE );
  }

  @AnyLogicInternalCodegenAPI
  protected void onChange_otherHosp2SE_xjal( double oldValue ) {
  }


  public
double  communityHosp2SE;

  /**
   * Returns default value for parameter <code>communityHosp2SE</code>.
   * <i>This method should not be called by user</i>
   */
  @AnyLogicInternalCodegenAPI
  public double _communityHosp2SE_DefaultValue_xjal() {
    final Main self = this;
    return
(double) selectFrom(parameters)
       .where(parameters.parameter.eq("class.2.spaCommunity hospital"))
       .uniqueResult(parameters.se)
;
  }

  public void set_communityHosp2SE( double communityHosp2SE ) {
    if (communityHosp2SE == this.communityHosp2SE) {
      return;
    }
    double _oldValue_xjal = this.communityHosp2SE;
    this.communityHosp2SE = communityHosp2SE;
    onChange_communityHosp2SE_xjal( _oldValue_xjal );
    onChange();
  }

  /**
   * Calls "On change" action for parameter communityHosp2SE.<br>
   * Note that 'oldValue' in that action will be unavailable if this method is called
by user
   * (current parameter value will be passed as 'oldValue').<br>
   * Please call <code>set_communityHosp2SE()</code> method instead.
   */
  protected void onChange_communityHosp2SE() {
    onChange_communityHosp2SE_xjal( communityHosp2SE );
  }

  @AnyLogicInternalCodegenAPI
  protected void onChange_communityHosp2SE_xjal( double oldValue ) {
  }


  public
double  districtHosp2SE;

  /**
   * Returns default value for parameter <code>districtHosp2SE</code>.
   * <i>This method should not be called by user</i>
```

```
   */
  @AnyLogicInternalCodegenAPI
  public double _districtHosp2SE_DefaultValue_xjal() {
    final Main self = this;
    return 
(double) selectFrom(parameters)
       .where(parameters.parameter.eq("class.2.spaDistrict hospital"))
       .uniqueResult(parameters.se)
;
  }

  public void set_districtHosp2SE( double districtHosp2SE ) {
    if (districtHosp2SE == this.districtHosp2SE) {
      return;
    }
    double _oldValue_xjal = this.districtHosp2SE;
    this.districtHosp2SE = districtHosp2SE;
    onChange_districtHosp2SE_xjal( _oldValue_xjal );
    onChange();
  }

  /**
   * Calls "On change" action for parameter districtHosp2SE.<br>
   * Note that 'oldValue' in that action will be unavailable if this method is called 
by user
   * (current parameter value will be passed as 'oldValue').<br>
   * Please call <code>set_districtHosp2SE()</code> method instead.
   */
  protected void onChange_districtHosp2SE() {
    onChange_districtHosp2SE_xjal( districtHosp2SE );
  }

  @AnyLogicInternalCodegenAPI
  protected void onChange_districtHosp2SE_xjal( double oldValue ) {  
  }


  public 
double  bobs2SE;

  /**
   * Returns default value for parameter <code>bobs2SE</code>.
   * <i>This method should not be called by user</i>
   */
  @AnyLogicInternalCodegenAPI
  public double _bobs2SE_DefaultValue_xjal() {
    final Main self = this;
    return 
(double) selectFrom(parameters)
       .where(parameters.parameter.eq("class.2.bobs"))
       .uniqueResult(parameters.se)
;
  }

  public void set_bobs2SE( double bobs2SE ) {
    if (bobs2SE == this.bobs2SE) {
      return;
    }
    double _oldValue_xjal = this.bobs2SE;
    this.bobs2SE = bobs2SE;
    onChange_bobs2SE_xjal( _oldValue_xjal );
    onChange();
  }
```

```
  /**
   * Calls "On change" action for parameter bobs2SE.<br>
   * Note that 'oldValue' in that action will be unavailable if this method is called
by user
   * (current parameter value will be passed as 'oldValue').<br>
   * Please call <code>set_bobs2SE()</code> method instead.
   */
  protected void onChange_bobs2SE() {
    onChange_bobs2SE_xjal( bobs2SE );
  }

  @AnyLogicInternalCodegenAPI
  protected void onChange_bobs2SE_xjal( double oldValue ) {
  }


  public
double  fees2SE;

  /**
   * Returns default value for parameter <code>fees2SE</code>.
   * <i>This method should not be called by user</i>
   */
  @AnyLogicInternalCodegenAPI
  public double _fees2SE_DefaultValue_xjal() {
    final Main self = this;
    return
(double) selectFrom(parameters)
       .where(parameters.parameter.eq("class.2.fees"))
       .uniqueResult(parameters.se)
;
  }

  public void set_fees2SE( double fees2SE ) {
    if (fees2SE == this.fees2SE) {
      return;
    }
    double _oldValue_xjal = this.fees2SE;
    this.fees2SE = fees2SE;
    onChange_fees2SE_xjal( _oldValue_xjal );
    onChange();
  }

  /**
   * Calls "On change" action for parameter fees2SE.<br>
   * Note that 'oldValue' in that action will be unavailable if this method is called
by user
   * (current parameter value will be passed as 'oldValue').<br>
   * Please call <code>set_fees2SE()</code> method instead.
   */
  protected void onChange_fees2SE() {
    onChange_fees2SE_xjal( fees2SE );
  }

  @AnyLogicInternalCodegenAPI
  protected void onChange_fees2SE_xjal( double oldValue ) {
  }


  public
double  centralCS;
```

```
  /**
   * Returns default value for parameter <code>centralCS</code>.
   * <i>This method should not be called by user</i>
   */
  @AnyLogicInternalCodegenAPI
  public double _centralCS_DefaultValue_xjal() {
    final Main self = this;
    return
(double) selectFrom(mortality)
      .where(mortality.type.eq("CentralCS"))
      .uniqueResult(mortality.mort)
;
  }

  public void set_centralCS( double centralCS ) {
    if (centralCS == this.centralCS) {
      return;
    }
    double _oldValue_xjal = this.centralCS;
    this.centralCS = centralCS;
    onChange_centralCS_xjal( _oldValue_xjal );
    onChange();
  }

  /**
   * Calls "On change" action for parameter centralCS.<br>
   * Note that 'oldValue' in that action will be unavailable if this method is called
by user
   * (current parameter value will be passed as 'oldValue').<br>
   * Please call <code>set_centralCS()</code> method instead.
   */
  protected void onChange_centralCS() {
    onChange_centralCS_xjal( centralCS );
  }

  @AnyLogicInternalCodegenAPI
  protected void onChange_centralCS_xjal( double oldValue ) {
  }


  public
double  clinicCS;

  /**
   * Returns default value for parameter <code>clinicCS</code>.
   * <i>This method should not be called by user</i>
   */
  @AnyLogicInternalCodegenAPI
  public double _clinicCS_DefaultValue_xjal() {
    final Main self = this;
    return
(double) selectFrom(mortality)
      .where(mortality.type.eq("ClinicCS"))
      .uniqueResult(mortality.mort)
;
  }

  public void set_clinicCS( double clinicCS ) {
    if (clinicCS == this.clinicCS) {
      return;
    }
    double _oldValue_xjal = this.clinicCS;
    this.clinicCS = clinicCS;
```

```
    onChange_clinicCS_xjal( _oldValue_xjal );
    onChange();
  }

  /**
   * Calls "On change" action for parameter clinicCS.<br>
   * Note that 'oldValue' in that action will be unavailable if this method is called
by user
   * (current parameter value will be passed as 'oldValue').<br>
   * Please call <code>set_clinicCS()</code> method instead.
   */
  protected void onChange_clinicCS() {
    onChange_clinicCS_xjal( clinicCS );
  }

  @AnyLogicInternalCodegenAPI
  protected void onChange_clinicCS_xjal( double oldValue ) {
  }


  public
double  maternityCS;

  /**
   * Returns default value for parameter <code>maternityCS</code>.
   * <i>This method should not be called by user</i>
   */
  @AnyLogicInternalCodegenAPI
  public double _maternityCS_DefaultValue_xjal() {
    final Main self = this;
    return
(double) selectFrom(mortality)
      .where(mortality.type.eq("MaternityCS"))
      .uniqueResult(mortality.mort)
;
  }

  public void set_maternityCS( double maternityCS ) {
    if (maternityCS == this.maternityCS) {
      return;
    }
    double _oldValue_xjal = this.maternityCS;
    this.maternityCS = maternityCS;
    onChange_maternityCS_xjal( _oldValue_xjal );
    onChange();
  }

  /**
   * Calls "On change" action for parameter maternityCS.<br>
   * Note that 'oldValue' in that action will be unavailable if this method is called
by user
   * (current parameter value will be passed as 'oldValue').<br>
   * Please call <code>set_maternityCS()</code> method instead.
   */
  protected void onChange_maternityCS() {
    onChange_maternityCS_xjal( maternityCS );
  }

  @AnyLogicInternalCodegenAPI
  protected void onChange_maternityCS_xjal( double oldValue ) {
  }
```

```
  public
double  healthCenterCS;

  /**
   * Returns default value for parameter <code>healthCenterCS</code>.
   * <i>This method should not be called by user</i>
   */
  @AnyLogicInternalCodegenAPI
  public double _healthCenterCS_DefaultValue_xjal() {
    final Main self = this;
    return
(double) selectFrom(mortality)
       .where(mortality.type.eq("HealthCenterCS"))
       .uniqueResult(mortality.mort)
;
  }

  public void set_healthCenterCS( double healthCenterCS ) {
    if (healthCenterCS == this.healthCenterCS) {
      return;
    }
    double _oldValue_xjal = this.healthCenterCS;
    this.healthCenterCS = healthCenterCS;
    onChange_healthCenterCS_xjal( _oldValue_xjal );
    onChange();
  }

  /**
   * Calls "On change" action for parameter healthCenterCS.<br>
   * Note that 'oldValue' in that action will be unavailable if this method is called
by user
   * (current parameter value will be passed as 'oldValue').<br>
   * Please call <code>set_healthCenterCS()</code> method instead.
   */
  protected void onChange_healthCenterCS() {
    onChange_healthCenterCS_xjal( healthCenterCS );
  }

  @AnyLogicInternalCodegenAPI
  protected void onChange_healthCenterCS_xjal( double oldValue ) {
  }


  public
double  otherCS;

  /**
   * Returns default value for parameter <code>otherCS</code>.
   * <i>This method should not be called by user</i>
   */
  @AnyLogicInternalCodegenAPI
  public double _otherCS_DefaultValue_xjal() {
    final Main self = this;
    return
(double) selectFrom(mortality)
       .where(mortality.type.eq("OtherCS"))
       .uniqueResult(mortality.mort)
;
  }

  public void set_otherCS( double otherCS ) {
    if (otherCS == this.otherCS) {
      return;
```

```
    }
    double _oldValue_xjal = this.otherCS;
    this.otherCS = otherCS;
    onChange_otherCS_xjal( _oldValue_xjal );
    onChange();
  }

  /**
   * Calls "On change" action for parameter otherCS.<br>
   * Note that 'oldValue' in that action will be unavailable if this method is called
by user
   * (current parameter value will be passed as 'oldValue').<br>
   * Please call <code>set_otherCS()</code> method instead.
   */
  protected void onChange_otherCS() {
    onChange_otherCS_xjal( otherCS );
  }

  @AnyLogicInternalCodegenAPI
  protected void onChange_otherCS_xjal( double oldValue ) {
  }


  public
double  communityCS;

  /**
   * Returns default value for parameter <code>communityCS</code>.
   * <i>This method should not be called by user</i>
   */
  @AnyLogicInternalCodegenAPI
  public double _communityCS_DefaultValue_xjal() {
    final Main self = this;
    return
(double) selectFrom(mortality)
      .where(mortality.type.eq("CommunityCS"))
      .uniqueResult(mortality.mort)
;
  }

  public void set_communityCS( double communityCS ) {
    if (communityCS == this.communityCS) {
      return;
    }
    double _oldValue_xjal = this.communityCS;
    this.communityCS = communityCS;
    onChange_communityCS_xjal( _oldValue_xjal );
    onChange();
  }

  /**
   * Calls "On change" action for parameter communityCS.<br>
   * Note that 'oldValue' in that action will be unavailable if this method is called
by user
   * (current parameter value will be passed as 'oldValue').<br>
   * Please call <code>set_communityCS()</code> method instead.
   */
  protected void onChange_communityCS() {
    onChange_communityCS_xjal( communityCS );
  }

  @AnyLogicInternalCodegenAPI
  protected void onChange_communityCS_xjal( double oldValue ) {
```

```
  }


  public
double  districtCS;

  /**
   * Returns default value for parameter <code>districtCS</code>.
   * <i>This method should not be called by user</i>
   */
  @AnyLogicInternalCodegenAPI
  public double _districtCS_DefaultValue_xjal() {
    final Main self = this;
    return
(double) selectFrom(mortality)
       .where(mortality.type.eq("DistrictCS"))
       .uniqueResult(mortality.mort)
;
  }

  public void set_districtCS( double districtCS ) {
    if (districtCS == this.districtCS) {
      return;
    }
    double _oldValue_xjal = this.districtCS;
    this.districtCS = districtCS;
    onChange_districtCS_xjal( _oldValue_xjal );
    onChange();
  }

  /**
   * Calls "On change" action for parameter districtCS.<br>
   * Note that 'oldValue' in that action will be unavailable if this method is called
by user
   * (current parameter value will be passed as 'oldValue').<br>
   * Please call <code>set_districtCS()</code> method instead.
   */
  protected void onChange_districtCS() {
    onChange_districtCS_xjal( districtCS );
  }

  @AnyLogicInternalCodegenAPI
  protected void onChange_districtCS_xjal( double oldValue ) {
  }


  public
double  centralCSSE;

  /**
   * Returns default value for parameter <code>centralCSSE</code>.
   * <i>This method should not be called by user</i>
   */
  @AnyLogicInternalCodegenAPI
  public double _centralCSSE_DefaultValue_xjal() {
    final Main self = this;
    return
(double) selectFrom(mortality)
       .where(mortality.type.eq("CentralCS"))
       .uniqueResult(mortality.sd)
;
  }
```

```
  public void set_centralCSSE( double centralCSSE ) {
    if (centralCSSE == this.centralCSSE) {
      return;
    }
    double _oldValue_xjal = this.centralCSSE;
    this.centralCSSE = centralCSSE;
    onChange_centralCSSE_xjal( _oldValue_xjal );
    onChange();
  }

  /**
   * Calls "On change" action for parameter centralCSSE.<br>
   * Note that 'oldValue' in that action will be unavailable if this method is called
by user
   * (current parameter value will be passed as 'oldValue').<br>
   * Please call <code>set_centralCSSE()</code> method instead.
   */
  protected void onChange_centralCSSE() {
    onChange_centralCSSE_xjal( centralCSSE );
  }

  @AnyLogicInternalCodegenAPI
  protected void onChange_centralCSSE_xjal( double oldValue ) {
  }


  public
double  clinicCSSE;

  /**
   * Returns default value for parameter <code>clinicCSSE</code>.
   * <i>This method should not be called by user</i>
   */
  @AnyLogicInternalCodegenAPI
  public double _clinicCSSE_DefaultValue_xjal() {
    final Main self = this;
    return
(double) selectFrom(mortality)
       .where(mortality.type.eq("ClinicCS"))
       .uniqueResult(mortality.sd)
;
  }

  public void set_clinicCSSE( double clinicCSSE ) {
    if (clinicCSSE == this.clinicCSSE) {
      return;
    }
    double _oldValue_xjal = this.clinicCSSE;
    this.clinicCSSE = clinicCSSE;
    onChange_clinicCSSE_xjal( _oldValue_xjal );
    onChange();
  }

  /**
   * Calls "On change" action for parameter clinicCSSE.<br>
   * Note that 'oldValue' in that action will be unavailable if this method is called
by user
   * (current parameter value will be passed as 'oldValue').<br>
   * Please call <code>set_clinicCSSE()</code> method instead.
   */
  protected void onChange_clinicCSSE() {
    onChange_clinicCSSE_xjal( clinicCSSE );
  }
```

```
  @AnyLogicInternalCodegenAPI
  protected void onChange_clinicCSSE_xjal( double oldValue ) {
  }


  public
double  maternityCSSE;

  /**
   * Returns default value for parameter <code>maternityCSSE</code>.
   * <i>This method should not be called by user</i>
   */
  @AnyLogicInternalCodegenAPI
  public double _maternityCSSE_DefaultValue_xjal() {
    final Main self = this;
    return
(double) selectFrom(mortality)
      .where(mortality.type.eq("MaternityCS"))
      .uniqueResult(mortality.sd)
;
  }

  public void set_maternityCSSE( double maternityCSSE ) {
    if (maternityCSSE == this.maternityCSSE) {
      return;
    }
    double _oldValue_xjal = this.maternityCSSE;
    this.maternityCSSE = maternityCSSE;
    onChange_maternityCSSE_xjal( _oldValue_xjal );
    onChange();
  }

  /**
   * Calls "On change" action for parameter maternityCSSE.<br>
   * Note that 'oldValue' in that action will be unavailable if this method is called
by user
   * (current parameter value will be passed as 'oldValue').<br>
   * Please call <code>set_maternityCSSE()</code> method instead.
   */
  protected void onChange_maternityCSSE() {
    onChange_maternityCSSE_xjal( maternityCSSE );
  }

  @AnyLogicInternalCodegenAPI
  protected void onChange_maternityCSSE_xjal( double oldValue ) {
  }


  public
double  healthCenterCSSE;

  /**
   * Returns default value for parameter <code>healthCenterCSSE</code>.
   * <i>This method should not be called by user</i>
   */
  @AnyLogicInternalCodegenAPI
  public double _healthCenterCSSE_DefaultValue_xjal() {
    final Main self = this;
    return
(double) selectFrom(mortality)
      .where(mortality.type.eq("HealthCenterCS"))
      .uniqueResult(mortality.sd)
```

```
;
  }

  public void set_healthCenterCSSE( double healthCenterCSSE ) {
    if (healthCenterCSSE == this.healthCenterCSSE) {
      return;
    }
    double _oldValue_xjal = this.healthCenterCSSE;
    this.healthCenterCSSE = healthCenterCSSE;
    onChange_healthCenterCSSE_xjal( _oldValue_xjal );
    onChange();
  }

  /**
   * Calls "On change" action for parameter healthCenterCSSE.<br>
   * Note that 'oldValue' in that action will be unavailable if this method is called
by user
   * (current parameter value will be passed as 'oldValue').<br>
   * Please call <code>set_healthCenterCSSE()</code> method instead.
   */
  protected void onChange_healthCenterCSSE() {
    onChange_healthCenterCSSE_xjal( healthCenterCSSE );
  }

  @AnyLogicInternalCodegenAPI
  protected void onChange_healthCenterCSSE_xjal( double oldValue ) {
  }


  public
double  otherCSSE;

  /**
   * Returns default value for parameter <code>otherCSSE</code>.
   * <i>This method should not be called by user</i>
   */
  @AnyLogicInternalCodegenAPI
  public double _otherCSSE_DefaultValue_xjal() {
    final Main self = this;
    return
(double) selectFrom(mortality)
      .where(mortality.type.eq("OtherCS"))
      .uniqueResult(mortality.sd)
;
  }

  public void set_otherCSSE( double otherCSSE ) {
    if (otherCSSE == this.otherCSSE) {
      return;
    }
    double _oldValue_xjal = this.otherCSSE;
    this.otherCSSE = otherCSSE;
    onChange_otherCSSE_xjal( _oldValue_xjal );
    onChange();
  }

  /**
   * Calls "On change" action for parameter otherCSSE.<br>
   * Note that 'oldValue' in that action will be unavailable if this method is called
by user
   * (current parameter value will be passed as 'oldValue').<br>
   * Please call <code>set_otherCSSE()</code> method instead.
   */
```

```
  protected void onChange_otherCSSE() {
    onChange_otherCSSE_xjal( otherCSSE );
  }

  @AnyLogicInternalCodegenAPI
  protected void onChange_otherCSSE_xjal( double oldValue ) {
  }


  public
double  communityCSSE;

  /**
   * Returns default value for parameter <code>communityCSSE</code>.
   * <i>This method should not be called by user</i>
   */
  @AnyLogicInternalCodegenAPI
  public double _communityCSSE_DefaultValue_xjal() {
    final Main self = this;
    return
(double) selectFrom(mortality)
      .where(mortality.type.eq("CommunityCS"))
      .uniqueResult(mortality.sd)
;
  }

  public void set_communityCSSE( double communityCSSE ) {
    if (communityCSSE == this.communityCSSE) {
      return;
    }
    double _oldValue_xjal = this.communityCSSE;
    this.communityCSSE = communityCSSE;
    onChange_communityCSSE_xjal( _oldValue_xjal );
    onChange();
  }

  /**
   * Calls "On change" action for parameter communityCSSE.<br>
   * Note that 'oldValue' in that action will be unavailable if this method is called
by user
   * (current parameter value will be passed as 'oldValue').<br>
   * Please call <code>set_communityCSSE()</code> method instead.
   */
  protected void onChange_communityCSSE() {
    onChange_communityCSSE_xjal( communityCSSE );
  }

  @AnyLogicInternalCodegenAPI
  protected void onChange_communityCSSE_xjal( double oldValue ) {
  }


  public
double  districtCSSE;

  /**
   * Returns default value for parameter <code>districtCSSE</code>.
   * <i>This method should not be called by user</i>
   */
  @AnyLogicInternalCodegenAPI
  public double _districtCSSE_DefaultValue_xjal() {
    final Main self = this;
    return
```

```
(double) selectFrom(mortality)
       .where(mortality.type.eq("DistrictCS"))
       .uniqueResult(mortality.sd)
;
  }

  public void set_districtCSSE( double districtCSSE ) {
    if (districtCSSE == this.districtCSSE) {
      return;
    }
    double _oldValue_xjal = this.districtCSSE;
    this.districtCSSE = districtCSSE;
    onChange_districtCSSE_xjal( _oldValue_xjal );
    onChange();
  }

  /**
   * Calls "On change" action for parameter districtCSSE.<br>
   * Note that 'oldValue' in that action will be unavailable if this method is called
by user
   * (current parameter value will be passed as 'oldValue').<br>
   * Please call <code>set_districtCSSE()</code> method instead.
   */
  protected void onChange_districtCSSE() {
    onChange_districtCSSE_xjal( districtCSSE );
  }

  @AnyLogicInternalCodegenAPI
  protected void onChange_districtCSSE_xjal( double oldValue ) {
  }


  public
double  centralNo;

  /**
   * Returns default value for parameter <code>centralNo</code>.
   * <i>This method should not be called by user</i>
   */
  @AnyLogicInternalCodegenAPI
  public double _centralNo_DefaultValue_xjal() {
    final Main self = this;
    return
(double) selectFrom(mortality)
       .where(mortality.type.eq("CentralNo"))
       .uniqueResult(mortality.mort)
;
  }

  public void set_centralNo( double centralNo ) {
    if (centralNo == this.centralNo) {
      return;
    }
    double _oldValue_xjal = this.centralNo;
    this.centralNo = centralNo;
    onChange_centralNo_xjal( _oldValue_xjal );
    onChange();
  }

  /**
   * Calls "On change" action for parameter centralNo.<br>
   * Note that 'oldValue' in that action will be unavailable if this method is called
by user
```

```
   * (current parameter value will be passed as 'oldValue').<br>
   * Please call <code>set_centralNo()</code> method instead.
   */
  protected void onChange_centralNo() {
    onChange_centralNo_xjal( centralNo );
  }

  @AnyLogicInternalCodegenAPI
  protected void onChange_centralNo_xjal( double oldValue ) {
  }


  public
double  clinicNo;

  /**
   * Returns default value for parameter <code>clinicNo</code>.
   * <i>This method should not be called by user</i>
   */
  @AnyLogicInternalCodegenAPI
  public double _clinicNo_DefaultValue_xjal() {
    final Main self = this;
    return
(double) selectFrom(mortality)
       .where(mortality.type.eq("ClinicNo"))
       .uniqueResult(mortality.mort)
;
  }

  public void set_clinicNo( double clinicNo ) {
    if (clinicNo == this.clinicNo) {
      return;
    }
    double _oldValue_xjal = this.clinicNo;
    this.clinicNo = clinicNo;
    onChange_clinicNo_xjal( _oldValue_xjal );
    onChange();
  }

  /**
   * Calls "On change" action for parameter clinicNo.<br>
   * Note that 'oldValue' in that action will be unavailable if this method is called
by user
   * (current parameter value will be passed as 'oldValue').<br>
   * Please call <code>set_clinicNo()</code> method instead.
   */
  protected void onChange_clinicNo() {
    onChange_clinicNo_xjal( clinicNo );
  }

  @AnyLogicInternalCodegenAPI
  protected void onChange_clinicNo_xjal( double oldValue ) {
  }


  public
double  maternityNo;

  /**
   * Returns default value for parameter <code>maternityNo</code>.
   * <i>This method should not be called by user</i>
   */
  @AnyLogicInternalCodegenAPI
```

```
  public double _maternityNo_DefaultValue_xjal() {
    final Main self = this;
    return
(double) selectFrom(mortality)
       .where(mortality.type.eq("MaternityNo"))
       .uniqueResult(mortality.mort)
;
  }

  public void set_maternityNo( double maternityNo ) {
    if (maternityNo == this.maternityNo) {
      return;
    }
    double _oldValue_xjal = this.maternityNo;
    this.maternityNo = maternityNo;
    onChange_maternityNo_xjal( _oldValue_xjal );
    onChange();
  }

  /**
   * Calls "On change" action for parameter maternityNo.<br>
   * Note that 'oldValue' in that action will be unavailable if this method is called
by user
   * (current parameter value will be passed as 'oldValue').<br>
   * Please call <code>set_maternityNo()</code> method instead.
   */
  protected void onChange_maternityNo() {
    onChange_maternityNo_xjal( maternityNo );
  }

  @AnyLogicInternalCodegenAPI
  protected void onChange_maternityNo_xjal( double oldValue ) {
  }


  public
double  healthCenterNo;

  /**
   * Returns default value for parameter <code>healthCenterNo</code>.
   * <i>This method should not be called by user</i>
   */
  @AnyLogicInternalCodegenAPI
  public double _healthCenterNo_DefaultValue_xjal() {
    final Main self = this;
    return
(double) selectFrom(mortality)
       .where(mortality.type.eq("HealthCenterNo"))
       .uniqueResult(mortality.mort)
;
  }

  public void set_healthCenterNo( double healthCenterNo ) {
    if (healthCenterNo == this.healthCenterNo) {
      return;
    }
    double _oldValue_xjal = this.healthCenterNo;
    this.healthCenterNo = healthCenterNo;
    onChange_healthCenterNo_xjal( _oldValue_xjal );
    onChange();
  }

  /**
```

```
   * Calls "On change" action for parameter healthCenterNo.<br>
   * Note that 'oldValue' in that action will be unavailable if this method is called
by user
   * (current parameter value will be passed as 'oldValue').<br>
   * Please call <code>set_healthCenterNo()</code> method instead.
   */
  protected void onChange_healthCenterNo() {
    onChange_healthCenterNo_xjal( healthCenterNo );
  }

  @AnyLogicInternalCodegenAPI
  protected void onChange_healthCenterNo_xjal( double oldValue ) {
  }


  public
double  otherNo;

  /**
   * Returns default value for parameter <code>otherNo</code>.
   * <i>This method should not be called by user</i>
   */
  @AnyLogicInternalCodegenAPI
  public double _otherNo_DefaultValue_xjal() {
    final Main self = this;
    return
(double) selectFrom(mortality)
       .where(mortality.type.eq("OtherNo"))
       .uniqueResult(mortality.mort)
;
  }

  public void set_otherNo( double otherNo ) {
    if (otherNo == this.otherNo) {
      return;
    }
    double _oldValue_xjal = this.otherNo;
    this.otherNo = otherNo;
    onChange_otherNo_xjal( _oldValue_xjal );
    onChange();
  }

  /**
   * Calls "On change" action for parameter otherNo.<br>
   * Note that 'oldValue' in that action will be unavailable if this method is called
by user
   * (current parameter value will be passed as 'oldValue').<br>
   * Please call <code>set_otherNo()</code> method instead.
   */
  protected void onChange_otherNo() {
    onChange_otherNo_xjal( otherNo );
  }

  @AnyLogicInternalCodegenAPI
  protected void onChange_otherNo_xjal( double oldValue ) {
  }


  public
double  communityNo;

  /**
   * Returns default value for parameter <code>communityNo</code>.
```

```
   * <i>This method should not be called by user</i>
   */
  @AnyLogicInternalCodegenAPI
  public double _communityNo_DefaultValue_xjal() {
    final Main self = this;
    return
(double) selectFrom(mortality)
       .where(mortality.type.eq("CommunityNo"))
       .uniqueResult(mortality.mort)
;
  }

  public void set_communityNo( double communityNo ) {
    if (communityNo == this.communityNo) {
      return;
    }
    double _oldValue_xjal = this.communityNo;
    this.communityNo = communityNo;
    onChange_communityNo_xjal( _oldValue_xjal );
    onChange();
  }

  /**
   * Calls "On change" action for parameter communityNo.<br>
   * Note that 'oldValue' in that action will be unavailable if this method is called
by user
   * (current parameter value will be passed as 'oldValue').<br>
   * Please call <code>set_communityNo()</code> method instead.
   */
  protected void onChange_communityNo() {
    onChange_communityNo_xjal( communityNo );
  }

  @AnyLogicInternalCodegenAPI
  protected void onChange_communityNo_xjal( double oldValue ) {
  }


  public
double  districtNo;

  /**
   * Returns default value for parameter <code>districtNo</code>.
   * <i>This method should not be called by user</i>
   */
  @AnyLogicInternalCodegenAPI
  public double _districtNo_DefaultValue_xjal() {
    final Main self = this;
    return
(double) selectFrom(mortality)
       .where(mortality.type.eq("DistrictNo"))
       .uniqueResult(mortality.mort)
;
  }

  public void set_districtNo( double districtNo ) {
    if (districtNo == this.districtNo) {
      return;
    }
    double _oldValue_xjal = this.districtNo;
    this.districtNo = districtNo;
    onChange_districtNo_xjal( _oldValue_xjal );
    onChange();
```

```
  }

  /**
   * Calls "On change" action for parameter districtNo.<br>
   * Note that 'oldValue' in that action will be unavailable if this method is called
by user
   * (current parameter value will be passed as 'oldValue').<br>
   * Please call <code>set_districtNo()</code> method instead.
   */
  protected void onChange_districtNo() {
    onChange_districtNo_xjal( districtNo );
  }

  @AnyLogicInternalCodegenAPI
  protected void onChange_districtNo_xjal( double oldValue ) {
  }


  public
double  centralNoSE;

  /**
   * Returns default value for parameter <code>centralNoSE</code>.
   * <i>This method should not be called by user</i>
   */
  @AnyLogicInternalCodegenAPI
  public double _centralNoSE_DefaultValue_xjal() {
    final Main self = this;
    return
(double) selectFrom(mortality)
      .where(mortality.type.eq("CentralNo"))
      .uniqueResult(mortality.sd)
;
  }

  public void set_centralNoSE( double centralNoSE ) {
    if (centralNoSE == this.centralNoSE) {
      return;
    }
    double _oldValue_xjal = this.centralNoSE;
    this.centralNoSE = centralNoSE;
    onChange_centralNoSE_xjal( _oldValue_xjal );
    onChange();
  }

  /**
   * Calls "On change" action for parameter centralNoSE.<br>
   * Note that 'oldValue' in that action will be unavailable if this method is called
by user
   * (current parameter value will be passed as 'oldValue').<br>
   * Please call <code>set_centralNoSE()</code> method instead.
   */
  protected void onChange_centralNoSE() {
    onChange_centralNoSE_xjal( centralNoSE );
  }

  @AnyLogicInternalCodegenAPI
  protected void onChange_centralNoSE_xjal( double oldValue ) {
  }


  public
double  clinicNoSE;
```

```
  /**
   * Returns default value for parameter <code>clinicNoSE</code>.
   * <i>This method should not be called by user</i>
   */
  @AnyLogicInternalCodegenAPI
  public double _clinicNoSE_DefaultValue_xjal() {
    final Main self = this;
    return
(double) selectFrom(mortality)
      .where(mortality.type.eq("ClinicNo"))
      .uniqueResult(mortality.sd)
;
  }

  public void set_clinicNoSE( double clinicNoSE ) {
    if (clinicNoSE == this.clinicNoSE) {
      return;
    }
    double _oldValue_xjal = this.clinicNoSE;
    this.clinicNoSE = clinicNoSE;
    onChange_clinicNoSE_xjal( _oldValue_xjal );
    onChange();
  }

  /**
   * Calls "On change" action for parameter clinicNoSE.<br>
   * Note that 'oldValue' in that action will be unavailable if this method is called
by user
   * (current parameter value will be passed as 'oldValue').<br>
   * Please call <code>set_clinicNoSE()</code> method instead.
   */
  protected void onChange_clinicNoSE() {
    onChange_clinicNoSE_xjal( clinicNoSE );
  }

  @AnyLogicInternalCodegenAPI
  protected void onChange_clinicNoSE_xjal( double oldValue ) {
  }


  public
double  maternityNoSE;

  /**
   * Returns default value for parameter <code>maternityNoSE</code>.
   * <i>This method should not be called by user</i>
   */
  @AnyLogicInternalCodegenAPI
  public double _maternityNoSE_DefaultValue_xjal() {
    final Main self = this;
    return
(double) selectFrom(mortality)
      .where(mortality.type.eq("MaternityNo"))
      .uniqueResult(mortality.sd)
;
  }

  public void set_maternityNoSE( double maternityNoSE ) {
    if (maternityNoSE == this.maternityNoSE) {
      return;
    }
    double _oldValue_xjal = this.maternityNoSE;
```

```
    this.maternityNoSE = maternityNoSE;
    onChange_maternityNoSE_xjal( _oldValue_xjal );
    onChange();
  }

  /**
   * Calls "On change" action for parameter maternityNoSE.<br>
   * Note that 'oldValue' in that action will be unavailable if this method is called
by user
   * (current parameter value will be passed as 'oldValue').<br>
   * Please call <code>set_maternityNoSE()</code> method instead.
   */
  protected void onChange_maternityNoSE() {
    onChange_maternityNoSE_xjal( maternityNoSE );
  }

  @AnyLogicInternalCodegenAPI
  protected void onChange_maternityNoSE_xjal( double oldValue ) {
  }


  public
double  healthCenterNoSE;

  /**
   * Returns default value for parameter <code>healthCenterNoSE</code>.
   * <i>This method should not be called by user</i>
   */
  @AnyLogicInternalCodegenAPI
  public double _healthCenterNoSE_DefaultValue_xjal() {
    final Main self = this;
    return
(double) selectFrom(mortality)
       .where(mortality.type.eq("HealthCenterNo"))
       .uniqueResult(mortality.sd)
;
  }

  public void set_healthCenterNoSE( double healthCenterNoSE ) {
    if (healthCenterNoSE == this.healthCenterNoSE) {
      return;
    }
    double _oldValue_xjal = this.healthCenterNoSE;
    this.healthCenterNoSE = healthCenterNoSE;
    onChange_healthCenterNoSE_xjal( _oldValue_xjal );
    onChange();
  }

  /**
   * Calls "On change" action for parameter healthCenterNoSE.<br>
   * Note that 'oldValue' in that action will be unavailable if this method is called
by user
   * (current parameter value will be passed as 'oldValue').<br>
   * Please call <code>set_healthCenterNoSE()</code> method instead.
   */
  protected void onChange_healthCenterNoSE() {
    onChange_healthCenterNoSE_xjal( healthCenterNoSE );
  }

  @AnyLogicInternalCodegenAPI
  protected void onChange_healthCenterNoSE_xjal( double oldValue ) {
  }
```

```
  public
double  otherNoSE;

  /**
   * Returns default value for parameter <code>otherNoSE</code>.
   * <i>This method should not be called by user</i>
   */
  @AnyLogicInternalCodegenAPI
  public double _otherNoSE_DefaultValue_xjal() {
    final Main self = this;
    return
(double) selectFrom(mortality)
      .where(mortality.type.eq("OtherNo"))
      .uniqueResult(mortality.sd)
;
  }

  public void set_otherNoSE( double otherNoSE ) {
    if (otherNoSE == this.otherNoSE) {
      return;
    }
    double _oldValue_xjal = this.otherNoSE;
    this.otherNoSE = otherNoSE;
    onChange_otherNoSE_xjal( _oldValue_xjal );
    onChange();
  }

  /**
   * Calls "On change" action for parameter otherNoSE.<br>
   * Note that 'oldValue' in that action will be unavailable if this method is called
by user
   * (current parameter value will be passed as 'oldValue').<br>
   * Please call <code>set_otherNoSE()</code> method instead.
   */
  protected void onChange_otherNoSE() {
    onChange_otherNoSE_xjal( otherNoSE );
  }

  @AnyLogicInternalCodegenAPI
  protected void onChange_otherNoSE_xjal( double oldValue ) {
  }


  public
double  communityNoSE;

  /**
   * Returns default value for parameter <code>communityNoSE</code>.
   * <i>This method should not be called by user</i>
   */
  @AnyLogicInternalCodegenAPI
  public double _communityNoSE_DefaultValue_xjal() {
    final Main self = this;
    return
(double) selectFrom(mortality)
      .where(mortality.type.eq("CommunityNo"))
      .uniqueResult(mortality.sd)
;
  }

  public void set_communityNoSE( double communityNoSE ) {
    if (communityNoSE == this.communityNoSE) {
```

```
      return;
    }
    double _oldValue_xjal = this.communityNoSE;
    this.communityNoSE = communityNoSE;
    onChange_communityNoSE_xjal( _oldValue_xjal );
    onChange();
  }

  /**
   * Calls "On change" action for parameter communityNoSE.<br>
   * Note that 'oldValue' in that action will be unavailable if this method is called
by user
   * (current parameter value will be passed as 'oldValue').<br>
   * Please call <code>set_communityNoSE()</code> method instead.
   */
  protected void onChange_communityNoSE() {
    onChange_communityNoSE_xjal( communityNoSE );
  }

  @AnyLogicInternalCodegenAPI
  protected void onChange_communityNoSE_xjal( double oldValue ) {
  }


  public
double  districtNoSE;

  /**
   * Returns default value for parameter <code>districtNoSE</code>.
   * <i>This method should not be called by user</i>
   */
  @AnyLogicInternalCodegenAPI
  public double _districtNoSE_DefaultValue_xjal() {
    final Main self = this;
    return
(double) selectFrom(mortality)
       .where(mortality.type.eq("DistrictNo"))
       .uniqueResult(mortality.sd)
;
  }

  public void set_districtNoSE( double districtNoSE ) {
    if (districtNoSE == this.districtNoSE) {
      return;
    }
    double _oldValue_xjal = this.districtNoSE;
    this.districtNoSE = districtNoSE;
    onChange_districtNoSE_xjal( _oldValue_xjal );
    onChange();
  }

  /**
   * Calls "On change" action for parameter districtNoSE.<br>
   * Note that 'oldValue' in that action will be unavailable if this method is called
by user
   * (current parameter value will be passed as 'oldValue').<br>
   * Please call <code>set_districtNoSE()</code> method instead.
   */
  protected void onChange_districtNoSE() {
    onChange_districtNoSE_xjal( districtNoSE );
  }

  @AnyLogicInternalCodegenAPI
```

```
  protected void onChange_districtNoSE_xjal( double oldValue ) {
  }


  public
double  central5;

  /**
   * Returns default value for parameter <code>central5</code>.
   * <i>This method should not be called by user</i>
   */
  @AnyLogicInternalCodegenAPI
  public double _central5_DefaultValue_xjal() {
    final Main self = this;
    return
(double) selectFrom(mortality)
       .where(mortality.type.eq("Central5"))
       .uniqueResult(mortality.mort)
;
  }

  public void set_central5( double central5 ) {
    if (central5 == this.central5) {
      return;
    }
    double _oldValue_xjal = this.central5;
    this.central5 = central5;
    onChange_central5_xjal( _oldValue_xjal );
    onChange();
  }

  /**
   * Calls "On change" action for parameter central5.<br>
   * Note that 'oldValue' in that action will be unavailable if this method is called
by user
   * (current parameter value will be passed as 'oldValue').<br>
   * Please call <code>set_central5()</code> method instead.
   */
  protected void onChange_central5() {
    onChange_central5_xjal( central5 );
  }

  @AnyLogicInternalCodegenAPI
  protected void onChange_central5_xjal( double oldValue ) {
  }


  public
double  clinic5;

  /**
   * Returns default value for parameter <code>clinic5</code>.
   * <i>This method should not be called by user</i>
   */
  @AnyLogicInternalCodegenAPI
  public double _clinic5_DefaultValue_xjal() {
    final Main self = this;
    return
(double) selectFrom(mortality)
       .where(mortality.type.eq("Clinic5"))
       .uniqueResult(mortality.mort)
;
  }
```

```
  public void set_clinic5( double clinic5 ) {
    if (clinic5 == this.clinic5) {
      return;
    }
    double _oldValue_xjal = this.clinic5;
    this.clinic5 = clinic5;
    onChange_clinic5_xjal( _oldValue_xjal );
    onChange();
  }

  /**
   * Calls "On change" action for parameter clinic5.<br>
   * Note that 'oldValue' in that action will be unavailable if this method is called
by user
   * (current parameter value will be passed as 'oldValue').<br>
   * Please call <code>set_clinic5()</code> method instead.
   */
  protected void onChange_clinic5() {
    onChange_clinic5_xjal( clinic5 );
  }

  @AnyLogicInternalCodegenAPI
  protected void onChange_clinic5_xjal( double oldValue ) {
  }


  public
double  maternity5;

  /**
   * Returns default value for parameter <code>maternity5</code>.
   * <i>This method should not be called by user</i>
   */
  @AnyLogicInternalCodegenAPI
  public double _maternity5_DefaultValue_xjal() {
    final Main self = this;
    return
(double) selectFrom(mortality)
       .where(mortality.type.eq("Maternity5"))
       .uniqueResult(mortality.mort)
;
  }

  public void set_maternity5( double maternity5 ) {
    if (maternity5 == this.maternity5) {
      return;
    }
    double _oldValue_xjal = this.maternity5;
    this.maternity5 = maternity5;
    onChange_maternity5_xjal( _oldValue_xjal );
    onChange();
  }

  /**
   * Calls "On change" action for parameter maternity5.<br>
   * Note that 'oldValue' in that action will be unavailable if this method is called
by user
   * (current parameter value will be passed as 'oldValue').<br>
   * Please call <code>set_maternity5()</code> method instead.
   */
  protected void onChange_maternity5() {
    onChange_maternity5_xjal( maternity5 );
```

```
  }

  @AnyLogicInternalCodegenAPI
  protected void onChange_maternity5_xjal( double oldValue ) {
  }


  public
double  healthCenter5;

  /**
   * Returns default value for parameter <code>healthCenter5</code>.
   * <i>This method should not be called by user</i>
   */
  @AnyLogicInternalCodegenAPI
  public double _healthCenter5_DefaultValue_xjal() {
    final Main self = this;
    return
(double) selectFrom(mortality)
       .where(mortality.type.eq("HealthCenter5"))
       .uniqueResult(mortality.mort)
;
  }

  public void set_healthCenter5( double healthCenter5 ) {
    if (healthCenter5 == this.healthCenter5) {
      return;
    }
    double _oldValue_xjal = this.healthCenter5;
    this.healthCenter5 = healthCenter5;
    onChange_healthCenter5_xjal( _oldValue_xjal );
    onChange();
  }

  /**
   * Calls "On change" action for parameter healthCenter5.<br>
   * Note that 'oldValue' in that action will be unavailable if this method is called
by user
   * (current parameter value will be passed as 'oldValue').<br>
   * Please call <code>set_healthCenter5()</code> method instead.
   */
  protected void onChange_healthCenter5() {
    onChange_healthCenter5_xjal( healthCenter5 );
  }

  @AnyLogicInternalCodegenAPI
  protected void onChange_healthCenter5_xjal( double oldValue ) {
  }


  public
double  other5;

  /**
   * Returns default value for parameter <code>other5</code>.
   * <i>This method should not be called by user</i>
   */
  @AnyLogicInternalCodegenAPI
  public double _other5_DefaultValue_xjal() {
    final Main self = this;
    return
(double) selectFrom(mortality)
       .where(mortality.type.eq("Other5"))
```

```
        .uniqueResult(mortality.mort)
;
  }

  public void set_other5( double other5 ) {
    if (other5 == this.other5) {
      return;
    }
    double _oldValue_xjal = this.other5;
    this.other5 = other5;
    onChange_other5_xjal( _oldValue_xjal );
    onChange();
  }

  /**
   * Calls "On change" action for parameter other5.<br>
   * Note that 'oldValue' in that action will be unavailable if this method is called
by user
   * (current parameter value will be passed as 'oldValue').<br>
   * Please call <code>set_other5()</code> method instead.
   */
  protected void onChange_other5() {
    onChange_other5_xjal( other5 );
  }

  @AnyLogicInternalCodegenAPI
  protected void onChange_other5_xjal( double oldValue ) {
  }


  public
double  community5;

  /**
   * Returns default value for parameter <code>community5</code>.
   * <i>This method should not be called by user</i>
   */
  @AnyLogicInternalCodegenAPI
  public double _community5_DefaultValue_xjal() {
    final Main self = this;
    return
(double) selectFrom(mortality)
        .where(mortality.type.eq("Community5"))
        .uniqueResult(mortality.mort)
;
  }

  public void set_community5( double community5 ) {
    if (community5 == this.community5) {
      return;
    }
    double _oldValue_xjal = this.community5;
    this.community5 = community5;
    onChange_community5_xjal( _oldValue_xjal );
    onChange();
  }

  /**
   * Calls "On change" action for parameter community5.<br>
   * Note that 'oldValue' in that action will be unavailable if this method is called
by user
   * (current parameter value will be passed as 'oldValue').<br>
   * Please call <code>set_community5()</code> method instead.
```

```
   */
  protected void onChange_community5() {
    onChange_community5_xjal( community5 );
  }

  @AnyLogicInternalCodegenAPI
  protected void onChange_community5_xjal( double oldValue ) {
  }


  public
double  district5;

  /**
   * Returns default value for parameter <code>district5</code>.
   * <i>This method should not be called by user</i>
   */
  @AnyLogicInternalCodegenAPI
  public double _district5_DefaultValue_xjal() {
    final Main self = this;
    return
(double) selectFrom(mortality)
       .where(mortality.type.eq("District5"))
       .uniqueResult(mortality.mort)
;
  }

  public void set_district5( double district5 ) {
    if (district5 == this.district5) {
      return;
    }
    double _oldValue_xjal = this.district5;
    this.district5 = district5;
    onChange_district5_xjal( _oldValue_xjal );
    onChange();
  }

  /**
   * Calls "On change" action for parameter district5.<br>
   * Note that 'oldValue' in that action will be unavailable if this method is called
by user
   * (current parameter value will be passed as 'oldValue').<br>
   * Please call <code>set_district5()</code> method instead.
   */
  protected void onChange_district5() {
    onChange_district5_xjal( district5 );
  }

  @AnyLogicInternalCodegenAPI
  protected void onChange_district5_xjal( double oldValue ) {
  }


  public
double  central5SE;

  /**
   * Returns default value for parameter <code>central5SE</code>.
   * <i>This method should not be called by user</i>
   */
  @AnyLogicInternalCodegenAPI
  public double _central5SE_DefaultValue_xjal() {
    final Main self = this;
```

```
    return
(double) selectFrom(mortality)
       .where(mortality.type.eq("Central5"))
       .uniqueResult(mortality.sd)
;
  }

  public void set_central5SE( double central5SE ) {
    if (central5SE == this.central5SE) {
      return;
    }
    double _oldValue_xjal = this.central5SE;
    this.central5SE = central5SE;
    onChange_central5SE_xjal( _oldValue_xjal );
    onChange();
  }

  /**
   * Calls "On change" action for parameter central5SE.<br>
   * Note that 'oldValue' in that action will be unavailable if this method is called
by user
   * (current parameter value will be passed as 'oldValue').<br>
   * Please call <code>set_central5SE()</code> method instead.
   */
  protected void onChange_central5SE() {
    onChange_central5SE_xjal( central5SE );
  }

  @AnyLogicInternalCodegenAPI
  protected void onChange_central5SE_xjal( double oldValue ) {
  }


  public
double  clinic5SE;

  /**
   * Returns default value for parameter <code>clinic5SE</code>.
   * <i>This method should not be called by user</i>
   */
  @AnyLogicInternalCodegenAPI
  public double _clinic5SE_DefaultValue_xjal() {
    final Main self = this;
    return
(double) selectFrom(mortality)
       .where(mortality.type.eq("Clinic5"))
       .uniqueResult(mortality.sd)
;
  }

  public void set_clinic5SE( double clinic5SE ) {
    if (clinic5SE == this.clinic5SE) {
      return;
    }
    double _oldValue_xjal = this.clinic5SE;
    this.clinic5SE = clinic5SE;
    onChange_clinic5SE_xjal( _oldValue_xjal );
    onChange();
  }

  /**
   * Calls "On change" action for parameter clinic5SE.<br>
```

```
   * Note that 'oldValue' in that action will be unavailable if this method is called
by user
   * (current parameter value will be passed as 'oldValue').<br>
   * Please call <code>set_clinic5SE()</code> method instead.
   */
  protected void onChange_clinic5SE() {
    onChange_clinic5SE_xjal( clinic5SE );
  }

  @AnyLogicInternalCodegenAPI
  protected void onChange_clinic5SE_xjal( double oldValue ) {
  }


  public
double  maternity5SE;

  /**
   * Returns default value for parameter <code>maternity5SE</code>.
   * <i>This method should not be called by user</i>
   */
  @AnyLogicInternalCodegenAPI
  public double _maternity5SE_DefaultValue_xjal() {
    final Main self = this;
    return
(double) selectFrom(mortality)
       .where(mortality.type.eq("Maternity5"))
       .uniqueResult(mortality.sd)
;
  }

  public void set_maternity5SE( double maternity5SE ) {
    if (maternity5SE == this.maternity5SE) {
      return;
    }
    double _oldValue_xjal = this.maternity5SE;
    this.maternity5SE = maternity5SE;
    onChange_maternity5SE_xjal( _oldValue_xjal );
    onChange();
  }

  /**
   * Calls "On change" action for parameter maternity5SE.<br>
   * Note that 'oldValue' in that action will be unavailable if this method is called
by user
   * (current parameter value will be passed as 'oldValue').<br>
   * Please call <code>set_maternity5SE()</code> method instead.
   */
  protected void onChange_maternity5SE() {
    onChange_maternity5SE_xjal( maternity5SE );
  }

  @AnyLogicInternalCodegenAPI
  protected void onChange_maternity5SE_xjal( double oldValue ) {
  }


  public
double  healthCenter5SE;

  /**
   * Returns default value for parameter <code>healthCenter5SE</code>.
   * <i>This method should not be called by user</i>
```

```
   */
  @AnyLogicInternalCodegenAPI
  public double _healthCenter5SE_DefaultValue_xjal() {
    final Main self = this;
    return
(double) selectFrom(mortality)
       .where(mortality.type.eq("HealthCenter5"))
       .uniqueResult(mortality.sd)
;
  }

  public void set_healthCenter5SE( double healthCenter5SE ) {
    if (healthCenter5SE == this.healthCenter5SE) {
      return;
    }
    double _oldValue_xjal = this.healthCenter5SE;
    this.healthCenter5SE = healthCenter5SE;
    onChange_healthCenter5SE_xjal( _oldValue_xjal );
    onChange();
  }

  /**
   * Calls "On change" action for parameter healthCenter5SE.<br>
   * Note that 'oldValue' in that action will be unavailable if this method is called
by user
   * (current parameter value will be passed as 'oldValue').<br>
   * Please call <code>set_healthCenter5SE()</code> method instead.
   */
  protected void onChange_healthCenter5SE() {
    onChange_healthCenter5SE_xjal( healthCenter5SE );
  }

  @AnyLogicInternalCodegenAPI
  protected void onChange_healthCenter5SE_xjal( double oldValue ) {
  }


  public
double  other5SE;

  /**
   * Returns default value for parameter <code>other5SE</code>.
   * <i>This method should not be called by user</i>
   */
  @AnyLogicInternalCodegenAPI
  public double _other5SE_DefaultValue_xjal() {
    final Main self = this;
    return
(double) selectFrom(mortality)
       .where(mortality.type.eq("Other5"))
       .uniqueResult(mortality.sd)
;
  }

  public void set_other5SE( double other5SE ) {
    if (other5SE == this.other5SE) {
      return;
    }
    double _oldValue_xjal = this.other5SE;
    this.other5SE = other5SE;
    onChange_other5SE_xjal( _oldValue_xjal );
    onChange();
  }
```

```
  /**
   * Calls "On change" action for parameter other5SE.<br>
   * Note that 'oldValue' in that action will be unavailable if this method is called
by user
   * (current parameter value will be passed as 'oldValue').<br>
   * Please call <code>set_other5SE()</code> method instead.
   */
  protected void onChange_other5SE() {
    onChange_other5SE_xjal( other5SE );
  }

  @AnyLogicInternalCodegenAPI
  protected void onChange_other5SE_xjal( double oldValue ) {
  }


  public
double  community5SE;

  /**
   * Returns default value for parameter <code>community5SE</code>.
   * <i>This method should not be called by user</i>
   */
  @AnyLogicInternalCodegenAPI
  public double _community5SE_DefaultValue_xjal() {
    final Main self = this;
    return
(double) selectFrom(mortality)
      .where(mortality.type.eq("Community5"))
      .uniqueResult(mortality.sd)
;
  }

  public void set_community5SE( double community5SE ) {
    if (community5SE == this.community5SE) {
      return;
    }
    double _oldValue_xjal = this.community5SE;
    this.community5SE = community5SE;
    onChange_community5SE_xjal( _oldValue_xjal );
    onChange();
  }

  /**
   * Calls "On change" action for parameter community5SE.<br>
   * Note that 'oldValue' in that action will be unavailable if this method is called
by user
   * (current parameter value will be passed as 'oldValue').<br>
   * Please call <code>set_community5SE()</code> method instead.
   */
  protected void onChange_community5SE() {
    onChange_community5SE_xjal( community5SE );
  }

  @AnyLogicInternalCodegenAPI
  protected void onChange_community5SE_xjal( double oldValue ) {
  }


  public
double  district5SE;
```

```
  /**
   * Returns default value for parameter <code>district5SE</code>.
   * <i>This method should not be called by user</i>
   */
  @AnyLogicInternalCodegenAPI
  public double _district5SE_DefaultValue_xjal() {
    final Main self = this;
    return
(double) selectFrom(mortality)
       .where(mortality.type.eq("District5"))
       .uniqueResult(mortality.sd)
;
  }

  public void set_district5SE( double district5SE ) {
    if (district5SE == this.district5SE) {
      return;
    }
    double _oldValue_xjal = this.district5SE;
    this.district5SE = district5SE;
    onChange_district5SE_xjal( _oldValue_xjal );
    onChange();
  }

  /**
   * Calls "On change" action for parameter district5SE.<br>
   * Note that 'oldValue' in that action will be unavailable if this method is called
by user
   * (current parameter value will be passed as 'oldValue').<br>
   * Please call <code>set_district5SE()</code> method instead.
   */
  protected void onChange_district5SE() {
    onChange_district5SE_xjal( district5SE );
  }

  @AnyLogicInternalCodegenAPI
  protected void onChange_district5SE_xjal( double oldValue ) {
  }


  public
double  centralNo5;

  /**
   * Returns default value for parameter <code>centralNo5</code>.
   * <i>This method should not be called by user</i>
   */
  @AnyLogicInternalCodegenAPI
  public double _centralNo5_DefaultValue_xjal() {
    final Main self = this;
    return
(double) selectFrom(mortality)
       .where(mortality.type.eq("CentralNo5"))
       .uniqueResult(mortality.mort)
;
  }

  public void set_centralNo5( double centralNo5 ) {
    if (centralNo5 == this.centralNo5) {
      return;
    }
    double _oldValue_xjal = this.centralNo5;
    this.centralNo5 = centralNo5;
```

```
    onChange_centralNo5_xjal( _oldValue_xjal );
    onChange();
  }

  /**
   * Calls "On change" action for parameter centralNo5.<br>
   * Note that 'oldValue' in that action will be unavailable if this method is called
by user
   * (current parameter value will be passed as 'oldValue').<br>
   * Please call <code>set_centralNo5()</code> method instead.
   */
  protected void onChange_centralNo5() {
    onChange_centralNo5_xjal( centralNo5 );
  }

  @AnyLogicInternalCodegenAPI
  protected void onChange_centralNo5_xjal( double oldValue ) {
  }


  public
double  clinicNo5;

  /**
   * Returns default value for parameter <code>clinicNo5</code>.
   * <i>This method should not be called by user</i>
   */
  @AnyLogicInternalCodegenAPI
  public double _clinicNo5_DefaultValue_xjal() {
    final Main self = this;
    return
(double) selectFrom(mortality)
      .where(mortality.type.eq("ClinicNo5"))
      .uniqueResult(mortality.mort)
;
  }

  public void set_clinicNo5( double clinicNo5 ) {
    if (clinicNo5 == this.clinicNo5) {
      return;
    }
    double _oldValue_xjal = this.clinicNo5;
    this.clinicNo5 = clinicNo5;
    onChange_clinicNo5_xjal( _oldValue_xjal );
    onChange();
  }

  /**
   * Calls "On change" action for parameter clinicNo5.<br>
   * Note that 'oldValue' in that action will be unavailable if this method is called
by user
   * (current parameter value will be passed as 'oldValue').<br>
   * Please call <code>set_clinicNo5()</code> method instead.
   */
  protected void onChange_clinicNo5() {
    onChange_clinicNo5_xjal( clinicNo5 );
  }

  @AnyLogicInternalCodegenAPI
  protected void onChange_clinicNo5_xjal( double oldValue ) {
  }
```

```
  public
double  maternityNo5;

  /**
   * Returns default value for parameter <code>maternityNo5</code>.
   * <i>This method should not be called by user</i>
   */
  @AnyLogicInternalCodegenAPI
  public double _maternityNo5_DefaultValue_xjal() {
    final Main self = this;
    return
(double) selectFrom(mortality)
       .where(mortality.type.eq("MaternityNo5"))
       .uniqueResult(mortality.mort)
;
  }

  public void set_maternityNo5( double maternityNo5 ) {
    if (maternityNo5 == this.maternityNo5) {
      return;
    }
    double _oldValue_xjal = this.maternityNo5;
    this.maternityNo5 = maternityNo5;
    onChange_maternityNo5_xjal( _oldValue_xjal );
    onChange();
  }

  /**
   * Calls "On change" action for parameter maternityNo5.<br>
   * Note that 'oldValue' in that action will be unavailable if this method is called
by user
   * (current parameter value will be passed as 'oldValue').<br>
   * Please call <code>set_maternityNo5()</code> method instead.
   */
  protected void onChange_maternityNo5() {
    onChange_maternityNo5_xjal( maternityNo5 );
  }

  @AnyLogicInternalCodegenAPI
  protected void onChange_maternityNo5_xjal( double oldValue ) {
  }


  public
double  healthCenterNo5;

  /**
   * Returns default value for parameter <code>healthCenterNo5</code>.
   * <i>This method should not be called by user</i>
   */
  @AnyLogicInternalCodegenAPI
  public double _healthCenterNo5_DefaultValue_xjal() {
    final Main self = this;
    return
(double) selectFrom(mortality)
       .where(mortality.type.eq("HealthCenterNo5"))
       .uniqueResult(mortality.mort)
;
  }

  public void set_healthCenterNo5( double healthCenterNo5 ) {
    if (healthCenterNo5 == this.healthCenterNo5) {
      return;
```

```
    }
    double _oldValue_xjal = this.healthCenterNo5;
    this.healthCenterNo5 = healthCenterNo5;
    onChange_healthCenterNo5_xjal( _oldValue_xjal );
    onChange();
  }

  /**
   * Calls "On change" action for parameter healthCenterNo5.<br>
   * Note that 'oldValue' in that action will be unavailable if this method is called
by user
   * (current parameter value will be passed as 'oldValue').<br>
   * Please call <code>set_healthCenterNo5()</code> method instead.
   */
  protected void onChange_healthCenterNo5() {
    onChange_healthCenterNo5_xjal( healthCenterNo5 );
  }

  @AnyLogicInternalCodegenAPI
  protected void onChange_healthCenterNo5_xjal( double oldValue ) {
  }


  public
double  otherNo5;

  /**
   * Returns default value for parameter <code>otherNo5</code>.
   * <i>This method should not be called by user</i>
   */
  @AnyLogicInternalCodegenAPI
  public double _otherNo5_DefaultValue_xjal() {
    final Main self = this;
    return
(double) selectFrom(mortality)
      .where(mortality.type.eq("OtherNo5"))
      .uniqueResult(mortality.mort)
;
  }

  public void set_otherNo5( double otherNo5 ) {
    if (otherNo5 == this.otherNo5) {
      return;
    }
    double _oldValue_xjal = this.otherNo5;
    this.otherNo5 = otherNo5;
    onChange_otherNo5_xjal( _oldValue_xjal );
    onChange();
  }

  /**
   * Calls "On change" action for parameter otherNo5.<br>
   * Note that 'oldValue' in that action will be unavailable if this method is called
by user
   * (current parameter value will be passed as 'oldValue').<br>
   * Please call <code>set_otherNo5()</code> method instead.
   */
  protected void onChange_otherNo5() {
    onChange_otherNo5_xjal( otherNo5 );
  }

  @AnyLogicInternalCodegenAPI
  protected void onChange_otherNo5_xjal( double oldValue ) {
```

```
  }


  public
double  communityNo5;

  /**
   * Returns default value for parameter <code>communityNo5</code>.
   * <i>This method should not be called by user</i>
   */
  @AnyLogicInternalCodegenAPI
  public double _communityNo5_DefaultValue_xjal() {
    final Main self = this;
    return
(double) selectFrom(mortality)
       .where(mortality.type.eq("CommunityNo5"))
       .uniqueResult(mortality.mort)
;
  }

  public void set_communityNo5( double communityNo5 ) {
    if (communityNo5 == this.communityNo5) {
      return;
    }
    double _oldValue_xjal = this.communityNo5;
    this.communityNo5 = communityNo5;
    onChange_communityNo5_xjal( _oldValue_xjal );
    onChange();
  }

  /**
   * Calls "On change" action for parameter communityNo5.<br>
   * Note that 'oldValue' in that action will be unavailable if this method is called
by user
   * (current parameter value will be passed as 'oldValue').<br>
   * Please call <code>set_communityNo5()</code> method instead.
   */
  protected void onChange_communityNo5() {
    onChange_communityNo5_xjal( communityNo5 );
  }

  @AnyLogicInternalCodegenAPI
  protected void onChange_communityNo5_xjal( double oldValue ) {
  }


  public
double  districtNo5;

  /**
   * Returns default value for parameter <code>districtNo5</code>.
   * <i>This method should not be called by user</i>
   */
  @AnyLogicInternalCodegenAPI
  public double _districtNo5_DefaultValue_xjal() {
    final Main self = this;
    return
(double) selectFrom(mortality)
       .where(mortality.type.eq("DistrictNo5"))
       .uniqueResult(mortality.mort)
;
  }
```

```
  public void set_districtNo5( double districtNo5 ) {
    if (districtNo5 == this.districtNo5) {
      return;
    }
    double _oldValue_xjal = this.districtNo5;
    this.districtNo5 = districtNo5;
    onChange_districtNo5_xjal( _oldValue_xjal );
    onChange();
  }

  /**
   * Calls "On change" action for parameter districtNo5.<br>
   * Note that 'oldValue' in that action will be unavailable if this method is called
by user
   * (current parameter value will be passed as 'oldValue').<br>
   * Please call <code>set_districtNo5()</code> method instead.
   */
  protected void onChange_districtNo5() {
    onChange_districtNo5_xjal( districtNo5 );
  }

  @AnyLogicInternalCodegenAPI
  protected void onChange_districtNo5_xjal( double oldValue ) {
  }


  public
double  centralNo5SE;

  /**
   * Returns default value for parameter <code>centralNo5SE</code>.
   * <i>This method should not be called by user</i>
   */
  @AnyLogicInternalCodegenAPI
  public double _centralNo5SE_DefaultValue_xjal() {
    final Main self = this;
    return
(double) selectFrom(mortality)
       .where(mortality.type.eq("CentralNo5"))
       .uniqueResult(mortality.sd)
;
  }

  public void set_centralNo5SE( double centralNo5SE ) {
    if (centralNo5SE == this.centralNo5SE) {
      return;
    }
    double _oldValue_xjal = this.centralNo5SE;
    this.centralNo5SE = centralNo5SE;
    onChange_centralNo5SE_xjal( _oldValue_xjal );
    onChange();
  }

  /**
   * Calls "On change" action for parameter centralNo5SE.<br>
   * Note that 'oldValue' in that action will be unavailable if this method is called
by user
   * (current parameter value will be passed as 'oldValue').<br>
   * Please call <code>set_centralNo5SE()</code> method instead.
   */
  protected void onChange_centralNo5SE() {
    onChange_centralNo5SE_xjal( centralNo5SE );
  }
```

```
  @AnyLogicInternalCodegenAPI
  protected void onChange_centralNo5SE_xjal( double oldValue ) {
  }


  public
double  clinicNo5SE;

  /**
   * Returns default value for parameter <code>clinicNo5SE</code>.
   * <i>This method should not be called by user</i>
   */
  @AnyLogicInternalCodegenAPI
  public double _clinicNo5SE_DefaultValue_xjal() {
    final Main self = this;
    return
(double) selectFrom(mortality)
       .where(mortality.type.eq("ClinicNo5"))
       .uniqueResult(mortality.sd)
;
  }

  public void set_clinicNo5SE( double clinicNo5SE ) {
    if (clinicNo5SE == this.clinicNo5SE) {
      return;
    }
    double _oldValue_xjal = this.clinicNo5SE;
    this.clinicNo5SE = clinicNo5SE;
    onChange_clinicNo5SE_xjal( _oldValue_xjal );
    onChange();
  }

  /**
   * Calls "On change" action for parameter clinicNo5SE.<br>
   * Note that 'oldValue' in that action will be unavailable if this method is called
by user
   * (current parameter value will be passed as 'oldValue').<br>
   * Please call <code>set_clinicNo5SE()</code> method instead.
   */
  protected void onChange_clinicNo5SE() {
    onChange_clinicNo5SE_xjal( clinicNo5SE );
  }

  @AnyLogicInternalCodegenAPI
  protected void onChange_clinicNo5SE_xjal( double oldValue ) {
  }


  public
double  maternityNo5SE;

  /**
   * Returns default value for parameter <code>maternityNo5SE</code>.
   * <i>This method should not be called by user</i>
   */
  @AnyLogicInternalCodegenAPI
  public double _maternityNo5SE_DefaultValue_xjal() {
    final Main self = this;
    return
(double) selectFrom(mortality)
       .where(mortality.type.eq("MaternityNo5"))
       .uniqueResult(mortality.sd)
```

```
;
  }

  public void set_maternityNo5SE( double maternityNo5SE ) {
    if (maternityNo5SE == this.maternityNo5SE) {
      return;
    }
    double _oldValue_xjal = this.maternityNo5SE;
    this.maternityNo5SE = maternityNo5SE;
    onChange_maternityNo5SE_xjal( _oldValue_xjal );
    onChange();
  }

  /**
   * Calls "On change" action for parameter maternityNo5SE.<br>
   * Note that 'oldValue' in that action will be unavailable if this method is called
by user
   * (current parameter value will be passed as 'oldValue').<br>
   * Please call <code>set_maternityNo5SE()</code> method instead.
   */
  protected void onChange_maternityNo5SE() {
    onChange_maternityNo5SE_xjal( maternityNo5SE );
  }

  @AnyLogicInternalCodegenAPI
  protected void onChange_maternityNo5SE_xjal( double oldValue ) {
  }


  public
double  healthCenterNo5SE;

  /**
   * Returns default value for parameter <code>healthCenterNo5SE</code>.
   * <i>This method should not be called by user</i>
   */
  @AnyLogicInternalCodegenAPI
  public double _healthCenterNo5SE_DefaultValue_xjal() {
    final Main self = this;
    return
(double) selectFrom(mortality)
       .where(mortality.type.eq("HealthCenterNo5"))
       .uniqueResult(mortality.sd)
;
  }

  public void set_healthCenterNo5SE( double healthCenterNo5SE ) {
    if (healthCenterNo5SE == this.healthCenterNo5SE) {
      return;
    }
    double _oldValue_xjal = this.healthCenterNo5SE;
    this.healthCenterNo5SE = healthCenterNo5SE;
    onChange_healthCenterNo5SE_xjal( _oldValue_xjal );
    onChange();
  }

  /**
   * Calls "On change" action for parameter healthCenterNo5SE.<br>
   * Note that 'oldValue' in that action will be unavailable if this method is called
by user
   * (current parameter value will be passed as 'oldValue').<br>
   * Please call <code>set_healthCenterNo5SE()</code> method instead.
   */
```

```
  protected void onChange_healthCenterNo5SE() {
    onChange_healthCenterNo5SE_xjal( healthCenterNo5SE );
  }

  @AnyLogicInternalCodegenAPI
  protected void onChange_healthCenterNo5SE_xjal( double oldValue ) {
  }


  public
double  otherNo5SE;

  /**
   * Returns default value for parameter <code>otherNo5SE</code>.
   * <i>This method should not be called by user</i>
   */
  @AnyLogicInternalCodegenAPI
  public double _otherNo5SE_DefaultValue_xjal() {
    final Main self = this;
    return
(double) selectFrom(mortality)
      .where(mortality.type.eq("OtherNo5"))
      .uniqueResult(mortality.sd)
;
  }

  public void set_otherNo5SE( double otherNo5SE ) {
    if (otherNo5SE == this.otherNo5SE) {
      return;
    }
    double _oldValue_xjal = this.otherNo5SE;
    this.otherNo5SE = otherNo5SE;
    onChange_otherNo5SE_xjal( _oldValue_xjal );
    onChange();
  }

  /**
   * Calls "On change" action for parameter otherNo5SE.<br>
   * Note that 'oldValue' in that action will be unavailable if this method is called
by user
   * (current parameter value will be passed as 'oldValue').<br>
   * Please call <code>set_otherNo5SE()</code> method instead.
   */
  protected void onChange_otherNo5SE() {
    onChange_otherNo5SE_xjal( otherNo5SE );
  }

  @AnyLogicInternalCodegenAPI
  protected void onChange_otherNo5SE_xjal( double oldValue ) {
  }


  public
double  communityNo5SE;

  /**
   * Returns default value for parameter <code>communityNo5SE</code>.
   * <i>This method should not be called by user</i>
   */
  @AnyLogicInternalCodegenAPI
  public double _communityNo5SE_DefaultValue_xjal() {
    final Main self = this;
    return
```

```
(double) selectFrom(mortality)
       .where(mortality.type.eq("CommunityNo5"))
       .uniqueResult(mortality.sd)
;
  }

  public void set_communityNo5SE( double communityNo5SE ) {
    if (communityNo5SE == this.communityNo5SE) {
      return;
    }
    double _oldValue_xjal = this.communityNo5SE;
    this.communityNo5SE = communityNo5SE;
    onChange_communityNo5SE_xjal( _oldValue_xjal );
    onChange();
  }

  /**
   * Calls "On change" action for parameter communityNo5SE.<br>
   * Note that 'oldValue' in that action will be unavailable if this method is called
by user
   * (current parameter value will be passed as 'oldValue').<br>
   * Please call <code>set_communityNo5SE()</code> method instead.
   */
  protected void onChange_communityNo5SE() {
    onChange_communityNo5SE_xjal( communityNo5SE );
  }

  @AnyLogicInternalCodegenAPI
  protected void onChange_communityNo5SE_xjal( double oldValue ) {
  }


  public
double  districtNo5SE;

  /**
   * Returns default value for parameter <code>districtNo5SE</code>.
   * <i>This method should not be called by user</i>
   */
  @AnyLogicInternalCodegenAPI
  public double _districtNo5SE_DefaultValue_xjal() {
    final Main self = this;
    return
(double) selectFrom(mortality)
       .where(mortality.type.eq("DistrictNo5"))
       .uniqueResult(mortality.sd)
;
  }

  public void set_districtNo5SE( double districtNo5SE ) {
    if (districtNo5SE == this.districtNo5SE) {
      return;
    }
    double _oldValue_xjal = this.districtNo5SE;
    this.districtNo5SE = districtNo5SE;
    onChange_districtNo5SE_xjal( _oldValue_xjal );
    onChange();
  }

  /**
   * Calls "On change" action for parameter districtNo5SE.<br>
   * Note that 'oldValue' in that action will be unavailable if this method is called
by user
```

```
   * (current parameter value will be passed as 'oldValue').<br>
   * Please call <code>set_districtNo5SE()</code> method instead.
   */
  protected void onChange_districtNo5SE() {
    onChange_districtNo5SE_xjal( districtNo5SE );
  }

  @AnyLogicInternalCodegenAPI
  protected void onChange_districtNo5SE_xjal( double oldValue ) {
  }


  public
double  anc4;

  /**
   * Returns default value for parameter <code>anc4</code>.
   * <i>This method should not be called by user</i>
   */
  @AnyLogicInternalCodegenAPI
  public double _anc4_DefaultValue_xjal() {
    final Main self = this;
    return
(double) selectFrom(parameters)
       .where(parameters.parameter.eq("anc4"))
       .uniqueResult(parameters.starting_value)
;
  }

  public void set_anc4( double anc4 ) {
    if (anc4 == this.anc4) {
      return;
    }
    double _oldValue_xjal = this.anc4;
    this.anc4 = anc4;
    onChange_anc4_xjal( _oldValue_xjal );
    onChange();
  }

  /**
   * Calls "On change" action for parameter anc4.<br>
   * Note that 'oldValue' in that action will be unavailable if this method is called
by user
   * (current parameter value will be passed as 'oldValue').<br>
   * Please call <code>set_anc4()</code> method instead.
   */
  protected void onChange_anc4() {
    onChange_anc4_xjal( anc4 );
  }

  @AnyLogicInternalCodegenAPI
  protected void onChange_anc4_xjal( double oldValue ) {
  }


  public
double  unwanted;

  /**
   * Returns default value for parameter <code>unwanted</code>.
   * <i>This method should not be called by user</i>
   */
  @AnyLogicInternalCodegenAPI
```

```
  public double _unwanted_DefaultValue_xjal() {
    final Main self = this;
    return
(double) selectFrom(parameters)
       .where(parameters.parameter.eq("unwanted"))
       .uniqueResult(parameters.starting_value)
;
  }

  public void set_unwanted( double unwanted ) {
    if (unwanted == this.unwanted) {
      return;
    }
    double _oldValue_xjal = this.unwanted;
    this.unwanted = unwanted;
    onChange_unwanted_xjal( _oldValue_xjal );
    onChange();
  }

  /**
   * Calls "On change" action for parameter unwanted.<br>
   * Note that 'oldValue' in that action will be unavailable if this method is called
by user
   * (current parameter value will be passed as 'oldValue').<br>
   * Please call <code>set_unwanted()</code> method instead.
   */
  protected void onChange_unwanted() {
    onChange_unwanted_xjal( unwanted );
  }

  @AnyLogicInternalCodegenAPI
  protected void onChange_unwanted_xjal( double oldValue ) {
  }


  public
double  risk;

  /**
   * Returns default value for parameter <code>risk</code>.
   * <i>This method should not be called by user</i>
   */
  @AnyLogicInternalCodegenAPI
  public double _risk_DefaultValue_xjal() {
    final Main self = this;
    return
(double) selectFrom(parameters)
       .where(parameters.parameter.eq("risk"))
       .uniqueResult(parameters.starting_value)
;
  }

  public void set_risk( double risk ) {
    if (risk == this.risk) {
      return;
    }
    double _oldValue_xjal = this.risk;
    this.risk = risk;
    onChange_risk_xjal( _oldValue_xjal );
    onChange();
  }

  /**
```

```
   * Calls "On change" action for parameter risk.<br>
   * Note that 'oldValue' in that action will be unavailable if this method is called
by user
   * (current parameter value will be passed as 'oldValue').<br>
   * Please call <code>set_risk()</code> method instead.
   */
  protected void onChange_risk() {
    onChange_risk_xjal( risk );
  }

  @AnyLogicInternalCodegenAPI
  protected void onChange_risk_xjal( double oldValue ) {
  }


  public
double  urban;

  /**
   * Returns default value for parameter <code>urban</code>.
   * <i>This method should not be called by user</i>
   */
  @AnyLogicInternalCodegenAPI
  public double _urban_DefaultValue_xjal() {
    final Main self = this;
    return
(double) selectFrom(parameters)
       .where(parameters.parameter.eq("urban"))
       .uniqueResult(parameters.starting_value)
;
  }

  public void set_urban( double urban ) {
    if (urban == this.urban) {
      return;
    }
    double _oldValue_xjal = this.urban;
    this.urban = urban;
    onChange_urban_xjal( _oldValue_xjal );
    onChange();
  }

  /**
   * Calls "On change" action for parameter urban.<br>
   * Note that 'oldValue' in that action will be unavailable if this method is called
by user
   * (current parameter value will be passed as 'oldValue').<br>
   * Please call <code>set_urban()</code> method instead.
   */
  protected void onChange_urban() {
    onChange_urban_xjal( urban );
  }

  @AnyLogicInternalCodegenAPI
  protected void onChange_urban_xjal( double oldValue ) {
  }


  public
double  twins;

  /**
   * Returns default value for parameter <code>twins</code>.
```

```
   * <i>This method should not be called by user</i>
   */
  @AnyLogicInternalCodegenAPI
  public double _twins_DefaultValue_xjal() {
    final Main self = this;
    return
(double) selectFrom(parameters)
       .where(parameters.parameter.eq("twins"))
       .uniqueResult(parameters.starting_value)
;
  }

  public void set_twins( double twins ) {
    if (twins == this.twins) {
      return;
    }
    double _oldValue_xjal = this.twins;
    this.twins = twins;
    onChange_twins_xjal( _oldValue_xjal );
    onChange();
  }

  /**
   * Calls "On change" action for parameter twins.<br>
   * Note that 'oldValue' in that action will be unavailable if this method is called
by user
   * (current parameter value will be passed as 'oldValue').<br>
   * Please call <code>set_twins()</code> method instead.
   */
  protected void onChange_twins() {
    onChange_twins_xjal( twins );
  }

  @AnyLogicInternalCodegenAPI
  protected void onChange_twins_xjal( double oldValue ) {
  }


  public
double  educSec;

  /**
   * Returns default value for parameter <code>educSec</code>.
   * <i>This method should not be called by user</i>
   */
  @AnyLogicInternalCodegenAPI
  public double _educSec_DefaultValue_xjal() {
    final Main self = this;
    return
(double) selectFrom(parameters)
       .where(parameters.parameter.eq("educsec"))
       .uniqueResult(parameters.starting_value)
;
  }

  public void set_educSec( double educSec ) {
    if (educSec == this.educSec) {
      return;
    }
    double _oldValue_xjal = this.educSec;
    this.educSec = educSec;
    onChange_educSec_xjal( _oldValue_xjal );
    onChange();
```

```
  }

  /**
   * Calls "On change" action for parameter educSec.<br>
   * Note that 'oldValue' in that action will be unavailable if this method is called
by user
   * (current parameter value will be passed as 'oldValue').<br>
   * Please call <code>set_educSec()</code> method instead.
   */
  protected void onChange_educSec() {
    onChange_educSec_xjal( educSec );
  }

  @AnyLogicInternalCodegenAPI
  protected void onChange_educSec_xjal( double oldValue ) {
  }


  public
double  spouseAge;

  /**
   * Returns default value for parameter <code>spouseAge</code>.
   * <i>This method should not be called by user</i>
   */
  @AnyLogicInternalCodegenAPI
  public double _spouseAge_DefaultValue_xjal() {
    final Main self = this;
    return
(double) selectFrom(parameters)
      .where(parameters.parameter.eq("spouseage"))
      .uniqueResult(parameters.starting_value)
;
  }

  public void set_spouseAge( double spouseAge ) {
    if (spouseAge == this.spouseAge) {
      return;
    }
    double _oldValue_xjal = this.spouseAge;
    this.spouseAge = spouseAge;
    onChange_spouseAge_xjal( _oldValue_xjal );
    onChange();
  }

  /**
   * Calls "On change" action for parameter spouseAge.<br>
   * Note that 'oldValue' in that action will be unavailable if this method is called
by user
   * (current parameter value will be passed as 'oldValue').<br>
   * Please call <code>set_spouseAge()</code> method instead.
   */
  protected void onChange_spouseAge() {
    onChange_spouseAge_xjal( spouseAge );
  }

  @AnyLogicInternalCodegenAPI
  protected void onChange_spouseAge_xjal( double oldValue ) {
  }


  public
double  age;
```

```
  /**
   * Returns default value for parameter <code>age</code>.
   * <i>This method should not be called by user</i>
   */
  @AnyLogicInternalCodegenAPI
  public double _age_DefaultValue_xjal() {
    final Main self = this;
    return
(double) selectFrom(parameters)
      .where(parameters.parameter.eq("age"))
      .uniqueResult(parameters.starting_value)
;
  }

  public void set_age( double age ) {
    if (age == this.age) {
      return;
    }
    double _oldValue_xjal = this.age;
    this.age = age;
    onChange_age_xjal( _oldValue_xjal );
    onChange();
  }

  /**
   * Calls "On change" action for parameter age.<br>
   * Note that 'oldValue' in that action will be unavailable if this method is called
by user
   * (current parameter value will be passed as 'oldValue').<br>
   * Please call <code>set_age()</code> method instead.
   */
  protected void onChange_age() {
    onChange_age_xjal( age );
  }

  @AnyLogicInternalCodegenAPI
  protected void onChange_age_xjal( double oldValue ) {
  }


  public
double  csPlanned;

  /**
   * Returns default value for parameter <code>csPlanned</code>.
   * <i>This method should not be called by user</i>
   */
  @AnyLogicInternalCodegenAPI
  public double _csPlanned_DefaultValue_xjal() {
    final Main self = this;
    return
(double) selectFrom(parameters)
      .where(parameters.parameter.eq("csplanned"))
      .uniqueResult(parameters.starting_value)
;
  }

  public void set_csPlanned( double csPlanned ) {
    if (csPlanned == this.csPlanned) {
      return;
    }
    double _oldValue_xjal = this.csPlanned;
```

```
    this.csPlanned = csPlanned;
    onChange_csPlanned_xjal( _oldValue_xjal );
    onChange();
  }

  /**
   * Calls "On change" action for parameter csPlanned.<br>
   * Note that 'oldValue' in that action will be unavailable if this method is called
by user
   * (current parameter value will be passed as 'oldValue').<br>
   * Please call <code>set_csPlanned()</code> method instead.
   */
  protected void onChange_csPlanned() {
    onChange_csPlanned_xjal( csPlanned );
  }

  @AnyLogicInternalCodegenAPI
  protected void onChange_csPlanned_xjal( double oldValue ) {
  }


  public
double  anc4SE;

  /**
   * Returns default value for parameter <code>anc4SE</code>.
   * <i>This method should not be called by user</i>
   */
  @AnyLogicInternalCodegenAPI
  public double _anc4SE_DefaultValue_xjal() {
    final Main self = this;
    return
(double) selectFrom(parameters)
      .where(parameters.parameter.eq("anc4"))
      .uniqueResult(parameters.se)
;
  }

  public void set_anc4SE( double anc4SE ) {
    if (anc4SE == this.anc4SE) {
      return;
    }
    double _oldValue_xjal = this.anc4SE;
    this.anc4SE = anc4SE;
    onChange_anc4SE_xjal( _oldValue_xjal );
    onChange();
  }

  /**
   * Calls "On change" action for parameter anc4SE.<br>
   * Note that 'oldValue' in that action will be unavailable if this method is called
by user
   * (current parameter value will be passed as 'oldValue').<br>
   * Please call <code>set_anc4SE()</code> method instead.
   */
  protected void onChange_anc4SE() {
    onChange_anc4SE_xjal( anc4SE );
  }

  @AnyLogicInternalCodegenAPI
  protected void onChange_anc4SE_xjal( double oldValue ) {
  }
```

```
  public
double  unwantedSE;

  /**
   * Returns default value for parameter <code>unwantedSE</code>.
   * <i>This method should not be called by user</i>
   */
  @AnyLogicInternalCodegenAPI
  public double _unwantedSE_DefaultValue_xjal() {
    final Main self = this;
    return
(double) selectFrom(parameters)
       .where(parameters.parameter.eq("unwanted"))
       .uniqueResult(parameters.se)
;
  }

  public void set_unwantedSE( double unwantedSE ) {
    if (unwantedSE == this.unwantedSE) {
      return;
    }
    double _oldValue_xjal = this.unwantedSE;
    this.unwantedSE = unwantedSE;
    onChange_unwantedSE_xjal( _oldValue_xjal );
    onChange();
  }

  /**
   * Calls "On change" action for parameter unwantedSE.<br>
   * Note that 'oldValue' in that action will be unavailable if this method is called
by user
   * (current parameter value will be passed as 'oldValue').<br>
   * Please call <code>set_unwantedSE()</code> method instead.
   */
  protected void onChange_unwantedSE() {
    onChange_unwantedSE_xjal( unwantedSE );
  }

  @AnyLogicInternalCodegenAPI
  protected void onChange_unwantedSE_xjal( double oldValue ) {
  }


  public
double  riskSE;

  /**
   * Returns default value for parameter <code>riskSE</code>.
   * <i>This method should not be called by user</i>
   */
  @AnyLogicInternalCodegenAPI
  public double _riskSE_DefaultValue_xjal() {
    final Main self = this;
    return
(double) selectFrom(parameters)
       .where(parameters.parameter.eq("risk"))
       .uniqueResult(parameters.se)
;
  }

  public void set_riskSE( double riskSE ) {
    if (riskSE == this.riskSE) {
```

```
      return;
    }
    double _oldValue_xjal = this.riskSE;
    this.riskSE = riskSE;
    onChange_riskSE_xjal( _oldValue_xjal );
    onChange();
  }

  /**
   * Calls "On change" action for parameter riskSE.<br>
   * Note that 'oldValue' in that action will be unavailable if this method is called
by user
   * (current parameter value will be passed as 'oldValue').<br>
   * Please call <code>set_riskSE()</code> method instead.
   */
  protected void onChange_riskSE() {
    onChange_riskSE_xjal( riskSE );
  }

  @AnyLogicInternalCodegenAPI
  protected void onChange_riskSE_xjal( double oldValue ) {
  }


  public
double  urbanSE;

  /**
   * Returns default value for parameter <code>urbanSE</code>.
   * <i>This method should not be called by user</i>
   */
  @AnyLogicInternalCodegenAPI
  public double _urbanSE_DefaultValue_xjal() {
    final Main self = this;
    return
(double) selectFrom(parameters)
       .where(parameters.parameter.eq("urban"))
       .uniqueResult(parameters.se)
;
  }

  public void set_urbanSE( double urbanSE ) {
    if (urbanSE == this.urbanSE) {
      return;
    }
    double _oldValue_xjal = this.urbanSE;
    this.urbanSE = urbanSE;
    onChange_urbanSE_xjal( _oldValue_xjal );
    onChange();
  }

  /**
   * Calls "On change" action for parameter urbanSE.<br>
   * Note that 'oldValue' in that action will be unavailable if this method is called
by user
   * (current parameter value will be passed as 'oldValue').<br>
   * Please call <code>set_urbanSE()</code> method instead.
   */
  protected void onChange_urbanSE() {
    onChange_urbanSE_xjal( urbanSE );
  }

  @AnyLogicInternalCodegenAPI
```

```
  protected void onChange_urbanSE_xjal( double oldValue ) {
  }


  public
double  twinsSE;

  /**
   * Returns default value for parameter <code>twinsSE</code>.
   * <i>This method should not be called by user</i>
   */
  @AnyLogicInternalCodegenAPI
  public double _twinsSE_DefaultValue_xjal() {
    final Main self = this;
    return
(double) selectFrom(parameters)
       .where(parameters.parameter.eq("twins"))
       .uniqueResult(parameters.se)
;
  }

  public void set_twinsSE( double twinsSE ) {
    if (twinsSE == this.twinsSE) {
      return;
    }
    double _oldValue_xjal = this.twinsSE;
    this.twinsSE = twinsSE;
    onChange_twinsSE_xjal( _oldValue_xjal );
    onChange();
  }

  /**
   * Calls "On change" action for parameter twinsSE.<br>
   * Note that 'oldValue' in that action will be unavailable if this method is called
by user
   * (current parameter value will be passed as 'oldValue').<br>
   * Please call <code>set_twinsSE()</code> method instead.
   */
  protected void onChange_twinsSE() {
    onChange_twinsSE_xjal( twinsSE );
  }

  @AnyLogicInternalCodegenAPI
  protected void onChange_twinsSE_xjal( double oldValue ) {
  }


  public
double  educSecSE;

  /**
   * Returns default value for parameter <code>educSecSE</code>.
   * <i>This method should not be called by user</i>
   */
  @AnyLogicInternalCodegenAPI
  public double _educSecSE_DefaultValue_xjal() {
    final Main self = this;
    return
(double) selectFrom(parameters)
       .where(parameters.parameter.eq("educsec"))
       .uniqueResult(parameters.se)
;
  }
```

```
  public void set_educSecSE( double educSecSE ) {
    if (educSecSE == this.educSecSE) {
      return;
    }
    double _oldValue_xjal = this.educSecSE;
    this.educSecSE = educSecSE;
    onChange_educSecSE_xjal( _oldValue_xjal );
    onChange();
  }

  /**
   * Calls "On change" action for parameter educSecSE.<br>
   * Note that 'oldValue' in that action will be unavailable if this method is called
by user
   * (current parameter value will be passed as 'oldValue').<br>
   * Please call <code>set_educSecSE()</code> method instead.
   */
  protected void onChange_educSecSE() {
    onChange_educSecSE_xjal( educSecSE );
  }

  @AnyLogicInternalCodegenAPI
  protected void onChange_educSecSE_xjal( double oldValue ) {
  }


  public
double  spouseAgeSE;

  /**
   * Returns default value for parameter <code>spouseAgeSE</code>.
   * <i>This method should not be called by user</i>
   */
  @AnyLogicInternalCodegenAPI
  public double _spouseAgeSE_DefaultValue_xjal() {
    final Main self = this;
    return
(double) selectFrom(parameters)
      .where(parameters.parameter.eq("spouseage"))
      .uniqueResult(parameters.se)
;
  }

  public void set_spouseAgeSE( double spouseAgeSE ) {
    if (spouseAgeSE == this.spouseAgeSE) {
      return;
    }
    double _oldValue_xjal = this.spouseAgeSE;
    this.spouseAgeSE = spouseAgeSE;
    onChange_spouseAgeSE_xjal( _oldValue_xjal );
    onChange();
  }

  /**
   * Calls "On change" action for parameter spouseAgeSE.<br>
   * Note that 'oldValue' in that action will be unavailable if this method is called
by user
   * (current parameter value will be passed as 'oldValue').<br>
   * Please call <code>set_spouseAgeSE()</code> method instead.
   */
  protected void onChange_spouseAgeSE() {
    onChange_spouseAgeSE_xjal( spouseAgeSE );
```

```
  }

  @AnyLogicInternalCodegenAPI
  protected void onChange_spouseAgeSE_xjal( double oldValue ) {
  }


  public
double  ageSE;

  /**
   * Returns default value for parameter <code>ageSE</code>.
   * <i>This method should not be called by user</i>
   */
  @AnyLogicInternalCodegenAPI
  public double _ageSE_DefaultValue_xjal() {
    final Main self = this;
    return
(double) selectFrom(parameters)
       .where(parameters.parameter.eq("age"))
       .uniqueResult(parameters.se)
;
  }

  public void set_ageSE( double ageSE ) {
    if (ageSE == this.ageSE) {
      return;
    }
    double _oldValue_xjal = this.ageSE;
    this.ageSE = ageSE;
    onChange_ageSE_xjal( _oldValue_xjal );
    onChange();
  }

  /**
   * Calls "On change" action for parameter ageSE.<br>
   * Note that 'oldValue' in that action will be unavailable if this method is called
by user
   * (current parameter value will be passed as 'oldValue').<br>
   * Please call <code>set_ageSE()</code> method instead.
   */
  protected void onChange_ageSE() {
    onChange_ageSE_xjal( ageSE );
  }

  @AnyLogicInternalCodegenAPI
  protected void onChange_ageSE_xjal( double oldValue ) {
  }


  public
double  csPlannedSE;

  /**
   * Returns default value for parameter <code>csPlannedSE</code>.
   * <i>This method should not be called by user</i>
   */
  @AnyLogicInternalCodegenAPI
  public double _csPlannedSE_DefaultValue_xjal() {
    final Main self = this;
    return
(double) selectFrom(parameters)
       .where(parameters.parameter.eq("csplanned"))
```

```
      .uniqueResult(parameters.se)
;
  }

  public void set_csPlannedSE( double csPlannedSE ) {
    if (csPlannedSE == this.csPlannedSE) {
      return;
    }
    double _oldValue_xjal = this.csPlannedSE;
    this.csPlannedSE = csPlannedSE;
    onChange_csPlannedSE_xjal( _oldValue_xjal );
    onChange();
  }

  /**
   * Calls "On change" action for parameter csPlannedSE.<br>
   * Note that 'oldValue' in that action will be unavailable if this method is called
by user
   * (current parameter value will be passed as 'oldValue').<br>
   * Please call <code>set_csPlannedSE()</code> method instead.
   */
  protected void onChange_csPlannedSE() {
    onChange_csPlannedSE_xjal( csPlannedSE );
  }

  @AnyLogicInternalCodegenAPI
  protected void onChange_csPlannedSE_xjal( double oldValue ) {
  }


  /**
   * Addition to the hospital's base quality level in the optimization experiment
   */
  public
double  QIParam;

  /**
   * Returns default value for parameter <code>QIParam</code>.
   * <i>This method should not be called by user</i>
   */
  @AnyLogicInternalCodegenAPI
  public double _QIParam_DefaultValue_xjal() {
    final Main self = this;
    return
0.0
;
  }

  public void set_QIParam( double QIParam ) {
    if (QIParam == this.QIParam) {
      return;
    }
    double _oldValue_xjal = this.QIParam;
    this.QIParam = QIParam;
    onChange_QIParam_xjal( _oldValue_xjal );
    onChange();
  }

  /**
   * Calls "On change" action for parameter QIParam.<br>
   * Note that 'oldValue' in that action will be unavailable if this method is called
by user
   * (current parameter value will be passed as 'oldValue').<br>
```

```
   * Please call <code>set_QIParam()</code> method instead.
   */
  protected void onChange_QIParam() {
    onChange_QIParam_xjal( QIParam );
  }

  @AnyLogicInternalCodegenAPI
  protected void onChange_QIParam_xjal( double oldValue ) {
  }


  public
int  strategy;

  /**
   * Returns default value for parameter <code>strategy</code>.
   * <i>This method should not be called by user</i>
   */
  @AnyLogicInternalCodegenAPI
  public int _strategy_DefaultValue_xjal() {
    final Main self = this;
    return 0;
  }

  public void set_strategy( int strategy ) {
    if (strategy == this.strategy) {
      return;
    }
    int _oldValue_xjal = this.strategy;
    this.strategy = strategy;
    onChange_strategy_xjal( _oldValue_xjal );
    onChange();
  }

  /**
   * Calls "On change" action for parameter strategy.<br>
   * Note that 'oldValue' in that action will be unavailable if this method is called
by user
   * (current parameter value will be passed as 'oldValue').<br>
   * Please call <code>set_strategy()</code> method instead.
   */
  protected void onChange_strategy() {
    onChange_strategy_xjal( strategy );
  }

  @AnyLogicInternalCodegenAPI
  protected void onChange_strategy_xjal( int oldValue ) {
  }


  @Override
  public void setParametersToDefaultValues() {
    super.setParametersToDefaultValues();
    allowFeesParam = _allowFeesParam_DefaultValue_xjal();
    centralHosp1 = _centralHosp1_DefaultValue_xjal();
    distance1 = _distance1_DefaultValue_xjal();
    clinic1 = _clinic1_DefaultValue_xjal();
    maternity1 = _maternity1_DefaultValue_xjal();
    healthCenter1 = _healthCenter1_DefaultValue_xjal();
    otherHosp1 = _otherHosp1_DefaultValue_xjal();
    communityHosp1 = _communityHosp1_DefaultValue_xjal();
    districtHosp1 = _districtHosp1_DefaultValue_xjal();
    bobs1 = _bobs1_DefaultValue_xjal();
```

```
fees1 = _fees1_DefaultValue_xjal();
allowAllParam = _allowAllParam_DefaultValue_xjal();
intercept = _intercept_DefaultValue_xjal();
centralHosp2 = _centralHosp2_DefaultValue_xjal();
distance2 = _distance2_DefaultValue_xjal();
clinic2 = _clinic2_DefaultValue_xjal();
maternity2 = _maternity2_DefaultValue_xjal();
healthCenter2 = _healthCenter2_DefaultValue_xjal();
otherHosp2 = _otherHosp2_DefaultValue_xjal();
communityHosp2 = _communityHosp2_DefaultValue_xjal();
districtHosp2 = _districtHosp2_DefaultValue_xjal();
bobs2 = _bobs2_DefaultValue_xjal();
fees2 = _fees2_DefaultValue_xjal();
distCenter = _distCenter_DefaultValue_xjal();
distRMS = _distRMS_DefaultValue_xjal();
centralHosp1SE = _centralHosp1SE_DefaultValue_xjal();
distance1SE = _distance1SE_DefaultValue_xjal();
clinic1SE = _clinic1SE_DefaultValue_xjal();
maternity1SE = _maternity1SE_DefaultValue_xjal();
healthCenter1SE = _healthCenter1SE_DefaultValue_xjal();
otherHosp1SE = _otherHosp1SE_DefaultValue_xjal();
communityHosp1SE = _communityHosp1SE_DefaultValue_xjal();
districtHosp1SE = _districtHosp1SE_DefaultValue_xjal();
bobs1SE = _bobs1SE_DefaultValue_xjal();
fees1SE = _fees1SE_DefaultValue_xjal();
interceptSE = _interceptSE_DefaultValue_xjal();
centralHosp2SE = _centralHosp2SE_DefaultValue_xjal();
distance2SE = _distance2SE_DefaultValue_xjal();
clinic2SE = _clinic2SE_DefaultValue_xjal();
maternity2SE = _maternity2SE_DefaultValue_xjal();
healthCenter2SE = _healthCenter2SE_DefaultValue_xjal();
otherHosp2SE = _otherHosp2SE_DefaultValue_xjal();
communityHosp2SE = _communityHosp2SE_DefaultValue_xjal();
districtHosp2SE = _districtHosp2SE_DefaultValue_xjal();
bobs2SE = _bobs2SE_DefaultValue_xjal();
fees2SE = _fees2SE_DefaultValue_xjal();
centralCS = _centralCS_DefaultValue_xjal();
clinicCS = _clinicCS_DefaultValue_xjal();
maternityCS = _maternityCS_DefaultValue_xjal();
healthCenterCS = _healthCenterCS_DefaultValue_xjal();
otherCS = _otherCS_DefaultValue_xjal();
communityCS = _communityCS_DefaultValue_xjal();
districtCS = _districtCS_DefaultValue_xjal();
centralCSSE = _centralCSSE_DefaultValue_xjal();
clinicCSSE = _clinicCSSE_DefaultValue_xjal();
maternityCSSE = _maternityCSSE_DefaultValue_xjal();
healthCenterCSSE = _healthCenterCSSE_DefaultValue_xjal();
otherCSSE = _otherCSSE_DefaultValue_xjal();
communityCSSE = _communityCSSE_DefaultValue_xjal();
districtCSSE = _districtCSSE_DefaultValue_xjal();
centralNo = _centralNo_DefaultValue_xjal();
clinicNo = _clinicNo_DefaultValue_xjal();
maternityNo = _maternityNo_DefaultValue_xjal();
healthCenterNo = _healthCenterNo_DefaultValue_xjal();
otherNo = _otherNo_DefaultValue_xjal();
communityNo = _communityNo_DefaultValue_xjal();
districtNo = _districtNo_DefaultValue_xjal();
centralNoSE = _centralNoSE_DefaultValue_xjal();
clinicNoSE = _clinicNoSE_DefaultValue_xjal();
maternityNoSE = _maternityNoSE_DefaultValue_xjal();
healthCenterNoSE = _healthCenterNoSE_DefaultValue_xjal();
otherNoSE = _otherNoSE_DefaultValue_xjal();
communityNoSE = _communityNoSE_DefaultValue_xjal();
```

```java
    districtNoSE = _districtNoSE_DefaultValue_xjal();
    central5 = _central5_DefaultValue_xjal();
    clinic5 = _clinic5_DefaultValue_xjal();
    maternity5 = _maternity5_DefaultValue_xjal();
    healthCenter5 = _healthCenter5_DefaultValue_xjal();
    other5 = _other5_DefaultValue_xjal();
    community5 = _community5_DefaultValue_xjal();
    district5 = _district5_DefaultValue_xjal();
    central5SE = _central5SE_DefaultValue_xjal();
    clinic5SE = _clinic5SE_DefaultValue_xjal();
    maternity5SE = _maternity5SE_DefaultValue_xjal();
    healthCenter5SE = _healthCenter5SE_DefaultValue_xjal();
    other5SE = _other5SE_DefaultValue_xjal();
    community5SE = _community5SE_DefaultValue_xjal();
    district5SE = _district5SE_DefaultValue_xjal();
    centralNo5 = _centralNo5_DefaultValue_xjal();
    clinicNo5 = _clinicNo5_DefaultValue_xjal();
    maternityNo5 = _maternityNo5_DefaultValue_xjal();
    healthCenterNo5 = _healthCenterNo5_DefaultValue_xjal();
    otherNo5 = _otherNo5_DefaultValue_xjal();
    communityNo5 = _communityNo5_DefaultValue_xjal();
    districtNo5 = _districtNo5_DefaultValue_xjal();
    centralNo5SE = _centralNo5SE_DefaultValue_xjal();
    clinicNo5SE = _clinicNo5SE_DefaultValue_xjal();
    maternityNo5SE = _maternityNo5SE_DefaultValue_xjal();
    healthCenterNo5SE = _healthCenterNo5SE_DefaultValue_xjal();
    otherNo5SE = _otherNo5SE_DefaultValue_xjal();
    communityNo5SE = _communityNo5SE_DefaultValue_xjal();
    districtNo5SE = _districtNo5SE_DefaultValue_xjal();
    anc4 = _anc4_DefaultValue_xjal();
    unwanted = _unwanted_DefaultValue_xjal();
    risk = _risk_DefaultValue_xjal();
    urban = _urban_DefaultValue_xjal();
    twins = _twins_DefaultValue_xjal();
    educSec = _educSec_DefaultValue_xjal();
    spouseAge = _spouseAge_DefaultValue_xjal();
    age = _age_DefaultValue_xjal();
    csPlanned = _csPlanned_DefaultValue_xjal();
    anc4SE = _anc4SE_DefaultValue_xjal();
    unwantedSE = _unwantedSE_DefaultValue_xjal();
    riskSE = _riskSE_DefaultValue_xjal();
    urbanSE = _urbanSE_DefaultValue_xjal();
    twinsSE = _twinsSE_DefaultValue_xjal();
    educSecSE = _educSecSE_DefaultValue_xjal();
    spouseAgeSE = _spouseAgeSE_DefaultValue_xjal();
    ageSE = _ageSE_DefaultValue_xjal();
    csPlannedSE = _csPlannedSE_DefaultValue_xjal();
    QIParam = _QIParam_DefaultValue_xjal();
    strategy = _strategy_DefaultValue_xjal();
  }

  @Override
  public boolean setParameter(String _name_xjal, Object _value_xjal, boolean
_callOnChange_xjal) {
    switch ( _name_xjal ) {
    case "allowFeesParam":
      if ( _callOnChange_xjal ) {
        set_allowFeesParam( (Boolean) _value_xjal );
      } else {
        allowFeesParam = (Boolean) _value_xjal;
      }
      return true;
    case "centralHosp1":
```

```
  if ( _callOnChange_xjal ) {
    set_centralHosp1( ((Number) _value_xjal).doubleValue() );
  } else {
    centralHosp1 = ((Number) _value_xjal).doubleValue();
  }
  return true;
case "distance1":
  if ( _callOnChange_xjal ) {
    set_distance1( ((Number) _value_xjal).doubleValue() );
  } else {
    distance1 = ((Number) _value_xjal).doubleValue();
  }
  return true;
case "clinic1":
  if ( _callOnChange_xjal ) {
    set_clinic1( ((Number) _value_xjal).doubleValue() );
  } else {
    clinic1 = ((Number) _value_xjal).doubleValue();
  }
  return true;
case "maternity1":
  if ( _callOnChange_xjal ) {
    set_maternity1( ((Number) _value_xjal).doubleValue() );
  } else {
    maternity1 = ((Number) _value_xjal).doubleValue();
  }
  return true;
case "healthCenter1":
  if ( _callOnChange_xjal ) {
    set_healthCenter1( ((Number) _value_xjal).doubleValue() );
  } else {
    healthCenter1 = ((Number) _value_xjal).doubleValue();
  }
  return true;
case "otherHosp1":
  if ( _callOnChange_xjal ) {
    set_otherHosp1( ((Number) _value_xjal).doubleValue() );
  } else {
    otherHosp1 = ((Number) _value_xjal).doubleValue();
  }
  return true;
case "communityHosp1":
  if ( _callOnChange_xjal ) {
    set_communityHosp1( ((Number) _value_xjal).doubleValue() );
  } else {
    communityHosp1 = ((Number) _value_xjal).doubleValue();
  }
  return true;
case "districtHosp1":
  if ( _callOnChange_xjal ) {
    set_districtHosp1( ((Number) _value_xjal).doubleValue() );
  } else {
    districtHosp1 = ((Number) _value_xjal).doubleValue();
  }
  return true;
case "bobs1":
  if ( _callOnChange_xjal ) {
    set_bobs1( ((Number) _value_xjal).doubleValue() );
  } else {
    bobs1 = ((Number) _value_xjal).doubleValue();
  }
  return true;
case "fees1":
```

```
  if ( _callOnChange_xjal ) {
    set_fees1( ((Number) _value_xjal).doubleValue() );
  } else {
    fees1 = ((Number) _value_xjal).doubleValue();
  }
  return true;
case "allowAllParam":
  if ( _callOnChange_xjal ) {
    set_allowAllParam( (Boolean) _value_xjal );
  } else {
    allowAllParam = (Boolean) _value_xjal;
  }
  return true;
case "intercept":
  if ( _callOnChange_xjal ) {
    set_intercept( ((Number) _value_xjal).doubleValue() );
  } else {
    intercept = ((Number) _value_xjal).doubleValue();
  }
  return true;
case "centralHosp2":
  if ( _callOnChange_xjal ) {
    set_centralHosp2( ((Number) _value_xjal).doubleValue() );
  } else {
    centralHosp2 = ((Number) _value_xjal).doubleValue();
  }
  return true;
case "distance2":
  if ( _callOnChange_xjal ) {
    set_distance2( ((Number) _value_xjal).doubleValue() );
  } else {
    distance2 = ((Number) _value_xjal).doubleValue();
  }
  return true;
case "clinic2":
  if ( _callOnChange_xjal ) {
    set_clinic2( ((Number) _value_xjal).doubleValue() );
  } else {
    clinic2 = ((Number) _value_xjal).doubleValue();
  }
  return true;
case "maternity2":
  if ( _callOnChange_xjal ) {
    set_maternity2( ((Number) _value_xjal).doubleValue() );
  } else {
    maternity2 = ((Number) _value_xjal).doubleValue();
  }
  return true;
case "healthCenter2":
  if ( _callOnChange_xjal ) {
    set_healthCenter2( ((Number) _value_xjal).doubleValue() );
  } else {
    healthCenter2 = ((Number) _value_xjal).doubleValue();
  }
  return true;
case "otherHosp2":
  if ( _callOnChange_xjal ) {
    set_otherHosp2( ((Number) _value_xjal).doubleValue() );
  } else {
    otherHosp2 = ((Number) _value_xjal).doubleValue();
  }
  return true;
case "communityHosp2":
```

```
  if ( _callOnChange_xjal ) {
    set_communityHosp2( ((Number) _value_xjal).doubleValue() );
  } else {
    communityHosp2 = ((Number) _value_xjal).doubleValue();
  }
  return true;
case "districtHosp2":
  if ( _callOnChange_xjal ) {
    set_districtHosp2( ((Number) _value_xjal).doubleValue() );
  } else {
    districtHosp2 = ((Number) _value_xjal).doubleValue();
  }
  return true;
case "bobs2":
  if ( _callOnChange_xjal ) {
    set_bobs2( ((Number) _value_xjal).doubleValue() );
  } else {
    bobs2 = ((Number) _value_xjal).doubleValue();
  }
  return true;
case "fees2":
  if ( _callOnChange_xjal ) {
    set_fees2( ((Number) _value_xjal).doubleValue() );
  } else {
    fees2 = ((Number) _value_xjal).doubleValue();
  }
  return true;
case "distCenter":
  if ( _callOnChange_xjal ) {
    set_distCenter( ((Number) _value_xjal).doubleValue() );
  } else {
    distCenter = ((Number) _value_xjal).doubleValue();
  }
  return true;
case "distRMS":
  if ( _callOnChange_xjal ) {
    set_distRMS( ((Number) _value_xjal).doubleValue() );
  } else {
    distRMS = ((Number) _value_xjal).doubleValue();
  }
  return true;
case "centralHosp1SE":
  if ( _callOnChange_xjal ) {
    set_centralHosp1SE( ((Number) _value_xjal).doubleValue() );
  } else {
    centralHosp1SE = ((Number) _value_xjal).doubleValue();
  }
  return true;
case "distance1SE":
  if ( _callOnChange_xjal ) {
    set_distance1SE( ((Number) _value_xjal).doubleValue() );
  } else {
    distance1SE = ((Number) _value_xjal).doubleValue();
  }
  return true;
case "clinic1SE":
  if ( _callOnChange_xjal ) {
    set_clinic1SE( ((Number) _value_xjal).doubleValue() );
  } else {
    clinic1SE = ((Number) _value_xjal).doubleValue();
  }
  return true;
case "maternity1SE":
```

```
  if ( _callOnChange_xjal ) {
    set_maternity1SE( ((Number) _value_xjal).doubleValue() );
  } else {
    maternity1SE = ((Number) _value_xjal).doubleValue();
  }
  return true;
case "healthCenter1SE":
  if ( _callOnChange_xjal ) {
    set_healthCenter1SE( ((Number) _value_xjal).doubleValue() );
  } else {
    healthCenter1SE = ((Number) _value_xjal).doubleValue();
  }
  return true;
case "otherHosp1SE":
  if ( _callOnChange_xjal ) {
    set_otherHosp1SE( ((Number) _value_xjal).doubleValue() );
  } else {
    otherHosp1SE = ((Number) _value_xjal).doubleValue();
  }
  return true;
case "communityHosp1SE":
  if ( _callOnChange_xjal ) {
    set_communityHosp1SE( ((Number) _value_xjal).doubleValue() );
  } else {
    communityHosp1SE = ((Number) _value_xjal).doubleValue();
  }
  return true;
case "districtHosp1SE":
  if ( _callOnChange_xjal ) {
    set_districtHosp1SE( ((Number) _value_xjal).doubleValue() );
  } else {
    districtHosp1SE = ((Number) _value_xjal).doubleValue();
  }
  return true;
case "bobs1SE":
  if ( _callOnChange_xjal ) {
    set_bobs1SE( ((Number) _value_xjal).doubleValue() );
  } else {
    bobs1SE = ((Number) _value_xjal).doubleValue();
  }
  return true;
case "fees1SE":
  if ( _callOnChange_xjal ) {
    set_fees1SE( ((Number) _value_xjal).doubleValue() );
  } else {
    fees1SE = ((Number) _value_xjal).doubleValue();
  }
  return true;
case "interceptSE":
  if ( _callOnChange_xjal ) {
    set_interceptSE( ((Number) _value_xjal).doubleValue() );
  } else {
    interceptSE = ((Number) _value_xjal).doubleValue();
  }
  return true;
case "centralHosp2SE":
  if ( _callOnChange_xjal ) {
    set_centralHosp2SE( ((Number) _value_xjal).doubleValue() );
  } else {
    centralHosp2SE = ((Number) _value_xjal).doubleValue();
  }
  return true;
case "distance2SE":
```

```
  if ( _callOnChange_xjal ) {
    set_distance2SE( ((Number) _value_xjal).doubleValue() );
  } else {
    distance2SE = ((Number) _value_xjal).doubleValue();
  }
  return true;
case "clinic2SE":
  if ( _callOnChange_xjal ) {
    set_clinic2SE( ((Number) _value_xjal).doubleValue() );
  } else {
    clinic2SE = ((Number) _value_xjal).doubleValue();
  }
  return true;
case "maternity2SE":
  if ( _callOnChange_xjal ) {
    set_maternity2SE( ((Number) _value_xjal).doubleValue() );
  } else {
    maternity2SE = ((Number) _value_xjal).doubleValue();
  }
  return true;
case "healthCenter2SE":
  if ( _callOnChange_xjal ) {
    set_healthCenter2SE( ((Number) _value_xjal).doubleValue() );
  } else {
    healthCenter2SE = ((Number) _value_xjal).doubleValue();
  }
  return true;
case "otherHosp2SE":
  if ( _callOnChange_xjal ) {
    set_otherHosp2SE( ((Number) _value_xjal).doubleValue() );
  } else {
    otherHosp2SE = ((Number) _value_xjal).doubleValue();
  }
  return true;
case "communityHosp2SE":
  if ( _callOnChange_xjal ) {
    set_communityHosp2SE( ((Number) _value_xjal).doubleValue() );
  } else {
    communityHosp2SE = ((Number) _value_xjal).doubleValue();
  }
  return true;
case "districtHosp2SE":
  if ( _callOnChange_xjal ) {
    set_districtHosp2SE( ((Number) _value_xjal).doubleValue() );
  } else {
    districtHosp2SE = ((Number) _value_xjal).doubleValue();
  }
  return true;
case "bobs2SE":
  if ( _callOnChange_xjal ) {
    set_bobs2SE( ((Number) _value_xjal).doubleValue() );
  } else {
    bobs2SE = ((Number) _value_xjal).doubleValue();
  }
  return true;
case "fees2SE":
  if ( _callOnChange_xjal ) {
    set_fees2SE( ((Number) _value_xjal).doubleValue() );
  } else {
    fees2SE = ((Number) _value_xjal).doubleValue();
  }
  return true;
case "centralCS":
```

```
  if ( _callOnChange_xjal ) {
    set_centralCS( ((Number) _value_xjal).doubleValue() );
  } else {
    centralCS = ((Number) _value_xjal).doubleValue();
  }
  return true;
case "clinicCS":
  if ( _callOnChange_xjal ) {
    set_clinicCS( ((Number) _value_xjal).doubleValue() );
  } else {
    clinicCS = ((Number) _value_xjal).doubleValue();
  }
  return true;
case "maternityCS":
  if ( _callOnChange_xjal ) {
    set_maternityCS( ((Number) _value_xjal).doubleValue() );
  } else {
    maternityCS = ((Number) _value_xjal).doubleValue();
  }
  return true;
case "healthCenterCS":
  if ( _callOnChange_xjal ) {
    set_healthCenterCS( ((Number) _value_xjal).doubleValue() );
  } else {
    healthCenterCS = ((Number) _value_xjal).doubleValue();
  }
  return true;
case "otherCS":
  if ( _callOnChange_xjal ) {
    set_otherCS( ((Number) _value_xjal).doubleValue() );
  } else {
    otherCS = ((Number) _value_xjal).doubleValue();
  }
  return true;
case "communityCS":
  if ( _callOnChange_xjal ) {
    set_communityCS( ((Number) _value_xjal).doubleValue() );
  } else {
    communityCS = ((Number) _value_xjal).doubleValue();
  }
  return true;
case "districtCS":
  if ( _callOnChange_xjal ) {
    set_districtCS( ((Number) _value_xjal).doubleValue() );
  } else {
    districtCS = ((Number) _value_xjal).doubleValue();
  }
  return true;
case "centralCSSE":
  if ( _callOnChange_xjal ) {
    set_centralCSSE( ((Number) _value_xjal).doubleValue() );
  } else {
    centralCSSE = ((Number) _value_xjal).doubleValue();
  }
  return true;
case "clinicCSSE":
  if ( _callOnChange_xjal ) {
    set_clinicCSSE( ((Number) _value_xjal).doubleValue() );
  } else {
    clinicCSSE = ((Number) _value_xjal).doubleValue();
  }
  return true;
case "maternityCSSE":
```

```
  if ( _callOnChange_xjal ) {
    set_maternityCSSE( ((Number) _value_xjal).doubleValue() );
  } else {
    maternityCSSE = ((Number) _value_xjal).doubleValue();
  }
  return true;
case "healthCenterCSSE":
  if ( _callOnChange_xjal ) {
    set_healthCenterCSSE( ((Number) _value_xjal).doubleValue() );
  } else {
    healthCenterCSSE = ((Number) _value_xjal).doubleValue();
  }
  return true;
case "otherCSSE":
  if ( _callOnChange_xjal ) {
    set_otherCSSE( ((Number) _value_xjal).doubleValue() );
  } else {
    otherCSSE = ((Number) _value_xjal).doubleValue();
  }
  return true;
case "communityCSSE":
  if ( _callOnChange_xjal ) {
    set_communityCSSE( ((Number) _value_xjal).doubleValue() );
  } else {
    communityCSSE = ((Number) _value_xjal).doubleValue();
  }
  return true;
case "districtCSSE":
  if ( _callOnChange_xjal ) {
    set_districtCSSE( ((Number) _value_xjal).doubleValue() );
  } else {
    districtCSSE = ((Number) _value_xjal).doubleValue();
  }
  return true;
case "centralNo":
  if ( _callOnChange_xjal ) {
    set_centralNo( ((Number) _value_xjal).doubleValue() );
  } else {
    centralNo = ((Number) _value_xjal).doubleValue();
  }
  return true;
case "clinicNo":
  if ( _callOnChange_xjal ) {
    set_clinicNo( ((Number) _value_xjal).doubleValue() );
  } else {
    clinicNo = ((Number) _value_xjal).doubleValue();
  }
  return true;
case "maternityNo":
  if ( _callOnChange_xjal ) {
    set_maternityNo( ((Number) _value_xjal).doubleValue() );
  } else {
    maternityNo = ((Number) _value_xjal).doubleValue();
  }
  return true;
case "healthCenterNo":
  if ( _callOnChange_xjal ) {
    set_healthCenterNo( ((Number) _value_xjal).doubleValue() );
  } else {
    healthCenterNo = ((Number) _value_xjal).doubleValue();
  }
  return true;
case "otherNo":
```

```
    if ( _callOnChange_xjal ) {
      set_otherNo( ((Number) _value_xjal).doubleValue() );
    } else {
      otherNo = ((Number) _value_xjal).doubleValue();
    }
    return true;
  case "communityNo":
    if ( _callOnChange_xjal ) {
      set_communityNo( ((Number) _value_xjal).doubleValue() );
    } else {
      communityNo = ((Number) _value_xjal).doubleValue();
    }
    return true;
  case "districtNo":
    if ( _callOnChange_xjal ) {
      set_districtNo( ((Number) _value_xjal).doubleValue() );
    } else {
      districtNo = ((Number) _value_xjal).doubleValue();
    }
    return true;
  case "centralNoSE":
    if ( _callOnChange_xjal ) {
      set_centralNoSE( ((Number) _value_xjal).doubleValue() );
    } else {
      centralNoSE = ((Number) _value_xjal).doubleValue();
    }
    return true;
  case "clinicNoSE":
    if ( _callOnChange_xjal ) {
      set_clinicNoSE( ((Number) _value_xjal).doubleValue() );
    } else {
      clinicNoSE = ((Number) _value_xjal).doubleValue();
    }
    return true;
  case "maternityNoSE":
    if ( _callOnChange_xjal ) {
      set_maternityNoSE( ((Number) _value_xjal).doubleValue() );
    } else {
      maternityNoSE = ((Number) _value_xjal).doubleValue();
    }
    return true;
  case "healthCenterNoSE":
    if ( _callOnChange_xjal ) {
      set_healthCenterNoSE( ((Number) _value_xjal).doubleValue() );
    } else {
      healthCenterNoSE = ((Number) _value_xjal).doubleValue();
    }
    return true;
  case "otherNoSE":
    if ( _callOnChange_xjal ) {
      set_otherNoSE( ((Number) _value_xjal).doubleValue() );
    } else {
      otherNoSE = ((Number) _value_xjal).doubleValue();
    }
    return true;
  case "communityNoSE":
    if ( _callOnChange_xjal ) {
      set_communityNoSE( ((Number) _value_xjal).doubleValue() );
    } else {
      communityNoSE = ((Number) _value_xjal).doubleValue();
    }
    return true;
  case "districtNoSE":
```

```
  if ( _callOnChange_xjal ) {
    set_districtNoSE( ((Number) _value_xjal).doubleValue() );
  } else {
    districtNoSE = ((Number) _value_xjal).doubleValue();
  }
  return true;
case "central5":
  if ( _callOnChange_xjal ) {
    set_central5( ((Number) _value_xjal).doubleValue() );
  } else {
    central5 = ((Number) _value_xjal).doubleValue();
  }
  return true;
case "clinic5":
  if ( _callOnChange_xjal ) {
    set_clinic5( ((Number) _value_xjal).doubleValue() );
  } else {
    clinic5 = ((Number) _value_xjal).doubleValue();
  }
  return true;
case "maternity5":
  if ( _callOnChange_xjal ) {
    set_maternity5( ((Number) _value_xjal).doubleValue() );
  } else {
    maternity5 = ((Number) _value_xjal).doubleValue();
  }
  return true;
case "healthCenter5":
  if ( _callOnChange_xjal ) {
    set_healthCenter5( ((Number) _value_xjal).doubleValue() );
  } else {
    healthCenter5 = ((Number) _value_xjal).doubleValue();
  }
  return true;
case "other5":
  if ( _callOnChange_xjal ) {
    set_other5( ((Number) _value_xjal).doubleValue() );
  } else {
    other5 = ((Number) _value_xjal).doubleValue();
  }
  return true;
case "community5":
  if ( _callOnChange_xjal ) {
    set_community5( ((Number) _value_xjal).doubleValue() );
  } else {
    community5 = ((Number) _value_xjal).doubleValue();
  }
  return true;
case "district5":
  if ( _callOnChange_xjal ) {
    set_district5( ((Number) _value_xjal).doubleValue() );
  } else {
    district5 = ((Number) _value_xjal).doubleValue();
  }
  return true;
case "central5SE":
  if ( _callOnChange_xjal ) {
    set_central5SE( ((Number) _value_xjal).doubleValue() );
  } else {
    central5SE = ((Number) _value_xjal).doubleValue();
  }
  return true;
case "clinic5SE":
```

```
  if ( _callOnChange_xjal ) {
    set_clinic5SE( ((Number) _value_xjal).doubleValue() );
  } else {
    clinic5SE = ((Number) _value_xjal).doubleValue();
  }
  return true;
case "maternity5SE":
  if ( _callOnChange_xjal ) {
    set_maternity5SE( ((Number) _value_xjal).doubleValue() );
  } else {
    maternity5SE = ((Number) _value_xjal).doubleValue();
  }
  return true;
case "healthCenter5SE":
  if ( _callOnChange_xjal ) {
    set_healthCenter5SE( ((Number) _value_xjal).doubleValue() );
  } else {
    healthCenter5SE = ((Number) _value_xjal).doubleValue();
  }
  return true;
case "other5SE":
  if ( _callOnChange_xjal ) {
    set_other5SE( ((Number) _value_xjal).doubleValue() );
  } else {
    other5SE = ((Number) _value_xjal).doubleValue();
  }
  return true;
case "community5SE":
  if ( _callOnChange_xjal ) {
    set_community5SE( ((Number) _value_xjal).doubleValue() );
  } else {
    community5SE = ((Number) _value_xjal).doubleValue();
  }
  return true;
case "district5SE":
  if ( _callOnChange_xjal ) {
    set_district5SE( ((Number) _value_xjal).doubleValue() );
  } else {
    district5SE = ((Number) _value_xjal).doubleValue();
  }
  return true;
case "centralNo5":
  if ( _callOnChange_xjal ) {
    set_centralNo5( ((Number) _value_xjal).doubleValue() );
  } else {
    centralNo5 = ((Number) _value_xjal).doubleValue();
  }
  return true;
case "clinicNo5":
  if ( _callOnChange_xjal ) {
    set_clinicNo5( ((Number) _value_xjal).doubleValue() );
  } else {
    clinicNo5 = ((Number) _value_xjal).doubleValue();
  }
  return true;
case "maternityNo5":
  if ( _callOnChange_xjal ) {
    set_maternityNo5( ((Number) _value_xjal).doubleValue() );
  } else {
    maternityNo5 = ((Number) _value_xjal).doubleValue();
  }
  return true;
case "healthCenterNo5":
```

```
  if ( _callOnChange_xjal ) {
    set_healthCenterNo5( ((Number) _value_xjal).doubleValue() );
  } else {
    healthCenterNo5 = ((Number) _value_xjal).doubleValue();
  }
  return true;
case "otherNo5":
  if ( _callOnChange_xjal ) {
    set_otherNo5( ((Number) _value_xjal).doubleValue() );
  } else {
    otherNo5 = ((Number) _value_xjal).doubleValue();
  }
  return true;
case "communityNo5":
  if ( _callOnChange_xjal ) {
    set_communityNo5( ((Number) _value_xjal).doubleValue() );
  } else {
    communityNo5 = ((Number) _value_xjal).doubleValue();
  }
  return true;
case "districtNo5":
  if ( _callOnChange_xjal ) {
    set_districtNo5( ((Number) _value_xjal).doubleValue() );
  } else {
    districtNo5 = ((Number) _value_xjal).doubleValue();
  }
  return true;
case "centralNo5SE":
  if ( _callOnChange_xjal ) {
    set_centralNo5SE( ((Number) _value_xjal).doubleValue() );
  } else {
    centralNo5SE = ((Number) _value_xjal).doubleValue();
  }
  return true;
case "clinicNo5SE":
  if ( _callOnChange_xjal ) {
    set_clinicNo5SE( ((Number) _value_xjal).doubleValue() );
  } else {
    clinicNo5SE = ((Number) _value_xjal).doubleValue();
  }
  return true;
case "maternityNo5SE":
  if ( _callOnChange_xjal ) {
    set_maternityNo5SE( ((Number) _value_xjal).doubleValue() );
  } else {
    maternityNo5SE = ((Number) _value_xjal).doubleValue();
  }
  return true;
case "healthCenterNo5SE":
  if ( _callOnChange_xjal ) {
    set_healthCenterNo5SE( ((Number) _value_xjal).doubleValue() );
  } else {
    healthCenterNo5SE = ((Number) _value_xjal).doubleValue();
  }
  return true;
case "otherNo5SE":
  if ( _callOnChange_xjal ) {
    set_otherNo5SE( ((Number) _value_xjal).doubleValue() );
  } else {
    otherNo5SE = ((Number) _value_xjal).doubleValue();
  }
  return true;
case "communityNo5SE":
```

```
  if ( _callOnChange_xjal ) {
    set_communityNo5SE( ((Number) _value_xjal).doubleValue() );
  } else {
    communityNo5SE = ((Number) _value_xjal).doubleValue();
  }
  return true;
case "districtNo5SE":
  if ( _callOnChange_xjal ) {
    set_districtNo5SE( ((Number) _value_xjal).doubleValue() );
  } else {
    districtNo5SE = ((Number) _value_xjal).doubleValue();
  }
  return true;
case "anc4":
  if ( _callOnChange_xjal ) {
    set_anc4( ((Number) _value_xjal).doubleValue() );
  } else {
    anc4 = ((Number) _value_xjal).doubleValue();
  }
  return true;
case "unwanted":
  if ( _callOnChange_xjal ) {
    set_unwanted( ((Number) _value_xjal).doubleValue() );
  } else {
    unwanted = ((Number) _value_xjal).doubleValue();
  }
  return true;
case "risk":
  if ( _callOnChange_xjal ) {
    set_risk( ((Number) _value_xjal).doubleValue() );
  } else {
    risk = ((Number) _value_xjal).doubleValue();
  }
  return true;
case "urban":
  if ( _callOnChange_xjal ) {
    set_urban( ((Number) _value_xjal).doubleValue() );
  } else {
    urban = ((Number) _value_xjal).doubleValue();
  }
  return true;
case "twins":
  if ( _callOnChange_xjal ) {
    set_twins( ((Number) _value_xjal).doubleValue() );
  } else {
    twins = ((Number) _value_xjal).doubleValue();
  }
  return true;
case "educSec":
  if ( _callOnChange_xjal ) {
    set_educSec( ((Number) _value_xjal).doubleValue() );
  } else {
    educSec = ((Number) _value_xjal).doubleValue();
  }
  return true;
case "spouseAge":
  if ( _callOnChange_xjal ) {
    set_spouseAge( ((Number) _value_xjal).doubleValue() );
  } else {
    spouseAge = ((Number) _value_xjal).doubleValue();
  }
  return true;
case "age":
```

```
  if ( _callOnChange_xjal ) {
    set_age( ((Number) _value_xjal).doubleValue() );
  } else {
    age = ((Number) _value_xjal).doubleValue();
  }
  return true;
case "csPlanned":
  if ( _callOnChange_xjal ) {
    set_csPlanned( ((Number) _value_xjal).doubleValue() );
  } else {
    csPlanned = ((Number) _value_xjal).doubleValue();
  }
  return true;
case "anc4SE":
  if ( _callOnChange_xjal ) {
    set_anc4SE( ((Number) _value_xjal).doubleValue() );
  } else {
    anc4SE = ((Number) _value_xjal).doubleValue();
  }
  return true;
case "unwantedSE":
  if ( _callOnChange_xjal ) {
    set_unwantedSE( ((Number) _value_xjal).doubleValue() );
  } else {
    unwantedSE = ((Number) _value_xjal).doubleValue();
  }
  return true;
case "riskSE":
  if ( _callOnChange_xjal ) {
    set_riskSE( ((Number) _value_xjal).doubleValue() );
  } else {
    riskSE = ((Number) _value_xjal).doubleValue();
  }
  return true;
case "urbanSE":
  if ( _callOnChange_xjal ) {
    set_urbanSE( ((Number) _value_xjal).doubleValue() );
  } else {
    urbanSE = ((Number) _value_xjal).doubleValue();
  }
  return true;
case "twinsSE":
  if ( _callOnChange_xjal ) {
    set_twinsSE( ((Number) _value_xjal).doubleValue() );
  } else {
    twinsSE = ((Number) _value_xjal).doubleValue();
  }
  return true;
case "educSecSE":
  if ( _callOnChange_xjal ) {
    set_educSecSE( ((Number) _value_xjal).doubleValue() );
  } else {
    educSecSE = ((Number) _value_xjal).doubleValue();
  }
  return true;
case "spouseAgeSE":
  if ( _callOnChange_xjal ) {
    set_spouseAgeSE( ((Number) _value_xjal).doubleValue() );
  } else {
    spouseAgeSE = ((Number) _value_xjal).doubleValue();
  }
  return true;
case "ageSE":
```

```
    if ( _callOnChange_xjal ) {
      set_ageSE( ((Number) _value_xjal).doubleValue() );
    } else {
      ageSE = ((Number) _value_xjal).doubleValue();
    }
    return true;
  case "csPlannedSE":
    if ( _callOnChange_xjal ) {
      set_csPlannedSE( ((Number) _value_xjal).doubleValue() );
    } else {
      csPlannedSE = ((Number) _value_xjal).doubleValue();
    }
    return true;
  case "QIParam":
    if ( _callOnChange_xjal ) {
      set_QIParam( ((Number) _value_xjal).doubleValue() );
    } else {
      QIParam = ((Number) _value_xjal).doubleValue();
    }
    return true;
  case "strategy":
    if ( _callOnChange_xjal ) {
      set_strategy( ((Number) _value_xjal).intValue() );
    } else {
      strategy = ((Number) _value_xjal).intValue();
    }
    return true;
  default:
    return super.setParameter( _name_xjal, _value_xjal, _callOnChange_xjal );
  }
}

@Override
public <T> T getParameter(String _name_xjal) {
  Object _result_xjal;
  switch ( _name_xjal ) {
  case "allowFeesParam": _result_xjal = allowFeesParam; break;
  case "centralHosp1": _result_xjal = centralHosp1; break;
  case "distance1": _result_xjal = distance1; break;
  case "clinic1": _result_xjal = clinic1; break;
  case "maternity1": _result_xjal = maternity1; break;
  case "healthCenter1": _result_xjal = healthCenter1; break;
  case "otherHosp1": _result_xjal = otherHosp1; break;
  case "communityHosp1": _result_xjal = communityHosp1; break;
  case "districtHosp1": _result_xjal = districtHosp1; break;
  case "bobs1": _result_xjal = bobs1; break;
  case "fees1": _result_xjal = fees1; break;
  case "allowAllParam": _result_xjal = allowAllParam; break;
  case "intercept": _result_xjal = intercept; break;
  case "centralHosp2": _result_xjal = centralHosp2; break;
  case "distance2": _result_xjal = distance2; break;
  case "clinic2": _result_xjal = clinic2; break;
  case "maternity2": _result_xjal = maternity2; break;
  case "healthCenter2": _result_xjal = healthCenter2; break;
  case "otherHosp2": _result_xjal = otherHosp2; break;
  case "communityHosp2": _result_xjal = communityHosp2; break;
  case "districtHosp2": _result_xjal = districtHosp2; break;
  case "bobs2": _result_xjal = bobs2; break;
  case "fees2": _result_xjal = fees2; break;
  case "distCenter": _result_xjal = distCenter; break;
  case "distRMS": _result_xjal = distRMS; break;
  case "centralHosp1SE": _result_xjal = centralHosp1SE; break;
  case "distance1SE": _result_xjal = distance1SE; break;
```

```
case "clinic1SE": _result_xjal = clinic1SE; break;
case "maternity1SE": _result_xjal = maternity1SE; break;
case "healthCenter1SE": _result_xjal = healthCenter1SE; break;
case "otherHosp1SE": _result_xjal = otherHosp1SE; break;
case "communityHosp1SE": _result_xjal = communityHosp1SE; break;
case "districtHosp1SE": _result_xjal = districtHosp1SE; break;
case "bobs1SE": _result_xjal = bobs1SE; break;
case "fees1SE": _result_xjal = fees1SE; break;
case "interceptSE": _result_xjal = interceptSE; break;
case "centralHosp2SE": _result_xjal = centralHosp2SE; break;
case "distance2SE": _result_xjal = distance2SE; break;
case "clinic2SE": _result_xjal = clinic2SE; break;
case "maternity2SE": _result_xjal = maternity2SE; break;
case "healthCenter2SE": _result_xjal = healthCenter2SE; break;
case "otherHosp2SE": _result_xjal = otherHosp2SE; break;
case "communityHosp2SE": _result_xjal = communityHosp2SE; break;
case "districtHosp2SE": _result_xjal = districtHosp2SE; break;
case "bobs2SE": _result_xjal = bobs2SE; break;
case "fees2SE": _result_xjal = fees2SE; break;
case "centralCS": _result_xjal = centralCS; break;
case "clinicCS": _result_xjal = clinicCS; break;
case "maternityCS": _result_xjal = maternityCS; break;
case "healthCenterCS": _result_xjal = healthCenterCS; break;
case "otherCS": _result_xjal = otherCS; break;
case "communityCS": _result_xjal = communityCS; break;
case "districtCS": _result_xjal = districtCS; break;
case "centralCSSE": _result_xjal = centralCSSE; break;
case "clinicCSSE": _result_xjal = clinicCSSE; break;
case "maternityCSSE": _result_xjal = maternityCSSE; break;
case "healthCenterCSSE": _result_xjal = healthCenterCSSE; break;
case "otherCSSE": _result_xjal = otherCSSE; break;
case "communityCSSE": _result_xjal = communityCSSE; break;
case "districtCSSE": _result_xjal = districtCSSE; break;
case "centralNo": _result_xjal = centralNo; break;
case "clinicNo": _result_xjal = clinicNo; break;
case "maternityNo": _result_xjal = maternityNo; break;
case "healthCenterNo": _result_xjal = healthCenterNo; break;
case "otherNo": _result_xjal = otherNo; break;
case "communityNo": _result_xjal = communityNo; break;
case "districtNo": _result_xjal = districtNo; break;
case "centralNoSE": _result_xjal = centralNoSE; break;
case "clinicNoSE": _result_xjal = clinicNoSE; break;
case "maternityNoSE": _result_xjal = maternityNoSE; break;
case "healthCenterNoSE": _result_xjal = healthCenterNoSE; break;
case "otherNoSE": _result_xjal = otherNoSE; break;
case "communityNoSE": _result_xjal = communityNoSE; break;
case "districtNoSE": _result_xjal = districtNoSE; break;
case "central5": _result_xjal = central5; break;
case "clinic5": _result_xjal = clinic5; break;
case "maternity5": _result_xjal = maternity5; break;
case "healthCenter5": _result_xjal = healthCenter5; break;
case "other5": _result_xjal = other5; break;
case "community5": _result_xjal = community5; break;
case "district5": _result_xjal = district5; break;
case "central5SE": _result_xjal = central5SE; break;
case "clinic5SE": _result_xjal = clinic5SE; break;
case "maternity5SE": _result_xjal = maternity5SE; break;
case "healthCenter5SE": _result_xjal = healthCenter5SE; break;
case "other5SE": _result_xjal = other5SE; break;
case "community5SE": _result_xjal = community5SE; break;
case "district5SE": _result_xjal = district5SE; break;
case "centralNo5": _result_xjal = centralNo5; break;
case "clinicNo5": _result_xjal = clinicNo5; break;
```

```
    case "maternityNo5": _result_xjal = maternityNo5; break;
    case "healthCenterNo5": _result_xjal = healthCenterNo5; break;
    case "otherNo5": _result_xjal = otherNo5; break;
    case "communityNo5": _result_xjal = communityNo5; break;
    case "districtNo5": _result_xjal = districtNo5; break;
    case "centralNo5SE": _result_xjal = centralNo5SE; break;
    case "clinicNo5SE": _result_xjal = clinicNo5SE; break;
    case "maternityNo5SE": _result_xjal = maternityNo5SE; break;
    case "healthCenterNo5SE": _result_xjal = healthCenterNo5SE; break;
    case "otherNo5SE": _result_xjal = otherNo5SE; break;
    case "communityNo5SE": _result_xjal = communityNo5SE; break;
    case "districtNo5SE": _result_xjal = districtNo5SE; break;
    case "anc4": _result_xjal = anc4; break;
    case "unwanted": _result_xjal = unwanted; break;
    case "risk": _result_xjal = risk; break;
    case "urban": _result_xjal = urban; break;
    case "twins": _result_xjal = twins; break;
    case "educSec": _result_xjal = educSec; break;
    case "spouseAge": _result_xjal = spouseAge; break;
    case "age": _result_xjal = age; break;
    case "csPlanned": _result_xjal = csPlanned; break;
    case "anc4SE": _result_xjal = anc4SE; break;
    case "unwantedSE": _result_xjal = unwantedSE; break;
    case "riskSE": _result_xjal = riskSE; break;
    case "urbanSE": _result_xjal = urbanSE; break;
    case "twinsSE": _result_xjal = twinsSE; break;
    case "educSecSE": _result_xjal = educSecSE; break;
    case "spouseAgeSE": _result_xjal = spouseAgeSE; break;
    case "ageSE": _result_xjal = ageSE; break;
    case "csPlannedSE": _result_xjal = csPlannedSE; break;
    case "QIParam": _result_xjal = QIParam; break;
    case "strategy": _result_xjal = strategy; break;
    default: _result_xjal = super.getParameter( _name_xjal ); break;
    }
    return (T) _result_xjal;
  }

  @AnyLogicInternalCodegenAPI
  private static String[] _parameterNames_xjal;

  @Override
  public String[] getParameterNames() {
    String[] result = _parameterNames_xjal;
    if (result == null) {
      List<String> list = new ArrayList<>( Arrays.asList( super.getParameterNames() )
);
      list.add( "allowFeesParam" );
      list.add( "centralHosp1" );
      list.add( "distance1" );
      list.add( "clinic1" );
      list.add( "maternity1" );
      list.add( "healthCenter1" );
      list.add( "otherHosp1" );
      list.add( "communityHosp1" );
      list.add( "districtHosp1" );
      list.add( "bobs1" );
      list.add( "fees1" );
      list.add( "allowAllParam" );
      list.add( "intercept" );
      list.add( "centralHosp2" );
      list.add( "distance2" );
      list.add( "clinic2" );
      list.add( "maternity2" );
```

```
list.add( "healthCenter2" );
list.add( "otherHosp2" );
list.add( "communityHosp2" );
list.add( "districtHosp2" );
list.add( "bobs2" );
list.add( "fees2" );
list.add( "distCenter" );
list.add( "distRMS" );
list.add( "centralHosp1SE" );
list.add( "distance1SE" );
list.add( "clinic1SE" );
list.add( "maternity1SE" );
list.add( "healthCenter1SE" );
list.add( "otherHosp1SE" );
list.add( "communityHosp1SE" );
list.add( "districtHosp1SE" );
list.add( "bobs1SE" );
list.add( "fees1SE" );
list.add( "interceptSE" );
list.add( "centralHosp2SE" );
list.add( "distance2SE" );
list.add( "clinic2SE" );
list.add( "maternity2SE" );
list.add( "healthCenter2SE" );
list.add( "otherHosp2SE" );
list.add( "communityHosp2SE" );
list.add( "districtHosp2SE" );
list.add( "bobs2SE" );
list.add( "fees2SE" );
list.add( "centralCS" );
list.add( "clinicCS" );
list.add( "maternityCS" );
list.add( "healthCenterCS" );
list.add( "otherCS" );
list.add( "communityCS" );
list.add( "districtCS" );
list.add( "centralCSSE" );
list.add( "clinicCSSE" );
list.add( "maternityCSSE" );
list.add( "healthCenterCSSE" );
list.add( "otherCSSE" );
list.add( "communityCSSE" );
list.add( "districtCSSE" );
list.add( "centralNo" );
list.add( "clinicNo" );
list.add( "maternityNo" );
list.add( "healthCenterNo" );
list.add( "otherNo" );
list.add( "communityNo" );
list.add( "districtNo" );
list.add( "centralNoSE" );
list.add( "clinicNoSE" );
list.add( "maternityNoSE" );
list.add( "healthCenterNoSE" );
list.add( "otherNoSE" );
list.add( "communityNoSE" );
list.add( "districtNoSE" );
list.add( "central5" );
list.add( "clinic5" );
list.add( "maternity5" );
list.add( "healthCenter5" );
list.add( "other5" );
list.add( "community5" );
```

```
      list.add( "district5" );
      list.add( "central5SE" );
      list.add( "clinic5SE" );
      list.add( "maternity5SE" );
      list.add( "healthCenter5SE" );
      list.add( "other5SE" );
      list.add( "community5SE" );
      list.add( "district5SE" );
      list.add( "centralNo5" );
      list.add( "clinicNo5" );
      list.add( "maternityNo5" );
      list.add( "healthCenterNo5" );
      list.add( "otherNo5" );
      list.add( "communityNo5" );
      list.add( "districtNo5" );
      list.add( "centralNo5SE" );
      list.add( "clinicNo5SE" );
      list.add( "maternityNo5SE" );
      list.add( "healthCenterNo5SE" );
      list.add( "otherNo5SE" );
      list.add( "communityNo5SE" );
      list.add( "districtNo5SE" );
      list.add( "anc4" );
      list.add( "unwanted" );
      list.add( "risk" );
      list.add( "urban" );
      list.add( "twins" );
      list.add( "educSec" );
      list.add( "spouseAge" );
      list.add( "age" );
      list.add( "csPlanned" );
      list.add( "anc4SE" );
      list.add( "unwantedSE" );
      list.add( "riskSE" );
      list.add( "urbanSE" );
      list.add( "twinsSE" );
      list.add( "educSecSE" );
      list.add( "spouseAgeSE" );
      list.add( "ageSE" );
      list.add( "csPlannedSE" );
      list.add( "QIParam" );
      list.add( "strategy" );
      result = list.toArray( new String[ list.size() ] );
      _parameterNames_xjal = result;
    }
    return result;
  }
  // Plain Variables

  public
double
 bCentral1;
  public
double
 bCentral2;
  public
double
 bDistrict1;
  public
double
 bOther1;
  public
double
```

```
 bCommunity1;
  public
double
 bClinic1;
  public
double
 bMaternity1;
  public
double
 bHealthCenter1;
  public
double
 bBobs1;
  public
double
 bFees1;
  public
double
 bQuality1;
  public
double
 bDistance1;
  public
double
 bDistrict2;
  public
double
 bOther2;
  public
double
 bCommunity2;
  public
double
 bClinic2;
  public
double
 bMaternity2;
  public
double
 bHealthCenter2;
  public
double
 bBobs2;
  public
double
 bFees2;
  public
double
 bQuality2;
  public
double
 bDistance2;
  public
double
 bPoorest;
  public
double
 bMiddle;
  public
double
 bPoor;
  public
double
```

```
 bIntercept;
  public
double
 bRichest;
  public
double
 bRich;
  public
double
 bIlliterate;
  public
double
 bBlind;
  public
double
 bPrimip;
  public
double
 bUrban;
  public
double
 bSpouseAge;
  public
double
 bAge;
  public
double
 bUnwanted;
  public
double
 bTwins;
  public
double
 bEducSec;
  public
double
 bCSPlanned;
  public
double
 bRisk;
  public
double
 bANC4;
  public
double
 vAgentID;
  public
boolean
 allowFees;
  public
boolean
 allowAll;
  public
double
 QI;

  // Collection Variables
  public
ArrayList <
Hospital > hospitalList = new ArrayList<Hospital>();
  /**
   * All facilities
   */
```

```
  public
ArrayList <
Hospital > allowedList = new ArrayList<Hospital>();
  @AnyLogicInternalCodegenAPI
  private static Map<String, IElementDescriptor> elementDesciptors_xjal =
createElementDescriptors( Main.class );

  @AnyLogicInternalCodegenAPI
  @Override
  public Map<String, IElementDescriptor> getElementDesciptors() {
    return elementDesciptors_xjal;
  }


  // Events

  public EventCondition classesAssigned = new EventCondition(this);


  @Override
  @AnyLogicInternalCodegenAPI
  public String getNameOf( EventCondition _e ) {
    if ( _e == classesAssigned ) return "classesAssigned";
    return super.getNameOf( _e );
  }

  @Override
  @AnyLogicInternalCodegenAPI
  public boolean testConditionOf( EventCondition _e ) {

    if ( _e == classesAssigned) return
this.classAssigned == this.popSize
;
    return super.testConditionOf( _e );
  }

  @Override
  @AnyLogicInternalCodegenAPI
  public void executeActionOf( EventCondition _e ) {
    if ( _e == classesAssigned) {
      EventCondition self = _e;

people(0).sendToAll("Decide");
;
      return ;
    }
    super.executeActionOf( _e );
  }





  // Embedded Objects

  @AnyLogicInternalCodegenAPI
  private static final AgentAnimationSettings _hospitals_animationSettings_xjal = new
AgentAnimationSettings(1000L, 1000000000L);
  @AnyLogicInternalCodegenAPI
  private static final AgentAnimationSettings _people_animationSettings_xjal = new
AgentAnimationSettings(1000L, 1000000000L);

  public String getNameOf( Agent ao ) {
```

```
    return super.getNameOf( ao );
  }

  public AgentAnimationSettings getAnimationSettingsOf( Agent ao ) {
    return super.getAnimationSettingsOf( ao );
  }

  public class _hospitals_Population extends AgentArrayList<Hospital> {
    _hospitals_Population( Agent owner ) {
      super( owner );
    }

    @AnyLogicInternalCodegenAPI
    public Hospital instantiateAgent( int index ) {
       return instantiate_hospitals_xjal( index );
    }

    @AnyLogicInternalCodegenAPI
    public void callSetupParameters( Hospital agent, int index, TableInput tableInput
) {
      setupParameters_hospitals_xjal( agent, index, tableInput );
    }

    @AnyLogicInternalCodegenAPI
    public void callCreate( Hospital agent, int index, TableInput tableInput ) {
      create_hospitals_xjal( agent, index, tableInput );
    }

    @AnyLogicInternalCodegenAPI
    public boolean isPresentationEnabled() {
      return true;
    }


    public double hospitalBobs() {
      return _hospitals_hospitalBobs_xjal();
    }

    public double hospitalFees() {
      return _hospitals_hospitalFees_xjal();
    }
  }

  @AnyLogicCustomProposalType(value = AnyLogicCustomProposalType.Label.POPULATION,
customText = "Hospital")
  public _hospitals_Population hospitals = new _hospitals_Population( this );
  public class _people_Population extends AgentArrayList<Person> {
    _people_Population( Agent owner ) {
      super( owner );
    }

    @AnyLogicInternalCodegenAPI
    public Person instantiateAgent( int index ) {
       return instantiate_people_xjal( index );
    }

    @AnyLogicInternalCodegenAPI
    public void callSetupParameters( Person agent, int index, TableInput tableInput )
{
      setupParameters_people_xjal( agent, index, tableInput );
    }

    @AnyLogicInternalCodegenAPI
```

```
public void callCreate( Person agent, int index, TableInput tableInput ) {
  create_people_xjal( agent, index, tableInput );
}

@AnyLogicInternalCodegenAPI
public boolean isPresentationEnabled() {
  return true;
}


public int travelTime() {
  return _people_travelTime_xjal();
}

public int nPoorest() {
  return _people_nPoorest_xjal();
}

public int nPoor() {
  return _people_nPoor_xjal();
}

public int nMiddle() {
  return _people_nMiddle_xjal();
}

public int nRich() {
  return _people_nRich_xjal();
}

public int nRichest() {
  return _people_nRichest_xjal();
}

public int nClass2() {
  return _people_nClass2_xjal();
}

public int nUrban() {
  return _people_nUrban_xjal();
}

public double uDiff() {
  return _people_uDiff_xjal();
}

public int nCentral() {
  return _people_nCentral_xjal();
}

public int nDistrict() {
  return _people_nDistrict_xjal();
}

public int nCommunity() {
  return _people_nCommunity_xjal();
}

public int nOther() {
  return _people_nOther_xjal();
}

public int nHealthCenter() {
```

```
    return _people_nHealthCenter_xjal();
  }

  public int nMaternity() {
    return _people_nMaternity_xjal();
  }

  public int nClinic() {
    return _people_nClinic_xjal();
  }

  public int classAssigned() {
    return _people_classAssigned_xjal();
  }

  public int hospitalAssigned() {
    return _people_hospitalAssigned_xjal();
  }

  public double uDiffPoorest() {
    return _people_uDiffPoorest_xjal();
  }

  public double uDiffPoor() {
    return _people_uDiffPoor_xjal();
  }

  public double uDiffMiddle() {
    return _people_uDiffMiddle_xjal();
  }

  public double uDiffRich() {
    return _people_uDiffRich_xjal();
  }

  public double uDiffRichest() {
    return _people_uDiffRichest_xjal();
  }

  public int dist60km() {
    return _people_dist60km_xjal();
  }

  public int distFurther() {
    return _people_distFurther_xjal();
  }

  public double mortPoorest() {
    return _people_mortPoorest_xjal();
  }

  public double mortPoor() {
    return _people_mortPoor_xjal();
  }

  public double mortMiddle() {
    return _people_mortMiddle_xjal();
  }

  public double mortRich() {
    return _people_mortRich_xjal();
  }
```

```
    public double mortRichest() {
      return _people_mortRichest_xjal();
    }

    public int numCS() {
      return _people_numCS_xjal();
    }

    public double totalCost() {
      return _people_totalCost_xjal();
    }

    public double catExpPoorest() {
      return _people_catExpPoorest_xjal();
    }

    public double catExpPoor() {
      return _people_catExpPoor_xjal();
    }

    public double catExpMiddle() {
      return _people_catExpMiddle_xjal();
    }

    public double catExpRich() {
      return _people_catExpRich_xjal();
    }

    public double catExpRichest() {
      return _people_catExpRichest_xjal();
    }
  }

  @AnyLogicCustomProposalType(value = AnyLogicCustomProposalType.Label.POPULATION,
customText = "Person")
  public _people_Population people = new _people_Population( this );

  public String getNameOf( AgentList<?> aolist ) {
    if( aolist == hospitals ) return "hospitals";
    if( aolist == people ) return "people";
    return super.getNameOf( aolist );
  }

  public AgentAnimationSettings getAnimationSettingsOf( AgentList<?> aolist ) {
    if( aolist == hospitals ) return _hospitals_animationSettings_xjal;
    if( aolist == people ) return _people_animationSettings_xjal;
    return super.getAnimationSettingsOf( aolist );
  }

  /**
   * Returns the agent element at the specified position in hospitals
   * population.
   * @see com.anylogic.engine.AgentList#get(int)
   * @since 7.3.7
   */
  public Hospital hospitals(int index) {
    return hospitals.get( index );
  }

  /**
   * This method creates and adds new embedded object in the replicated embedded
object collection hospitals<br>
   * @return newly created embedded object
```

```
    */
  public Hospital add_hospitals() {
    int index = hospitals.size();
    Hospital _result_xjal = instantiate_hospitals_xjal( index );
    hospitals.callSetupParameters( _result_xjal, index );
    hospitals.callCreate( _result_xjal, index );
    _result_xjal.start();
    return _result_xjal;
  }

  /**
   * This method creates and adds new embedded object in the replicated embedded
object collection hospitals<br>
   * This method uses given parameter values to setup created embedded object<br>
   * Index of this new embedded object instance can be obtained through calling
<code>hospitals.size()</code> method <strong>before</strong> this method is called
   * @param id
   * @param type
   * @param management
   * @param urban
   * @param latitude
   * @param longitude
   * @param bobsCounterfactual
   * @param q
   * @param feesCounterfactual
   * @param distance
   * @param hospital
   * @param cs
   * @param bemonc
   * @param upgradeCS
   * @param upgradeBemonc
   * @return newly created embedded object
   */
  public Hospital add_hospitals( String id, String type, String management, boolean
urban, double latitude, double longitude, double bobsCounterfactual, double q, int
feesCounterfactual, double distance, boolean hospital, boolean cs, boolean bemonc,
boolean upgradeCS, boolean upgradeBemonc ) {
    int index = hospitals.size();
    Hospital _result_xjal = instantiate_hospitals_xjal( index );
    // Setup parameters
    _result_xjal.markParametersAreSet();
    _result_xjal.id = id;
    _result_xjal.type = type;
    _result_xjal.management = management;
    _result_xjal.urban = urban;
    _result_xjal.latitude = latitude;
    _result_xjal.longitude = longitude;
    _result_xjal.bobsCounterfactual = bobsCounterfactual;
    _result_xjal.q = q;
    _result_xjal.feesCounterfactual = feesCounterfactual;
    _result_xjal.distance = distance;
    _result_xjal.hospital = hospital;
    _result_xjal.cs = cs;
    _result_xjal.bemonc = bemonc;
    _result_xjal.upgradeCS = upgradeCS;
    _result_xjal.upgradeBemonc = upgradeBemonc;
    // Finish embedded object creation
    hospitals.callCreate( _result_xjal, index );
    _result_xjal.start();
    return _result_xjal;
  }

  /**
```

```
   * This method removes the given embedded object from the replicated embedded object
collection hospitals<br>
   * The given object is destroyed, but not immediately in common case.
   * @param object the active object - element of replicated embedded object hospitals
- which should be removed
   * @return <code>true</code> if object was removed successfully, <code>false</code>
if it doesn't belong to hospitals
   */
  public boolean remove_hospitals( Hospital object ) {
    if( ! hospitals._remove( object ) ) {
      return false;
    }
    object.removeFromFlowchart();
    object.setDestroyed();
    return true;
  }
  /**
   * Returns the agent element at the specified position in people
   * population.
   * @see com.anylogic.engine.AgentList#get(int)
   * @since 7.3.7
   */
  public Person people(int index) {
    return people.get( index );
  }

  /**
   * This method creates and adds new embedded object in the replicated embedded
object collection people<br>
   * @return newly created embedded object
   */
  public Person add_people() {
    int index = people.size();
    Person _result_xjal = instantiate_people_xjal( index );
    people.callSetupParameters( _result_xjal, index );
    people.callCreate( _result_xjal, index );
    _result_xjal.start();
    return _result_xjal;
  }

  /**
   * This method creates and adds new embedded object in the replicated embedded
object collection people<br>
   * This method uses given parameter values to setup created embedded object<br>
   * Index of this new embedded object instance can be obtained through calling
<code>people.size()</code> method <strong>before</strong> this method is called
   * @param longitude
   * @param latitude
   * @param wealth
   * @param educSec
   * @param age
   * @param ANC4
   * @param urban
   * @param twins
   * @param unwanted
   * @param primip
   * @param blind
   * @param illiterate
   * @param spouseAge
   * @param risk
   * @param csPlanned
   * @param income
   * @return newly created embedded object
```

```
   */
  public Person add_people( double longitude, double latitude, int wealth, int
educSec, int age, int ANC4, int urban, int twins, int unwanted, int primip, int blind,
int illiterate, int spouseAge, int risk, int csPlanned, double income ) {
    int index = people.size();
    Person _result_xjal = instantiate_people_xjal( index );
    // Setup parameters
    _result_xjal.markParametersAreSet();
    _result_xjal.longitude = longitude;
    _result_xjal.latitude = latitude;
    _result_xjal.wealth = wealth;
    _result_xjal.educSec = educSec;
    _result_xjal.age = age;
    _result_xjal.ANC4 = ANC4;
    _result_xjal.urban = urban;
    _result_xjal.twins = twins;
    _result_xjal.unwanted = unwanted;
    _result_xjal.primip = primip;
    _result_xjal.blind = blind;
    _result_xjal.illiterate = illiterate;
    _result_xjal.spouseAge = spouseAge;
    _result_xjal.risk = risk;
    _result_xjal.csPlanned = csPlanned;
    _result_xjal.income = income;
    // Finish embedded object creation
    people.callCreate( _result_xjal, index );
    _result_xjal.start();
    return _result_xjal;
  }

  /**
   * This method removes the given embedded object from the replicated embedded object
collection people<br>
   * The given object is destroyed, but not immediately in common case.
   * @param object the active object - element of replicated embedded object people -
which should be removed
   * @return <code>true</code> if object was removed successfully, <code>false</code>
if it doesn't belong to people
   */
  public boolean remove_people( Person object ) {
    if( ! people._remove( object ) ) {
      return false;
    }
    object.removeFromFlowchart();
    object.setDestroyed();
    return true;
  }

  /**
   * Creates an embedded object instance and adds it to the end of replicated embedded
object list<br>
   * <i>This method should not be called by user</i>
   */
  protected Hospital instantiate_hospitals_xjal( final int index ) {
    Hospital _result_xjal = new Hospital( getEngine(), this, hospitals );

    hospitals._add( _result_xjal );

    return _result_xjal;
  }

  /**
   * Setups parameters of an embedded object instance<br>
```

```
   * This method should not be called by user
   */
  private void setupParameters_hospitals_xjal( final Hospital self, final int index )
{
    setupParameters_hospitals_xjal( self, index, null );
  }

  /**
   * Setups an embedded object instance<br>
   * This method should not be called by user
   */
  @AnyLogicInternalCodegenAPI
  private void create_hospitals_xjal( Hospital self, final int index ) {
    create_hospitals_xjal(self, index, null );
  }


  /**
   * Setups parameters of an embedded object instance<br>
   * This method should not be called by user
   */
  private void setupParameters_hospitals_xjal( final Hospital self, final int index,
TableInput _t ) {
    if (_t != null) {
      self.id = _t.getValue( "id", String.class );
    } else {
    self.id = self._id_DefaultValue_xjal();
    }
    if (_t != null) {
      self.type = _t.getValue( "type", String.class );
    } else {
    self.type = self._type_DefaultValue_xjal();
    }
    if (_t != null) {
      self.management = _t.getValue( "management", String.class );
    } else {
    self.management = self._management_DefaultValue_xjal();
    }
    if (_t != null) {
      self.urban = _t.getValue( "urban", boolean.class );
    } else {
    self.urban = self._urban_DefaultValue_xjal();
    }
    if (_t != null) {
      self.latitude = _t.getValue( "latitude", double.class );
    } else {
    self.latitude = self._latitude_DefaultValue_xjal();
    }
    if (_t != null) {
      self.longitude = _t.getValue( "longitude", double.class );
    } else {
    self.longitude = self._longitude_DefaultValue_xjal();
    }
    if (_t != null) {
      self.bobsCounterfactual = _t.getValue( "bobs", double.class );
    } else {
    self.bobsCounterfactual = self._bobsCounterfactual_DefaultValue_xjal();
    }
    if (_t != null) {
      self.q = _t.getValue( "q", double.class );
    } else {
    self.q = self._q_DefaultValue_xjal();
    }
```

```
    if (_t != null) {
      self.feesCounterfactual = _t.getValue( "fees", int.class );
    } else {
    self.feesCounterfactual = self._feesCounterfactual_DefaultValue_xjal();
    }
    self.distance = self._distance_DefaultValue_xjal();
    if (_t != null) {
      self.hospital = _t.getValue( "hospital", boolean.class );
    } else {
    self.hospital = self._hospital_DefaultValue_xjal();
    }
    if (_t != null) {
      self.cs = _t.getValue( "cs", boolean.class );
    } else {
    self.cs = self._cs_DefaultValue_xjal();
    }
    if (_t != null) {
      self.bemonc = _t.getValue( "bemonc", boolean.class );
    } else {
    self.bemonc = self._bemonc_DefaultValue_xjal();
    }
    if (_t != null) {
      self.upgradeCS = _t.getValue( "upgradecs", boolean.class );
    } else {
    self.upgradeCS = self._upgradeCS_DefaultValue_xjal();
    }
    if (_t != null) {
      self.upgradeBemonc = _t.getValue( "upgradebemonc", boolean.class );
    } else {
    self.upgradeBemonc = self._upgradeBemonc_DefaultValue_xjal();
    }
  }

  /**
   * Setups an embedded object instance<br>
   * This method should not be called by user
   */
  @AnyLogicInternalCodegenAPI
  private void create_hospitals_xjal( Hospital self, final int index, TableInput _t )
{
      double _initial_speed =
0
;
      self.setSpeed(_initial_speed, MPS);
    self.setEnvironment( this );
    double _x_xjal =
self.latitude
;
    double _y_xjal =
self.longitude
;
    self.setXY( _x_xjal, _y_xjal );
    self.create();

    // Port connections
  }
  /**
   * Creates an embedded object instance and adds it to the end of replicated embedded
object list<br>
   * <i>This method should not be called by user</i>
   */
  protected Person instantiate_people_xjal( final int index ) {
    Person _result_xjal = new Person( getEngine(), this, people );
```

```
    people._add( _result_xjal );

    return _result_xjal;
  }

  /**
   * Setups parameters of an embedded object instance<br>
   * This method should not be called by user
   */
  private void setupParameters_people_xjal( final Person self, final int index ) {
    setupParameters_people_xjal( self, index, null );
  }

  /**
   * Setups an embedded object instance<br>
   * This method should not be called by user
   */
  @AnyLogicInternalCodegenAPI
  private void create_people_xjal( Person self, final int index ) {
    create_people_xjal(self, index, null );
  }


  /**
   * Setups parameters of an embedded object instance<br>
   * This method should not be called by user
   */
  private void setupParameters_people_xjal( final Person self, final int index,
TableInput _t ) {
    if (_t != null) {
      self.longitude = _t.getValue( "longitude", double.class );
    } else {
    self.longitude = self._longitude_DefaultValue_xjal();
    }
    if (_t != null) {
      self.latitude = _t.getValue( "latitude", double.class );
    } else {
    self.latitude = self._latitude_DefaultValue_xjal();
    }
    if (_t != null) {
      self.wealth = _t.getValue( "wealth", int.class );
    } else {
    self.wealth = self._wealth_DefaultValue_xjal();
    }
    self.educSec = self._educSec_DefaultValue_xjal();
    if (_t != null) {
      self.age = _t.getValue( "age", int.class );
    } else {
    self.age = self._age_DefaultValue_xjal();
    }
    self.ANC4 = self._ANC4_DefaultValue_xjal();
    self.urban = self._urban_DefaultValue_xjal();
    self.twins = self._twins_DefaultValue_xjal();
    self.unwanted = self._unwanted_DefaultValue_xjal();
    self.primip = self._primip_DefaultValue_xjal();
    self.blind = self._blind_DefaultValue_xjal();
    self.illiterate = self._illiterate_DefaultValue_xjal();
    self.spouseAge = self._spouseAge_DefaultValue_xjal();
    self.risk = self._risk_DefaultValue_xjal();
    self.csPlanned = self._csPlanned_DefaultValue_xjal();
    if (_t != null) {
      self.income = _t.getValue( "income", double.class );
```

```
    } else {
    self.income = self._income_DefaultValue_xjal();
    }
  }

  /**
   * Setups an embedded object instance<br>
   * This method should not be called by user
   */
  @AnyLogicInternalCodegenAPI
  private void create_people_xjal( Person self, final int index, TableInput _t ) {
       double _initial_speed =
30 //(randomTrue(0.014)) ? 10.0 : 1.34
;
       self.setSpeed(_initial_speed, KPH);
    self.setEnvironment( this );
    double _x_xjal =
self.latitude
;
    double _y_xjal =
self.longitude
;
    self.setXY( _x_xjal, _y_xjal );
    self.create();

    // Port connections
  }

  /**
   * <i>This method should not be called by user</i>
   */
  private double _hospitals_hospitalBobs_xjal() {
    int _cnt = 0;
    double _value = 0;
    for ( Hospital item : hospitals ) {
        _cnt++;
        _value +=
item.bobs
;
    }
    return _cnt > 0 ? _value / _cnt : 0;
  }
  /**
   * <i>This method should not be called by user</i>
   */
  private double _hospitals_hospitalFees_xjal() {
    int _cnt = 0;
    double _value = 0;
    for ( Hospital item : hospitals ) {
        _cnt++;
        _value +=
item.fees
;
    }
    return _cnt > 0 ? _value / _cnt : 0;
  }
  /**
   * <i>This method should not be called by user</i>
   */
  private int _people_travelTime_xjal() {
    int _value = 0;
    for ( Person item : people ) {
      boolean _t =
```

```
item.travelTime > 2.0
;
      if ( _t ) {
        _value++;
      }
    }
    return _value;
  }
  /**
   * <i>This method should not be called by user</i>
   */
  private int _people_nPoorest_xjal() {
    int _value = 0;
    for ( Person item : people ) {
      boolean _t =
item.wealth == 1
;
      if ( _t ) {
        _value++;
      }
    }
    return _value;
  }
  /**
   * <i>This method should not be called by user</i>
   */
  private int _people_nPoor_xjal() {
    int _value = 0;
    for ( Person item : people ) {
      boolean _t =
item.wealth == 2
;
      if ( _t ) {
        _value++;
      }
    }
    return _value;
  }
  /**
   * <i>This method should not be called by user</i>
   */
  private int _people_nMiddle_xjal() {
    int _value = 0;
    for ( Person item : people ) {
      boolean _t =
item.wealth == 3
;
      if ( _t ) {
        _value++;
      }
    }
    return _value;
  }
  /**
   * <i>This method should not be called by user</i>
   */
  private int _people_nRich_xjal() {
    int _value = 0;
    for ( Person item : people ) {
      boolean _t =
item.wealth == 4
;
      if ( _t ) {
```

```
        _value++;
      }
    }
    return _value;
  }
  /**
   * <i>This method should not be called by user</i>
   */
  private int _people_nRichest_xjal() {
    int _value = 0;
    for ( Person item : people ) {
      boolean _t =
item.wealth == 5
;
      if ( _t ) {
        _value++;
      }
    }
    return _value;
  }
  /**
   * <i>This method should not be called by user</i>
   */
  private int _people_nClass2_xjal() {
    int _value = 0;
    for ( Person item : people ) {
      boolean _t =
item.segment == 2
;
      if ( _t ) {
        _value++;
      }
    }
    return _value;
  }
  /**
   * <i>This method should not be called by user</i>
   */
  private int _people_nUrban_xjal() {
    int _value = 0;
    for ( Person item : people ) {
      boolean _t =
item.urban == 1
;
      if ( _t ) {
        _value++;
      }
    }
    return _value;
  }
  /**
   * <i>This method should not be called by user</i>
   */
  private double _people_uDiff_xjal() {
    int _cnt = 0;
    double _value = 0;
    for ( Person item : people ) {
        _cnt++;
        _value +=
item.uDiff
;
    }
    return _cnt > 0 ? _value / _cnt : 0;
```

```
  }
  /**
   * <i>This method should not be called by user</i>
   */
  private int _people_nCentral_xjal() {
    int _value = 0;
    for ( Person item : people ) {
      boolean _t =
item.destType.equalsIgnoreCase("central hospital")
;
      if ( _t ) {
        _value++;
      }
    }
    return _value;
  }
  /**
   * <i>This method should not be called by user</i>
   */
  private int _people_nDistrict_xjal() {
    int _value = 0;
    for ( Person item : people ) {
      boolean _t =
item.destType.equalsIgnoreCase("District hospital")
;
      if ( _t ) {
        _value++;
      }
    }
    return _value;
  }
  /**
   * <i>This method should not be called by user</i>
   */
  private int _people_nCommunity_xjal() {
    int _value = 0;
    for ( Person item : people ) {
      boolean _t =
item.destType.equalsIgnoreCase("Community hospital")
;
      if ( _t ) {
        _value++;
      }
    }
    return _value;
  }
  /**
   * <i>This method should not be called by user</i>
   */
  private int _people_nOther_xjal() {
    int _value = 0;
    for ( Person item : people ) {
      boolean _t =
item.destType.equalsIgnoreCase("Other hospital")
;
      if ( _t ) {
        _value++;
      }
    }
    return _value;
  }
  /**
   * <i>This method should not be called by user</i>
```

```
   */
  private int _people_nHealthCenter_xjal() {
    int _value = 0;
    for ( Person item : people ) {
      boolean _t = 
item.destType.equalsIgnoreCase("Health center")
;
      if ( _t ) {
        _value++;
      }
    }
    return _value;
  }
  /**
   * <i>This method should not be called by user</i>
   */
  private int _people_nMaternity_xjal() {
    int _value = 0;
    for ( Person item : people ) {
      boolean _t = 
item.destType.equalsIgnoreCase("Maternity")
;
      if ( _t ) {
        _value++;
      }
    }
    return _value;
  }
  /**
   * <i>This method should not be called by user</i>
   */
  private int _people_nClinic_xjal() {
    int _value = 0;
    for ( Person item : people ) {
      boolean _t = 
item.destType.equalsIgnoreCase("Clinic")
;
      if ( _t ) {
        _value++;
      }
    }
    return _value;
  }
  /**
   * <i>This method should not be called by user</i>
   */
  private int _people_classAssigned_xjal() {
    int _value = 0;
    for ( Person item : people ) {
      boolean _t = 
item.segment > 0
;
      if ( _t ) {
        _value++;
      }
    }
    return _value;
  }
  /**
   * <i>This method should not be called by user</i>
   */
  private int _people_hospitalAssigned_xjal() {
    int _value = 0;
```

```
    for ( Person item : people ) {
      boolean _t = 
item.destHospital != null
;
      if ( _t ) {
        _value++;
      }
    }
    return _value;
  }
  /**
   * <i>This method should not be called by user</i>
   */
  private double _people_uDiffPoorest_xjal() {
    int _cnt = 0;
    double _value = 0;
    for ( Person item : people ) {
      boolean _t = 
item.wealth == 1
;
      if ( _t ) {
        _cnt++;
        _value += 
item.uDiff
;
      }
    }
    return _cnt > 0 ? _value / _cnt : 0;
  }
  /**
   * <i>This method should not be called by user</i>
   */
  private double _people_uDiffPoor_xjal() {
    int _cnt = 0;
    double _value = 0;
    for ( Person item : people ) {
      boolean _t = 
item.wealth == 2
;
      if ( _t ) {
        _cnt++;
        _value += 
item.uDiff
;
      }
    }
    return _cnt > 0 ? _value / _cnt : 0;
  }
  /**
   * <i>This method should not be called by user</i>
   */
  private double _people_uDiffMiddle_xjal() {
    int _cnt = 0;
    double _value = 0;
    for ( Person item : people ) {
      boolean _t = 
item.wealth == 3
;
      if ( _t ) {
        _cnt++;
        _value += 
item.uDiff
;
```

```
      }
    }
    return _cnt > 0 ? _value / _cnt : 0;
  }
  /**
   * <i>This method should not be called by user</i>
   */
  private double _people_uDiffRich_xjal() {
    int _cnt = 0;
    double _value = 0;
    for ( Person item : people ) {
      boolean _t =
item.wealth == 4
;
      if ( _t ) {
        _cnt++;
        _value +=
item.uDiff
;
      }
    }
    return _cnt > 0 ? _value / _cnt : 0;
  }
  /**
   * <i>This method should not be called by user</i>
   */
  private double _people_uDiffRichest_xjal() {
    int _cnt = 0;
    double _value = 0;
    for ( Person item : people ) {
      boolean _t =
item.wealth == 5
;
      if ( _t ) {
        _cnt++;
        _value +=
item.uDiff
;
      }
    }
    return _cnt > 0 ? _value / _cnt : 0;
  }
  /**
   * <i>This method should not be called by user</i>
   */
  private int _people_dist60km_xjal() {
    int _value = 0;
    for ( Person item : people ) {
      boolean _t =
item.travelDistance >= 60
;
      if ( _t ) {
        _value++;
      }
    }
    return _value;
  }
  /**
   * <i>This method should not be called by user</i>
   */
  private int _people_distFurther_xjal() {
    int _value = 0;
    for ( Person item : people ) {
```

```
      boolean _t =
item.distanceFurther
;
      if ( _t ) {
        _value++;
      }
    }
    return _value;
  }
  /**
   * <i>This method should not be called by user</i>
   */
  private double _people_mortPoorest_xjal() {
    double _value = 0;
    for ( Person item : people ) {
      boolean _t =
item.wealth == 1
;
      if ( _t ) {
        _value +=
item.deadBaby
;
      }
    }
    return _value;
  }
  /**
   * <i>This method should not be called by user</i>
   */
  private double _people_mortPoor_xjal() {
    double _value = 0;
    for ( Person item : people ) {
      boolean _t =
item.wealth == 2
;
      if ( _t ) {
        _value +=
item.deadBaby
;
      }
    }
    return _value;
  }
  /**
   * <i>This method should not be called by user</i>
   */
  private double _people_mortMiddle_xjal() {
    double _value = 0;
    for ( Person item : people ) {
      boolean _t =
item.wealth == 3
;
      if ( _t ) {
        _value +=
item.deadBaby
;
      }
    }
    return _value;
  }
  /**
   * <i>This method should not be called by user</i>
   */
```

```
  private double _people_mortRich_xjal() {
    double _value = 0;
    for ( Person item : people ) {
      boolean _t =
item.wealth == 4
;
      if ( _t ) {
        _value +=
item.deadBaby
;
      }
    }
    return _value;
  }
  /**
   * <i>This method should not be called by user</i>
   */
  private double _people_mortRichest_xjal() {
    double _value = 0;
    for ( Person item : people ) {
      boolean _t =
item.wealth == 5
;
      if ( _t ) {
        _value +=
item.deadBaby
;
      }
    }
    return _value;
  }
  /**
   * <i>This method should not be called by user</i>
   */
  private int _people_numCS_xjal() {
    int _value = 0;
    for ( Person item : people ) {
      boolean _t =
item.csPerformed
;
      if ( _t ) {
        _value++;
      }
    }
    return _value;
  }
  /**
   * <i>This method should not be called by user</i>
   */
  private double _people_totalCost_xjal() {
    double _value = 0;
    for ( Person item : people ) {
        _value +=
item.costPaid
;
    }
    return _value;
  }
  /**
   * <i>This method should not be called by user</i>
   */
  private double _people_catExpPoorest_xjal() {
    double _value = 0;
```

```
    for ( Person item : people ) {
      boolean _t = 
item.wealth == 1
;
      if ( _t ) {
        _value += 
item.catExp
;
      }
    }
    return _value;
  }
  /**
   * <i>This method should not be called by user</i>
   */
  private double _people_catExpPoor_xjal() {
    double _value = 0;
    for ( Person item : people ) {
      boolean _t = 
item.wealth == 2
;
      if ( _t ) {
        _value += 
item.catExp
;
      }
    }
    return _value;
  }
  /**
   * <i>This method should not be called by user</i>
   */
  private double _people_catExpMiddle_xjal() {
    double _value = 0;
    for ( Person item : people ) {
      boolean _t = 
item.wealth == 3
;
      if ( _t ) {
        _value += 
item.catExp
;
      }
    }
    return _value;
  }
  /**
   * <i>This method should not be called by user</i>
   */
  private double _people_catExpRich_xjal() {
    double _value = 0;
    for ( Person item : people ) {
      boolean _t = 
item.wealth == 4
;
      if ( _t ) {
        _value += 
item.catExp
;
      }
    }
    return _value;
  }
```

```
  /**
   * <i>This method should not be called by user</i>
   */
  private double _people_catExpRichest_xjal() {
    double _value = 0;
    for ( Person item : people ) {
      boolean _t =
item.wealth == 5
;
      if ( _t ) {
        _value +=
item.catExp
;
      }
    }
    return _value;
  }
  // Analysis Data Elements
  public HistogramSmartData uDiff = new HistogramSmartData( 50,
0.1
, true, true, 0.05, 0.05 );

  public HistogramSmartData travelDistance = new HistogramSmartData( 50,
0.1
, true, true, 0.05, 0.05 );

  public HistogramSmartData selectedBobs = new HistogramSmartData( 50,
0.1
, true, true, 0.05, 0.05 );

  public double travelTime;
  public void update_travelTime() {
    travelTime =
(double)people.travelTime() / (double)people.size() ;
  }
  public void update_travelTime(double _value) {
    travelTime = _value;
  }

  public int popSize;
  public void update_popSize() {
    popSize =
people.size() ;
  }
  public void update_popSize(int _value) {
    popSize = _value;
  }

  public double segment2;
  public void update_segment2() {
    segment2 =
(double) people.nClass2() / (double) people.size() ;
  }
  public void update_segment2(double _value) {
    segment2 = _value;
  }

  public double urbanPct;
  public void update_urbanPct() {
    urbanPct =
(double) people.nUrban() / (double) people.size() ;
  }
  public void update_urbanPct(double _value) {
```

```
    urbanPct = _value;
  }

  public double utilityDifference;
  public void update_utilityDifference() {
    utilityDifference =
this.uDiff.mean() ;
  }
  public void update_utilityDifference(double _value) {
    utilityDifference = _value;
  }

  public int classAssigned;
  public void update_classAssigned() {
    classAssigned =
people.classAssigned() ;
  }
  public void update_classAssigned(int _value) {
    classAssigned = _value;
  }

  public double avgBobs;
  public void update_avgBobs() {
    avgBobs =
hospitals.hospitalBobs() ;
  }
  public void update_avgBobs(double _value) {
    avgBobs = _value;
  }

  public double avgFees;
  public void update_avgFees() {
    avgFees =
hospitals.hospitalFees() ;
  }
  public void update_avgFees(double _value) {
    avgFees = _value;
  }

  public int hospitalAssigned;
  public void update_hospitalAssigned() {
    hospitalAssigned =
people.hospitalAssigned() ;
  }
  public void update_hospitalAssigned(int _value) {
    hospitalAssigned = _value;
  }

  public double meanDistance;
  public void update_meanDistance() {
    meanDistance =
this.travelDistance.mean() ;
  }
  public void update_meanDistance(double _value) {
    meanDistance = _value;
  }

  public double dist60km;
  public void update_dist60km() {
    dist60km =
(double)people.dist60km() / (double)people.size() ;
  }
  public void update_dist60km(double _value) {
```

```
    dist60km = _value;
  }

  public double distFurther;
  public void update_distFurther() {
    distFurther =
(double)people.distFurther() / (double)people.size() ;
  }
  public void update_distFurther(double _value) {
    distFurther = _value;
  }

  public double meanBobs;
  public void update_meanBobs() {
    meanBobs =
this.selectedBobs.mean() ;
  }
  public void update_meanBobs(double _value) {
    meanBobs = _value;
  }

  public double neonatalMortality;
  public void update_neonatalMortality() {
    neonatalMortality =
(double) (people.mortPoorest() + people.mortPoor() + people.mortMiddle() +
people.mortRich() + people.mortRichest()) / (double) people.size() ;
  }
  public void update_neonatalMortality(double _value) {
    neonatalMortality = _value;
  }

  public double csRate;
  public void update_csRate() {
    csRate =
(double) people.numCS() / (double) people.size() ;
  }
  public void update_csRate(double _value) {
    csRate = _value;
  }

  public double avgOOP;
  public void update_avgOOP() {
    avgOOP =
(double) people.totalCost() / (double) people.size() ;
  }
  public void update_avgOOP(double _value) {
    avgOOP = _value;
  }

  public double catExpPoorest;
  public void update_catExpPoorest() {
    catExpPoorest =
(double) people.catExpPoorest() / (double) people.nPoorest() ;
  }
  public void update_catExpPoorest(double _value) {
    catExpPoorest = _value;
  }

  public double catExpTotal;
  public void update_catExpTotal() {
    catExpTotal =
(double) (people.catExpPoorest() + people.catExpPoor() + people.catExpMiddle() +
people.catExpRich() + people.catExpRichest()) / (double) people.size() ;
```

```
  }
  public void update_catExpTotal(double _value) {
    catExpTotal = _value;
  }

  public double catExpRichest;
  public void update_catExpRichest() {
    catExpRichest =
(double) people.catExpRichest() / (double) people.nRichest() ;
  }
  public void update_catExpRichest(double _value) {
    catExpRichest = _value;
  }

  public double catExpRich;
  public void update_catExpRich() {
    catExpRich =
(double) people.catExpRich() / (double) people.nRich() ;
  }
  public void update_catExpRich(double _value) {
    catExpRich = _value;
  }

  public double catExpMiddle;
  public void update_catExpMiddle() {
    catExpMiddle =
(double) people.catExpMiddle() / (double) people.nMiddle() ;
  }
  public void update_catExpMiddle(double _value) {
    catExpMiddle = _value;
  }

  public double catExpPoor;
  public void update_catExpPoor() {
    catExpPoor =
(double) people.catExpPoor() / (double) people.nPoor() ;
  }
  public void update_catExpPoor(double _value) {
    catExpPoor = _value;
  }

  // View areas
  public ViewArea _origin_VA = new ViewArea( this, "[Origin]", 0, 0, 1550.0, 890.0 );
  @Override
  @AnyLogicInternalCodegenAPI
  public int getViewAreas(Map<String, ViewArea> _output) {
    if ( _output != null ) {
      _output.put( "_origin_VA", this._origin_VA );
    }
    return 1 + super.getViewAreas( _output );
  }
  @AnyLogicInternalCodegenAPI
  protected static final Font _agentID_Font = new Font("SansSerif", 0, 30 );
  @AnyLogicInternalCodegenAPI
  protected static final Font _delLocText_Font = new Font("Serif", 0, 18 );
  @AnyLogicInternalCodegenAPI
  protected static final Font _text2_Font = _delLocText_Font;
  @AnyLogicInternalCodegenAPI
  protected static final Font _text3_Font = _delLocText_Font;
  @AnyLogicInternalCodegenAPI
  protected static final Font _mapLayer_Font = _agentID_Font;
  @AnyLogicInternalCodegenAPI
  protected static final Font _text4_Font = new Font("Serif", 0, 12 );
```

```
@AnyLogicInternalCodegenAPI
protected static final Font _text5_Font = _text4_Font;
@AnyLogicInternalCodegenAPI
protected static final Font _delLocText1_Font = _delLocText_Font;
@AnyLogicInternalCodegenAPI
protected static final Font _travelDistText_Font = _delLocText_Font;
@AnyLogicInternalCodegenAPI
protected static final Font _text6_Font = _delLocText_Font;
@AnyLogicInternalCodegenAPI
protected static final Font _text7_Font = _delLocText_Font;
@AnyLogicInternalCodegenAPI
protected static final Font _text8_Font = _text4_Font;
@AnyLogicInternalCodegenAPI
protected static final Font _text9_Font = _text4_Font;
@AnyLogicInternalCodegenAPI
protected static final Font _StdErrorText_Font = new Font("Serif", 0, 30 );
@AnyLogicInternalCodegenAPI
protected static final Font _MeanText1_Font = _StdErrorText_Font;
@AnyLogicInternalCodegenAPI
protected static final Font _text10_Font = _delLocText_Font;
@AnyLogicInternalCodegenAPI
protected static final Font _text11_Font = _delLocText_Font;
@AnyLogicInternalCodegenAPI
protected static final Font _MeanText2_Font = _StdErrorText_Font;
@AnyLogicInternalCodegenAPI
protected static final Font _travelDistText1_Font = _delLocText_Font;
@AnyLogicInternalCodegenAPI
protected static final Font _StdErrorText1_Font = _StdErrorText_Font;
@AnyLogicInternalCodegenAPI
protected static final Font _text12_Font = _delLocText_Font;
@AnyLogicInternalCodegenAPI
protected static final Font _text13_Font = _delLocText_Font;
@AnyLogicInternalCodegenAPI
protected static final Font _delLocText2_Font = _delLocText_Font;
@AnyLogicInternalCodegenAPI
protected static final Font _text14_Font = _delLocText_Font;
@AnyLogicInternalCodegenAPI
protected static final Font _text15_Font = _delLocText_Font;
@AnyLogicInternalCodegenAPI
protected static final Font _text_Font = new Font("SansSerif", 0, 10 );
@AnyLogicInternalCodegenAPI
protected static final int _rectangle1 = 1;
@AnyLogicInternalCodegenAPI
protected static final int _rectangle = 2;
@AnyLogicInternalCodegenAPI
protected static final int _map = 3;
@AnyLogicInternalCodegenAPI
protected static final int _hospitals_presentation = 4;
@AnyLogicInternalCodegenAPI
protected static final int _people_presentation = 5;
@AnyLogicInternalCodegenAPI
protected static final int _agentID = 6;
@AnyLogicInternalCodegenAPI
protected static final int _delLocText = 7;
@AnyLogicInternalCodegenAPI
protected static final int _text2 = 8;
@AnyLogicInternalCodegenAPI
protected static final int _text3 = 9;
@AnyLogicInternalCodegenAPI
protected static final int _mapLayer = 10;
@AnyLogicInternalCodegenAPI
protected static final int _text4 = 11;
@AnyLogicInternalCodegenAPI
```

```
protected static final int _text5 = 12;
@AnyLogicInternalCodegenAPI
protected static final int _delLocText1 = 13;
@AnyLogicInternalCodegenAPI
protected static final int _travelDistText = 14;
@AnyLogicInternalCodegenAPI
protected static final int _text6 = 15;
@AnyLogicInternalCodegenAPI
protected static final int _text7 = 16;
@AnyLogicInternalCodegenAPI
protected static final int _text8 = 17;
@AnyLogicInternalCodegenAPI
protected static final int _text9 = 18;
@AnyLogicInternalCodegenAPI
protected static final int _StdErrorText = 19;
@AnyLogicInternalCodegenAPI
protected static final int _MeanText1 = 20;
@AnyLogicInternalCodegenAPI
protected static final int _rectangle2 = 21;
@AnyLogicInternalCodegenAPI
protected static final int _text10 = 22;
@AnyLogicInternalCodegenAPI
protected static final int _text11 = 23;
@AnyLogicInternalCodegenAPI
protected static final int _MeanText2 = 24;
@AnyLogicInternalCodegenAPI
protected static final int _travelDistText1 = 25;
@AnyLogicInternalCodegenAPI
protected static final int _rectangle3 = 26;
@AnyLogicInternalCodegenAPI
protected static final int _StdErrorText1 = 27;
@AnyLogicInternalCodegenAPI
protected static final int _text12 = 28;
@AnyLogicInternalCodegenAPI
protected static final int _text13 = 29;
@AnyLogicInternalCodegenAPI
protected static final int _delLocText2 = 30;
@AnyLogicInternalCodegenAPI
protected static final int _text14 = 31;
@AnyLogicInternalCodegenAPI
protected static final int _text15 = 32;
@AnyLogicInternalCodegenAPI
protected static final int _rectangle4 = 33;
@AnyLogicInternalCodegenAPI
protected static final int _text = 34;
@AnyLogicInternalCodegenAPI
protected static final int _chart = 35;
@AnyLogicInternalCodegenAPI
protected static final int _uDiffHistogram = 36;
@AnyLogicInternalCodegenAPI
protected static final int _uDiffHistogram1 = 37;
@AnyLogicInternalCodegenAPI
protected static final int _selectedBobsHist = 38;
@AnyLogicInternalCodegenAPI
protected static final int _mortChart = 39;
@AnyLogicInternalCodegenAPI
protected static final int _Blantyre = 40;
@AnyLogicInternalCodegenAPI
protected static final int _Lilongwe = 41;

/** Internal constant, shouldn't be accessed by user */
@AnyLogicInternalCodegenAPI
protected static final int _SHAPE_NEXT_ID_xjal = 42;
```

```
  /**
   * Top-level presentation group id
   */
  @AnyLogicInternalCodegenAPI
  protected static final int _presentation = 0;

  @AnyLogicInternalCodegenAPI
  public boolean isPublicPresentationDefined() {
    return true;
  }

  @AnyLogicInternalCodegenAPI
  public boolean isEmbeddedAgentPresentationVisible( Agent _a ) {
    return super.isEmbeddedAgentPresentationVisible( _a );
  }
  /**
   * Top-level icon group id
   */
  @AnyLogicInternalCodegenAPI
  protected static final int _icon = -1;



  /**
   * <i>This method should not be called by user</i>
   */
  @AnyLogicInternalCodegenAPI
  private double _chart_DataItem0Value() {
    return
people.nCentral()
;
  }

  /**
   * <i>This method should not be called by user</i>
   */
  @AnyLogicInternalCodegenAPI
  private double _chart_DataItem1Value() {
    return
people.nDistrict()
;
  }

  /**
   * <i>This method should not be called by user</i>
   */
  @AnyLogicInternalCodegenAPI
  private double _chart_DataItem2Value() {
    return
people.nCommunity()
;
  }

  /**
   * <i>This method should not be called by user</i>
   */
  @AnyLogicInternalCodegenAPI
  private double _chart_DataItem3Value() {
    return
people.nOther()
;
```

```
  }

  /**
   * <i>This method should not be called by user</i>
   */
  @AnyLogicInternalCodegenAPI
  private double _chart_DataItem4Value() {
    return
people.nHealthCenter()
;
  }

  /**
   * <i>This method should not be called by user</i>
   */
  @AnyLogicInternalCodegenAPI
  private double _chart_DataItem5Value() {
    return
people.nMaternity()
;
  }

  /**
   * <i>This method should not be called by user</i>
   */
  @AnyLogicInternalCodegenAPI
  private double _chart_DataItem6Value() {
    return
people.nClinic()
;
  }

  /**
   * <i>This method should not be called by user</i>
   */
  @AnyLogicInternalCodegenAPI
  private double _mortChart_DataItem0Value() {
    return
(double) people.mortPoorest() / (double) people.nPoorest()
;
  }

  /**
   * <i>This method should not be called by user</i>
   */
  @AnyLogicInternalCodegenAPI
  private double _mortChart_DataItem1Value() {
    return
(double)people.mortPoor() / (double) people.nPoor()
;
  }

  /**
   * <i>This method should not be called by user</i>
   */
  @AnyLogicInternalCodegenAPI
  private double _mortChart_DataItem2Value() {
    return
(double)people.mortMiddle() / (double)people.nMiddle()
;
  }

  /**
```

```
   * <i>This method should not be called by user</i>
   */
  @AnyLogicInternalCodegenAPI
  private double _mortChart_DataItem3Value() {
    return 
(double)people.mortRich() / (double) people.nRich()
;
  }

  /**
   * <i>This method should not be called by user</i>
   */
  @AnyLogicInternalCodegenAPI
  private double _mortChart_DataItem4Value() {
    return 
(double)people.mortRichest() / (double)people.nRichest()
;
  }
  protected BarChart chart;
  protected Histogram uDiffHistogram;
  protected Histogram uDiffHistogram1;
  protected Histogram selectedBobsHist;
  protected BarChart mortChart;
  protected ShapeRectangle rectangle1;
  protected ShapeRectangle rectangle;
  protected ShapeGISMap map;

  /**
   * <i>This method should not be called by user</i>
   */
  @AnyLogicInternalCodegenAPI
  protected ShapeEmbeddedObjectPresentation 
_hospitals_presentation_createShapeWithStaticProperties_xjal( final Agent _a, final 
int _index ) {
    ShapeEmbeddedObjectPresentation shape = new ShapeEmbeddedObjectPresentation( 
Main.this, SHAPE_DRAW_2D3D, true, -13.277582231456787, 35.06144928525293, 0.0, 0.0,
             false, true, _a );

    return shape;
  }

  protected ShapeAgentPopulationGroup hospitals_presentation;

  /**
   * <i>This method should not be called by user</i>
   */
  @AnyLogicInternalCodegenAPI
  protected ShapeEmbeddedObjectPresentation 
_people_presentation_createShapeWithStaticProperties_xjal( final Agent _a, final int 
_index ) {
    ShapeEmbeddedObjectPresentation shape = new ShapeEmbeddedObjectPresentation( 
Main.this, SHAPE_DRAW_2D3D, true, -13.277582231456787, 35.06144928525293, 0.0, 0.0,
             false, true, _a );

    return shape;
  }

  protected ShapeAgentPopulationGroup people_presentation;

  /**
   * <i>This method should not be called by user</i>
   */
  @AnyLogicInternalCodegenAPI
```

```
  private void _agentID_SetDynamicParams_xjal( ShapeText shape ) {
    boolean _visible =
false
;
    shape.setVisible( _visible );
      if ( _visible ) {
    shape.setText(
bobs2
);
      }
  }

  protected ShapeText agentID;
  protected ShapeText delLocText;
  protected ShapeText text2;
  protected ShapeText text3;

  /**
   * <i>This method should not be called by user</i>
   */
  @AnyLogicInternalCodegenAPI
  private void _mapLayer_SetDynamicParams_xjal( ShapeText shape ) {
    boolean _visible =
false
;
    shape.setVisible( _visible );
      if ( _visible ) {
    shape.setText(
map.getLayers().toString()
);
      }
  }

  protected ShapeText mapLayer;
  protected ShapeText text4;
  protected ShapeText text5;
  protected ShapeText delLocText1;
  protected ShapeText travelDistText;
  protected ShapeText text6;
  protected ShapeText text7;
  protected ShapeText text8;
  protected ShapeText text9;
  protected ShapeText StdErrorText;
  protected ShapeText MeanText1;
  protected ShapeRectangle rectangle2;
  protected ShapeText text10;
  protected ShapeText text11;
  protected ShapeText MeanText2;
  protected ShapeText travelDistText1;
  protected ShapeRectangle rectangle3;
  protected ShapeText StdErrorText1;
  protected ShapeText text12;
  protected ShapeText text13;
  protected ShapeText delLocText2;
  protected ShapeText text14;
  protected ShapeText text15;
  protected ShapeRectangle rectangle4;
  protected ShapeText text;
  protected GISRegion Blantyre;
  protected GISRegion Lilongwe;
  @AnyLogicInternalCodegenAPI
  private void _createPersistentElementsBP0_xjal() {
    rectangle1 = new ShapeRectangle(
```

```
   SHAPE_DRAW_2D3D, true,-840.0, 10.0, 0.0, 0.0,
        black, white,
                450.0, 380.0, 10.0, 1.0, LINE_STYLE_SOLID );

rectangle = new ShapeRectangle(
   SHAPE_DRAW_2D3D, true,-1310.0, 10.0, 0.0, 0.0,
        black, white,
                450.0, 380.0, 10.0, 1.0, LINE_STYLE_SOLID );

agentID = new ShapeText(
    SHAPE_DRAW_2D, true,40.0, 20.0, 0.0, 0.0,
    black,"Agent ID",
    _agentID_Font, ALIGNMENT_LEFT ) {
  @Override
  public void updateDynamicProperties(boolean publicOnly) {
    _agentID_SetDynamicParams_xjal( this );
    super.updateDynamicProperties(publicOnly);
  }
};

delLocText = new ShapeText(
    SHAPE_DRAW_2D, true,740.0, 150.0, 0.0, 0.0,
    black,"Delivery location",
    _delLocText_Font, ALIGNMENT_LEFT );

text2 = new ShapeText(
    SHAPE_DRAW_2D, true,-820.0, 70.0, 0.0, 0.0,
    black,"Lower model",
    _text2_Font, ALIGNMENT_LEFT );

text3 = new ShapeText(
    SHAPE_DRAW_2D, true,-560.0, 70.0, 0.0, 0.0,
    black,"Class Assignment",
    _text3_Font, ALIGNMENT_LEFT );

mapLayer = new ShapeText(
    SHAPE_DRAW_2D, true,40.0, 70.0, 0.0, 0.0,
    black,"Agent ID",
    _mapLayer_Font, ALIGNMENT_LEFT ) {
  @Override
  public void updateDynamicProperties(boolean publicOnly) {
    _mapLayer_SetDynamicParams_xjal( this );
    super.updateDynamicProperties(publicOnly);
  }
};

mapLayer.setVisible( false );
text4 = new ShapeText(
    SHAPE_DRAW_2D, true,-820.0, 100.0, 0.0, 0.0,
    black,"Class 1",
    _text4_Font, ALIGNMENT_LEFT );

text5 = new ShapeText(
    SHAPE_DRAW_2D, true,-700.0, 100.0, 0.0, 0.0,
    black,"Class 2",
    _text5_Font, ALIGNMENT_LEFT );

delLocText1 = new ShapeText(
    SHAPE_DRAW_2D, true,1110.0, 610.0, 0.0, 0.0,
    black,"Utility difference (millions)\t",
    _delLocText1_Font, ALIGNMENT_LEFT );

travelDistText = new ShapeText(
```

```
    SHAPE_DRAW_2D, true,1130.0, 380.0, 0.0, 0.0,
    black,"Travel distance",
    _travelDistText_Font, ALIGNMENT_LEFT );

text6 = new ShapeText(
    SHAPE_DRAW_2D, true,-1300.0, 70.0, 0.0, 0.0,
    black,"Lower model",
    _text6_Font, ALIGNMENT_LEFT );

text7 = new ShapeText(
    SHAPE_DRAW_2D, true,-1040.0, 70.0, 0.0, 0.0,
    black,"Class Assignment",
    _text7_Font, ALIGNMENT_LEFT );

text8 = new ShapeText(
    SHAPE_DRAW_2D, true,-1300.0, 100.0, 0.0, 0.0,
    black,"Class 1",
    _text8_Font, ALIGNMENT_LEFT );

text9 = new ShapeText(
    SHAPE_DRAW_2D, true,-1180.0, 100.0, 0.0, 0.0,
    black,"Class 2",
    _text9_Font, ALIGNMENT_LEFT );

StdErrorText = new ShapeText(
    SHAPE_DRAW_2D, true,-1300.0, 30.0, 0.0, 0.0,
    black,"Standard Errors",
    _StdErrorText_Font, ALIGNMENT_LEFT );

MeanText1 = new ShapeText(
    SHAPE_DRAW_2D, true,-830.0, 30.0, 0.0, 0.0,
    black,"Means",
    _MeanText1_Font, ALIGNMENT_LEFT );

rectangle2 = new ShapeRectangle(
   SHAPE_DRAW_2D3D, true,-840.0, 400.0, 0.0, 0.0,
        black, white,
                450.0, 380.0, 10.0, 1.0, LINE_STYLE_SOLID );

text10 = new ShapeText(
    SHAPE_DRAW_2D, true,-820.0, 460.0, 0.0, 0.0,
    black,"Lower model",
    _text10_Font, ALIGNMENT_LEFT );

text11 = new ShapeText(
    SHAPE_DRAW_2D, true,-570.0, 460.0, 0.0, 0.0,
    black,"Class Assignment",
    _text11_Font, ALIGNMENT_LEFT );

MeanText2 = new ShapeText(
    SHAPE_DRAW_2D, true,-830.0, 420.0, 0.0, 0.0,
    black,"Instantiation",
    _MeanText2_Font, ALIGNMENT_LEFT );

travelDistText1 = new ShapeText(
    SHAPE_DRAW_2D, true,1110.0, 150.0, 0.0, 0.0,
    black,"Bobs",
    _travelDistText1_Font, ALIGNMENT_LEFT );

rectangle3 = new ShapeRectangle(
   SHAPE_DRAW_2D3D, true,-1760.0, 400.0, 0.0, 0.0,
        black, white,
                900.0, 380.0, 10.0, 1.0, LINE_STYLE_SOLID );
```

```
    StdErrorText1 = new ShapeText(
        SHAPE_DRAW_2D, true,-1740.0, 420.0, 0.0, 0.0,
        black,"Neonatal Mortality",
        _StdErrorText1_Font, ALIGNMENT_LEFT );

    text12 = new ShapeText(
        SHAPE_DRAW_2D, true,-1300.0, 460.0, 0.0, 0.0,
        black,"With CS",
        _text12_Font, ALIGNMENT_LEFT );

    text13 = new ShapeText(
        SHAPE_DRAW_2D, true,-1050.0, 460.0, 0.0, 0.0,
        black,"No CS",
        _text13_Font, ALIGNMENT_LEFT );

    delLocText2 = new ShapeText(
        SHAPE_DRAW_2D, true,740.0, 520.0, 0.0, 0.0,
        black,"Neonatal mortality",
        _delLocText2_Font, ALIGNMENT_LEFT );

    text14 = new ShapeText(
        SHAPE_DRAW_2D, true,-1710.0, 460.0, 0.0, 0.0,
        black,"With BEMC5",
        _text14_Font, ALIGNMENT_LEFT );

    text15 = new ShapeText(
        SHAPE_DRAW_2D, true,-1510.0, 460.0, 0.0, 0.0,
        black,"No BEMC5",
        _text15_Font, ALIGNMENT_LEFT );

    rectangle4 = new ShapeRectangle(
       SHAPE_DRAW_2D3D, true,-1040.0, 340.0, 0.0, 0.0,
            gold, yellow,
                    160.0, 40.0, 10.0, 1.0, LINE_STYLE_SOLID );

    text = new ShapeText(
        SHAPE_DRAW_2D, true,-1030.0, 342.0, 0.0, 0.0,
        black,"Note: wealth, blind, illliterate\nand primip are declared in\nthe
onStartup() of Main",
        _text_Font, ALIGNMENT_LEFT );

    Blantyre = new GISRegion( map, true, this.<double[]>getElementProperty("Blantyre",
IElementDescriptor.LAT_LON_PAIRS), defaultGisFillColor, brown, 1.0, LINE_STYLE_DASHED,
"Blantyre, Southern Region, Malawi", 2.3209617548125E9 );

    Lilongwe = new GISRegion( map, true, this.<double[]>getElementProperty("Lilongwe",
IElementDescriptor.LAT_LON_PAIRS), defaultGisFillColor, brown, 1.0, LINE_STYLE_DASHED,
"Lilongwe, Central Region, Malawi", 6.205527466875E9 );

  }

  @AnyLogicInternalCodegenAPI
  private void _createPersistentElementsAP0_xjal() {
    {
    List<DataItem> _items = new ArrayList<DataItem>( 7 );
    _items.add( new DataItem() {
      @Override
      public void update() {
        setValue( _chart_DataItem0Value() );
      }
    });
    _items.add( new DataItem() {
```

```
  @Override
  public void update() {
    setValue( _chart_DataItem1Value() );
  }
});
_items.add( new DataItem() {
  @Override
  public void update() {
    setValue( _chart_DataItem2Value() );
  }
});
_items.add( new DataItem() {
  @Override
  public void update() {
    setValue( _chart_DataItem3Value() );
  }
});
_items.add( new DataItem() {
  @Override
  public void update() {
    setValue( _chart_DataItem4Value() );
  }
});
_items.add( new DataItem() {
  @Override
  public void update() {
    setValue( _chart_DataItem5Value() );
  }
});
_items.add( new DataItem() {
  @Override
  public void update() {
    setValue( _chart_DataItem6Value() );
  }
});
List<String> _titles = new ArrayList<String>( 7 );
_titles.add( "Central Hospital" );
_titles.add( "District Hospital" );
_titles.add( "Community Hospital" );
_titles.add( "Other Hospital" );
_titles.add( "Health Center" );
_titles.add( "Maternity" );
_titles.add( "Clinic" );
List<Color> _colors = new ArrayList<Color>( 7 );
_colors.add( red );
_colors.add( blue );
_colors.add( coral );
_colors.add( yellow );
_colors.add( silver );
_colors.add( magenta );
_colors.add( green );
chart = new BarChart(
             Main.this, true, 750.0, 150.0,
                310.0, 360.0,
        null, null,
        50.0, 30.0,
                230.0, 150.0, white, black, black,
        150.0, Chart.SOUTH,
        Chart.NORTH, Chart.SCALE_100_PERCENT,
        0, 0, 0.8,
        Chart.GRID_DEFAULT,
        darkGray, darkGray, _items, _titles, _colors );
```

```
    }
    {
    HistogramData _item;
    List<HistogramData> _items = new ArrayList<HistogramData>( 1 );
    _item =
uDiff
;
    _items.add( _item );
    List<String> _titles = new ArrayList<String>( 1 );
    _titles.add( "Utility difference (optimal minus selected)" );
    List<Histogram.Appearance> _appearances = new ArrayList<Histogram.Appearance>( 1
);
    _appearances.add( new Histogram.Appearance( darkKhaki, darkKhaki, mediumSeaGreen,
coral, 1, lightSlateBlue ) );
    uDiffHistogram = new Histogram(
                 Main.this, true, 1100.0, 610.0,
                    370.0, 260.0,
            null, null,
            50.0, 30.0,
                    290.0, 170.0, white, black, black,
            30.0, Chart.SOUTH,
            Chart.GRID_DEFAULT, Chart.GRID_DEFAULT,
            darkGray, darkGray,
            true, true, true, 0.8,
            _items, _titles, _appearances );

    }
    {
    HistogramData _item;
    List<HistogramData> _items = new ArrayList<HistogramData>( 1 );
    _item =
travelDistance
;
    _items.add( _item );
    List<String> _titles = new ArrayList<String>( 1 );
    _titles.add( "Travel distance (km)" );
    List<Histogram.Appearance> _appearances = new ArrayList<Histogram.Appearance>( 1
);
    _appearances.add( new Histogram.Appearance( peru, peru, brown, navy, 1,
lightSlateBlue ) );
    uDiffHistogram1 = new Histogram(
                 Main.this, true, 1100.0, 380.0,
                    370.0, 230.0,
            null, null,
            50.0, 30.0,
                    290.0, 140.0, white, black, black,
            30.0, Chart.SOUTH,
            Chart.GRID_DEFAULT, Chart.GRID_DEFAULT,
            darkGray, darkGray,
            true, true, true, 0.8,
            _items, _titles, _appearances );

    }
    {
    HistogramData _item;
    List<HistogramData> _items = new ArrayList<HistogramData>( 1 );
    _item =
selectedBobs
;
    _items.add( _item );
    List<String> _titles = new ArrayList<String>( 1 );
    _titles.add( "Hospital basic obstetric readiness" );
```

```
    List<Histogram.Appearance> _appearances = new ArrayList<Histogram.Appearance>( 1
);
    _appearances.add( new Histogram.Appearance( navy, navy, skyBlue, maroon, 1,
lightSlateBlue ) );
    selectedBobsHist = new Histogram(
                 Main.this, true, 1070.0, 150.0,
                    370.0, 230.0,
            null, null,
            50.0, 30.0,
                    290.0, 140.0, white, black, black,
            30.0, Chart.SOUTH,
            Chart.GRID_DEFAULT, Chart.GRID_DEFAULT,
            darkGray, darkGray,
            true, true, true, 0.8,
            _items, _titles, _appearances );

    }
    {
    List<DataItem> _items = new ArrayList<DataItem>( 5 );
    _items.add( new DataItem() {
      @Override
      public void update() {
        setValue( _mortChart_DataItem0Value() );
      }
    });
    _items.add( new DataItem() {
      @Override
      public void update() {
        setValue( _mortChart_DataItem1Value() );
      }
    });
    _items.add( new DataItem() {
      @Override
      public void update() {
        setValue( _mortChart_DataItem2Value() );
      }
    });
    _items.add( new DataItem() {
      @Override
      public void update() {
        setValue( _mortChart_DataItem3Value() );
      }
    });
    _items.add( new DataItem() {
      @Override
      public void update() {
        setValue( _mortChart_DataItem4Value() );
      }
    });
    List<String> _titles = new ArrayList<String>( 5 );
    _titles.add( "Poorest" );
    _titles.add( "Poor" );
    _titles.add( "Middle" );
    _titles.add( "Rich" );
    _titles.add( "Richest" );
    List<Color> _colors = new ArrayList<Color>( 5 );
    _colors.add( darkSalmon );
    _colors.add( lightCoral );
    _colors.add( indianRed );
    _colors.add( fireBrick );
    _colors.add( maroon );
    mortChart = new BarChart(
                 Main.this, true, 750.0, 520.0,
```

```
                310.0, 290.0,
            null, null,
            50.0, 30.0,
                230.0, 150.0, white, black, black,
            80.0, Chart.SOUTH,
            Chart.NORTH, Chart.SCALE_AUTO,
            0, 0, 0.8,
            Chart.GRID_DEFAULT,
            darkGray, darkGray, _items, _titles, _colors );

    }
    hospitals_presentation = new ShapeAgentPopulationGroup(this, SHAPE_DRAW_2D3D, 
true, hospitals) {

      @Override
      public ShapeEmbeddedObjectPresentation createShapeWithStaticProperties_xjal( 
final Agent _a, int index ) {
        ShapeEmbeddedObjectPresentation _e = 
_hospitals_presentation_createShapeWithStaticProperties_xjal( _a, index );
        return _e;
      }
    };
    people_presentation = new ShapeAgentPopulationGroup(this, SHAPE_DRAW_2D3D, true, 
people) {

      @Override
      public ShapeEmbeddedObjectPresentation createShapeWithStaticProperties_xjal( 
final Agent _a, int index ) {
        ShapeEmbeddedObjectPresentation _e = 
_people_presentation_createShapeWithStaticProperties_xjal( _a, index );
        return _e;
      }
    };
  }


  // Static initialization of persistent elements
  {
    map = new ShapeGISMap(
             Main.this, SHAPE_DRAW_2D3D, true, 0.0, 0.0,
                720.0, 780.0, "/malawi_recentralization_model_2017_06_04/", new 
ShapeGISMap.Layer[] {
             new ShapeGISMap.Layer( "Malawi_Population.shp", "Malawi_Population.dbf", 
black, white, 0, false ), }, -13.525957106075987, 34.78690304927513, 2.5E-7, silver, 
             white, true,
             TileURLProviderType.HUMANITARIAN,
             new GraphHopperRouteProvider("Routing Graph", "astarbi", 
RoutingMethod.FASTEST),
             1, false );

    _createPersistentElementsBP0_xjal();
  }
  protected ShapeTopLevelPresentationGroup presentation;
  protected ShapeModelElementsGroup icon;

  @Override
  @AnyLogicInternalCodegenAPI
  public ShapeTopLevelPresentationGroup getPresentationShape() {
    return presentation;
  }

  @Override
  @AnyLogicInternalCodegenAPI
```

```
  public ShapeModelElementsGroup getModelElementsShape() {
    return icon;
  }

  @Override
  @AnyLogicInternalCodegenAPI
  public Object getPersistentShape( int _shape ) {
    switch (_shape) {
      case _presentation: return presentation;
      case _icon: return icon;
      case _chart: return chart;
      case _uDiffHistogram: return uDiffHistogram;
      case _uDiffHistogram1: return uDiffHistogram1;
      case _selectedBobsHist: return selectedBobsHist;
      case _mortChart: return mortChart;
      case _rectangle1: return rectangle1;
      case _rectangle: return rectangle;
      case _map: return map;
      case _hospitals_presentation: return hospitals_presentation;
      case _people_presentation: return people_presentation;
      case _agentID: return agentID;
      case _delLocText: return delLocText;
      case _text2: return text2;
      case _text3: return text3;
      case _mapLayer: return mapLayer;
      case _text4: return text4;
      case _text5: return text5;
      case _delLocText1: return delLocText1;
      case _travelDistText: return travelDistText;
      case _text6: return text6;
      case _text7: return text7;
      case _text8: return text8;
      case _text9: return text9;
      case _StdErrorText: return StdErrorText;
      case _MeanText1: return MeanText1;
      case _rectangle2: return rectangle2;
      case _text10: return text10;
      case _text11: return text11;
      case _MeanText2: return MeanText2;
      case _travelDistText1: return travelDistText1;
      case _rectangle3: return rectangle3;
      case _StdErrorText1: return StdErrorText1;
      case _text12: return text12;
      case _text13: return text13;
      case _delLocText2: return delLocText2;
      case _text14: return text14;
      case _text15: return text15;
      case _rectangle4: return rectangle4;
      case _text: return text;
      case _Blantyre: return Blantyre;
      case _Lilongwe: return Lilongwe;
      default: return super.getPersistentShape( _shape );
    }
  }

  @Override
  @AnyLogicInternalCodegenAPI
  public String getNameOfShape_xjal( Object _shape ) {
    try {
      if ( _shape == null ) return null;
      String _name_xjal;
      _name_xjal = checkNameOfShape_xjal( _shape, presentation, "presentation" ); if
(_name_xjal != null) return _name_xjal;
```

```
      _name_xjal = checkNameOfShape_xjal( _shape, icon, "icon" ); if (_name_xjal !=
null) return _name_xjal;
      _name_xjal = checkNameOfShape_xjal( _shape, chart, "chart" ); if (_name_xjal !=
null) return _name_xjal;
      _name_xjal = checkNameOfShape_xjal( _shape, uDiffHistogram, "uDiffHistogram" );
if (_name_xjal != null) return _name_xjal;
      _name_xjal = checkNameOfShape_xjal( _shape, uDiffHistogram1, "uDiffHistogram1"
); if (_name_xjal != null) return _name_xjal;
      _name_xjal = checkNameOfShape_xjal( _shape, selectedBobsHist, "selectedBobsHist"
); if (_name_xjal != null) return _name_xjal;
      _name_xjal = checkNameOfShape_xjal( _shape, mortChart, "mortChart" ); if
(_name_xjal != null) return _name_xjal;
      _name_xjal = checkNameOfShape_xjal( _shape, rectangle1, "rectangle1" ); if
(_name_xjal != null) return _name_xjal;
      _name_xjal = checkNameOfShape_xjal( _shape, rectangle, "rectangle" ); if
(_name_xjal != null) return _name_xjal;
      _name_xjal = checkNameOfShape_xjal( _shape, map, "map" ); if (_name_xjal !=
null) return _name_xjal;
      _name_xjal = checkNameOfShape_xjal( _shape, hospitals_presentation,
"hospitals_presentation" ); if (_name_xjal != null) return _name_xjal;
      _name_xjal = checkNameOfShape_xjal( _shape, people_presentation,
"people_presentation" ); if (_name_xjal != null) return _name_xjal;
      _name_xjal = checkNameOfShape_xjal( _shape, agentID, "agentID" ); if (_name_xjal
!= null) return _name_xjal;
      _name_xjal = checkNameOfShape_xjal( _shape, delLocText, "delLocText" ); if
(_name_xjal != null) return _name_xjal;
      _name_xjal = checkNameOfShape_xjal( _shape, text2, "text2" ); if (_name_xjal !=
null) return _name_xjal;
      _name_xjal = checkNameOfShape_xjal( _shape, text3, "text3" ); if (_name_xjal !=
null) return _name_xjal;
      _name_xjal = checkNameOfShape_xjal( _shape, mapLayer, "mapLayer" ); if
(_name_xjal != null) return _name_xjal;
      _name_xjal = checkNameOfShape_xjal( _shape, text4, "text4" ); if (_name_xjal !=
null) return _name_xjal;
      _name_xjal = checkNameOfShape_xjal( _shape, text5, "text5" ); if (_name_xjal !=
null) return _name_xjal;
      _name_xjal = checkNameOfShape_xjal( _shape, delLocText1, "delLocText1" ); if
(_name_xjal != null) return _name_xjal;
      _name_xjal = checkNameOfShape_xjal( _shape, travelDistText, "travelDistText" );
if (_name_xjal != null) return _name_xjal;
      _name_xjal = checkNameOfShape_xjal( _shape, text6, "text6" ); if (_name_xjal !=
null) return _name_xjal;
      _name_xjal = checkNameOfShape_xjal( _shape, text7, "text7" ); if (_name_xjal !=
null) return _name_xjal;
      _name_xjal = checkNameOfShape_xjal( _shape, text8, "text8" ); if (_name_xjal !=
null) return _name_xjal;
      _name_xjal = checkNameOfShape_xjal( _shape, text9, "text9" ); if (_name_xjal !=
null) return _name_xjal;
      _name_xjal = checkNameOfShape_xjal( _shape, StdErrorText, "StdErrorText" ); if
(_name_xjal != null) return _name_xjal;
      _name_xjal = checkNameOfShape_xjal( _shape, MeanText1, "MeanText1" ); if
(_name_xjal != null) return _name_xjal;
      _name_xjal = checkNameOfShape_xjal( _shape, rectangle2, "rectangle2" ); if
(_name_xjal != null) return _name_xjal;
      _name_xjal = checkNameOfShape_xjal( _shape, text10, "text10" ); if (_name_xjal
!= null) return _name_xjal;
      _name_xjal = checkNameOfShape_xjal( _shape, text11, "text11" ); if (_name_xjal
!= null) return _name_xjal;
      _name_xjal = checkNameOfShape_xjal( _shape, MeanText2, "MeanText2" ); if
(_name_xjal != null) return _name_xjal;
      _name_xjal = checkNameOfShape_xjal( _shape, travelDistText1, "travelDistText1"
); if (_name_xjal != null) return _name_xjal;
```

```
      _name_xjal = checkNameOfShape_xjal( _shape, rectangle3, "rectangle3" ); if
(_name_xjal != null) return _name_xjal;
      _name_xjal = checkNameOfShape_xjal( _shape, StdErrorText1, "StdErrorText1" ); if
(_name_xjal != null) return _name_xjal;
      _name_xjal = checkNameOfShape_xjal( _shape, text12, "text12" ); if (_name_xjal
!= null) return _name_xjal;
      _name_xjal = checkNameOfShape_xjal( _shape, text13, "text13" ); if (_name_xjal
!= null) return _name_xjal;
      _name_xjal = checkNameOfShape_xjal( _shape, delLocText2, "delLocText2" ); if
(_name_xjal != null) return _name_xjal;
      _name_xjal = checkNameOfShape_xjal( _shape, text14, "text14" ); if (_name_xjal
!= null) return _name_xjal;
      _name_xjal = checkNameOfShape_xjal( _shape, text15, "text15" ); if (_name_xjal
!= null) return _name_xjal;
      _name_xjal = checkNameOfShape_xjal( _shape, rectangle4, "rectangle4" ); if
(_name_xjal != null) return _name_xjal;
      _name_xjal = checkNameOfShape_xjal( _shape, text, "text" ); if (_name_xjal !=
null) return _name_xjal;
      _name_xjal = checkNameOfShape_xjal( _shape, Blantyre, "Blantyre" ); if
(_name_xjal != null) return _name_xjal;
      _name_xjal = checkNameOfShape_xjal( _shape, Lilongwe, "Lilongwe" ); if
(_name_xjal != null) return _name_xjal;
    } catch (Exception e) {
      return null;
    }
    return super.getNameOfShape_xjal( _shape );
  }

  @AnyLogicInternalCodegenAPI
  private void drawModelElements_Connectivity_xjal(Panel _panel, Graphics2D _g,
boolean _publicOnly, boolean _isSuperClass ) {
    if (!_publicOnly) {
      drawExcelFile( _panel, _g, -160, 410, 10, 0, "distanceWTP", distanceWTP );
    }
    if (!_publicOnly) {
      drawExcelFile( _panel, _g, -150, 460, 10, 0, "newClassAssignment",
newClassAssignment );
    }
  }

  @AnyLogicInternalCodegenAPI
  private void drawModelElements_Events_xjal(Panel _panel, Graphics2D _g, boolean
_publicOnly, boolean _isSuperClass ) {
    if (!_publicOnly) {
      drawEvent( _panel, _g, -160, 360, 10, 0, "classesAssigned", classesAssigned );
    }
  }

  @AnyLogicInternalCodegenAPI
  private void drawModelElements_Parameters_xjal(Panel _panel, Graphics2D _g, boolean
_publicOnly, boolean _isSuperClass ) {
    if (!_publicOnly) {
      drawParameter( _panel, _g, -160, 190, 10, 0, "allowFeesParam", allowFeesParam, 0
);
    }
    if (!_publicOnly) {
      drawParameter( _panel, _g, -810, 120, 10, 0, "centralHosp1", centralHosp1, 0 );
    }
    if (!_publicOnly) {
      drawParameter( _panel, _g, -810, 310, 10, 0, "distance1", distance1, 0 );
    }
    if (!_publicOnly) {
      drawParameter( _panel, _g, -810, 240, 10, 0, "clinic1", clinic1, 0 );
```

```
    }
    if (!_publicOnly) {
      drawParameter( _panel, _g, -810, 220, 10, 0, "maternity1", maternity1, 0 );
    }
    if (!_publicOnly) {
      drawParameter( _panel, _g, -810, 200, 10, 0, "healthCenter1", healthCenter1, 0
);
    }
    if (!_publicOnly) {
      drawParameter( _panel, _g, -810, 180, 10, 0, "otherHosp1", otherHosp1, 0 );
    }
    if (!_publicOnly) {
      drawParameter( _panel, _g, -810, 160, 10, 0, "communityHosp1", communityHosp1, 0
);
    }
    if (!_publicOnly) {
      drawParameter( _panel, _g, -810, 140, 10, 0, "districtHosp1", districtHosp1, 0
);
    }
    if (!_publicOnly) {
      drawParameter( _panel, _g, -810, 370, 10, 0, "bobs1", bobs1, 0 );
    }
    if (!_publicOnly) {
      drawParameter( _panel, _g, -810, 350, 10, 0, "fees1", fees1, 0 );
    }
    if (!_publicOnly) {
      drawParameter( _panel, _g, -160, 220, 10, 0, "allowAllParam", allowAllParam, 0
);
    }
    if (!_publicOnly) {
      drawParameter( _panel, _g, -550, 100, 10, 0, "intercept", intercept, 0 );
    }
    if (!_publicOnly) {
      drawParameter( _panel, _g, -690, 120, 10, 0, "centralHosp2", centralHosp2, 0 );
    }
    if (!_publicOnly) {
      drawParameter( _panel, _g, -690, 310, 10, 0, "distance2", distance2, 0 );
    }
    if (!_publicOnly) {
      drawParameter( _panel, _g, -690, 240, 10, 0, "clinic2", clinic2, 0 );
    }
    if (!_publicOnly) {
      drawParameter( _panel, _g, -690, 220, 10, 0, "maternity2", maternity2, 0 );
    }
    if (!_publicOnly) {
      drawParameter( _panel, _g, -690, 200, 10, 0, "healthCenter2", healthCenter2, 0
);
    }
    if (!_publicOnly) {
      drawParameter( _panel, _g, -690, 180, 10, 0, "otherHosp2", otherHosp2, 0 );
    }
    if (!_publicOnly) {
      drawParameter( _panel, _g, -690, 160, 10, 0, "communityHosp2", communityHosp2, 0
);
    }
    if (!_publicOnly) {
      drawParameter( _panel, _g, -690, 140, 10, 0, "districtHosp2", districtHosp2, 0
);
    }
    if (!_publicOnly) {
      drawParameter( _panel, _g, -690, 370, 10, 0, "bobs2", bobs2, 0 );
    }
    if (!_publicOnly) {
```

```
      drawParameter( _panel, _g, -690, 350, 10, 0, "fees2", fees2, 0 );
    }
    if (!_publicOnly) {
      drawParameter( _panel, _g, -550, 350, 10, 0, "distCenter", distCenter, 0 );
    }
    if (!_publicOnly) {
      drawParameter( _panel, _g, -550, 370, 10, 0, "distRMS", distRMS, 0 );
    }
    if (!_publicOnly) {
      drawParameter( _panel, _g, -1290, 120, 10, 0, "centralHosp1SE", centralHosp1SE,
0 );
    }
    if (!_publicOnly) {
      drawParameter( _panel, _g, -1290, 310, 10, 0, "distance1SE", distance1SE, 0 );
    }
    if (!_publicOnly) {
      drawParameter( _panel, _g, -1290, 240, 10, 0, "clinic1SE", clinic1SE, 0 );
    }
    if (!_publicOnly) {
      drawParameter( _panel, _g, -1290, 220, 10, 0, "maternity1SE", maternity1SE, 0 );
    }
    if (!_publicOnly) {
      drawParameter( _panel, _g, -1290, 200, 10, 0, "healthCenter1SE",
healthCenter1SE, 0 );
    }
    if (!_publicOnly) {
      drawParameter( _panel, _g, -1290, 180, 10, 0, "otherHosp1SE", otherHosp1SE, 0 );
    }
    if (!_publicOnly) {
      drawParameter( _panel, _g, -1290, 160, 10, 0, "communityHosp1SE",
communityHosp1SE, 0 );
    }
    if (!_publicOnly) {
      drawParameter( _panel, _g, -1290, 140, 10, 0, "districtHosp1SE",
districtHosp1SE, 0 );
    }
    if (!_publicOnly) {
      drawParameter( _panel, _g, -1290, 370, 10, 0, "bobs1SE", bobs1SE, 0 );
    }
    if (!_publicOnly) {
      drawParameter( _panel, _g, -1290, 350, 10, 0, "fees1SE", fees1SE, 0 );
    }
    if (!_publicOnly) {
      drawParameter( _panel, _g, -1020, 100, 10, 0, "interceptSE", interceptSE, 0 );
    }
    if (!_publicOnly) {
      drawParameter( _panel, _g, -1170, 120, 10, 0, "centralHosp2SE", centralHosp2SE,
0 );
    }
    if (!_publicOnly) {
      drawParameter( _panel, _g, -1170, 310, 10, 0, "distance2SE", distance2SE, 0 );
    }
    if (!_publicOnly) {
      drawParameter( _panel, _g, -1170, 240, 10, 0, "clinic2SE", clinic2SE, 0 );
    }
    if (!_publicOnly) {
      drawParameter( _panel, _g, -1170, 220, 10, 0, "maternity2SE", maternity2SE, 0 );
    }
    if (!_publicOnly) {
      drawParameter( _panel, _g, -1170, 200, 10, 0, "healthCenter2SE",
healthCenter2SE, 0 );
    }
    if (!_publicOnly) {
```

```
      drawParameter( _panel, _g, -1170, 180, 10, 0, "otherHosp2SE", otherHosp2SE, 0 );
    }
    if (!_publicOnly) {
      drawParameter( _panel, _g, -1170, 160, 10, 0, "communityHosp2SE",
communityHosp2SE, 0 );
    }
    if (!_publicOnly) {
      drawParameter( _panel, _g, -1170, 140, 10, 0, "districtHosp2SE",
districtHosp2SE, 0 );
    }
    if (!_publicOnly) {
      drawParameter( _panel, _g, -1170, 370, 10, 0, "bobs2SE", bobs2SE, 0 );
    }
    if (!_publicOnly) {
      drawParameter( _panel, _g, -1170, 350, 10, 0, "fees2SE", fees2SE, 0 );
    }
    if (!_publicOnly) {
      drawParameter( _panel, _g, -1290, 490, 10, 0, "centralCS", centralCS, 0 );
    }
    if (!_publicOnly) {
      drawParameter( _panel, _g, -1290, 610, 10, 0, "clinicCS", clinicCS, 0 );
    }
    if (!_publicOnly) {
      drawParameter( _panel, _g, -1290, 590, 10, 0, "maternityCS", maternityCS, 0 );
    }
    if (!_publicOnly) {
      drawParameter( _panel, _g, -1290, 570, 10, 0, "healthCenterCS", healthCenterCS,
0 );
    }
    if (!_publicOnly) {
      drawParameter( _panel, _g, -1290, 550, 10, 0, "otherCS", otherCS, 0 );
    }
    if (!_publicOnly) {
      drawParameter( _panel, _g, -1290, 530, 10, 0, "communityCS", communityCS, 0 );
    }
    if (!_publicOnly) {
      drawParameter( _panel, _g, -1290, 510, 10, 0, "districtCS", districtCS, 0 );
    }
    if (!_publicOnly) {
      drawParameter( _panel, _g, -1290, 640, 10, 0, "centralCSSE", centralCSSE, 0 );
    }
    if (!_publicOnly) {
      drawParameter( _panel, _g, -1290, 760, 10, 0, "clinicCSSE", clinicCSSE, 0 );
    }
    if (!_publicOnly) {
      drawParameter( _panel, _g, -1290, 740, 10, 0, "maternityCSSE", maternityCSSE, 0
);
    }
    if (!_publicOnly) {
      drawParameter( _panel, _g, -1290, 720, 10, 0, "healthCenterCSSE",
healthCenterCSSE, 0 );
    }
    if (!_publicOnly) {
      drawParameter( _panel, _g, -1290, 700, 10, 0, "otherCSSE", otherCSSE, 0 );
    }
    if (!_publicOnly) {
      drawParameter( _panel, _g, -1290, 680, 10, 0, "communityCSSE", communityCSSE, 0
);
    }
    if (!_publicOnly) {
      drawParameter( _panel, _g, -1290, 660, 10, 0, "districtCSSE", districtCSSE, 0 );
    }
    if (!_publicOnly) {
```

```
      drawParameter( _panel, _g, -1040, 490, 10, 0, "centralNo", centralNo, 0 );
    }
    if (!_publicOnly) {
      drawParameter( _panel, _g, -1040, 610, 10, 0, "clinicNo", clinicNo, 0 );
    }
    if (!_publicOnly) {
      drawParameter( _panel, _g, -1040, 590, 10, 0, "maternityNo", maternityNo, 0 );
    }
    if (!_publicOnly) {
      drawParameter( _panel, _g, -1040, 570, 10, 0, "healthCenterNo", healthCenterNo,
0 );
    }
    if (!_publicOnly) {
      drawParameter( _panel, _g, -1040, 550, 10, 0, "otherNo", otherNo, 0 );
    }
    if (!_publicOnly) {
      drawParameter( _panel, _g, -1040, 530, 10, 0, "communityNo", communityNo, 0 );
    }
    if (!_publicOnly) {
      drawParameter( _panel, _g, -1040, 510, 10, 0, "districtNo", districtNo, 0 );
    }
    if (!_publicOnly) {
      drawParameter( _panel, _g, -1040, 640, 10, 0, "centralNoSE", centralNoSE, 0 );
    }
    if (!_publicOnly) {
      drawParameter( _panel, _g, -1040, 760, 10, 0, "clinicNoSE", clinicNoSE, 0 );
    }
    if (!_publicOnly) {
      drawParameter( _panel, _g, -1040, 740, 10, 0, "maternityNoSE", maternityNoSE, 0
);
    }
    if (!_publicOnly) {
      drawParameter( _panel, _g, -1040, 720, 10, 0, "healthCenterNoSE",
healthCenterNoSE, 0 );
    }
    if (!_publicOnly) {
      drawParameter( _panel, _g, -1040, 700, 10, 0, "otherNoSE", otherNoSE, 0 );
    }
    if (!_publicOnly) {
      drawParameter( _panel, _g, -1040, 680, 10, 0, "communityNoSE", communityNoSE, 0
);
    }
    if (!_publicOnly) {
      drawParameter( _panel, _g, -1040, 660, 10, 0, "districtNoSE", districtNoSE, 0 );
    }
    if (!_publicOnly) {
      drawParameter( _panel, _g, -1700, 490, 10, 0, "central5", central5, 0 );
    }
    if (!_publicOnly) {
      drawParameter( _panel, _g, -1700, 610, 10, 0, "clinic5", clinic5, 0 );
    }
    if (!_publicOnly) {
      drawParameter( _panel, _g, -1700, 590, 10, 0, "maternity5", maternity5, 0 );
    }
    if (!_publicOnly) {
      drawParameter( _panel, _g, -1700, 570, 10, 0, "healthCenter5", healthCenter5, 0
);
    }
    if (!_publicOnly) {
      drawParameter( _panel, _g, -1700, 550, 10, 0, "other5", other5, 0 );
    }
    if (!_publicOnly) {
      drawParameter( _panel, _g, -1700, 530, 10, 0, "community5", community5, 0 );
```

```
    }
    if (!_publicOnly) {
      drawParameter( _panel, _g, -1700, 510, 10, 0, "district5", district5, 0 );
    }
    if (!_publicOnly) {
      drawParameter( _panel, _g, -1700, 640, 10, 0, "central5SE", central5SE, 0 );
    }
    if (!_publicOnly) {
      drawParameter( _panel, _g, -1700, 760, 10, 0, "clinic5SE", clinic5SE, 0 );
    }
    if (!_publicOnly) {
      drawParameter( _panel, _g, -1700, 740, 10, 0, "maternity5SE", maternity5SE, 0 );
    }
    if (!_publicOnly) {
      drawParameter( _panel, _g, -1700, 720, 10, 0, "healthCenter5SE",
healthCenter5SE, 0 );
    }
    if (!_publicOnly) {
      drawParameter( _panel, _g, -1700, 700, 10, 0, "other5SE", other5SE, 0 );
    }
    if (!_publicOnly) {
      drawParameter( _panel, _g, -1700, 680, 10, 0, "community5SE", community5SE, 0 );
    }
    if (!_publicOnly) {
      drawParameter( _panel, _g, -1700, 660, 10, 0, "district5SE", district5SE, 0 );
    }
    if (!_publicOnly) {
      drawParameter( _panel, _g, -1500, 490, 10, 0, "centralNo5", centralNo5, 0 );
    }
    if (!_publicOnly) {
      drawParameter( _panel, _g, -1500, 610, 10, 0, "clinicNo5", clinicNo5, 0 );
    }
    if (!_publicOnly) {
      drawParameter( _panel, _g, -1500, 590, 10, 0, "maternityNo5", maternityNo5, 0 );
    }
    if (!_publicOnly) {
      drawParameter( _panel, _g, -1500, 570, 10, 0, "healthCenterNo5",
healthCenterNo5, 0 );
    }
    if (!_publicOnly) {
      drawParameter( _panel, _g, -1500, 550, 10, 0, "otherNo5", otherNo5, 0 );
    }
    if (!_publicOnly) {
      drawParameter( _panel, _g, -1500, 530, 10, 0, "communityNo5", communityNo5, 0 );
    }
    if (!_publicOnly) {
      drawParameter( _panel, _g, -1500, 510, 10, 0, "districtNo5", districtNo5, 0 );
    }
    if (!_publicOnly) {
      drawParameter( _panel, _g, -1500, 640, 10, 0, "centralNo5SE", centralNo5SE, 0 );
    }
    if (!_publicOnly) {
      drawParameter( _panel, _g, -1500, 760, 10, 0, "clinicNo5SE", clinicNo5SE, 0 );
    }
    if (!_publicOnly) {
      drawParameter( _panel, _g, -1500, 740, 10, 0, "maternityNo5SE", maternityNo5SE,
0 );
    }
    if (!_publicOnly) {
      drawParameter( _panel, _g, -1500, 720, 10, 0, "healthCenterNo5SE",
healthCenterNo5SE, 0 );
    }
    if (!_publicOnly) {
```

```
      drawParameter( _panel, _g, -1500, 700, 10, 0, "otherNo5SE", otherNo5SE, 0 );
    }
    if (!_publicOnly) {
      drawParameter( _panel, _g, -1500, 680, 10, 0, "communityNo5SE", communityNo5SE,
0 );
    }
    if (!_publicOnly) {
      drawParameter( _panel, _g, -1500, 660, 10, 0, "districtNo5SE", districtNo5SE, 0
);
    }
    if (!_publicOnly) {
      drawParameter( _panel, _g, -550, 280, 10, 0, "anc4", anc4, 0 );
    }
    if (!_publicOnly) {
      drawParameter( _panel, _g, -550, 260, 10, 0, "unwanted", unwanted, 0 );
    }
    if (!_publicOnly) {
      drawParameter( _panel, _g, -550, 300, 10, 0, "risk", risk, 0 );
    }
    if (!_publicOnly) {
      drawParameter( _panel, _g, -550, 140, 10, 0, "urban", urban, 0 );
    }
    if (!_publicOnly) {
      drawParameter( _panel, _g, -550, 220, 10, 0, "twins", twins, 0 );
    }
    if (!_publicOnly) {
      drawParameter( _panel, _g, -550, 200, 10, 0, "educSec", educSec, 0 );
    }
    if (!_publicOnly) {
      drawParameter( _panel, _g, -550, 180, 10, 0, "spouseAge", spouseAge, 0 );
    }
    if (!_publicOnly) {
      drawParameter( _panel, _g, -550, 160, 10, 0, "age", age, 0 );
    }
    if (!_publicOnly) {
      drawParameter( _panel, _g, -550, 320, 10, 0, "csPlanned", csPlanned, 0 );
    }
    if (!_publicOnly) {
      drawParameter( _panel, _g, -1020, 280, 10, 0, "anc4SE", anc4SE, 0 );
    }
    if (!_publicOnly) {
      drawParameter( _panel, _g, -1020, 260, 10, 0, "unwantedSE", unwantedSE, 0 );
    }
    if (!_publicOnly) {
      drawParameter( _panel, _g, -1020, 300, 10, 0, "riskSE", riskSE, 0 );
    }
    if (!_publicOnly) {
      drawParameter( _panel, _g, -1020, 140, 10, 0, "urbanSE", urbanSE, 0 );
    }
    if (!_publicOnly) {
      drawParameter( _panel, _g, -1020, 220, 10, 0, "twinsSE", twinsSE, 0 );
    }
    if (!_publicOnly) {
      drawParameter( _panel, _g, -1020, 200, 10, 0, "educSecSE", educSecSE, 0 );
    }
    if (!_publicOnly) {
      drawParameter( _panel, _g, -1020, 180, 10, 0, "spouseAgeSE", spouseAgeSE, 0 );
    }
    if (!_publicOnly) {
      drawParameter( _panel, _g, -1020, 160, 10, 0, "ageSE", ageSE, 0 );
    }
    if (!_publicOnly) {
      drawParameter( _panel, _g, -1020, 320, 10, 0, "csPlannedSE", csPlannedSE, 0 );
```

```
    }
    if (!_publicOnly) {
      drawParameter( _panel, _g, -160, 160, 10, 0, "QIParam", QIParam, 0 );
    }
    if (!_publicOnly) {
      drawParameter( _panel, _g, -280, 270, 10, 0, "strategy", strategy, 0 );
    }
  }

  @AnyLogicInternalCodegenAPI
  private void drawModelElements_PlainVariables_xjal(Panel _panel, Graphics2D _g,
boolean _publicOnly, boolean _isSuperClass ) {
    if (!_publicOnly) {
      drawPlainVariable( _panel, _g, -810, 500, 10, 0, "bCentral1", bCentral1, false
);
    }
    if (!_publicOnly) {
      drawPlainVariable( _panel, _g, -700, 500, 10, 0, "bCentral2", bCentral2, false
);
    }
    if (!_publicOnly) {
      drawPlainVariable( _panel, _g, -810, 520, 10, 0, "bDistrict1", bDistrict1, false
);
    }
    if (!_publicOnly) {
      drawPlainVariable( _panel, _g, -810, 560, 10, 0, "bOther1", bOther1, false );
    }
    if (!_publicOnly) {
      drawPlainVariable( _panel, _g, -810, 540, 10, 0, "bCommunity1", bCommunity1,
false );
    }
    if (!_publicOnly) {
      drawPlainVariable( _panel, _g, -810, 620, 10, 0, "bClinic1", bClinic1, false );
    }
    if (!_publicOnly) {
      drawPlainVariable( _panel, _g, -810, 600, 10, 0, "bMaternity1", bMaternity1,
false );
    }
    if (!_publicOnly) {
      drawPlainVariable( _panel, _g, -810, 580, 10, 0, "bHealthCenter1",
bHealthCenter1, false );
    }
    if (!_publicOnly) {
      drawPlainVariable( _panel, _g, -810, 720, 10, 0, "bBobs1", bBobs1, false );
    }
    if (!_publicOnly) {
      drawPlainVariable( _panel, _g, -810, 700, 10, 0, "bFees1", bFees1, false );
    }
    if (!_publicOnly) {
      drawPlainVariable( _panel, _g, -810, 680, 10, 0, "bQuality1", bQuality1, false
);
    }
    if (!_publicOnly) {
      drawPlainVariable( _panel, _g, -810, 660, 10, 0, "bDistance1", bDistance1, false
);
    }
    if (!_publicOnly) {
      drawPlainVariable( _panel, _g, -700, 520, 10, 0, "bDistrict2", bDistrict2, false
);
    }
    if (!_publicOnly) {
      drawPlainVariable( _panel, _g, -700, 560, 10, 0, "bOther2", bOther2, false );
    }
```

```
    if (!_publicOnly) {
      drawPlainVariable( _panel, _g, -700, 540, 10, 0, "bCommunity2", bCommunity2,
false );
    }
    if (!_publicOnly) {
      drawPlainVariable( _panel, _g, -700, 620, 10, 0, "bClinic2", bClinic2, false );
    }
    if (!_publicOnly) {
      drawPlainVariable( _panel, _g, -700, 600, 10, 0, "bMaternity2", bMaternity2,
false );
    }
    if (!_publicOnly) {
      drawPlainVariable( _panel, _g, -700, 580, 10, 0, "bHealthCenter2",
bHealthCenter2, false );
    }
    if (!_publicOnly) {
      drawPlainVariable( _panel, _g, -700, 720, 10, 0, "bBobs2", bBobs2, false );
    }
    if (!_publicOnly) {
      drawPlainVariable( _panel, _g, -700, 700, 10, 0, "bFees2", bFees2, false );
    }
    if (!_publicOnly) {
      drawPlainVariable( _panel, _g, -700, 680, 10, 0, "bQuality2", bQuality2, false
);
    }
    if (!_publicOnly) {
      drawPlainVariable( _panel, _g, -700, 660, 10, 0, "bDistance2", bDistance2, false
);
    }
    if (!_publicOnly) {
      drawPlainVariable( _panel, _g, -560, 500, 10, 0, "bPoorest", bPoorest, false );
    }
    if (!_publicOnly) {
      drawPlainVariable( _panel, _g, -560, 540, 10, 0, "bMiddle", bMiddle, false );
    }
    if (!_publicOnly) {
      drawPlainVariable( _panel, _g, -560, 520, 10, 0, "bPoor", bPoor, false );
    }
    if (!_publicOnly) {
      drawPlainVariable( _panel, _g, -560, 600, 10, 0, "bIntercept", bIntercept, false
);
    }
    if (!_publicOnly) {
      drawPlainVariable( _panel, _g, -560, 580, 10, 0, "bRichest", bRichest, false );
    }
    if (!_publicOnly) {
      drawPlainVariable( _panel, _g, -560, 560, 10, 0, "bRich", bRich, false );
    }
    if (!_publicOnly) {
      drawPlainVariable( _panel, _g, -560, 680, 10, 0, "bIlliterate", bIlliterate,
false );
    }
    if (!_publicOnly) {
      drawPlainVariable( _panel, _g, -560, 660, 10, 0, "bBlind", bBlind, false );
    }
    if (!_publicOnly) {
      drawPlainVariable( _panel, _g, -560, 640, 10, 0, "bPrimip", bPrimip, false );
    }
    if (!_publicOnly) {
      drawPlainVariable( _panel, _g, -480, 500, 10, 0, "bUrban", bUrban, false );
    }
    if (!_publicOnly) {
```

```
      drawPlainVariable( _panel, _g, -480, 540, 10, 0, "bSpouseAge", bSpouseAge, false
);
    }
    if (!_publicOnly) {
      drawPlainVariable( _panel, _g, -480, 520, 10, 0, "bAge", bAge, false );
    }
    if (!_publicOnly) {
      drawPlainVariable( _panel, _g, -480, 600, 10, 0, "bUnwanted", bUnwanted, false
);
    }
    if (!_publicOnly) {
      drawPlainVariable( _panel, _g, -480, 580, 10, 0, "bTwins", bTwins, false );
    }
    if (!_publicOnly) {
      drawPlainVariable( _panel, _g, -480, 560, 10, 0, "bEducSec", bEducSec, false );
    }
    if (!_publicOnly) {
      drawPlainVariable( _panel, _g, -480, 680, 10, 0, "bCSPlanned", bCSPlanned, false
);
    }
    if (!_publicOnly) {
      drawPlainVariable( _panel, _g, -480, 660, 10, 0, "bRisk", bRisk, false );
    }
    if (!_publicOnly) {
      drawPlainVariable( _panel, _g, -480, 640, 10, 0, "bANC4", bANC4, false );
    }
    if (!_publicOnly) {
      drawPlainVariable( _panel, _g, -160, 100, 10, 0, "vAgentID", vAgentID, false );
    }
    if (!_publicOnly) {
      drawPlainVariable( _panel, _g, -280, 190, 10, 0, "allowFees", allowFees, false
);
    }
    if (!_publicOnly) {
      drawPlainVariable( _panel, _g, -280, 220, 10, 0, "allowAll", allowAll, false );
    }
    if (!_publicOnly) {
      drawPlainVariable( _panel, _g, -280, 160, 10, 0, "QI", QI, false );
    }
  }

  @AnyLogicInternalCodegenAPI
  private void drawModelElements_CollectionVariables_xjal(Panel _panel, Graphics2D _g,
boolean _publicOnly, boolean _isSuperClass ) {
    if (!_publicOnly) {
      drawCollection( _panel, _g, -160, 260, 10, 0, "hospitalList", hospitalList );
    }
    if (!_publicOnly) {
      drawCollection( _panel, _g, -160, 300, 10, 0, "allowedList", allowedList );
    }
  }

  @AnyLogicInternalCodegenAPI
  private void drawModelElements_DataElements_xjal(Panel _panel, Graphics2D _g,
boolean _publicOnly, boolean _isSuperClass ) {
    if (!_publicOnly) {
      drawHistogramData( _panel, _g, 1350, 620, 15, 0, "uDiff", uDiff );
    }
    if (!_publicOnly) {
      drawHistogramData( _panel, _g, 1300, 390, 15, 0, "travelDistance",
travelDistance );
    }
    if (!_publicOnly) {
```

```
      drawHistogramData( _panel, _g, 1230, 160, 15, 0, "selectedBobs", selectedBobs );
    }
  }

  @AnyLogicInternalCodegenAPI
  private void drawModelElements_Outputs_xjal(Panel _panel, Graphics2D _g, boolean
_publicOnly, boolean _isSuperClass ) {
    if (!_publicOnly) {
      drawOutput( _panel, _g, 750, 110, 15, 0, "travelTime", travelTime );
    }
    if (!_publicOnly) {
      drawOutput( _panel, _g, 750, 30, 15, 0, "popSize", popSize );
    }
    if (!_publicOnly) {
      drawOutput( _panel, _g, 850, 30, 15, 0, "segment2", segment2 );
    }
    if (!_publicOnly) {
      drawOutput( _panel, _g, 950, 30, 15, 0, "urbanPct", urbanPct );
    }
    if (!_publicOnly) {
      drawOutput( _panel, _g, 850, 110, 15, 0, "utilityDifference", utilityDifference
);
    }
    if (!_publicOnly) {
      drawOutput( _panel, _g, 750, 70, 15, 0, "classAssigned", classAssigned );
    }
    if (!_publicOnly) {
      drawOutput( _panel, _g, 980, 110, 15, 0, "avgBobs", avgBobs );
    }
    if (!_publicOnly) {
      drawOutput( _panel, _g, 1070, 110, 15, 0, "avgFees", avgFees );
    }
    if (!_publicOnly) {
      drawOutput( _panel, _g, 870, 70, 15, 0, "hospitalAssigned", hospitalAssigned );
    }
    if (!_publicOnly) {
      drawOutput( _panel, _g, 1050, 30, 15, 0, "meanDistance", meanDistance );
    }
    if (!_publicOnly) {
      drawOutput( _panel, _g, 1000, 70, 15, 0, "dist60km", dist60km );
    }
    if (!_publicOnly) {
      drawOutput( _panel, _g, 1090, 70, 15, 0, "distFurther", distFurther );
    }
    if (!_publicOnly) {
      drawOutput( _panel, _g, 1150, 110, 15, 0, "meanBobs", meanBobs );
    }
    if (!_publicOnly) {
      drawOutput( _panel, _g, 1190, 30, 15, 0, "neonatalMortality", neonatalMortality
);
    }
    if (!_publicOnly) {
      drawOutput( _panel, _g, 1200, 70, 15, 0, "csRate", csRate );
    }
    if (!_publicOnly) {
      drawOutput( _panel, _g, 1300, 70, 15, 0, "avgOOP", avgOOP );
    }
    if (!_publicOnly) {
      drawOutput( _panel, _g, 550, 340, 15, 0, "catExpPoorest", catExpPoorest );
    }
    if (!_publicOnly) {
      drawOutput( _panel, _g, 550, 540, 15, 0, "catExpTotal", catExpTotal );
    }
```

```
    if (!_publicOnly) {
      drawOutput( _panel, _g, 550, 500, 15, 0, "catExpRichest", catExpRichest );
    }
    if (!_publicOnly) {
      drawOutput( _panel, _g, 550, 460, 15, 0, "catExpRich", catExpRich );
    }
    if (!_publicOnly) {
      drawOutput( _panel, _g, 550, 420, 15, 0, "catExpMiddle", catExpMiddle );
    }
    if (!_publicOnly) {
      drawOutput( _panel, _g, 550, 380, 15, 0, "catExpPoor", catExpPoor );
    }
  }

  @AnyLogicInternalCodegenAPI
  private void drawModelElements_EmbeddeObjects_xjal(Panel _panel, Graphics2D _g,
boolean _publicOnly, boolean _isSuperClass ) {
    // Embedded object "hospitals"
    if (!_publicOnly) {
      drawEmbeddedObjectModelDefault( _panel, _g, -160 , 40 , 10, 0, "hospitals",
this.hospitals );
    }
    // Embedded object "people"
    if (!_publicOnly) {
      drawEmbeddedObjectModelDefault( _panel, _g, -160 , 70 , 10, 0, "people",
this.people );
    }
  }

  @AnyLogicInternalCodegenAPI
  private void drawModelElements_AgentLinks_xjal(Panel _panel, Graphics2D _g, boolean
_publicOnly, boolean _isSuperClass ) {
    if (_publicOnly) { return; }
      drawLinkToAgent( _panel, _g, 50, -50, 15, 0, "connections", true, connections );
  }

  @Override
  @AnyLogicInternalCodegenAPI
  public void drawModelElements( Panel _panel, Graphics2D _g, boolean _publicOnly,
boolean _isSuperClass ) {
       super.drawModelElements( _panel, _g, _publicOnly, true );
    drawModelElements_Connectivity_xjal( _panel, _g, _publicOnly, _isSuperClass );
    drawModelElements_Events_xjal( _panel, _g, _publicOnly, _isSuperClass );
    drawModelElements_Parameters_xjal( _panel, _g, _publicOnly, _isSuperClass );
    drawModelElements_PlainVariables_xjal( _panel, _g, _publicOnly, _isSuperClass );
    drawModelElements_CollectionVariables_xjal( _panel, _g, _publicOnly, _isSuperClass
);
    drawModelElements_DataElements_xjal( _panel, _g, _publicOnly, _isSuperClass );
    drawModelElements_Outputs_xjal( _panel, _g, _publicOnly, _isSuperClass );
    drawModelElements_EmbeddeObjects_xjal( _panel, _g, _publicOnly, _isSuperClass );
    drawModelElements_AgentLinks_xjal( _panel, _g, _publicOnly, _isSuperClass );
  }

  @AnyLogicInternalCodegenAPI
  private boolean onClickModelAt_EmbeddedObjects_xjal( Panel _panel, double _x, double
_y, int _clickCount, boolean _publicOnly, boolean _isSuperClass ) {
    if ( !hospitals.isEmpty() && modelElementContains(_x, _y, -160, 40) ) {
      if ( _clickCount == 2 ) {
        _panel.browseAgent_xjal( -160, 40, this, "hospitals" );
      } else {
        _panel.addInspect( -160, 40, this, "hospitals" );
      }
      return true;
```

```
    }
    if ( !people.isEmpty() && modelElementContains(_x, _y, -160, 70) ) {
      if ( _clickCount == 2 ) {
        _panel.browseAgent_xjal( -160, 70, this, "people" );
      } else {
        _panel.addInspect( -160, 70, this, "people" );
      }
      return true;
    }
    return false;
  }

  @AnyLogicInternalCodegenAPI
  private boolean onClickModelAt_AgentLinks_xjal( Panel _panel, double _x, double _y,
int _clickCount, boolean _publicOnly, boolean _isSuperClass ) {
    if ( modelElementContains(_x, _y, 50, -50) ) {
        _panel.addInspect_xjal( 50, -50, this, "connections",
Panel.INSPECT_CONNECTIONS_xjal );
      return true;
    }
    return false;
  }


  @AnyLogicInternalCodegenAPI
  private boolean onClickModelAt_Connectivity_xjal( Panel _panel, double _x, double
_y, int _clickCount, boolean _publicOnly, boolean _isSuperClass ) {
    if( !_publicOnly && modelElementContains(_x, _y, -160, 410) ) {
      _panel.addInspect( -160, 410, this, "distanceWTP" );
      return true;
    }
    if( !_publicOnly && modelElementContains(_x, _y, -150, 460) ) {
      _panel.addInspect( -150, 460, this, "newClassAssignment" );
      return true;
    }
    return false;
  }

  @AnyLogicInternalCodegenAPI
  private boolean onClickModelAt_Parameters_xjal( Panel _panel, double _x, double _y,
int _clickCount, boolean _publicOnly, boolean _isSuperClass ) {
    if( !_publicOnly && modelElementContains(_x, _y, -160, 190) ) {
      _panel.addInspect( -160, 190, this, "allowFeesParam" );
      return true;
    }
    if( !_publicOnly && modelElementContains(_x, _y, -810, 120) ) {
      _panel.addInspect( -810, 120, this, "centralHosp1" );
      return true;
    }
    if( !_publicOnly && modelElementContains(_x, _y, -810, 310) ) {
      _panel.addInspect( -810, 310, this, "distance1" );
      return true;
    }
    if( !_publicOnly && modelElementContains(_x, _y, -810, 240) ) {
      _panel.addInspect( -810, 240, this, "clinic1" );
      return true;
    }
    if( !_publicOnly && modelElementContains(_x, _y, -810, 220) ) {
      _panel.addInspect( -810, 220, this, "maternity1" );
      return true;
    }
    if( !_publicOnly && modelElementContains(_x, _y, -810, 200) ) {
      _panel.addInspect( -810, 200, this, "healthCenter1" );
```

```
    return true;
  }
  if( !_publicOnly && modelElementContains(_x, _y, -810, 180) ) {
    _panel.addInspect( -810, 180, this, "otherHosp1" );
    return true;
  }
  if( !_publicOnly && modelElementContains(_x, _y, -810, 160) ) {
    _panel.addInspect( -810, 160, this, "communityHosp1" );
    return true;
  }
  if( !_publicOnly && modelElementContains(_x, _y, -810, 140) ) {
    _panel.addInspect( -810, 140, this, "districtHosp1" );
    return true;
  }
  if( !_publicOnly && modelElementContains(_x, _y, -810, 370) ) {
    _panel.addInspect( -810, 370, this, "bobs1" );
    return true;
  }
  if( !_publicOnly && modelElementContains(_x, _y, -810, 350) ) {
    _panel.addInspect( -810, 350, this, "fees1" );
    return true;
  }
  if( !_publicOnly && modelElementContains(_x, _y, -160, 220) ) {
    _panel.addInspect( -160, 220, this, "allowAllParam" );
    return true;
  }
  if( !_publicOnly && modelElementContains(_x, _y, -550, 100) ) {
    _panel.addInspect( -550, 100, this, "intercept" );
    return true;
  }
  if( !_publicOnly && modelElementContains(_x, _y, -690, 120) ) {
    _panel.addInspect( -690, 120, this, "centralHosp2" );
    return true;
  }
  if( !_publicOnly && modelElementContains(_x, _y, -690, 310) ) {
    _panel.addInspect( -690, 310, this, "distance2" );
    return true;
  }
  if( !_publicOnly && modelElementContains(_x, _y, -690, 240) ) {
    _panel.addInspect( -690, 240, this, "clinic2" );
    return true;
  }
  if( !_publicOnly && modelElementContains(_x, _y, -690, 220) ) {
    _panel.addInspect( -690, 220, this, "maternity2" );
    return true;
  }
  if( !_publicOnly && modelElementContains(_x, _y, -690, 200) ) {
    _panel.addInspect( -690, 200, this, "healthCenter2" );
    return true;
  }
  if( !_publicOnly && modelElementContains(_x, _y, -690, 180) ) {
    _panel.addInspect( -690, 180, this, "otherHosp2" );
    return true;
  }
  if( !_publicOnly && modelElementContains(_x, _y, -690, 160) ) {
    _panel.addInspect( -690, 160, this, "communityHosp2" );
    return true;
  }
  if( !_publicOnly && modelElementContains(_x, _y, -690, 140) ) {
    _panel.addInspect( -690, 140, this, "districtHosp2" );
    return true;
  }
  if( !_publicOnly && modelElementContains(_x, _y, -690, 370) ) {
```

```
  _panel.addInspect( -690, 370, this, "bobs2" );
  return true;
}
if( !_publicOnly && modelElementContains(_x, _y, -690, 350) ) {
  _panel.addInspect( -690, 350, this, "fees2" );
  return true;
}
if( !_publicOnly && modelElementContains(_x, _y, -550, 350) ) {
  _panel.addInspect( -550, 350, this, "distCenter" );
  return true;
}
if( !_publicOnly && modelElementContains(_x, _y, -550, 370) ) {
  _panel.addInspect( -550, 370, this, "distRMS" );
  return true;
}
if( !_publicOnly && modelElementContains(_x, _y, -1290, 120) ) {
  _panel.addInspect( -1290, 120, this, "centralHosp1SE" );
  return true;
}
if( !_publicOnly && modelElementContains(_x, _y, -1290, 310) ) {
  _panel.addInspect( -1290, 310, this, "distance1SE" );
  return true;
}
if( !_publicOnly && modelElementContains(_x, _y, -1290, 240) ) {
  _panel.addInspect( -1290, 240, this, "clinic1SE" );
  return true;
}
if( !_publicOnly && modelElementContains(_x, _y, -1290, 220) ) {
  _panel.addInspect( -1290, 220, this, "maternity1SE" );
  return true;
}
if( !_publicOnly && modelElementContains(_x, _y, -1290, 200) ) {
  _panel.addInspect( -1290, 200, this, "healthCenter1SE" );
  return true;
}
if( !_publicOnly && modelElementContains(_x, _y, -1290, 180) ) {
  _panel.addInspect( -1290, 180, this, "otherHosp1SE" );
  return true;
}
if( !_publicOnly && modelElementContains(_x, _y, -1290, 160) ) {
  _panel.addInspect( -1290, 160, this, "communityHosp1SE" );
  return true;
}
if( !_publicOnly && modelElementContains(_x, _y, -1290, 140) ) {
  _panel.addInspect( -1290, 140, this, "districtHosp1SE" );
  return true;
}
if( !_publicOnly && modelElementContains(_x, _y, -1290, 370) ) {
  _panel.addInspect( -1290, 370, this, "bobs1SE" );
  return true;
}
if( !_publicOnly && modelElementContains(_x, _y, -1290, 350) ) {
  _panel.addInspect( -1290, 350, this, "fees1SE" );
  return true;
}
if( !_publicOnly && modelElementContains(_x, _y, -1020, 100) ) {
  _panel.addInspect( -1020, 100, this, "interceptSE" );
  return true;
}
if( !_publicOnly && modelElementContains(_x, _y, -1170, 120) ) {
  _panel.addInspect( -1170, 120, this, "centralHosp2SE" );
  return true;
}
```

```
if( !_publicOnly && modelElementContains(_x, _y, -1170, 310) ) {
  _panel.addInspect( -1170, 310, this, "distance2SE" );
  return true;
}
if( !_publicOnly && modelElementContains(_x, _y, -1170, 240) ) {
  _panel.addInspect( -1170, 240, this, "clinic2SE" );
  return true;
}
if( !_publicOnly && modelElementContains(_x, _y, -1170, 220) ) {
  _panel.addInspect( -1170, 220, this, "maternity2SE" );
  return true;
}
if( !_publicOnly && modelElementContains(_x, _y, -1170, 200) ) {
  _panel.addInspect( -1170, 200, this, "healthCenter2SE" );
  return true;
}
if( !_publicOnly && modelElementContains(_x, _y, -1170, 180) ) {
  _panel.addInspect( -1170, 180, this, "otherHosp2SE" );
  return true;
}
if( !_publicOnly && modelElementContains(_x, _y, -1170, 160) ) {
  _panel.addInspect( -1170, 160, this, "communityHosp2SE" );
  return true;
}
if( !_publicOnly && modelElementContains(_x, _y, -1170, 140) ) {
  _panel.addInspect( -1170, 140, this, "districtHosp2SE" );
  return true;
}
if( !_publicOnly && modelElementContains(_x, _y, -1170, 370) ) {
  _panel.addInspect( -1170, 370, this, "bobs2SE" );
  return true;
}
if( !_publicOnly && modelElementContains(_x, _y, -1170, 350) ) {
  _panel.addInspect( -1170, 350, this, "fees2SE" );
  return true;
}
if( !_publicOnly && modelElementContains(_x, _y, -1290, 490) ) {
  _panel.addInspect( -1290, 490, this, "centralCS" );
  return true;
}
if( !_publicOnly && modelElementContains(_x, _y, -1290, 610) ) {
  _panel.addInspect( -1290, 610, this, "clinicCS" );
  return true;
}
if( !_publicOnly && modelElementContains(_x, _y, -1290, 590) ) {
  _panel.addInspect( -1290, 590, this, "maternityCS" );
  return true;
}
if( !_publicOnly && modelElementContains(_x, _y, -1290, 570) ) {
  _panel.addInspect( -1290, 570, this, "healthCenterCS" );
  return true;
}
if( !_publicOnly && modelElementContains(_x, _y, -1290, 550) ) {
  _panel.addInspect( -1290, 550, this, "otherCS" );
  return true;
}
if( !_publicOnly && modelElementContains(_x, _y, -1290, 530) ) {
  _panel.addInspect( -1290, 530, this, "communityCS" );
  return true;
}
if( !_publicOnly && modelElementContains(_x, _y, -1290, 510) ) {
  _panel.addInspect( -1290, 510, this, "districtCS" );
  return true;
```

```
}
if( !_publicOnly && modelElementContains(_x, _y, -1290, 640) ) {
  _panel.addInspect( -1290, 640, this, "centralCSSE" );
  return true;
}
if( !_publicOnly && modelElementContains(_x, _y, -1290, 760) ) {
  _panel.addInspect( -1290, 760, this, "clinicCSSE" );
  return true;
}
if( !_publicOnly && modelElementContains(_x, _y, -1290, 740) ) {
  _panel.addInspect( -1290, 740, this, "maternityCSSE" );
  return true;
}
if( !_publicOnly && modelElementContains(_x, _y, -1290, 720) ) {
  _panel.addInspect( -1290, 720, this, "healthCenterCSSE" );
  return true;
}
if( !_publicOnly && modelElementContains(_x, _y, -1290, 700) ) {
  _panel.addInspect( -1290, 700, this, "otherCSSE" );
  return true;
}
if( !_publicOnly && modelElementContains(_x, _y, -1290, 680) ) {
  _panel.addInspect( -1290, 680, this, "communityCSSE" );
  return true;
}
if( !_publicOnly && modelElementContains(_x, _y, -1290, 660) ) {
  _panel.addInspect( -1290, 660, this, "districtCSSE" );
  return true;
}
if( !_publicOnly && modelElementContains(_x, _y, -1040, 490) ) {
  _panel.addInspect( -1040, 490, this, "centralNo" );
  return true;
}
if( !_publicOnly && modelElementContains(_x, _y, -1040, 610) ) {
  _panel.addInspect( -1040, 610, this, "clinicNo" );
  return true;
}
if( !_publicOnly && modelElementContains(_x, _y, -1040, 590) ) {
  _panel.addInspect( -1040, 590, this, "maternityNo" );
  return true;
}
if( !_publicOnly && modelElementContains(_x, _y, -1040, 570) ) {
  _panel.addInspect( -1040, 570, this, "healthCenterNo" );
  return true;
}
if( !_publicOnly && modelElementContains(_x, _y, -1040, 550) ) {
  _panel.addInspect( -1040, 550, this, "otherNo" );
  return true;
}
if( !_publicOnly && modelElementContains(_x, _y, -1040, 530) ) {
  _panel.addInspect( -1040, 530, this, "communityNo" );
  return true;
}
if( !_publicOnly && modelElementContains(_x, _y, -1040, 510) ) {
  _panel.addInspect( -1040, 510, this, "districtNo" );
  return true;
}
if( !_publicOnly && modelElementContains(_x, _y, -1040, 640) ) {
  _panel.addInspect( -1040, 640, this, "centralNoSE" );
  return true;
}
if( !_publicOnly && modelElementContains(_x, _y, -1040, 760) ) {
  _panel.addInspect( -1040, 760, this, "clinicNoSE" );
```

```
    return true;
  }
  if( !_publicOnly && modelElementContains(_x, _y, -1040, 740) ) {
    _panel.addInspect( -1040, 740, this, "maternityNoSE" );
    return true;
  }
  if( !_publicOnly && modelElementContains(_x, _y, -1040, 720) ) {
    _panel.addInspect( -1040, 720, this, "healthCenterNoSE" );
    return true;
  }
  if( !_publicOnly && modelElementContains(_x, _y, -1040, 700) ) {
    _panel.addInspect( -1040, 700, this, "otherNoSE" );
    return true;
  }
  if( !_publicOnly && modelElementContains(_x, _y, -1040, 680) ) {
    _panel.addInspect( -1040, 680, this, "communityNoSE" );
    return true;
  }
  if( !_publicOnly && modelElementContains(_x, _y, -1040, 660) ) {
    _panel.addInspect( -1040, 660, this, "districtNoSE" );
    return true;
  }
  if( !_publicOnly && modelElementContains(_x, _y, -1700, 490) ) {
    _panel.addInspect( -1700, 490, this, "central5" );
    return true;
  }
  if( !_publicOnly && modelElementContains(_x, _y, -1700, 610) ) {
    _panel.addInspect( -1700, 610, this, "clinic5" );
    return true;
  }
  if( !_publicOnly && modelElementContains(_x, _y, -1700, 590) ) {
    _panel.addInspect( -1700, 590, this, "maternity5" );
    return true;
  }
  if( !_publicOnly && modelElementContains(_x, _y, -1700, 570) ) {
    _panel.addInspect( -1700, 570, this, "healthCenter5" );
    return true;
  }
  if( !_publicOnly && modelElementContains(_x, _y, -1700, 550) ) {
    _panel.addInspect( -1700, 550, this, "other5" );
    return true;
  }
  if( !_publicOnly && modelElementContains(_x, _y, -1700, 530) ) {
    _panel.addInspect( -1700, 530, this, "community5" );
    return true;
  }
  if( !_publicOnly && modelElementContains(_x, _y, -1700, 510) ) {
    _panel.addInspect( -1700, 510, this, "district5" );
    return true;
  }
  if( !_publicOnly && modelElementContains(_x, _y, -1700, 640) ) {
    _panel.addInspect( -1700, 640, this, "central5SE" );
    return true;
  }
  if( !_publicOnly && modelElementContains(_x, _y, -1700, 760) ) {
    _panel.addInspect( -1700, 760, this, "clinic5SE" );
    return true;
  }
  if( !_publicOnly && modelElementContains(_x, _y, -1700, 740) ) {
    _panel.addInspect( -1700, 740, this, "maternity5SE" );
    return true;
  }
  if( !_publicOnly && modelElementContains(_x, _y, -1700, 720) ) {
```

```
  _panel.addInspect( -1700, 720, this, "healthCenter5SE" );
  return true;
}
if( !_publicOnly && modelElementContains(_x, _y, -1700, 700) ) {
  _panel.addInspect( -1700, 700, this, "other5SE" );
  return true;
}
if( !_publicOnly && modelElementContains(_x, _y, -1700, 680) ) {
  _panel.addInspect( -1700, 680, this, "community5SE" );
  return true;
}
if( !_publicOnly && modelElementContains(_x, _y, -1700, 660) ) {
  _panel.addInspect( -1700, 660, this, "district5SE" );
  return true;
}
if( !_publicOnly && modelElementContains(_x, _y, -1500, 490) ) {
  _panel.addInspect( -1500, 490, this, "centralNo5" );
  return true;
}
if( !_publicOnly && modelElementContains(_x, _y, -1500, 610) ) {
  _panel.addInspect( -1500, 610, this, "clinicNo5" );
  return true;
}
if( !_publicOnly && modelElementContains(_x, _y, -1500, 590) ) {
  _panel.addInspect( -1500, 590, this, "maternityNo5" );
  return true;
}
if( !_publicOnly && modelElementContains(_x, _y, -1500, 570) ) {
  _panel.addInspect( -1500, 570, this, "healthCenterNo5" );
  return true;
}
if( !_publicOnly && modelElementContains(_x, _y, -1500, 550) ) {
  _panel.addInspect( -1500, 550, this, "otherNo5" );
  return true;
}
if( !_publicOnly && modelElementContains(_x, _y, -1500, 530) ) {
  _panel.addInspect( -1500, 530, this, "communityNo5" );
  return true;
}
if( !_publicOnly && modelElementContains(_x, _y, -1500, 510) ) {
  _panel.addInspect( -1500, 510, this, "districtNo5" );
  return true;
}
if( !_publicOnly && modelElementContains(_x, _y, -1500, 640) ) {
  _panel.addInspect( -1500, 640, this, "centralNo5SE" );
  return true;
}
if( !_publicOnly && modelElementContains(_x, _y, -1500, 760) ) {
  _panel.addInspect( -1500, 760, this, "clinicNo5SE" );
  return true;
}
if( !_publicOnly && modelElementContains(_x, _y, -1500, 740) ) {
  _panel.addInspect( -1500, 740, this, "maternityNo5SE" );
  return true;
}
if( !_publicOnly && modelElementContains(_x, _y, -1500, 720) ) {
  _panel.addInspect( -1500, 720, this, "healthCenterNo5SE" );
  return true;
}
if( !_publicOnly && modelElementContains(_x, _y, -1500, 700) ) {
  _panel.addInspect( -1500, 700, this, "otherNo5SE" );
  return true;
}
```

```
if( !_publicOnly && modelElementContains(_x, _y, -1500, 680) ) {
  _panel.addInspect( -1500, 680, this, "communityNo5SE" );
  return true;
}
if( !_publicOnly && modelElementContains(_x, _y, -1500, 660) ) {
  _panel.addInspect( -1500, 660, this, "districtNo5SE" );
  return true;
}
if( !_publicOnly && modelElementContains(_x, _y, -550, 280) ) {
  _panel.addInspect( -550, 280, this, "anc4" );
  return true;
}
if( !_publicOnly && modelElementContains(_x, _y, -550, 260) ) {
  _panel.addInspect( -550, 260, this, "unwanted" );
  return true;
}
if( !_publicOnly && modelElementContains(_x, _y, -550, 300) ) {
  _panel.addInspect( -550, 300, this, "risk" );
  return true;
}
if( !_publicOnly && modelElementContains(_x, _y, -550, 140) ) {
  _panel.addInspect( -550, 140, this, "urban" );
  return true;
}
if( !_publicOnly && modelElementContains(_x, _y, -550, 220) ) {
  _panel.addInspect( -550, 220, this, "twins" );
  return true;
}
if( !_publicOnly && modelElementContains(_x, _y, -550, 200) ) {
  _panel.addInspect( -550, 200, this, "educSec" );
  return true;
}
if( !_publicOnly && modelElementContains(_x, _y, -550, 180) ) {
  _panel.addInspect( -550, 180, this, "spouseAge" );
  return true;
}
if( !_publicOnly && modelElementContains(_x, _y, -550, 160) ) {
  _panel.addInspect( -550, 160, this, "age" );
  return true;
}
if( !_publicOnly && modelElementContains(_x, _y, -550, 320) ) {
  _panel.addInspect( -550, 320, this, "csPlanned" );
  return true;
}
if( !_publicOnly && modelElementContains(_x, _y, -1020, 280) ) {
  _panel.addInspect( -1020, 280, this, "anc4SE" );
  return true;
}
if( !_publicOnly && modelElementContains(_x, _y, -1020, 260) ) {
  _panel.addInspect( -1020, 260, this, "unwantedSE" );
  return true;
}
if( !_publicOnly && modelElementContains(_x, _y, -1020, 300) ) {
  _panel.addInspect( -1020, 300, this, "riskSE" );
  return true;
}
if( !_publicOnly && modelElementContains(_x, _y, -1020, 140) ) {
  _panel.addInspect( -1020, 140, this, "urbanSE" );
  return true;
}
if( !_publicOnly && modelElementContains(_x, _y, -1020, 220) ) {
  _panel.addInspect( -1020, 220, this, "twinsSE" );
  return true;
```

```
    }
    if( !_publicOnly && modelElementContains(_x, _y, -1020, 200) ) {
      _panel.addInspect( -1020, 200, this, "educSecSE" );
      return true;
    }
    if( !_publicOnly && modelElementContains(_x, _y, -1020, 180) ) {
      _panel.addInspect( -1020, 180, this, "spouseAgeSE" );
      return true;
    }
    if( !_publicOnly && modelElementContains(_x, _y, -1020, 160) ) {
      _panel.addInspect( -1020, 160, this, "ageSE" );
      return true;
    }
    if( !_publicOnly && modelElementContains(_x, _y, -1020, 320) ) {
      _panel.addInspect( -1020, 320, this, "csPlannedSE" );
      return true;
    }
    if( !_publicOnly && modelElementContains(_x, _y, -160, 160) ) {
      _panel.addInspect( -160, 160, this, "QIParam" );
      return true;
    }
    if( !_publicOnly && modelElementContains(_x, _y, -280, 270) ) {
      _panel.addInspect( -280, 270, this, "strategy" );
      return true;
    }
    return false;
  }

  @AnyLogicInternalCodegenAPI
  private boolean onClickModelAt_PlainVariables_xjal( Panel _panel, double _x, double
_y, int _clickCount, boolean _publicOnly, boolean _isSuperClass ) {
    if( !_publicOnly && modelElementContains(_x, _y, -810, 500) ) {
      _panel.addInspect( -810, 500, this, "bCentral1" );
      return true;
    }
    if( !_publicOnly && modelElementContains(_x, _y, -700, 500) ) {
      _panel.addInspect( -700, 500, this, "bCentral2" );
      return true;
    }
    if( !_publicOnly && modelElementContains(_x, _y, -810, 520) ) {
      _panel.addInspect( -810, 520, this, "bDistrict1" );
      return true;
    }
    if( !_publicOnly && modelElementContains(_x, _y, -810, 560) ) {
      _panel.addInspect( -810, 560, this, "bOther1" );
      return true;
    }
    if( !_publicOnly && modelElementContains(_x, _y, -810, 540) ) {
      _panel.addInspect( -810, 540, this, "bCommunity1" );
      return true;
    }
    if( !_publicOnly && modelElementContains(_x, _y, -810, 620) ) {
      _panel.addInspect( -810, 620, this, "bClinic1" );
      return true;
    }
    if( !_publicOnly && modelElementContains(_x, _y, -810, 600) ) {
      _panel.addInspect( -810, 600, this, "bMaternity1" );
      return true;
    }
    if( !_publicOnly && modelElementContains(_x, _y, -810, 580) ) {
      _panel.addInspect( -810, 580, this, "bHealthCenter1" );
      return true;
    }
```

```
if( !_publicOnly && modelElementContains(_x, _y, -810, 720) ) {
  _panel.addInspect( -810, 720, this, "bBobs1" );
  return true;
}
if( !_publicOnly && modelElementContains(_x, _y, -810, 700) ) {
  _panel.addInspect( -810, 700, this, "bFees1" );
  return true;
}
if( !_publicOnly && modelElementContains(_x, _y, -810, 680) ) {
  _panel.addInspect( -810, 680, this, "bQuality1" );
  return true;
}
if( !_publicOnly && modelElementContains(_x, _y, -810, 660) ) {
  _panel.addInspect( -810, 660, this, "bDistance1" );
  return true;
}
if( !_publicOnly && modelElementContains(_x, _y, -700, 520) ) {
  _panel.addInspect( -700, 520, this, "bDistrict2" );
  return true;
}
if( !_publicOnly && modelElementContains(_x, _y, -700, 560) ) {
  _panel.addInspect( -700, 560, this, "bOther2" );
  return true;
}
if( !_publicOnly && modelElementContains(_x, _y, -700, 540) ) {
  _panel.addInspect( -700, 540, this, "bCommunity2" );
  return true;
}
if( !_publicOnly && modelElementContains(_x, _y, -700, 620) ) {
  _panel.addInspect( -700, 620, this, "bClinic2" );
  return true;
}
if( !_publicOnly && modelElementContains(_x, _y, -700, 600) ) {
  _panel.addInspect( -700, 600, this, "bMaternity2" );
  return true;
}
if( !_publicOnly && modelElementContains(_x, _y, -700, 580) ) {
  _panel.addInspect( -700, 580, this, "bHealthCenter2" );
  return true;
}
if( !_publicOnly && modelElementContains(_x, _y, -700, 720) ) {
  _panel.addInspect( -700, 720, this, "bBobs2" );
  return true;
}
if( !_publicOnly && modelElementContains(_x, _y, -700, 700) ) {
  _panel.addInspect( -700, 700, this, "bFees2" );
  return true;
}
if( !_publicOnly && modelElementContains(_x, _y, -700, 680) ) {
  _panel.addInspect( -700, 680, this, "bQuality2" );
  return true;
}
if( !_publicOnly && modelElementContains(_x, _y, -700, 660) ) {
  _panel.addInspect( -700, 660, this, "bDistance2" );
  return true;
}
if( !_publicOnly && modelElementContains(_x, _y, -560, 500) ) {
  _panel.addInspect( -560, 500, this, "bPoorest" );
  return true;
}
if( !_publicOnly && modelElementContains(_x, _y, -560, 540) ) {
  _panel.addInspect( -560, 540, this, "bMiddle" );
  return true;
```

```
}
if( !_publicOnly && modelElementContains(_x, _y, -560, 520) ) {
  _panel.addInspect( -560, 520, this, "bPoor" );
  return true;
}
if( !_publicOnly && modelElementContains(_x, _y, -560, 600) ) {
  _panel.addInspect( -560, 600, this, "bIntercept" );
  return true;
}
if( !_publicOnly && modelElementContains(_x, _y, -560, 580) ) {
  _panel.addInspect( -560, 580, this, "bRichest" );
  return true;
}
if( !_publicOnly && modelElementContains(_x, _y, -560, 560) ) {
  _panel.addInspect( -560, 560, this, "bRich" );
  return true;
}
if( !_publicOnly && modelElementContains(_x, _y, -560, 680) ) {
  _panel.addInspect( -560, 680, this, "bIlliterate" );
  return true;
}
if( !_publicOnly && modelElementContains(_x, _y, -560, 660) ) {
  _panel.addInspect( -560, 660, this, "bBlind" );
  return true;
}
if( !_publicOnly && modelElementContains(_x, _y, -560, 640) ) {
  _panel.addInspect( -560, 640, this, "bPrimip" );
  return true;
}
if( !_publicOnly && modelElementContains(_x, _y, -480, 500) ) {
  _panel.addInspect( -480, 500, this, "bUrban" );
  return true;
}
if( !_publicOnly && modelElementContains(_x, _y, -480, 540) ) {
  _panel.addInspect( -480, 540, this, "bSpouseAge" );
  return true;
}
if( !_publicOnly && modelElementContains(_x, _y, -480, 520) ) {
  _panel.addInspect( -480, 520, this, "bAge" );
  return true;
}
if( !_publicOnly && modelElementContains(_x, _y, -480, 600) ) {
  _panel.addInspect( -480, 600, this, "bUnwanted" );
  return true;
}
if( !_publicOnly && modelElementContains(_x, _y, -480, 580) ) {
  _panel.addInspect( -480, 580, this, "bTwins" );
  return true;
}
if( !_publicOnly && modelElementContains(_x, _y, -480, 560) ) {
  _panel.addInspect( -480, 560, this, "bEducSec" );
  return true;
}
if( !_publicOnly && modelElementContains(_x, _y, -480, 680) ) {
  _panel.addInspect( -480, 680, this, "bCSPlanned" );
  return true;
}
if( !_publicOnly && modelElementContains(_x, _y, -480, 660) ) {
  _panel.addInspect( -480, 660, this, "bRisk" );
  return true;
}
if( !_publicOnly && modelElementContains(_x, _y, -480, 640) ) {
  _panel.addInspect( -480, 640, this, "bANC4" );
```

```
      return true;
    }
    if( !_publicOnly && modelElementContains(_x, _y, -160, 100) ) {
      _panel.addInspect( -160, 100, this, "vAgentID" );
      return true;
    }
    if( !_publicOnly && modelElementContains(_x, _y, -280, 190) ) {
      _panel.addInspect( -280, 190, this, "allowFees" );
      return true;
    }
    if( !_publicOnly && modelElementContains(_x, _y, -280, 220) ) {
      _panel.addInspect( -280, 220, this, "allowAll" );
      return true;
    }
    if( !_publicOnly && modelElementContains(_x, _y, -280, 160) ) {
      _panel.addInspect( -280, 160, this, "QI" );
      return true;
    }
    return false;
  }

  @AnyLogicInternalCodegenAPI
  private boolean onClickModelAt_CollectionVariables_xjal( Panel _panel, double _x,
double _y, int _clickCount, boolean _publicOnly, boolean _isSuperClass ) {
    if( !_publicOnly && modelElementContains(_x, _y, -160, 260) ) {
      _panel.addInspect( -160, 260, this, "hospitalList" );
      return true;
    }
    if( !_publicOnly && modelElementContains(_x, _y, -160, 300) ) {
      _panel.addInspect( -160, 300, this, "allowedList" );
      return true;
    }
    return false;
  }

  @AnyLogicInternalCodegenAPI
  private boolean onClickModelAt_Events_xjal( Panel _panel, double _x, double _y, int
_clickCount, boolean _publicOnly, boolean _isSuperClass ) {
    if( !_publicOnly && modelElementContains(_x, _y, -160, 360) ) {
      _panel.addInspect( -160, 360, this, "classesAssigned" );
      return true;
    }
    return false;
  }

  @AnyLogicInternalCodegenAPI
  private boolean onClickModelAt_DataElements_xjal( Panel _panel, double _x, double
_y, int _clickCount, boolean _publicOnly, boolean _isSuperClass ) {
    if( !_publicOnly && modelElementContains(_x, _y, 1350, 620) ) {
      _panel.addInspect( 1350, 620, this, "uDiff" );
      return true;
    }
    if( !_publicOnly && modelElementContains(_x, _y, 1300, 390) ) {
      _panel.addInspect( 1300, 390, this, "travelDistance" );
      return true;
    }
    if( !_publicOnly && modelElementContains(_x, _y, 1230, 160) ) {
      _panel.addInspect( 1230, 160, this, "selectedBobs" );
      return true;
    }
    return false;
  }
```

```
  @AnyLogicInternalCodegenAPI
  private boolean onClickModelAt_Outputs_xjal( Panel _panel, double _x, double _y, int
_clickCount, boolean _publicOnly, boolean _isSuperClass ) {
    if( !_publicOnly && modelElementContains(_x, _y, 750, 110) ) {
      _panel.addInspect( 750, 110, this, "travelTime" );
      return true;
    }
    if( !_publicOnly && modelElementContains(_x, _y, 750, 30) ) {
      _panel.addInspect( 750, 30, this, "popSize" );
      return true;
    }
    if( !_publicOnly && modelElementContains(_x, _y, 850, 30) ) {
      _panel.addInspect( 850, 30, this, "segment2" );
      return true;
    }
    if( !_publicOnly && modelElementContains(_x, _y, 950, 30) ) {
      _panel.addInspect( 950, 30, this, "urbanPct" );
      return true;
    }
    if( !_publicOnly && modelElementContains(_x, _y, 850, 110) ) {
      _panel.addInspect( 850, 110, this, "utilityDifference" );
      return true;
    }
    if( !_publicOnly && modelElementContains(_x, _y, 750, 70) ) {
      _panel.addInspect( 750, 70, this, "classAssigned" );
      return true;
    }
    if( !_publicOnly && modelElementContains(_x, _y, 980, 110) ) {
      _panel.addInspect( 980, 110, this, "avgBobs" );
      return true;
    }
    if( !_publicOnly && modelElementContains(_x, _y, 1070, 110) ) {
      _panel.addInspect( 1070, 110, this, "avgFees" );
      return true;
    }
    if( !_publicOnly && modelElementContains(_x, _y, 870, 70) ) {
      _panel.addInspect( 870, 70, this, "hospitalAssigned" );
      return true;
    }
    if( !_publicOnly && modelElementContains(_x, _y, 1050, 30) ) {
      _panel.addInspect( 1050, 30, this, "meanDistance" );
      return true;
    }
    if( !_publicOnly && modelElementContains(_x, _y, 1000, 70) ) {
      _panel.addInspect( 1000, 70, this, "dist60km" );
      return true;
    }
    if( !_publicOnly && modelElementContains(_x, _y, 1090, 70) ) {
      _panel.addInspect( 1090, 70, this, "distFurther" );
      return true;
    }
    if( !_publicOnly && modelElementContains(_x, _y, 1150, 110) ) {
      _panel.addInspect( 1150, 110, this, "meanBobs" );
      return true;
    }
    if( !_publicOnly && modelElementContains(_x, _y, 1190, 30) ) {
      _panel.addInspect( 1190, 30, this, "neonatalMortality" );
      return true;
    }
    if( !_publicOnly && modelElementContains(_x, _y, 1200, 70) ) {
      _panel.addInspect( 1200, 70, this, "csRate" );
      return true;
    }
```

```
    if( !_publicOnly && modelElementContains(_x, _y, 1300, 70) ) {
      _panel.addInspect( 1300, 70, this, "avgOOP" );
      return true;
    }
    if( !_publicOnly && modelElementContains(_x, _y, 550, 340) ) {
      _panel.addInspect( 550, 340, this, "catExpPoorest" );
      return true;
    }
    if( !_publicOnly && modelElementContains(_x, _y, 550, 540) ) {
      _panel.addInspect( 550, 540, this, "catExpTotal" );
      return true;
    }
    if( !_publicOnly && modelElementContains(_x, _y, 550, 500) ) {
      _panel.addInspect( 550, 500, this, "catExpRichest" );
      return true;
    }
    if( !_publicOnly && modelElementContains(_x, _y, 550, 460) ) {
      _panel.addInspect( 550, 460, this, "catExpRich" );
      return true;
    }
    if( !_publicOnly && modelElementContains(_x, _y, 550, 420) ) {
      _panel.addInspect( 550, 420, this, "catExpMiddle" );
      return true;
    }
    if( !_publicOnly && modelElementContains(_x, _y, 550, 380) ) {
      _panel.addInspect( 550, 380, this, "catExpPoor" );
      return true;
    }
    return false;
  }

  @Override
  @AnyLogicInternalCodegenAPI
  public boolean onClickModelAt( Panel _panel, double _x, double _y, int _clickCount,
boolean _publicOnly, boolean _isSuperClass ) {
    if ( onClickModelAt_EmbeddedObjects_xjal( _panel, _x, _y, _clickCount,
_publicOnly, _isSuperClass ) ) { return true; }
    if ( onClickModelAt_AgentLinks_xjal( _panel, _x, _y, _clickCount, _publicOnly,
_isSuperClass ) ) { return true; }
    if ( onClickModelAt_Connectivity_xjal( _panel, _x, _y, _clickCount, _publicOnly,
_isSuperClass ) ) { return true; }
    if ( onClickModelAt_Parameters_xjal( _panel, _x, _y, _clickCount, _publicOnly,
_isSuperClass ) ) { return true; }
    if ( onClickModelAt_PlainVariables_xjal( _panel, _x, _y, _clickCount, _publicOnly,
_isSuperClass ) ) { return true; }
    if ( onClickModelAt_CollectionVariables_xjal( _panel, _x, _y, _clickCount,
_publicOnly, _isSuperClass ) ) { return true; }
    if ( onClickModelAt_Events_xjal( _panel, _x, _y, _clickCount, _publicOnly,
_isSuperClass ) ) { return true; }
    if ( onClickModelAt_DataElements_xjal( _panel, _x, _y, _clickCount, _publicOnly,
_isSuperClass ) ) { return true; }
    if ( onClickModelAt_Outputs_xjal( _panel, _x, _y, _clickCount, _publicOnly,
_isSuperClass ) ) { return true; }
    return super.onClickModelAt( _panel, _x, _y, _clickCount, _publicOnly, true );
  }



  /**
   * Constructor
   */
  public Main( Engine engine, Agent owner, AgentList<? extends Main> ownerPopulation )
{
```

```
    super( engine, owner, ownerPopulation );
    instantiateBaseStructureThis_xjal();
  }

  @AnyLogicInternalCodegenAPI
  public void onOwnerChanged_xjal() {
    super.onOwnerChanged_xjal();
    setupReferences_xjal();
  }

  @AnyLogicInternalCodegenAPI
  public void instantiateBaseStructure_xjal() {
    super.instantiateBaseStructure_xjal();
    instantiateBaseStructureThis_xjal();
  }

  @AnyLogicInternalCodegenAPI
  private void instantiateBaseStructureThis_xjal() {
    setupReferences_xjal();
    distanceWTP.readFile();
    newClassAssignment.readFile();
  }

  @AnyLogicInternalCodegenAPI
  private void setupReferences_xjal() {
  }

  /**
   * Simple constructor. Please add created agent to some population by calling
goToPopulation() function
   */
  public Main() {
  }

  /**
   * Simple constructor. Please add created agent to some population by calling
goToPopulation() function
   */
  public Main( boolean allowFeesParam, double centralHosp1, double distance1, double
clinic1, double maternity1, double healthCenter1, double otherHosp1, double
communityHosp1, double districtHosp1, double bobs1, double fees1, boolean
allowAllParam, double intercept, double centralHosp2, double distance2, double
clinic2, double maternity2, double healthCenter2, double otherHosp2, double
communityHosp2, double districtHosp2, double bobs2, double fees2, double distCenter,
double distRMS, double centralHosp1SE, double distance1SE, double clinic1SE, double
maternity1SE, double healthCenter1SE, double otherHosp1SE, double communityHosp1SE,
double districtHosp1SE, double bobs1SE, double fees1SE, double interceptSE, double
centralHosp2SE, double distance2SE, double clinic2SE, double maternity2SE, double
healthCenter2SE, double otherHosp2SE, double communityHosp2SE, double districtHosp2SE,
double bobs2SE, double fees2SE, double centralCS, double clinicCS, double maternityCS,
double healthCenterCS, double otherCS, double communityCS, double districtCS, double
centralCSSE, double clinicCSSE, double maternityCSSE, double healthCenterCSSE, double
otherCSSE, double communityCSSE, double districtCSSE, double centralNo, double
clinicNo, double maternityNo, double healthCenterNo, double otherNo, double
communityNo, double districtNo, double centralNoSE, double clinicNoSE, double
maternityNoSE, double healthCenterNoSE, double otherNoSE, double communityNoSE, double
districtNoSE, double central5, double clinic5, double maternity5, double
healthCenter5, double other5, double community5, double district5, double central5SE,
double clinic5SE, double maternity5SE, double healthCenter5SE, double other5SE, double
community5SE, double district5SE, double centralNo5, double clinicNo5, double
maternityNo5, double healthCenterNo5, double otherNo5, double communityNo5, double
districtNo5, double centralNo5SE, double clinicNo5SE, double maternityNo5SE, double
healthCenterNo5SE, double otherNo5SE, double communityNo5SE, double districtNo5SE,
```

```
double anc4, double unwanted, double risk, double urban, double twins, double educSec,
double spouseAge, double age, double csPlanned, double anc4SE, double unwantedSE,
double riskSE, double urbanSE, double twinsSE, double educSecSE, double spouseAgeSE,
double ageSE, double csPlannedSE, double QIParam, int strategy ) {
    markParametersAreSet();
    this.allowFeesParam = allowFeesParam;
    this.centralHosp1 = centralHosp1;
    this.distance1 = distance1;
    this.clinic1 = clinic1;
    this.maternity1 = maternity1;
    this.healthCenter1 = healthCenter1;
    this.otherHosp1 = otherHosp1;
    this.communityHosp1 = communityHosp1;
    this.districtHosp1 = districtHosp1;
    this.bobs1 = bobs1;
    this.fees1 = fees1;
    this.allowAllParam = allowAllParam;
    this.intercept = intercept;
    this.centralHosp2 = centralHosp2;
    this.distance2 = distance2;
    this.clinic2 = clinic2;
    this.maternity2 = maternity2;
    this.healthCenter2 = healthCenter2;
    this.otherHosp2 = otherHosp2;
    this.communityHosp2 = communityHosp2;
    this.districtHosp2 = districtHosp2;
    this.bobs2 = bobs2;
    this.fees2 = fees2;
    this.distCenter = distCenter;
    this.distRMS = distRMS;
    this.centralHosp1SE = centralHosp1SE;
    this.distance1SE = distance1SE;
    this.clinic1SE = clinic1SE;
    this.maternity1SE = maternity1SE;
    this.healthCenter1SE = healthCenter1SE;
    this.otherHosp1SE = otherHosp1SE;
    this.communityHosp1SE = communityHosp1SE;
    this.districtHosp1SE = districtHosp1SE;
    this.bobs1SE = bobs1SE;
    this.fees1SE = fees1SE;
    this.interceptSE = interceptSE;
    this.centralHosp2SE = centralHosp2SE;
    this.distance2SE = distance2SE;
    this.clinic2SE = clinic2SE;
    this.maternity2SE = maternity2SE;
    this.healthCenter2SE = healthCenter2SE;
    this.otherHosp2SE = otherHosp2SE;
    this.communityHosp2SE = communityHosp2SE;
    this.districtHosp2SE = districtHosp2SE;
    this.bobs2SE = bobs2SE;
    this.fees2SE = fees2SE;
    this.centralCS = centralCS;
    this.clinicCS = clinicCS;
    this.maternityCS = maternityCS;
    this.healthCenterCS = healthCenterCS;
    this.otherCS = otherCS;
    this.communityCS = communityCS;
    this.districtCS = districtCS;
    this.centralCSSE = centralCSSE;
    this.clinicCSSE = clinicCSSE;
    this.maternityCSSE = maternityCSSE;
    this.healthCenterCSSE = healthCenterCSSE;
    this.otherCSSE = otherCSSE;
```

```
this.communityCSSE = communityCSSE;
this.districtCSSE = districtCSSE;
this.centralNo = centralNo;
this.clinicNo = clinicNo;
this.maternityNo = maternityNo;
this.healthCenterNo = healthCenterNo;
this.otherNo = otherNo;
this.communityNo = communityNo;
this.districtNo = districtNo;
this.centralNoSE = centralNoSE;
this.clinicNoSE = clinicNoSE;
this.maternityNoSE = maternityNoSE;
this.healthCenterNoSE = healthCenterNoSE;
this.otherNoSE = otherNoSE;
this.communityNoSE = communityNoSE;
this.districtNoSE = districtNoSE;
this.central5 = central5;
this.clinic5 = clinic5;
this.maternity5 = maternity5;
this.healthCenter5 = healthCenter5;
this.other5 = other5;
this.community5 = community5;
this.district5 = district5;
this.central5SE = central5SE;
this.clinic5SE = clinic5SE;
this.maternity5SE = maternity5SE;
this.healthCenter5SE = healthCenter5SE;
this.other5SE = other5SE;
this.community5SE = community5SE;
this.district5SE = district5SE;
this.centralNo5 = centralNo5;
this.clinicNo5 = clinicNo5;
this.maternityNo5 = maternityNo5;
this.healthCenterNo5 = healthCenterNo5;
this.otherNo5 = otherNo5;
this.communityNo5 = communityNo5;
this.districtNo5 = districtNo5;
this.centralNo5SE = centralNo5SE;
this.clinicNo5SE = clinicNo5SE;
this.maternityNo5SE = maternityNo5SE;
this.healthCenterNo5SE = healthCenterNo5SE;
this.otherNo5SE = otherNo5SE;
this.communityNo5SE = communityNo5SE;
this.districtNo5SE = districtNo5SE;
this.anc4 = anc4;
this.unwanted = unwanted;
this.risk = risk;
this.urban = urban;
this.twins = twins;
this.educSec = educSec;
this.spouseAge = spouseAge;
this.age = age;
this.csPlanned = csPlanned;
this.anc4SE = anc4SE;
this.unwantedSE = unwantedSE;
this.riskSE = riskSE;
this.urbanSE = urbanSE;
this.twinsSE = twinsSE;
this.educSecSE = educSecSE;
this.spouseAgeSE = spouseAgeSE;
this.ageSE = ageSE;
this.csPlannedSE = csPlannedSE;
this.QIParam = QIParam;
```

```
    this.strategy = strategy;
  }

  /**
   * Creating embedded object instances
   */
  @AnyLogicInternalCodegenAPI
  private void instantiatePopulations_xjal() {
  }

  @Override
  @AnyLogicInternalCodegenAPI
  public void doCreate() {
    super.doCreate();
    // Creating embedded object instances
    instantiatePopulations_xjal();
    // Assigning initial values for plain variables
    setupPlainVariables_Main_xjal();
    // Dynamic initialization of persistent elements
    _createPersistentElementsAP0_xjal();
    presentation = new ShapeTopLevelPresentationGroup( Main.this, true, 0, 0, 0, 0 ,
rectangle1, rectangle, map, hospitals_presentation, people_presentation, agentID,
delLocText, text2, text3, mapLayer, text4, text5, delLocText1, travelDistText, text6,
text7, text8, text9, StdErrorText, MeanText1, rectangle2, text10, text11, MeanText2,
travelDistText1, rectangle3, StdErrorText1, text12, text13, delLocText2, text14,
text15, rectangle4, text, chart, uDiffHistogram, uDiffHistogram1, selectedBobsHist,
mortChart );
    // Creating contents for replicated shapes
    people_presentation.createShapes();
    hospitals_presentation.createShapes();
    // Creating embedded object instances
    instantiatePopulations_xjal();
    icon = new ShapeModelElementsGroup( Main.this, getElementProperty(
"malawi_recentralization_model_2017_06_04.Main.icon",
IElementDescriptor.MODEL_ELEMENT_DESCRIPTORS )  );
    icon.setIconOffsets( 0.0, 0.0 );
    // Environments setup
    setupSpace( map );
    disableSteps();
    setNetworkUserDefined();
        // Port connectors with non-replicated objects
    // Creating replicated embedded objects
    hospitals.setEnvironment( this );
    hospitals.fillFromTable( new TableInput( this,
(java.util.function.Supplier<ResultSet> & Serializable) () -> selectResultSet(
      "SELECT * FROM hospitals;"
)), null, true, false );
    people.setEnvironment( this );
    people.fillFromTable( new TableInput( this,
(java.util.function.Supplier<ResultSet> & Serializable) () -> selectResultSet(
      "SELECT * FROM pop_rand;"
)), null, true, false );
    setupInitialConditions_xjal( Main.class );
  }

  @AnyLogicInternalCodegenAPI
  public void setupExt_xjal(AgentExtension _ext) {
    // Agent properties setup
    if ( _ext instanceof ExtAgentWithSpatialMetrics && _ext instanceof
ExtWithSpaceType ) {
      double _value;
      _value =
```

```
;
      ((ExtAgentWithSpatialMetrics) _ext).setSpeed( _value, MPS );
    }
  }

  @Override
  @AnyLogicInternalCodegenAPI
  public void doStart() {
    super.doStart();
    classesAssigned.start();
    for (Agent embeddedObject : hospitals){
      embeddedObject.start();
    }
    for (Agent embeddedObject : people){
      embeddedObject.start();
    }
  }

  @AnyLogicInternalCodegenAPI
  public void onStartup() {
    super.onStartup();

update_popSize();


// Assign parameters we don't have room to declare graphically

double poor = (double) selectFrom(parameters)
       .where(parameters.parameter.eq("Poor"))
       .uniqueResult(parameters.starting_value);
double poorSE = (double) selectFrom(parameters)
       .where(parameters.parameter.eq("Poor"))
       .uniqueResult(parameters.se);

double middle = (double) selectFrom(parameters)
       .where(parameters.parameter.eq("Middle"))
       .uniqueResult(parameters.starting_value);
double middleSE = (double) selectFrom(parameters)
       .where(parameters.parameter.eq("Middle"))
       .uniqueResult(parameters.se);

double rich = (double) selectFrom(parameters)
       .where(parameters.parameter.eq("Rich"))
       .uniqueResult(parameters.starting_value);
double richSE = (double) selectFrom(parameters)
       .where(parameters.parameter.eq("Rich"))
       .uniqueResult(parameters.se);

double richest = (double) selectFrom(parameters)
       .where(parameters.parameter.eq("Richest"))
       .uniqueResult(parameters.starting_value);
double richestSE = (double) selectFrom(parameters)
       .where(parameters.parameter.eq("Richest"))
       .uniqueResult(parameters.se);

double primip = (double) selectFrom(parameters)
       .where(parameters.parameter.eq("primip"))
       .uniqueResult(parameters.starting_value);
double primipSE = (double) selectFrom(parameters)
       .where(parameters.parameter.eq("primip"))
       .uniqueResult(parameters.se);

double blind = (double) selectFrom(parameters)
```

```
        .where(parameters.parameter.eq("blind"))
        .uniqueResult(parameters.starting_value);
double blindSE = (double) selectFrom(parameters)
        .where(parameters.parameter.eq("blind"))
        .uniqueResult(parameters.se);

double illiterate = (double) selectFrom(parameters)
        .where(parameters.parameter.eq("illiterate"))
        .uniqueResult(parameters.starting_value);
double illiterateSE = (double) selectFrom(parameters)
        .where(parameters.parameter.eq("illiterate"))
        .uniqueResult(parameters.se);


//Draw parameters
        Random r = new MersenneTwisterRNG();  //from uncommon maths package (found in
the /Mark/Models folder)
        bCentral1 = 0.0;
        bCentral2 = 0.0;

        bDistrict1 = new GaussianGenerator(districtHosp1,    districtHosp1SE,
        r).nextValue();  //from uncommon maths package (found in the /Mark/Models
folder)
        bCommunity1 =       new GaussianGenerator(communityHosp1,   communityHosp1SE,
        r).nextValue();
        bOther1 =           new GaussianGenerator(otherHosp1,              otherHosp1SE,
               r).nextValue();
        bHealthCenter1 = new GaussianGenerator(healthCenter1,       healthCenter1SE,
        r).nextValue();
        bMaternity1 =       new GaussianGenerator(maternity1,              maternity1SE,
               r).nextValue();
        bClinic1 =          new GaussianGenerator(clinic1,                 clinic1SE,
               r).nextValue();

        bDistrict2 = new GaussianGenerator(districtHosp2,    districtHosp2SE,
        r).nextValue();
        bCommunity2 =       new GaussianGenerator(communityHosp2,   communityHosp2SE,
        r).nextValue();
        bOther2 =           new GaussianGenerator(otherHosp2,              otherHosp2SE,
               r).nextValue();
        bHealthCenter2 = new GaussianGenerator(healthCenter2,       healthCenter2SE,
        r).nextValue();
        bMaternity2 =       new GaussianGenerator(maternity2,              maternity2SE,
               r).nextValue();
        bClinic2 =          new GaussianGenerator(clinic2,                 clinic2SE,
               r).nextValue();

        bDistance1 = new GaussianGenerator(distance1, distance1SE,  r).nextValue();
        bBobs1 =            new GaussianGenerator(bobs1,            bobs1SE,
        r).nextValue();
        bFees1 =            new GaussianGenerator(fees1,            fees1SE,
        r).nextValue();

        bDistance2 = new GaussianGenerator(distance2, distance2SE,  r).nextValue();
        bBobs2 =            new GaussianGenerator(bobs2,            bobs2SE,
        r).nextValue();
        bFees2 =            new GaussianGenerator(fees2,            fees2SE,
        r).nextValue();

        bPoorest =          0.0;
        bPoor =             new GaussianGenerator(poor,             poorSE,
        r).nextValue();
```

```
	bMiddle =		new GaussianGenerator(middle,		middleSE,
	r).nextValue();
	bRich =			new GaussianGenerator(rich,		richSE,
	r).nextValue();
	bRichest =		new GaussianGenerator(richest,		richestSE,
	r).nextValue();
	bIntercept = new GaussianGenerator(intercept, interceptSE, r).nextValue();

	bBlind =		new GaussianGenerator(blind,		blindSE,
	r).nextValue();
	bIlliterate =		new GaussianGenerator(illiterate,	illiterateSE,
	r).nextValue();
	bPrimip =		new GaussianGenerator(primip,		primipSE,
	r).nextValue();

	bUrban =		new GaussianGenerator(urban,		urbanSE,
	r).nextValue();
	bAge =				new GaussianGenerator(age,		ageSE,
	r).nextValue();
	bSpouseAge = new GaussianGenerator(spouseAge, spouseAgeSE, r).nextValue();
	bEducSec =		new GaussianGenerator(educSec,		educSecSE,
	r).nextValue();
	bTwins =		new GaussianGenerator(twins,		twinsSE,
	r).nextValue();
	bUnwanted =  new GaussianGenerator(unwanted,  unwantedSE,  r).nextValue();

	bANC4 =				new GaussianGenerator(anc4,		anc4SE,
	r).nextValue();
	bRisk =			new GaussianGenerator(risk,			riskSE,
		r).nextValue();
	bCSPlanned = new GaussianGenerator(csPlanned, csPlannedSE, r).nextValue();


/*
//Non-stochastic
bCentral1 = 0.0;
	bCentral2 = 0.0;

	bDistrict1 = districtHosp1;
	bCommunity1 =		communityHosp1;
	bOther1 =		otherHosp1;
	bHealthCenter1 = healthCenter1;
	bMaternity1 =		maternity1;
	bClinic1 =		clinic1;

	bDistrict2 = districtHosp2;
	bCommunity2 =		communityHosp2;
	bOther2 =		otherHosp2;
	bHealthCenter2 = healthCenter2;
	bMaternity2 =		maternity2;
	bClinic2 =		clinic2;

	bDistance1 = distance1;
	bBobs1 =		bobs1;
	bFees1 =		fees1;

	bDistance2 = distance2;
	bBobs2 =		bobs2;
	bFees2 =		fees2;

	bPoorest =		0.0;
	bPoor =			poor;
	bMiddle =		middle;
```

```
        bRich =             rich;
        bRichest =          richest;
        bIntercept = intercept;

        bBlind =            blind;
        bIlliterate =       illiterate;
        bPrimip =           primip;

        bUrban =            urban;
        bAge =                      age;
        bSpouseAge = spouseAge;
        bEducSec =          educSec;
        bTwins =            twins;
        bUnwanted =  unwanted;

        bANC4 =                     anc4;
        bRisk =             risk;
        bCSPlanned = csPlanned;
        */

        //bDistrict1 = (r.nextGaussian() + districtHosp1) * districtHosp1SE;

//System.out.println("Poor\tPoorSE\tbPoor\tMiddle\tMiddleSE\tbMiddle\tRich\tRichSE\tbR
ich\tRichest\tRichestSE\tbRichest");
//System.out.println("Poor: " + poor + "\nPoorSE: " + poorSE + "\nbPoor: " + bPoor +
"\n"
//                                + "Middle: " + middle + "\nMiddleSE: " + middleSE +
"\nbMiddle: " + bMiddle + "\n"
//                                + "Rich: " + rich + "\nRichSE: " + richSE +
"\nbRich: " + bRich + "\n"
//                                + "Richest: " + richest + "\nRichestSE: " +
richestSE + "\nbRichest: " + bRichest);

/*newClassAssignment.setCellValue(bIntercept, "Sheet1", 2, 2);
newClassAssignment.setCellValue(bBlind,       "Sheet1", 2, 5);
newClassAssignment.setCellValue(bPrimip,      "Sheet1", 2, 6);
newClassAssignment.setCellValue(bIlliterate,"Sheet1", 2, 7);
newClassAssignment.setCellValue(bUrban,       "Sheet1", 2, 8);
newClassAssignment.setCellValue(bAge,         "Sheet1", 2, 9);
newClassAssignment.setCellValue(bSpouseAge, "Sheet1", 2, 10);
newClassAssignment.setCellValue(bEducSec,     "Sheet1", 2, 11);
newClassAssignment.setCellValue(bTwins,       "Sheet1", 2, 12);
newClassAssignment.setCellValue(bUnwanted,    "Sheet1", 2, 13);
newClassAssignment.setCellValue(bANC4,        "Sheet1", 2, 14);
newClassAssignment.setCellValue(bRisk,        "Sheet1", 2, 15);
newClassAssignment.setCellValue(bCSPlanned, "Sheet1", 2, 16);*/

//Set experiment
// Run 2-201: Base case
// Run 202-401: Centralized
// Run 402-601: Centralized, no fees
// Run 602-801: Centralized, QI = 1
// Run 802-1001: Centralized, no fees, QI = 1

//Moved to the Parameters Variation experiment, parameters section
//allowAll = (iterNumber < 21);
//allowFees = ((iterNumber < 41) | (iterNumber >= 61 & iterNumber < 81));
//QI = (iterNumber > 61) ? 1 : 0;



//System.out.println((bDistrict1/districtHosp1SE - districtHosp1) + "\t" +
(bDistrict2/districtHosp2SE - districtHosp2));
```

```
//System.out.println("Agent\tClass\tHospital\tType\tbType\tDistance\tBobs\tFees\tQ\tU\
tP\tCDF\tR\tSelected Hospital");
//System.out.println("Start time = " + System.currentTimeMillis());
//System.out.println("Agent\tClass\tSelected\tdist\tspa\tbobs\tfees\tQ\tU\tP\tOptimal\
tdist\tspa\tbobs\tfees\tQ\tU\tP\tuDiff");
  }

  /**
   * Assigning initial values for plain variables<br>
   * <em>This method isn't designed to be called by user and may be removed in future
releases.</em>
   */
  @AnyLogicInternalCodegenAPI
  public void setupPlainVariables_xjal() {
    setupPlainVariables_Main_xjal();
  }

  /**
   * Assigning initial values for plain variables<br>
   * <em>This method isn't designed to be called by user and may be removed in future
releases.</em>
   */
  @AnyLogicInternalCodegenAPI
  private void setupPlainVariables_Main_xjal() {
    vAgentID =
0
;
    allowFees =
allowFeesParam
;
    allowAll =
allowAllParam
;
    QI =
0
;
  }

  // User API -----------------------------------------------------
  @AnyLogicInternalCodegenAPI
  static LinkToAgentAnimationSettings _connections_commonAnimationSettings_xjal = new
LinkToAgentAnimationSettingsImpl( false, black, 1.0, LINE_STYLE_SOLID, ARROW_NONE, 0.0
);

  public LinkToAgentCollection<Agent, Agent> connections = new
LinkToAgentStandardImpl<Agent, Agent>(this,
_connections_commonAnimationSettings_xjal);
  @Override
  public LinkToAgentCollection<? extends Agent, ? extends Agent>
getLinkToAgentStandard_xjal() {
    return connections;
  }


  @AnyLogicInternalCodegenAPI
  public void drawLinksToAgents(boolean _underAgents_xjal, LinkToAgentAnimator
_animator_xjal) {
    super.drawLinksToAgents(_underAgents_xjal, _animator_xjal);
    if ( _underAgents_xjal ) {
      _animator_xjal.drawLink( this, connections, true, true );
    }
  }
```

```
  public List<Object> getEmbeddedObjects() {
    List<Object> list = super.getEmbeddedObjects();
    if (list == null) {
      list = new LinkedList<Object>();
    }
    list.add( hospitals );
    list.add( people );
    return list;
  }

  public AgentList<? extends Main> getPopulation() {
    return (AgentList<? extends Main>) super.getPopulation();
  }

  public List<? extends Main> agentsInRange( double distance ) {
    return (List<? extends Main>) super.agentsInRange( distance );
  }

  // Reaction on changes -------------------------------------
  public void onChange() {
    super.onChange();
    classesAssigned.onChange();
  }

  @AnyLogicInternalCodegenAPI
  public void onDestroy() {
    classesAssigned.onDestroy();
    for (Agent _item : hospitals) {
      _item.onDestroy();
    }
    for (Agent _item : people) {
      _item.onDestroy();
    }
    distanceWTP.writeFile();
    newClassAssignment.writeFile();
    map.destroy();
  // Analysis Data Elements
    uDiff.destroyUpdater_xjal();
    travelDistance.destroyUpdater_xjal();
    selectedBobs.destroyUpdater_xjal();
    logToDB( uDiff, "uDiff" );
    logToDB( travelDistance, "travelDistance" );
    logToDB( selectedBobs, "selectedBobs" );
    super.onDestroy();
  }

  @AnyLogicInternalCodegenAPI
  @Override
  public void doFinish() {
    for (Agent _item : hospitals) {
      _item.doFinish();
    }
    super.doFinish();
    for (Agent _item : people) {
      _item.doFinish();
    }
    super.doFinish();
  }



}
```

```java
package malawi_recentralization_model_2017_06_04;

import java.io.Serializable;
import java.sql.Connection;
import java.sql.SQLException;
import java.util.ArrayDeque;
import java.util.ArrayList;
import java.util.Arrays;
import java.util.Calendar;
import java.util.Collection;
import java.util.Collections;
import java.util.Comparator;
import java.util.Currency;
import java.util.Date;
import java.util.Enumeration;
import java.util.HashMap;
import java.util.HashSet;
import java.util.Hashtable;
import java.util.Iterator;
import java.util.LinkedHashMap;
import java.util.LinkedHashSet;
import java.util.LinkedList;
import java.util.List;
import java.util.ListIterator;
import java.util.Locale;
import java.util.Map;
import java.util.PriorityQueue;
import java.util.Random;
import java.util.Set;
import java.util.SortedMap;
import java.util.SortedSet;
import java.util.Stack;
import java.util.Timer;
import java.util.TreeMap;
import java.util.TreeSet;
import java.util.Vector;
import java.awt.Color;
import java.awt.Font;
import java.awt.Graphics2D;
import java.awt.geom.AffineTransform;
import com.anylogic.engine.connectivity.ResultSet;
import com.anylogic.engine.connectivity.Statement;
import com.anylogic.engine.elements.*;
import com.anylogic.engine.markup.Network;
import com.anylogic.engine.Position;
import com.anylogic.engine.markup.PedFlowStatistics;
import com.anylogic.engine.markup.DensityMap;


import static java.lang.Math.*;
import static com.anylogic.engine.UtilitiesArray.*;
import static com.anylogic.engine.UtilitiesCollection.*;
import static com.anylogic.engine.presentation.UtilitiesColor.*;
import static com.anylogic.engine.HyperArray.*;

import com.anylogic.engine.*;
import com.anylogic.engine.analysis.*;
import com.anylogic.engine.connectivity.*;
import com.anylogic.engine.database.*;
import com.anylogic.engine.gis.*;
```

```
import com.anylogic.engine.markup.*;
import com.anylogic.engine.presentation.*;

import com.mysema.query.Tuple;
import com.mysema.query.sql.SQLBindings;
import static malawi_recentralization_model_2017_06_04.DBDescriptor.*;

import java.awt.geom.Arc2D;

import org.uncommons.maths.random.MersenneTwisterRNG;
import org.uncommons.maths.random.GaussianGenerator;

public class Hospital extends Agent
{
  // Parameters

  public
String  id;

  /**
   * Returns default value for parameter <code>id</code>.
   * <i>This method should not be called by user</i>
   */
  @AnyLogicInternalCodegenAPI
  public String _id_DefaultValue_xjal() {
    final Hospital self = this;
    return null;
  }

  public void set_id( String id ) {
    if (id == this.id) {
      return;
    }
    String _oldValue_xjal = this.id;
    this.id = id;
    onChange_id_xjal( _oldValue_xjal );
    onChange();
  }

  /**
   * Calls "On change" action for parameter id.<br>
   * Note that 'oldValue' in that action will be unavailable if this method is called
by user
   * (current parameter value will be passed as 'oldValue').<br>
   * Please call <code>set_id()</code> method instead.
   */
  protected void onChange_id() {
    onChange_id_xjal( id );
  }

  @AnyLogicInternalCodegenAPI
  protected void onChange_id_xjal( String oldValue ) {
  }


  public
String  type;

  /**
   * Returns default value for parameter <code>type</code>.
   * <i>This method should not be called by user</i>
   */
  @AnyLogicInternalCodegenAPI
```

```
  public String _type_DefaultValue_xjal() {
    final Hospital self = this;
    return null;
  }

  public void set_type( String type ) {
    if (type == this.type) {
      return;
    }
    String _oldValue_xjal = this.type;
    this.type = type;
    onChange_type_xjal( _oldValue_xjal );
    onChange();
  }

  /**
   * Calls "On change" action for parameter type.<br>
   * Note that 'oldValue' in that action will be unavailable if this method is called
by user
   * (current parameter value will be passed as 'oldValue').<br>
   * Please call <code>set_type()</code> method instead.
   */
  protected void onChange_type() {
    onChange_type_xjal( type );
  }

  @AnyLogicInternalCodegenAPI
  protected void onChange_type_xjal( String oldValue ) {
  }


  public
String  management;

  /**
   * Returns default value for parameter <code>management</code>.
   * <i>This method should not be called by user</i>
   */
  @AnyLogicInternalCodegenAPI
  public String _management_DefaultValue_xjal() {
    final Hospital self = this;
    return null;
  }

  public void set_management( String management ) {
    if (management == this.management) {
      return;
    }
    String _oldValue_xjal = this.management;
    this.management = management;
    onChange_management_xjal( _oldValue_xjal );
    onChange();
  }

  /**
   * Calls "On change" action for parameter management.<br>
   * Note that 'oldValue' in that action will be unavailable if this method is called
by user
   * (current parameter value will be passed as 'oldValue').<br>
   * Please call <code>set_management()</code> method instead.
   */
  protected void onChange_management() {
    onChange_management_xjal( management );
```

```
  }

  @AnyLogicInternalCodegenAPI
  protected void onChange_management_xjal( String oldValue ) {
  }


  public
boolean  urban;

  /**
   * Returns default value for parameter <code>urban</code>.
   * <i>This method should not be called by user</i>
   */
  @AnyLogicInternalCodegenAPI
  public boolean _urban_DefaultValue_xjal() {
    final Hospital self = this;
    return false;
  }

  public void set_urban( boolean urban ) {
    if (urban == this.urban) {
      return;
    }
    boolean _oldValue_xjal = this.urban;
    this.urban = urban;
    onChange_urban_xjal( _oldValue_xjal );
    onChange();
  }

  /**
   * Calls "On change" action for parameter urban.<br>
   * Note that 'oldValue' in that action will be unavailable if this method is called
by user
   * (current parameter value will be passed as 'oldValue').<br>
   * Please call <code>set_urban()</code> method instead.
   */
  protected void onChange_urban() {
    onChange_urban_xjal( urban );
  }

  @AnyLogicInternalCodegenAPI
  protected void onChange_urban_xjal( boolean oldValue ) {
  }


  public
double  latitude;

  /**
   * Returns default value for parameter <code>latitude</code>.
   * <i>This method should not be called by user</i>
   */
  @AnyLogicInternalCodegenAPI
  public double _latitude_DefaultValue_xjal() {
    final Hospital self = this;
    return 0.0;
  }

  public void set_latitude( double latitude ) {
    if (latitude == this.latitude) {
      return;
    }
```

```
    double _oldValue_xjal = this.latitude;
    this.latitude = latitude;
    onChange_latitude_xjal( _oldValue_xjal );
    onChange();
  }

  /**
   * Calls "On change" action for parameter latitude.<br>
   * Note that 'oldValue' in that action will be unavailable if this method is called
by user
   * (current parameter value will be passed as 'oldValue').<br>
   * Please call <code>set_latitude()</code> method instead.
   */
  protected void onChange_latitude() {
    onChange_latitude_xjal( latitude );
  }

  @AnyLogicInternalCodegenAPI
  protected void onChange_latitude_xjal( double oldValue ) {
  }


  public
double  longitude;

  /**
   * Returns default value for parameter <code>longitude</code>.
   * <i>This method should not be called by user</i>
   */
  @AnyLogicInternalCodegenAPI
  public double _longitude_DefaultValue_xjal() {
    final Hospital self = this;
    return 0.0;
  }

  public void set_longitude( double longitude ) {
    if (longitude == this.longitude) {
      return;
    }
    double _oldValue_xjal = this.longitude;
    this.longitude = longitude;
    onChange_longitude_xjal( _oldValue_xjal );
    onChange();
  }

  /**
   * Calls "On change" action for parameter longitude.<br>
   * Note that 'oldValue' in that action will be unavailable if this method is called
by user
   * (current parameter value will be passed as 'oldValue').<br>
   * Please call <code>set_longitude()</code> method instead.
   */
  protected void onChange_longitude() {
    onChange_longitude_xjal( longitude );
  }

  @AnyLogicInternalCodegenAPI
  protected void onChange_longitude_xjal( double oldValue ) {
  }


  public
double  bobsCounterfactual;
```

```
  /**
   * Returns default value for parameter <code>bobsCounterfactual</code>.
   * <i>This method should not be called by user</i>
   */
  @AnyLogicInternalCodegenAPI
  public double _bobsCounterfactual_DefaultValue_xjal() {
    final Hospital self = this;
    return 0.0;
  }

  public void set_bobsCounterfactual( double bobsCounterfactual ) {
    if (bobsCounterfactual == this.bobsCounterfactual) {
      return;
    }
    double _oldValue_xjal = this.bobsCounterfactual;
    this.bobsCounterfactual = bobsCounterfactual;
    onChange_bobsCounterfactual_xjal( _oldValue_xjal );
    onChange();
  }

  /**
   * Calls "On change" action for parameter bobsCounterfactual.<br>
   * Note that 'oldValue' in that action will be unavailable if this method is called
by user
   * (current parameter value will be passed as 'oldValue').<br>
   * Please call <code>set_bobsCounterfactual()</code> method instead.
   */
  protected void onChange_bobsCounterfactual() {
    onChange_bobsCounterfactual_xjal( bobsCounterfactual );
  }

  @AnyLogicInternalCodegenAPI
  protected void onChange_bobsCounterfactual_xjal( double oldValue ) {
  }


  public
double  q;

  /**
   * Returns default value for parameter <code>q</code>.
   * <i>This method should not be called by user</i>
   */
  @AnyLogicInternalCodegenAPI
  public double _q_DefaultValue_xjal() {
    final Hospital self = this;
    return 0.0;
  }

  public void set_q( double q ) {
    if (q == this.q) {
      return;
    }
    double _oldValue_xjal = this.q;
    this.q = q;
    onChange_q_xjal( _oldValue_xjal );
    onChange();
  }

  /**
   * Calls "On change" action for parameter q.<br>
```

```
   * Note that 'oldValue' in that action will be unavailable if this method is called
by user
   * (current parameter value will be passed as 'oldValue').<br>
   * Please call <code>set_q()</code> method instead.
   */
  protected void onChange_q() {
    onChange_q_xjal( q );
  }

  @AnyLogicInternalCodegenAPI
  protected void onChange_q_xjal( double oldValue ) {
  }


  public
int  feesCounterfactual;

  /**
   * Returns default value for parameter <code>feesCounterfactual</code>.
   * <i>This method should not be called by user</i>
   */
  @AnyLogicInternalCodegenAPI
  public int _feesCounterfactual_DefaultValue_xjal() {
    final Hospital self = this;
    return 0;
  }

  public void set_feesCounterfactual( int feesCounterfactual ) {
    if (feesCounterfactual == this.feesCounterfactual) {
      return;
    }
    int _oldValue_xjal = this.feesCounterfactual;
    this.feesCounterfactual = feesCounterfactual;
    onChange_feesCounterfactual_xjal( _oldValue_xjal );
    onChange();
  }

  /**
   * Calls "On change" action for parameter feesCounterfactual.<br>
   * Note that 'oldValue' in that action will be unavailable if this method is called
by user
   * (current parameter value will be passed as 'oldValue').<br>
   * Please call <code>set_feesCounterfactual()</code> method instead.
   */
  protected void onChange_feesCounterfactual() {
    onChange_feesCounterfactual_xjal( feesCounterfactual );
  }

  @AnyLogicInternalCodegenAPI
  protected void onChange_feesCounterfactual_xjal( int oldValue ) {
  }


  public
double  distance;

  /**
   * Returns default value for parameter <code>distance</code>.
   * <i>This method should not be called by user</i>
   */
  @AnyLogicInternalCodegenAPI
  public double _distance_DefaultValue_xjal() {
    final Hospital self = this;
```

```
    return 0.0;
  }

  public void set_distance( double distance ) {
    if (distance == this.distance) {
      return;
    }
    double _oldValue_xjal = this.distance;
    this.distance = distance;
    onChange_distance_xjal( _oldValue_xjal );
    onChange();
  }

  /**
   * Calls "On change" action for parameter distance.<br>
   * Note that 'oldValue' in that action will be unavailable if this method is called
by user
   * (current parameter value will be passed as 'oldValue').<br>
   * Please call <code>set_distance()</code> method instead.
   */
  protected void onChange_distance() {
    onChange_distance_xjal( distance );
  }

  @AnyLogicInternalCodegenAPI
  protected void onChange_distance_xjal( double oldValue ) {
  }


  public
boolean  hospital;

  /**
   * Returns default value for parameter <code>hospital</code>.
   * <i>This method should not be called by user</i>
   */
  @AnyLogicInternalCodegenAPI
  public boolean _hospital_DefaultValue_xjal() {
    final Hospital self = this;
    return false;
  }

  public void set_hospital( boolean hospital ) {
    if (hospital == this.hospital) {
      return;
    }
    boolean _oldValue_xjal = this.hospital;
    this.hospital = hospital;
    onChange_hospital_xjal( _oldValue_xjal );
    onChange();
  }

  /**
   * Calls "On change" action for parameter hospital.<br>
   * Note that 'oldValue' in that action will be unavailable if this method is called
by user
   * (current parameter value will be passed as 'oldValue').<br>
   * Please call <code>set_hospital()</code> method instead.
   */
  protected void onChange_hospital() {
    onChange_hospital_xjal( hospital );
  }
```

```
  @AnyLogicInternalCodegenAPI
  protected void onChange_hospital_xjal( boolean oldValue ) {
  }


  public
boolean  cs;

  /**
   * Returns default value for parameter <code>cs</code>.
   * <i>This method should not be called by user</i>
   */
  @AnyLogicInternalCodegenAPI
  public boolean _cs_DefaultValue_xjal() {
    final Hospital self = this;
    return false;
  }

  public void set_cs( boolean cs ) {
    if (cs == this.cs) {
      return;
    }
    boolean _oldValue_xjal = this.cs;
    this.cs = cs;
    onChange_cs_xjal( _oldValue_xjal );
    onChange();
  }

  /**
   * Calls "On change" action for parameter cs.<br>
   * Note that 'oldValue' in that action will be unavailable if this method is called
by user
   * (current parameter value will be passed as 'oldValue').<br>
   * Please call <code>set_cs()</code> method instead.
   */
  protected void onChange_cs() {
    onChange_cs_xjal( cs );
  }

  @AnyLogicInternalCodegenAPI
  protected void onChange_cs_xjal( boolean oldValue ) {
  }


  public
boolean  bemonc;

  /**
   * Returns default value for parameter <code>bemonc</code>.
   * <i>This method should not be called by user</i>
   */
  @AnyLogicInternalCodegenAPI
  public boolean _bemonc_DefaultValue_xjal() {
    final Hospital self = this;
    return false;
  }

  public void set_bemonc( boolean bemonc ) {
    if (bemonc == this.bemonc) {
      return;
    }
    boolean _oldValue_xjal = this.bemonc;
    this.bemonc = bemonc;
```

```
    onChange_bemonc_xjal( _oldValue_xjal );
    onChange();
  }

  /**
   * Calls "On change" action for parameter bemonc.<br>
   * Note that 'oldValue' in that action will be unavailable if this method is called
by user
   * (current parameter value will be passed as 'oldValue').<br>
   * Please call <code>set_bemonc()</code> method instead.
   */
  protected void onChange_bemonc() {
    onChange_bemonc_xjal( bemonc );
  }

  @AnyLogicInternalCodegenAPI
  protected void onChange_bemonc_xjal( boolean oldValue ) {
  }


  public
boolean  upgradeCS;

  /**
   * Returns default value for parameter <code>upgradeCS</code>.
   * <i>This method should not be called by user</i>
   */
  @AnyLogicInternalCodegenAPI
  public boolean _upgradeCS_DefaultValue_xjal() {
    final Hospital self = this;
    return false;
  }

  public void set_upgradeCS( boolean upgradeCS ) {
    if (upgradeCS == this.upgradeCS) {
      return;
    }
    boolean _oldValue_xjal = this.upgradeCS;
    this.upgradeCS = upgradeCS;
    onChange_upgradeCS_xjal( _oldValue_xjal );
    onChange();
  }

  /**
   * Calls "On change" action for parameter upgradeCS.<br>
   * Note that 'oldValue' in that action will be unavailable if this method is called
by user
   * (current parameter value will be passed as 'oldValue').<br>
   * Please call <code>set_upgradeCS()</code> method instead.
   */
  protected void onChange_upgradeCS() {
    onChange_upgradeCS_xjal( upgradeCS );
  }

  @AnyLogicInternalCodegenAPI
  protected void onChange_upgradeCS_xjal( boolean oldValue ) {
  }


  public
boolean  upgradeBemonc;

  /**
```

```
   * Returns default value for parameter <code>upgradeBemonc</code>.
   * <i>This method should not be called by user</i>
   */
  @AnyLogicInternalCodegenAPI
  public boolean _upgradeBemonc_DefaultValue_xjal() {
    final Hospital self = this;
    return false;
  }

  public void set_upgradeBemonc( boolean upgradeBemonc ) {
    if (upgradeBemonc == this.upgradeBemonc) {
      return;
    }
    boolean _oldValue_xjal = this.upgradeBemonc;
    this.upgradeBemonc = upgradeBemonc;
    onChange_upgradeBemonc_xjal( _oldValue_xjal );
    onChange();
  }

  /**
   * Calls "On change" action for parameter upgradeBemonc.<br>
   * Note that 'oldValue' in that action will be unavailable if this method is called
by user
   * (current parameter value will be passed as 'oldValue').<br>
   * Please call <code>set_upgradeBemonc()</code> method instead.
   */
  protected void onChange_upgradeBemonc() {
    onChange_upgradeBemonc_xjal( upgradeBemonc );
  }

  @AnyLogicInternalCodegenAPI
  protected void onChange_upgradeBemonc_xjal( boolean oldValue ) {
  }


  @Override
  public void setParametersToDefaultValues() {
    super.setParametersToDefaultValues();
    id = _id_DefaultValue_xjal();
    type = _type_DefaultValue_xjal();
    management = _management_DefaultValue_xjal();
    urban = _urban_DefaultValue_xjal();
    latitude = _latitude_DefaultValue_xjal();
    longitude = _longitude_DefaultValue_xjal();
    bobsCounterfactual = _bobsCounterfactual_DefaultValue_xjal();
    q = _q_DefaultValue_xjal();
    feesCounterfactual = _feesCounterfactual_DefaultValue_xjal();
    distance = _distance_DefaultValue_xjal();
    hospital = _hospital_DefaultValue_xjal();
    cs = _cs_DefaultValue_xjal();
    bemonc = _bemonc_DefaultValue_xjal();
    upgradeCS = _upgradeCS_DefaultValue_xjal();
    upgradeBemonc = _upgradeBemonc_DefaultValue_xjal();
  }

  @Override
  public boolean setParameter(String _name_xjal, Object _value_xjal, boolean
_callOnChange_xjal) {
    switch ( _name_xjal ) {
    case "id":
      if ( _callOnChange_xjal ) {
        set_id( (String) _value_xjal );
      } else {
```

```
    id = (String) _value_xjal;
  }
  return true;
case "type":
  if ( _callOnChange_xjal ) {
    set_type( (String) _value_xjal );
  } else {
    type = (String) _value_xjal;
  }
  return true;
case "management":
  if ( _callOnChange_xjal ) {
    set_management( (String) _value_xjal );
  } else {
    management = (String) _value_xjal;
  }
  return true;
case "urban":
  if ( _callOnChange_xjal ) {
    set_urban( (Boolean) _value_xjal );
  } else {
    urban = (Boolean) _value_xjal;
  }
  return true;
case "latitude":
  if ( _callOnChange_xjal ) {
    set_latitude( ((Number) _value_xjal).doubleValue() );
  } else {
    latitude = ((Number) _value_xjal).doubleValue();
  }
  return true;
case "longitude":
  if ( _callOnChange_xjal ) {
    set_longitude( ((Number) _value_xjal).doubleValue() );
  } else {
    longitude = ((Number) _value_xjal).doubleValue();
  }
  return true;
case "bobsCounterfactual":
  if ( _callOnChange_xjal ) {
    set_bobsCounterfactual( ((Number) _value_xjal).doubleValue() );
  } else {
    bobsCounterfactual = ((Number) _value_xjal).doubleValue();
  }
  return true;
case "q":
  if ( _callOnChange_xjal ) {
    set_q( ((Number) _value_xjal).doubleValue() );
  } else {
    q = ((Number) _value_xjal).doubleValue();
  }
  return true;
case "feesCounterfactual":
  if ( _callOnChange_xjal ) {
    set_feesCounterfactual( ((Number) _value_xjal).intValue() );
  } else {
    feesCounterfactual = ((Number) _value_xjal).intValue();
  }
  return true;
case "distance":
  if ( _callOnChange_xjal ) {
    set_distance( ((Number) _value_xjal).doubleValue() );
  } else {
```

```
      distance = ((Number) _value_xjal).doubleValue();
    }
    return true;
  case "hospital":
    if ( _callOnChange_xjal ) {
      set_hospital( (Boolean) _value_xjal );
    } else {
      hospital = (Boolean) _value_xjal;
    }
    return true;
  case "cs":
    if ( _callOnChange_xjal ) {
      set_cs( (Boolean) _value_xjal );
    } else {
      cs = (Boolean) _value_xjal;
    }
    return true;
  case "bemonc":
    if ( _callOnChange_xjal ) {
      set_bemonc( (Boolean) _value_xjal );
    } else {
      bemonc = (Boolean) _value_xjal;
    }
    return true;
  case "upgradeCS":
    if ( _callOnChange_xjal ) {
      set_upgradeCS( (Boolean) _value_xjal );
    } else {
      upgradeCS = (Boolean) _value_xjal;
    }
    return true;
  case "upgradeBemonc":
    if ( _callOnChange_xjal ) {
      set_upgradeBemonc( (Boolean) _value_xjal );
    } else {
      upgradeBemonc = (Boolean) _value_xjal;
    }
    return true;
  default:
    return super.setParameter( _name_xjal, _value_xjal, _callOnChange_xjal );
  }
}

@Override
public <T> T getParameter(String _name_xjal) {
  Object _result_xjal;
  switch ( _name_xjal ) {
  case "id": _result_xjal = id; break;
  case "type": _result_xjal = type; break;
  case "management": _result_xjal = management; break;
  case "urban": _result_xjal = urban; break;
  case "latitude": _result_xjal = latitude; break;
  case "longitude": _result_xjal = longitude; break;
  case "bobsCounterfactual": _result_xjal = bobsCounterfactual; break;
  case "q": _result_xjal = q; break;
  case "feesCounterfactual": _result_xjal = feesCounterfactual; break;
  case "distance": _result_xjal = distance; break;
  case "hospital": _result_xjal = hospital; break;
  case "cs": _result_xjal = cs; break;
  case "bemonc": _result_xjal = bemonc; break;
  case "upgradeCS": _result_xjal = upgradeCS; break;
  case "upgradeBemonc": _result_xjal = upgradeBemonc; break;
  default: _result_xjal = super.getParameter( _name_xjal ); break;
```

```
    }
    return (T) _result_xjal;
  }

  @AnyLogicInternalCodegenAPI
  private static String[] _parameterNames_xjal;

  @Override
  public String[] getParameterNames() {
    String[] result = _parameterNames_xjal;
    if (result == null) {
      List<String> list = new ArrayList<>( Arrays.asList( super.getParameterNames() )
);
      list.add( "id" );
      list.add( "type" );
      list.add( "management" );
      list.add( "urban" );
      list.add( "latitude" );
      list.add( "longitude" );
      list.add( "bobsCounterfactual" );
      list.add( "q" );
      list.add( "feesCounterfactual" );
      list.add( "distance" );
      list.add( "hospital" );
      list.add( "cs" );
      list.add( "bemonc" );
      list.add( "upgradeCS" );
      list.add( "upgradeBemonc" );
      result = list.toArray( new String[ list.size() ] );
      _parameterNames_xjal = result;
    }
    return result;
  }
  // Plain Variables

  public
double
 u;
  public
double
 p;
  /**
   * Coefficient on hospital type for class1
   */
  public
double
 bType1;
  /**
   * Coefficient on hospital type for class1
   */
  public
double
 bType2;
  public
int
 fees;
  public
double
 bobs;
  public
double
 mortality;
  public
```

```
boolean
 doIExist;
  public
boolean
 csVar;
  public
boolean
 bemoncVar;
  @AnyLogicInternalCodegenAPI
  private static Map<String, IElementDescriptor> elementDesciptors_xjal =
createElementDescriptors( Hospital.class );

  @AnyLogicInternalCodegenAPI
  @Override
  public Map<String, IElementDescriptor> getElementDesciptors() {
    return elementDesciptors_xjal;
  }
  @AnyLogicCustomProposalPriority(type =
AnyLogicCustomProposalPriority.Type.STATIC_ELEMENT)
  public static final Scale scale = new Scale( 10.0 );

  @Override
  public Scale getScale() {
    return scale;
  }






  // View areas
  public ViewArea _origin_VA = new ViewArea( this, "[Origin]", 0, 0, 1550.0, 890.0 );
  @Override
  @AnyLogicInternalCodegenAPI
  public int getViewAreas(Map<String, ViewArea> _output) {
    if ( _output != null ) {
      _output.put( "_origin_VA", this._origin_VA );
    }
    return 1 + super.getViewAreas( _output );
  }
  @AnyLogicInternalCodegenAPI
  protected static final int _rectangle = 1;

  /** Internal constant, shouldn't be accessed by user */
  @AnyLogicInternalCodegenAPI
  protected static final int _SHAPE_NEXT_ID_xjal = 2;


  /**
   * Top-level presentation group id
   */
  @AnyLogicInternalCodegenAPI
  protected static final int _presentation = 0;

  @AnyLogicInternalCodegenAPI
  public boolean isPublicPresentationDefined() {
    return true;
  }

  @AnyLogicInternalCodegenAPI
  public boolean isEmbeddedAgentPresentationVisible( Agent _a ) {
    return super.isEmbeddedAgentPresentationVisible( _a );
```

```
  }
  /**
   * Top-level icon group id
   */
  @AnyLogicInternalCodegenAPI
  protected static final int _icon = -1;


  @Override
  @AnyLogicInternalCodegenAPI
  public boolean onShapeClick( int _shape, int index, double clickx, double clicky ){
    switch( _shape ){
      case _rectangle:
        if (true) {
          ShapeRectangle self = this.rectangle;

main.vAgentID = this.getLatitude();
        }
        break;
      default: return super.onShapeClick( _shape, index, clickx, clicky );
       }
       return false;
  }

  protected ShapeRectangle rectangle;
  @AnyLogicInternalCodegenAPI
  private void _createPersistentElementsBP0_xjal() {
    rectangle = new ShapeRectangle(
       SHAPE_DRAW_2D3D, true,-5.0, -5.0, 0.0, 0.0,
            black, dodgerBlue,
                    5.0, 5.0, 0.0, 1.0, LINE_STYLE_SOLID ) {

      @Override
      @AnyLogicInternalCodegenAPI
      public boolean onClick( double clickx, double clicky ) {
        return onShapeClick( _rectangle, 0, clickx, clicky );
      }
    };

  }

  @AnyLogicInternalCodegenAPI
  private void _createPersistentElementsAP0_xjal() {
  }


  // Static initialization of persistent elements
  {
    _createPersistentElementsBP0_xjal();
  }
  protected ShapeTopLevelPresentationGroup presentation;
  protected ShapeModelElementsGroup icon;

  @Override
  @AnyLogicInternalCodegenAPI
  public ShapeTopLevelPresentationGroup getPresentationShape() {
    return presentation;
  }

  @Override
  @AnyLogicInternalCodegenAPI
  public ShapeModelElementsGroup getModelElementsShape() {
    return icon;
```

```
  }

  @Override
  @AnyLogicInternalCodegenAPI
  public Object getPersistentShape( int _shape ) {
    switch (_shape) {
      case _presentation: return presentation;
      case _icon: return icon;
      case _rectangle: return rectangle;
      default: return super.getPersistentShape( _shape );
    }
  }

  @Override
  @AnyLogicInternalCodegenAPI
  public String getNameOfShape_xjal( Object _shape ) {
    try {
      if ( _shape == null ) return null;
      String _name_xjal;
      _name_xjal = checkNameOfShape_xjal( _shape, presentation, "presentation" ); if
(_name_xjal != null) return _name_xjal;
      _name_xjal = checkNameOfShape_xjal( _shape, icon, "icon" ); if (_name_xjal !=
null) return _name_xjal;
      _name_xjal = checkNameOfShape_xjal( _shape, rectangle, "rectangle" ); if
(_name_xjal != null) return _name_xjal;
    } catch (Exception e) {
      return null;
    }
    return super.getNameOfShape_xjal( _shape );
  }

  @AnyLogicInternalCodegenAPI
  private void drawModelElements_Parameters_xjal(Panel _panel, Graphics2D _g, boolean
_publicOnly, boolean _isSuperClass ) {
    if (!_publicOnly) {
      drawParameter( _panel, _g, 50, 50, 10, 0, "id", id, 0 );
    }
    if (!_publicOnly) {
      drawParameter( _panel, _g, 50, 100, 10, 0, "type", type, 0 );
    }
    if (!_publicOnly) {
      drawParameter( _panel, _g, 50, 150, 10, 0, "management", management, 0 );
    }
    if (!_publicOnly) {
      drawParameter( _panel, _g, 50, 200, 10, 0, "urban", urban, 0 );
    }
    if (!_publicOnly) {
      drawParameter( _panel, _g, 50, 250, 10, 0, "latitude", latitude, 0 );
    }
    if (!_publicOnly) {
      drawParameter( _panel, _g, 50, 300, 10, 0, "longitude", longitude, 0 );
    }
    if (!_publicOnly) {
      drawParameter( _panel, _g, 50, 350, 10, 0, "bobsCounterfactual",
bobsCounterfactual, 0 );
    }
    if (!_publicOnly) {
      drawParameter( _panel, _g, 50, 400, 10, 0, "q", q, 0 );
    }
    if (!_publicOnly) {
      drawParameter( _panel, _g, 50, 450, 10, 0, "feesCounterfactual",
feesCounterfactual, 0 );
    }
```

```
    if (!_publicOnly) {
      drawParameter( _panel, _g, 50, 500, 10, 0, "distance", distance, 0 );
    }
    if (!_publicOnly) {
      drawParameter( _panel, _g, 50, 550, 10, 0, "hospital", hospital, 0 );
    }
    if (!_publicOnly) {
      drawParameter( _panel, _g, 50, 600, 10, 0, "cs", cs, 0 );
    }
    if (!_publicOnly) {
      drawParameter( _panel, _g, 50, 650, 10, 0, "bemonc", bemonc, 0 );
    }
    if (!_publicOnly) {
      drawParameter( _panel, _g, 50, 700, 10, 0, "upgradeCS", upgradeCS, 0 );
    }
    if (!_publicOnly) {
      drawParameter( _panel, _g, 50, 750, 10, 0, "upgradeBemonc", upgradeBemonc, 0 );
    }
  }

  @AnyLogicInternalCodegenAPI
  private void drawModelElements_PlainVariables_xjal(Panel _panel, Graphics2D _g,
boolean _publicOnly, boolean _isSuperClass ) {
    if (!_publicOnly) {
      drawPlainVariable( _panel, _g, 150, 100, 10, 0, "u", u, false );
    }
    if (!_publicOnly) {
      drawPlainVariable( _panel, _g, 150, 150, 10, 0, "p", p, false );
    }
    if (!_publicOnly) {
      drawPlainVariable( _panel, _g, 150, 200, 10, 0, "bType1", bType1, false );
    }
    if (!_publicOnly) {
      drawPlainVariable( _panel, _g, 150, 250, 10, 0, "bType2", bType2, false );
    }
    if (!_publicOnly) {
      drawPlainVariable( _panel, _g, 150, 300, 10, 0, "fees", fees, false );
    }
    if (!_publicOnly) {
      drawPlainVariable( _panel, _g, 150, 400, 10, 0, "bobs", bobs, false );
    }
    if (!_publicOnly) {
      drawPlainVariable( _panel, _g, 150, 500, 10, 0, "mortality", mortality, false );
    }
    if (!_publicOnly) {
      drawPlainVariable( _panel, _g, 150, 50, 10, 0, "doIExist", doIExist, false );
    }
    if (!_publicOnly) {
      drawPlainVariable( _panel, _g, 150, 600, 10, 0, "csVar", csVar, false );
    }
    if (!_publicOnly) {
      drawPlainVariable( _panel, _g, 150, 650, 10, 0, "bemoncVar", bemoncVar, false );
    }
  }

  @AnyLogicInternalCodegenAPI
  private void drawModelElements_AgentLinks_xjal(Panel _panel, Graphics2D _g, boolean
_publicOnly, boolean _isSuperClass ) {
    if (_publicOnly) { return; }
      drawLinkToContainer( _panel, _g, 50, -100, 10, 0, "main", main );
      drawLinkToAgent( _panel, _g, 50, -50, 15, 0, "connections", true, connections );
  }
```

```
  @Override
  @AnyLogicInternalCodegenAPI
  public void drawModelElements( Panel _panel, Graphics2D _g, boolean _publicOnly,
boolean _isSuperClass ) {
      super.drawModelElements( _panel, _g, _publicOnly, true );
    drawModelElements_Parameters_xjal( _panel, _g, _publicOnly, _isSuperClass );
    drawModelElements_PlainVariables_xjal( _panel, _g, _publicOnly, _isSuperClass );
    drawModelElements_AgentLinks_xjal( _panel, _g, _publicOnly, _isSuperClass );
  }

  @AnyLogicInternalCodegenAPI
  private boolean onClickModelAt_AgentLinks_xjal( Panel _panel, double _x, double _y,
int _clickCount, boolean _publicOnly, boolean _isSuperClass ) {
    if ( modelElementContains(_x, _y, 50, -100) ) {
      if ( _clickCount == 2 ) {
        _panel.browseAgent_xjal( 50, -100, this, "main" );
      } else {
        _panel.addInspect( 50, -100, this, "main" );
      }
      return true;
    }
    if ( modelElementContains(_x, _y, 50, -50) ) {
        _panel.addInspect_xjal( 50, -50, this, "connections",
Panel.INSPECT_CONNECTIONS_xjal );
      return true;
    }
    return false;
  }


  @AnyLogicInternalCodegenAPI
  private boolean onClickModelAt_Parameters_xjal( Panel _panel, double _x, double _y,
int _clickCount, boolean _publicOnly, boolean _isSuperClass ) {
    if( !_publicOnly && modelElementContains(_x, _y, 50, 50) ) {
      _panel.addInspect( 50, 50, this, "id" );
      return true;
    }
    if( !_publicOnly && modelElementContains(_x, _y, 50, 100) ) {
      _panel.addInspect( 50, 100, this, "type" );
      return true;
    }
    if( !_publicOnly && modelElementContains(_x, _y, 50, 150) ) {
      _panel.addInspect( 50, 150, this, "management" );
      return true;
    }
    if( !_publicOnly && modelElementContains(_x, _y, 50, 200) ) {
      _panel.addInspect( 50, 200, this, "urban" );
      return true;
    }
    if( !_publicOnly && modelElementContains(_x, _y, 50, 250) ) {
      _panel.addInspect( 50, 250, this, "latitude" );
      return true;
    }
    if( !_publicOnly && modelElementContains(_x, _y, 50, 300) ) {
      _panel.addInspect( 50, 300, this, "longitude" );
      return true;
    }
    if( !_publicOnly && modelElementContains(_x, _y, 50, 350) ) {
      _panel.addInspect( 50, 350, this, "bobsCounterfactual" );
      return true;
    }
    if( !_publicOnly && modelElementContains(_x, _y, 50, 400) ) {
      _panel.addInspect( 50, 400, this, "q" );
```

```
        return true;
      }
      if( !_publicOnly && modelElementContains(_x, _y, 50, 450) ) {
        _panel.addInspect( 50, 450, this, "feesCounterfactual" );
        return true;
      }
      if( !_publicOnly && modelElementContains(_x, _y, 50, 500) ) {
        _panel.addInspect( 50, 500, this, "distance" );
        return true;
      }
      if( !_publicOnly && modelElementContains(_x, _y, 50, 550) ) {
        _panel.addInspect( 50, 550, this, "hospital" );
        return true;
      }
      if( !_publicOnly && modelElementContains(_x, _y, 50, 600) ) {
        _panel.addInspect( 50, 600, this, "cs" );
        return true;
      }
      if( !_publicOnly && modelElementContains(_x, _y, 50, 650) ) {
        _panel.addInspect( 50, 650, this, "bemonc" );
        return true;
      }
      if( !_publicOnly && modelElementContains(_x, _y, 50, 700) ) {
        _panel.addInspect( 50, 700, this, "upgradeCS" );
        return true;
      }
      if( !_publicOnly && modelElementContains(_x, _y, 50, 750) ) {
        _panel.addInspect( 50, 750, this, "upgradeBemonc" );
        return true;
      }
      return false;
    }

    @AnyLogicInternalCodegenAPI
    private boolean onClickModelAt_PlainVariables_xjal( Panel _panel, double _x, double
  _y, int _clickCount, boolean _publicOnly, boolean _isSuperClass ) {
      if( !_publicOnly && modelElementContains(_x, _y, 150, 100) ) {
        _panel.addInspect( 150, 100, this, "u" );
        return true;
      }
      if( !_publicOnly && modelElementContains(_x, _y, 150, 150) ) {
        _panel.addInspect( 150, 150, this, "p" );
        return true;
      }
      if( !_publicOnly && modelElementContains(_x, _y, 150, 200) ) {
        _panel.addInspect( 150, 200, this, "bType1" );
        return true;
      }
      if( !_publicOnly && modelElementContains(_x, _y, 150, 250) ) {
        _panel.addInspect( 150, 250, this, "bType2" );
        return true;
      }
      if( !_publicOnly && modelElementContains(_x, _y, 150, 300) ) {
        _panel.addInspect( 150, 300, this, "fees" );
        return true;
      }
      if( !_publicOnly && modelElementContains(_x, _y, 150, 400) ) {
        _panel.addInspect( 150, 400, this, "bobs" );
        return true;
      }
      if( !_publicOnly && modelElementContains(_x, _y, 150, 500) ) {
        _panel.addInspect( 150, 500, this, "mortality" );
        return true;
```

```
    }
    if( !_publicOnly && modelElementContains(_x, _y, 150, 50) ) {
      _panel.addInspect( 150, 50, this, "doIExist" );
      return true;
    }
    if( !_publicOnly && modelElementContains(_x, _y, 150, 600) ) {
      _panel.addInspect( 150, 600, this, "csVar" );
      return true;
    }
    if( !_publicOnly && modelElementContains(_x, _y, 150, 650) ) {
      _panel.addInspect( 150, 650, this, "bemoncVar" );
      return true;
    }
    return false;
  }

  @Override
  @AnyLogicInternalCodegenAPI
  public boolean onClickModelAt( Panel _panel, double _x, double _y, int _clickCount,
boolean _publicOnly, boolean _isSuperClass ) {
    if ( onClickModelAt_AgentLinks_xjal( _panel, _x, _y, _clickCount, _publicOnly,
_isSuperClass ) ) { return true; }
    if ( onClickModelAt_Parameters_xjal( _panel, _x, _y, _clickCount, _publicOnly,
_isSuperClass ) ) { return true; }
    if ( onClickModelAt_PlainVariables_xjal( _panel, _x, _y, _clickCount, _publicOnly,
_isSuperClass ) ) { return true; }
    return super.onClickModelAt( _panel, _x, _y, _clickCount, _publicOnly, true );
  }



  /**
   * Constructor
   */
  public Hospital( Engine engine, Agent owner, AgentList<? extends Hospital>
ownerPopulation ) {
    super( engine, owner, ownerPopulation );
    instantiateBaseStructureThis_xjal();
  }

  @AnyLogicInternalCodegenAPI
  public void onOwnerChanged_xjal() {
    super.onOwnerChanged_xjal();
    setupReferences_xjal();
  }

  @AnyLogicInternalCodegenAPI
  public void instantiateBaseStructure_xjal() {
    super.instantiateBaseStructure_xjal();
    instantiateBaseStructureThis_xjal();
  }

  @AnyLogicInternalCodegenAPI
  private void instantiateBaseStructureThis_xjal() {
    setupReferences_xjal();
  }

  @AnyLogicInternalCodegenAPI
  private void setupReferences_xjal() {
    main = get_Main();
  }

  /**
```

```
   * Simple constructor. Please add created agent to some population by calling
goToPopulation() function
   */
  public Hospital() {
  }

  /**
   * Simple constructor. Please add created agent to some population by calling
goToPopulation() function
   */
  public Hospital( String id, String type, String management, boolean urban, double
latitude, double longitude, double bobsCounterfactual, double q, int
feesCounterfactual, double distance, boolean hospital, boolean cs, boolean bemonc,
boolean upgradeCS, boolean upgradeBemonc ) {
    markParametersAreSet();
    this.id = id;
    this.type = type;
    this.management = management;
    this.urban = urban;
    this.latitude = latitude;
    this.longitude = longitude;
    this.bobsCounterfactual = bobsCounterfactual;
    this.q = q;
    this.feesCounterfactual = feesCounterfactual;
    this.distance = distance;
    this.hospital = hospital;
    this.cs = cs;
    this.bemonc = bemonc;
    this.upgradeCS = upgradeCS;
    this.upgradeBemonc = upgradeBemonc;
  }

  @Override
  @AnyLogicInternalCodegenAPI
  public void doCreate() {
    super.doCreate();
    // Assigning initial values for plain variables
    setupPlainVariables_Hospital_xjal();
    // Dynamic initialization of persistent elements
    _createPersistentElementsAP0_xjal();
    presentation = new ShapeTopLevelPresentationGroup( Hospital.this, true, 0, 0, 0, 0
, rectangle );
    icon = new ShapeModelElementsGroup( Hospital.this, getElementProperty(
"malawi_recentralization_model_2017_06_04.Hospital.icon",
IElementDescriptor.MODEL_ELEMENT_DESCRIPTORS )  );
    icon.setIconOffsets( 0.0, 0.0 );
        // Port connectors with non-replicated objects
    // Creating replicated embedded objects
    setupInitialConditions_xjal( Hospital.class );
  }

  @AnyLogicInternalCodegenAPI
  public void setupExt_xjal(AgentExtension _ext) {
    // Agent properties setup
    if ( _ext instanceof ExtAgentWithSpatialMetrics && _ext instanceof
ExtWithSpaceType ) {
      double _value;
      _value =
10
;
      ((ExtAgentWithSpatialMetrics) _ext).setSpeed( _value, MPS );
    }
  }
```

```
  @Override
  @AnyLogicInternalCodegenAPI
  public void doStart() {
    super.doStart();
  }

  @AnyLogicInternalCodegenAPI
  public void onStartup() {
    super.onStartup();

Random r = new MersenneTwisterRNG();  //from uncommon maths package (found in the
/Mark/Models folder)

if(main.strategy == 2 & this.upgradeCS){
	this.csVar = true;
}

if(main.strategy == 4 & this.upgradeBemonc){
	this.bemoncVar = true;
}

switch (this.type) {
	case "Central hospital":
		this.rectangle.setFillColor(Color.red);
		doIExist = true;
		this.bType1 = main.centralHosp1;
		this.bType2 = main.centralHosp2;

		if(this.bemoncVar){
			if(this.csVar){
				this.mortality = new GaussianGenerator(min(main.centralCS,
main.central5), max(main.centralCSSE, main.central5SE), r).nextValue(); //choosing the
best mortality AND highest variance (because this an assumption)
			} else {
				this.mortality = new GaussianGenerator(main.central5,
main.central5SE, r).nextValue();
			}
		} else {
			if(this.csVar){
				this.mortality = new GaussianGenerator(main.centralCS,
main.centralCSSE, r).nextValue();
			} else {
				this.mortality = new GaussianGenerator(max(main.centralCS,
main.central5), max(main.centralCSSE, main.central5SE), r).nextValue(); //choosing the
worst mortality AND highest variance (because this an assumption)
			}
		}

		//this.bType2 = main.bCentral2;
		break;
	case "District hospital":
		this.rectangle.setFillColor(Color.blue);
		doIExist = true;
		this.bType1 = main.districtHosp1;
		this.bType2 = main.districtHosp2;

		if(this.bemoncVar){
			if(this.csVar){
				this.mortality = new GaussianGenerator(min(main.districtCS,
main.district5), max(main.districtCSSE, main.district5SE), r).nextValue(); //choosing
the best mortality AND highest variance (because this an assumption)
			} else {
```

```
                    this.mortality = new GaussianGenerator(main.district5,
main.district5SE, r).nextValue();
                }
            } else {
                if(this.csVar){
                    this.mortality = new GaussianGenerator(main.districtCS,
main.districtCSSE, r).nextValue();
                } else {
                    this.mortality = new GaussianGenerator(max(main.districtCS,
main.district5), max(main.districtCSSE, main.district5SE), r).nextValue(); //choosing
the worst mortality AND highest variance (because this an assumption)
                }
            }

            //this.bType2 = main.bDistrict2;
            break;
        case "Community hospital":
            this.rectangle.setFillColor(Color.orange);
            doIExist = true;
            this.bType1 = main.communityHosp1;
            this.bType2 = main.communityHosp2;

            if(this.bemoncVar){
                if(this.csVar){
                    this.mortality = new
GaussianGenerator(min(main.communityCS, main.community5), max(main.communityCSSE,
main.community5SE), r).nextValue(); //choosing the best mortality AND highest variance
(because this an assumption)
                } else {
                    this.mortality = new GaussianGenerator(main.community5,
main.community5SE, r).nextValue();
                }
            } else {
                if(this.csVar){
                    this.mortality = new GaussianGenerator(main.communityCS,
main.communityCSSE, r).nextValue();
                } else {
                    this.mortality = new
GaussianGenerator(max(main.communityCS, main.community5), max(main.communityCSSE,
main.community5SE), r).nextValue(); //choosing the worst mortality AND highest
variance (because this an assumption)
                }
            }

            //this.bType2 = main.bCommunity2;
            break;
        case "Other hospital":
            this.rectangle.setFillColor(Color.yellow);
            doIExist = true;
            this.bType1 = main.otherHosp1;
            this.bType2 = main.otherHosp2;

            if(this.bemoncVar){
                if(this.csVar){
                    this.mortality = new GaussianGenerator(min(main.otherCS,
main.other5), max(main.otherCSSE, main.other5SE), r).nextValue(); //choosing the best
mortality AND highest variance (because this an assumption)
                } else {
                    this.mortality = new GaussianGenerator(main.other5,
main.other5SE, r).nextValue();
                }
            } else {
                if(this.csVar){
```

```
				this.mortality = new GaussianGenerator(main.otherCS, main.otherCSSE, r).nextValue();
			} else {
				this.mortality = new GaussianGenerator(max(main.otherCS, main.other5), max(main.otherCSSE, main.other5SE), r).nextValue(); //choosing the worst mortality AND highest variance (because this an assumption)
			}
		}

		//this.bType2 = main.bOther2;
		break;
	case "Health center":
		this.rectangle.setFillColor(Color.lightGray);
		if(main.allowAll) {doIExist = true;}
		this.bType1 = main.healthCenter1;
		this.bType2 = main.healthCenter2;

		if(this.bemoncVar){
			if(this.csVar){
				this.mortality = new GaussianGenerator(min(main.healthCenterCS, main.healthCenter5), max(main.healthCenterCSSE, main.healthCenter5SE), r).nextValue(); //choosing the best mortality AND highest variance (because this an assumption)
			} else {
				this.mortality = new GaussianGenerator(main.healthCenter5, main.healthCenter5SE, r).nextValue();
			}
		} else {
			if(this.csVar){
				this.mortality = new GaussianGenerator(main.healthCenterCS, main.healthCenterCSSE, r).nextValue();
			} else {
				this.mortality = new GaussianGenerator(max(main.healthCenterCS, main.healthCenter5), max(main.healthCenterCSSE, main.healthCenter5SE), r).nextValue(); //choosing the worst mortality AND highest variance (because this an assumption)
			}
		}

		//this.bType2 = main.bHealthCenter2;
		break;
	case "Maternity":
		this.rectangle.setFillColor(Color.pink);
		if(main.allowAll) {doIExist = true;}
		this.bType1 = main.maternity1;
		this.bType2 = main.maternity2;

		if(this.bemoncVar){
			if(this.csVar){
				this.mortality = new GaussianGenerator(min(main.maternityCS, main.maternity5), max(main.maternityCSSE, main.maternity5SE), r).nextValue(); //choosing the best mortality AND highest variance (because this an assumption)
			} else {
				this.mortality = new GaussianGenerator(main.maternity5, main.maternity5SE, r).nextValue();
			}
		} else {
			if(this.csVar){
				this.mortality = new GaussianGenerator(main.maternityCS, main.maternityCSSE, r).nextValue();
			} else {
```

```
                        this.mortality = new
GaussianGenerator(max(main.maternityCS, main.maternity5), max(main.maternityCSSE,
main.maternity5SE), r).nextValue(); //choosing the worst mortality AND highest
variance (because this an assumption)
                    }
                }

                //this.bType2 = main.bMaternity2;
                break;
            case "Clinic":
                this.rectangle.setFillColor(Color.green);
                if(main.allowAll) {doIExist = true;}
                this.bType1 = main.clinic1;
                this.bType2 = main.clinic2;

                if(this.bemoncVar){
                    if(this.csVar){
                        this.mortality = new GaussianGenerator(min(main.clinicCS,
main.clinic5), max(main.clinicCSSE, main.clinic5SE), r).nextValue(); //choosing the
best mortality AND highest variance (because this an assumption)
                    } else {
                        this.mortality = new GaussianGenerator(main.clinic5,
main.clinic5SE, r).nextValue();
                    }
                } else {
                    if(this.csVar){
                        this.mortality = new GaussianGenerator(main.clinicCS,
main.clinicCSSE, r).nextValue();
                    } else {
                        this.mortality = new GaussianGenerator(max(main.clinicCS,
main.clinic5), max(main.clinicCSSE, main.clinic5SE), r).nextValue(); //choosing the
worst mortality AND highest variance (because this an assumption)
                    }
                }

                //this.bType2 = main.bClinic2;
                break;
            default: throw new IllegalArgumentException("Hospital type not found: " +
this.type);
}

this.mortality = max(0, this.mortality); //prevent negative mortality rates

//double b = (this.bobsCounterfactual - 0.151515156) / (1.0 - 0.151515156);
//Color c = new Color(round(255f * (float)b), round(255f * (float)b), round(255f *
(float)b));
//this.rectangle.setFillColor(c);
//this.rectangle.setHeight(7);
//this.rectangle.setWidth(7);


if(!main.allowAll)
{
    this.bobs = min(1.0, this.bobsCounterfactual + main.QI);
} else {
    this.bobs = this.bobsCounterfactual;
}

if(main.allowFees)
{
    this.fees = feesCounterfactual;
} else {
    this.fees = 0;
```

```
}

main.hospitalList.add(this);

this.doIExist = false;
switch (main.strategy) {
	case 0: //status quo
		this.doIExist = true;
		break;
	case 1: //only CS
		if(this.cs) {this.doIExist = true;}
		break;
	case 2: //upgrade CS
		if(this.upgradeCS) {this.doIExist = true;}
		break;
	case 3: //only BEMC
		if(this.bemonc) {this.doIExist = true;}
		break;
	case 4: //upgrade BEMC
		if(this.upgradeBemonc) {this.doIExist = true;}
		break;
	default: throw new IllegalStateException("Strategy selected: " +
main.strategy);
}

if(this.doIExist) {main.allowedList.add(this);}
else {this.rectangle.setVisible(false);}

main.update_avgBobs();
main.update_avgFees();


//System.out.println(main.strategy + "\t" + this.getId() + "\t" + this.doIExist + "\t"
+ this.bemonc + "\t" + this.mortality + "\t" + main.allowedList.size());
  }

  /**
   * Assigning initial values for plain variables<br>
   * <em>This method isn't designed to be called by user and may be removed in future
releases.</em>
   */
  @AnyLogicInternalCodegenAPI
  public void setupPlainVariables_xjal() {
    setupPlainVariables_Hospital_xjal();
  }

  /**
   * Assigning initial values for plain variables<br>
   * <em>This method isn't designed to be called by user and may be removed in future
releases.</em>
   */
  @AnyLogicInternalCodegenAPI
  private void setupPlainVariables_Hospital_xjal() {
    doIExist =
false
;
    csVar =
cs
;
    bemoncVar =
bemonc
;
  }
```

```
  // User API -----------------------------------------------------
  public Main get_Main() {
    {
      Agent owner = getOwner();
      if ( owner instanceof Main ) return (Main) owner;
    }
    return null;
  }

  /**
   * Read-only variable. <em>Shouldn't be modified by user.</em>
   */
  @AnyLogicCustomSerialization(AnyLogicCustomSerializationMode.REFERENCE)
  public transient malawi_recentralization_model_2017_06_04.Main main;

  @AnyLogicInternalCodegenAPI
  static LinkToAgentAnimationSettings _connections_commonAnimationSettings_xjal = new
LinkToAgentAnimationSettingsImpl( false, black, 1.0, LINE_STYLE_SOLID, ARROW_NONE, 0.0
);

  public LinkToAgentCollection<Agent, Agent> connections = new
LinkToAgentStandardImpl<Agent, Agent>(this,
_connections_commonAnimationSettings_xjal);
  @Override
  public LinkToAgentCollection<? extends Agent, ? extends Agent>
getLinkToAgentStandard_xjal() {
    return connections;
  }


  @AnyLogicInternalCodegenAPI
  public void drawLinksToAgents(boolean _underAgents_xjal, LinkToAgentAnimator
_animator_xjal) {
    super.drawLinksToAgents(_underAgents_xjal, _animator_xjal);
    if ( _underAgents_xjal ) {
      _animator_xjal.drawLink( this, connections, true, true );
    }
  }

  public AgentList<? extends Hospital> getPopulation() {
    return (AgentList<? extends Hospital>) super.getPopulation();
  }

  public List<? extends Hospital> agentsInRange( double distance ) {
    return (List<? extends Hospital>) super.agentsInRange( distance );
  }



}
```

```
package malawi_recentralization_model_2017_06_04;

import java.io.Serializable;
import java.sql.Connection;
import java.sql.SQLException;
import java.util.ArrayDeque;
import java.util.ArrayList;
import java.util.Arrays;
```

```java
import java.util.Calendar;
import java.util.Collection;
import java.util.Collections;
import java.util.Comparator;
import java.util.Currency;
import java.util.Date;
import java.util.Enumeration;
import java.util.HashMap;
import java.util.HashSet;
import java.util.Hashtable;
import java.util.Iterator;
import java.util.LinkedHashMap;
import java.util.LinkedHashSet;
import java.util.LinkedList;
import java.util.List;
import java.util.ListIterator;
import java.util.Locale;
import java.util.Map;
import java.util.PriorityQueue;
import java.util.Random;
import java.util.Set;
import java.util.SortedMap;
import java.util.SortedSet;
import java.util.Stack;
import java.util.Timer;
import java.util.TreeMap;
import java.util.TreeSet;
import java.util.Vector;
import java.awt.Color;
import java.awt.Font;
import java.awt.Graphics2D;
import java.awt.geom.AffineTransform;
import com.anylogic.engine.connectivity.ResultSet;
import com.anylogic.engine.connectivity.Statement;
import com.anylogic.engine.elements.*;
import com.anylogic.engine.markup.Network;
import com.anylogic.engine.Position;
import com.anylogic.engine.markup.PedFlowStatistics;
import com.anylogic.engine.markup.DensityMap;


import static java.lang.Math.*;
import static com.anylogic.engine.UtilitiesArray.*;
import static com.anylogic.engine.UtilitiesCollection.*;
import static com.anylogic.engine.presentation.UtilitiesColor.*;
import static com.anylogic.engine.HyperArray.*;

import com.anylogic.engine.*;
import com.anylogic.engine.analysis.*;
import com.anylogic.engine.connectivity.*;
import com.anylogic.engine.database.*;
import com.anylogic.engine.gis.*;
import com.anylogic.engine.markup.*;
import com.anylogic.engine.presentation.*;

import com.mysema.query.Tuple;
import com.mysema.query.sql.SQLBindings;
import static malawi_recentralization_model_2017_06_04.DBDescriptor.*;

import java.awt.geom.Arc2D;

import com.mysema.query.BooleanBuilder;
import com.mysema.query.types.expr.MathExpressions;
```

```
import org.dom4j.IllegalAddException;
import org.apache.tools.ant.filters.TokenFilter.Trim;
import org.uncommons.maths.random.MersenneTwisterRNG;
import org.uncommons.maths.random.GaussianGenerator;

public class Person extends Agent
{
  // Parameters

  public
double  longitude;

  /**
   * Returns default value for parameter <code>longitude</code>.
   * <i>This method should not be called by user</i>
   */
  @AnyLogicInternalCodegenAPI
  public double _longitude_DefaultValue_xjal() {
    final Person self = this;
    return 0.0;
  }

  public void set_longitude( double longitude ) {
    if (longitude == this.longitude) {
      return;
    }
    double _oldValue_xjal = this.longitude;
    this.longitude = longitude;
    onChange_longitude_xjal( _oldValue_xjal );
    onChange();
  }

  /**
   * Calls "On change" action for parameter longitude.<br>
   * Note that 'oldValue' in that action will be unavailable if this method is called
by user
   * (current parameter value will be passed as 'oldValue').<br>
   * Please call <code>set_longitude()</code> method instead.
   */
  protected void onChange_longitude() {
    onChange_longitude_xjal( longitude );
  }

  @AnyLogicInternalCodegenAPI
  protected void onChange_longitude_xjal( double oldValue ) {
  }


  public
double  latitude;

  /**
   * Returns default value for parameter <code>latitude</code>.
   * <i>This method should not be called by user</i>
   */
  @AnyLogicInternalCodegenAPI
  public double _latitude_DefaultValue_xjal() {
    final Person self = this;
    return 0.0;
  }

  public void set_latitude( double latitude ) {
    if (latitude == this.latitude) {
```

```
      return;
    }
    double _oldValue_xjal = this.latitude;
    this.latitude = latitude;
    onChange_latitude_xjal( _oldValue_xjal );
    onChange();
  }

  /**
   * Calls "On change" action for parameter latitude.<br>
   * Note that 'oldValue' in that action will be unavailable if this method is called
by user
   * (current parameter value will be passed as 'oldValue').<br>
   * Please call <code>set_latitude()</code> method instead.
   */
  protected void onChange_latitude() {
    onChange_latitude_xjal( latitude );
  }

  @AnyLogicInternalCodegenAPI
  protected void onChange_latitude_xjal( double oldValue ) {
  }


  public
int  wealth;

  /**
   * Returns default value for parameter <code>wealth</code>.
   * <i>This method should not be called by user</i>
   */
  @AnyLogicInternalCodegenAPI
  public int _wealth_DefaultValue_xjal() {
    final Person self = this;
    return 0;
  }

  public void set_wealth( int wealth ) {
    if (wealth == this.wealth) {
      return;
    }
    int _oldValue_xjal = this.wealth;
    this.wealth = wealth;
    onChange_wealth_xjal( _oldValue_xjal );
    onChange();
  }

  /**
   * Calls "On change" action for parameter wealth.<br>
   * Note that 'oldValue' in that action will be unavailable if this method is called
by user
   * (current parameter value will be passed as 'oldValue').<br>
   * Please call <code>set_wealth()</code> method instead.
   */
  protected void onChange_wealth() {
    onChange_wealth_xjal( wealth );
  }

  @AnyLogicInternalCodegenAPI
  protected void onChange_wealth_xjal( int oldValue ) {
  }
```

```
  public
int  educSec;

  /**
   * Returns default value for parameter <code>educSec</code>.
   * <i>This method should not be called by user</i>
   */
  @AnyLogicInternalCodegenAPI
  public int _educSec_DefaultValue_xjal() {
    final Person self = this;
    return 0;
  }

  public void set_educSec( int educSec ) {
    if (educSec == this.educSec) {
      return;
    }
    int _oldValue_xjal = this.educSec;
    this.educSec = educSec;
    onChange_educSec_xjal( _oldValue_xjal );
    onChange();
  }

  /**
   * Calls "On change" action for parameter educSec.<br>
   * Note that 'oldValue' in that action will be unavailable if this method is called
by user
   * (current parameter value will be passed as 'oldValue').<br>
   * Please call <code>set_educSec()</code> method instead.
   */
  protected void onChange_educSec() {
    onChange_educSec_xjal( educSec );
  }

  @AnyLogicInternalCodegenAPI
  protected void onChange_educSec_xjal( int oldValue ) {
  }


  public
int  age;

  /**
   * Returns default value for parameter <code>age</code>.
   * <i>This method should not be called by user</i>
   */
  @AnyLogicInternalCodegenAPI
  public int _age_DefaultValue_xjal() {
    final Person self = this;
    return 0;
  }

  public void set_age( int age ) {
    if (age == this.age) {
      return;
    }
    int _oldValue_xjal = this.age;
    this.age = age;
    onChange_age_xjal( _oldValue_xjal );
    onChange();
  }

  /**
```

```
   * Calls "On change" action for parameter age.<br>
   * Note that 'oldValue' in that action will be unavailable if this method is called
by user
   * (current parameter value will be passed as 'oldValue').<br>
   * Please call <code>set_age()</code> method instead.
   */
  protected void onChange_age() {
    onChange_age_xjal( age );
  }

  @AnyLogicInternalCodegenAPI
  protected void onChange_age_xjal( int oldValue ) {
  }


  public
int  ANC4;

  /**
   * Returns default value for parameter <code>ANC4</code>.
   * <i>This method should not be called by user</i>
   */
  @AnyLogicInternalCodegenAPI
  public int _ANC4_DefaultValue_xjal() {
    final Person self = this;
    return 0;
  }

  public void set_ANC4( int ANC4 ) {
    if (ANC4 == this.ANC4) {
      return;
    }
    int _oldValue_xjal = this.ANC4;
    this.ANC4 = ANC4;
    onChange_ANC4_xjal( _oldValue_xjal );
    onChange();
  }

  /**
   * Calls "On change" action for parameter ANC4.<br>
   * Note that 'oldValue' in that action will be unavailable if this method is called
by user
   * (current parameter value will be passed as 'oldValue').<br>
   * Please call <code>set_ANC4()</code> method instead.
   */
  protected void onChange_ANC4() {
    onChange_ANC4_xjal( ANC4 );
  }

  @AnyLogicInternalCodegenAPI
  protected void onChange_ANC4_xjal( int oldValue ) {
  }


  public
int  urban;

  /**
   * Returns default value for parameter <code>urban</code>.
   * <i>This method should not be called by user</i>
   */
  @AnyLogicInternalCodegenAPI
  public int _urban_DefaultValue_xjal() {
```

```
    final Person self = this;
    return 0;
  }

  public void set_urban( int urban ) {
    if (urban == this.urban) {
      return;
    }
    int _oldValue_xjal = this.urban;
    this.urban = urban;
    onChange_urban_xjal( _oldValue_xjal );
    onChange();
  }

  /**
   * Calls "On change" action for parameter urban.<br>
   * Note that 'oldValue' in that action will be unavailable if this method is called
by user
   * (current parameter value will be passed as 'oldValue').<br>
   * Please call <code>set_urban()</code> method instead.
   */
  protected void onChange_urban() {
    onChange_urban_xjal( urban );
  }

  @AnyLogicInternalCodegenAPI
  protected void onChange_urban_xjal( int oldValue ) {
  }


  public
int  twins;

  /**
   * Returns default value for parameter <code>twins</code>.
   * <i>This method should not be called by user</i>
   */
  @AnyLogicInternalCodegenAPI
  public int _twins_DefaultValue_xjal() {
    final Person self = this;
    return 0;
  }

  public void set_twins( int twins ) {
    if (twins == this.twins) {
      return;
    }
    int _oldValue_xjal = this.twins;
    this.twins = twins;
    onChange_twins_xjal( _oldValue_xjal );
    onChange();
  }

  /**
   * Calls "On change" action for parameter twins.<br>
   * Note that 'oldValue' in that action will be unavailable if this method is called
by user
   * (current parameter value will be passed as 'oldValue').<br>
   * Please call <code>set_twins()</code> method instead.
   */
  protected void onChange_twins() {
    onChange_twins_xjal( twins );
  }
```

```
  @AnyLogicInternalCodegenAPI
  protected void onChange_twins_xjal( int oldValue ) {
  }


  public
int  unwanted;

  /**
   * Returns default value for parameter <code>unwanted</code>.
   * <i>This method should not be called by user</i>
   */
  @AnyLogicInternalCodegenAPI
  public int _unwanted_DefaultValue_xjal() {
    final Person self = this;
    return 0;
  }

  public void set_unwanted( int unwanted ) {
    if (unwanted == this.unwanted) {
      return;
    }
    int _oldValue_xjal = this.unwanted;
    this.unwanted = unwanted;
    onChange_unwanted_xjal( _oldValue_xjal );
    onChange();
  }

  /**
   * Calls "On change" action for parameter unwanted.<br>
   * Note that 'oldValue' in that action will be unavailable if this method is called
by user
   * (current parameter value will be passed as 'oldValue').<br>
   * Please call <code>set_unwanted()</code> method instead.
   */
  protected void onChange_unwanted() {
    onChange_unwanted_xjal( unwanted );
  }

  @AnyLogicInternalCodegenAPI
  protected void onChange_unwanted_xjal( int oldValue ) {
  }


  public
int  primip;

  /**
   * Returns default value for parameter <code>primip</code>.
   * <i>This method should not be called by user</i>
   */
  @AnyLogicInternalCodegenAPI
  public int _primip_DefaultValue_xjal() {
    final Person self = this;
    return 0;
  }

  public void set_primip( int primip ) {
    if (primip == this.primip) {
      return;
    }
    int _oldValue_xjal = this.primip;
```

```
    this.primip = primip;
    onChange_primip_xjal( _oldValue_xjal );
    onChange();
  }

  /**
   * Calls "On change" action for parameter primip.<br>
   * Note that 'oldValue' in that action will be unavailable if this method is called
by user
   * (current parameter value will be passed as 'oldValue').<br>
   * Please call <code>set_primip()</code> method instead.
   */
  protected void onChange_primip() {
    onChange_primip_xjal( primip );
  }

  @AnyLogicInternalCodegenAPI
  protected void onChange_primip_xjal( int oldValue ) {
  }


  public
int  blind;

  /**
   * Returns default value for parameter <code>blind</code>.
   * <i>This method should not be called by user</i>
   */
  @AnyLogicInternalCodegenAPI
  public int _blind_DefaultValue_xjal() {
    final Person self = this;
    return 0;
  }

  public void set_blind( int blind ) {
    if (blind == this.blind) {
      return;
    }
    int _oldValue_xjal = this.blind;
    this.blind = blind;
    onChange_blind_xjal( _oldValue_xjal );
    onChange();
  }

  /**
   * Calls "On change" action for parameter blind.<br>
   * Note that 'oldValue' in that action will be unavailable if this method is called
by user
   * (current parameter value will be passed as 'oldValue').<br>
   * Please call <code>set_blind()</code> method instead.
   */
  protected void onChange_blind() {
    onChange_blind_xjal( blind );
  }

  @AnyLogicInternalCodegenAPI
  protected void onChange_blind_xjal( int oldValue ) {
  }


  public
int  illiterate;
```

```
  /**
   * Returns default value for parameter <code>illiterate</code>.
   * <i>This method should not be called by user</i>
   */
  @AnyLogicInternalCodegenAPI
  public int _illiterate_DefaultValue_xjal() {
    final Person self = this;
    return 0;
  }

  public void set_illiterate( int illiterate ) {
    if (illiterate == this.illiterate) {
      return;
    }
    int _oldValue_xjal = this.illiterate;
    this.illiterate = illiterate;
    onChange_illiterate_xjal( _oldValue_xjal );
    onChange();
  }

  /**
   * Calls "On change" action for parameter illiterate.<br>
   * Note that 'oldValue' in that action will be unavailable if this method is called
by user
   * (current parameter value will be passed as 'oldValue').<br>
   * Please call <code>set_illiterate()</code> method instead.
   */
  protected void onChange_illiterate() {
    onChange_illiterate_xjal( illiterate );
  }

  @AnyLogicInternalCodegenAPI
  protected void onChange_illiterate_xjal( int oldValue ) {
  }


  public
int  spouseAge;

  /**
   * Returns default value for parameter <code>spouseAge</code>.
   * <i>This method should not be called by user</i>
   */
  @AnyLogicInternalCodegenAPI
  public int _spouseAge_DefaultValue_xjal() {
    final Person self = this;
    return 0;
  }

  public void set_spouseAge( int spouseAge ) {
    if (spouseAge == this.spouseAge) {
      return;
    }
    int _oldValue_xjal = this.spouseAge;
    this.spouseAge = spouseAge;
    onChange_spouseAge_xjal( _oldValue_xjal );
    onChange();
  }

  /**
   * Calls "On change" action for parameter spouseAge.<br>
   * Note that 'oldValue' in that action will be unavailable if this method is called
by user
```

```
   * (current parameter value will be passed as 'oldValue').<br>
   * Please call <code>set_spouseAge()</code> method instead.
   */
  protected void onChange_spouseAge() {
    onChange_spouseAge_xjal( spouseAge );
  }

  @AnyLogicInternalCodegenAPI
  protected void onChange_spouseAge_xjal( int oldValue ) {
  }


  public
int  risk;

  /**
   * Returns default value for parameter <code>risk</code>.
   * <i>This method should not be called by user</i>
   */
  @AnyLogicInternalCodegenAPI
  public int _risk_DefaultValue_xjal() {
    final Person self = this;
    return 0;
  }

  public void set_risk( int risk ) {
    if (risk == this.risk) {
      return;
    }
    int _oldValue_xjal = this.risk;
    this.risk = risk;
    onChange_risk_xjal( _oldValue_xjal );
    onChange();
  }

  /**
   * Calls "On change" action for parameter risk.<br>
   * Note that 'oldValue' in that action will be unavailable if this method is called
by user
   * (current parameter value will be passed as 'oldValue').<br>
   * Please call <code>set_risk()</code> method instead.
   */
  protected void onChange_risk() {
    onChange_risk_xjal( risk );
  }

  @AnyLogicInternalCodegenAPI
  protected void onChange_risk_xjal( int oldValue ) {
  }


  public
int  csPlanned;

  /**
   * Returns default value for parameter <code>csPlanned</code>.
   * <i>This method should not be called by user</i>
   */
  @AnyLogicInternalCodegenAPI
  public int _csPlanned_DefaultValue_xjal() {
    final Person self = this;
    return 0;
  }
```

```
  public void set_csPlanned( int csPlanned ) {
    if (csPlanned == this.csPlanned) {
      return;
    }
    int _oldValue_xjal = this.csPlanned;
    this.csPlanned = csPlanned;
    onChange_csPlanned_xjal( _oldValue_xjal );
    onChange();
  }

  /**
   * Calls "On change" action for parameter csPlanned.<br>
   * Note that 'oldValue' in that action will be unavailable if this method is called
by user
   * (current parameter value will be passed as 'oldValue').<br>
   * Please call <code>set_csPlanned()</code> method instead.
   */
  protected void onChange_csPlanned() {
    onChange_csPlanned_xjal( csPlanned );
  }

  @AnyLogicInternalCodegenAPI
  protected void onChange_csPlanned_xjal( int oldValue ) {
  }


  public
double  income;

  /**
   * Returns default value for parameter <code>income</code>.
   * <i>This method should not be called by user</i>
   */
  @AnyLogicInternalCodegenAPI
  public double _income_DefaultValue_xjal() {
    final Person self = this;
    return 0.0;
  }

  public void set_income( double income ) {
    if (income == this.income) {
      return;
    }
    double _oldValue_xjal = this.income;
    this.income = income;
    onChange_income_xjal( _oldValue_xjal );
    onChange();
  }

  /**
   * Calls "On change" action for parameter income.<br>
   * Note that 'oldValue' in that action will be unavailable if this method is called
by user
   * (current parameter value will be passed as 'oldValue').<br>
   * Please call <code>set_income()</code> method instead.
   */
  protected void onChange_income() {
    onChange_income_xjal( income );
  }

  @AnyLogicInternalCodegenAPI
  protected void onChange_income_xjal( double oldValue ) {
```

```
  }


  @Override
  public void setParametersToDefaultValues() {
    super.setParametersToDefaultValues();
    longitude = _longitude_DefaultValue_xjal();
    latitude = _latitude_DefaultValue_xjal();
    wealth = _wealth_DefaultValue_xjal();
    educSec = _educSec_DefaultValue_xjal();
    age = _age_DefaultValue_xjal();
    ANC4 = _ANC4_DefaultValue_xjal();
    urban = _urban_DefaultValue_xjal();
    twins = _twins_DefaultValue_xjal();
    unwanted = _unwanted_DefaultValue_xjal();
    primip = _primip_DefaultValue_xjal();
    blind = _blind_DefaultValue_xjal();
    illiterate = _illiterate_DefaultValue_xjal();
    spouseAge = _spouseAge_DefaultValue_xjal();
    risk = _risk_DefaultValue_xjal();
    csPlanned = _csPlanned_DefaultValue_xjal();
    income = _income_DefaultValue_xjal();
  }

  @Override
  public boolean setParameter(String _name_xjal, Object _value_xjal, boolean
_callOnChange_xjal) {
    switch ( _name_xjal ) {
    case "longitude":
      if ( _callOnChange_xjal ) {
        set_longitude( ((Number) _value_xjal).doubleValue() );
      } else {
        longitude = ((Number) _value_xjal).doubleValue();
      }
      return true;
    case "latitude":
      if ( _callOnChange_xjal ) {
        set_latitude( ((Number) _value_xjal).doubleValue() );
      } else {
        latitude = ((Number) _value_xjal).doubleValue();
      }
      return true;
    case "wealth":
      if ( _callOnChange_xjal ) {
        set_wealth( ((Number) _value_xjal).intValue() );
      } else {
        wealth = ((Number) _value_xjal).intValue();
      }
      return true;
    case "educSec":
      if ( _callOnChange_xjal ) {
        set_educSec( ((Number) _value_xjal).intValue() );
      } else {
        educSec = ((Number) _value_xjal).intValue();
      }
      return true;
    case "age":
      if ( _callOnChange_xjal ) {
        set_age( ((Number) _value_xjal).intValue() );
      } else {
        age = ((Number) _value_xjal).intValue();
      }
      return true;
```

```
case "ANC4":
  if ( _callOnChange_xjal ) {
    set_ANC4( ((Number) _value_xjal).intValue() );
  } else {
    ANC4 = ((Number) _value_xjal).intValue();
  }
  return true;
case "urban":
  if ( _callOnChange_xjal ) {
    set_urban( ((Number) _value_xjal).intValue() );
  } else {
    urban = ((Number) _value_xjal).intValue();
  }
  return true;
case "twins":
  if ( _callOnChange_xjal ) {
    set_twins( ((Number) _value_xjal).intValue() );
  } else {
    twins = ((Number) _value_xjal).intValue();
  }
  return true;
case "unwanted":
  if ( _callOnChange_xjal ) {
    set_unwanted( ((Number) _value_xjal).intValue() );
  } else {
    unwanted = ((Number) _value_xjal).intValue();
  }
  return true;
case "primip":
  if ( _callOnChange_xjal ) {
    set_primip( ((Number) _value_xjal).intValue() );
  } else {
    primip = ((Number) _value_xjal).intValue();
  }
  return true;
case "blind":
  if ( _callOnChange_xjal ) {
    set_blind( ((Number) _value_xjal).intValue() );
  } else {
    blind = ((Number) _value_xjal).intValue();
  }
  return true;
case "illiterate":
  if ( _callOnChange_xjal ) {
    set_illiterate( ((Number) _value_xjal).intValue() );
  } else {
    illiterate = ((Number) _value_xjal).intValue();
  }
  return true;
case "spouseAge":
  if ( _callOnChange_xjal ) {
    set_spouseAge( ((Number) _value_xjal).intValue() );
  } else {
    spouseAge = ((Number) _value_xjal).intValue();
  }
  return true;
case "risk":
  if ( _callOnChange_xjal ) {
    set_risk( ((Number) _value_xjal).intValue() );
  } else {
    risk = ((Number) _value_xjal).intValue();
  }
  return true;
```

```
    case "csPlanned":
      if ( _callOnChange_xjal ) {
        set_csPlanned( ((Number) _value_xjal).intValue() );
      } else {
        csPlanned = ((Number) _value_xjal).intValue();
      }
      return true;
    case "income":
      if ( _callOnChange_xjal ) {
        set_income( ((Number) _value_xjal).doubleValue() );
      } else {
        income = ((Number) _value_xjal).doubleValue();
      }
      return true;
    default:
      return super.setParameter( _name_xjal, _value_xjal, _callOnChange_xjal );
    }
  }

  @Override
  public <T> T getParameter(String _name_xjal) {
    Object _result_xjal;
    switch ( _name_xjal ) {
    case "longitude": _result_xjal = longitude; break;
    case "latitude": _result_xjal = latitude; break;
    case "wealth": _result_xjal = wealth; break;
    case "educSec": _result_xjal = educSec; break;
    case "age": _result_xjal = age; break;
    case "ANC4": _result_xjal = ANC4; break;
    case "urban": _result_xjal = urban; break;
    case "twins": _result_xjal = twins; break;
    case "unwanted": _result_xjal = unwanted; break;
    case "primip": _result_xjal = primip; break;
    case "blind": _result_xjal = blind; break;
    case "illiterate": _result_xjal = illiterate; break;
    case "spouseAge": _result_xjal = spouseAge; break;
    case "risk": _result_xjal = risk; break;
    case "csPlanned": _result_xjal = csPlanned; break;
    case "income": _result_xjal = income; break;
    default: _result_xjal = super.getParameter( _name_xjal ); break;
    }
    return (T) _result_xjal;
  }

  @AnyLogicInternalCodegenAPI
  private static String[] _parameterNames_xjal;

  @Override
  public String[] getParameterNames() {
    String[] result = _parameterNames_xjal;
    if (result == null) {
      List<String> list = new ArrayList<>( Arrays.asList( super.getParameterNames() )
);
      list.add( "longitude" );
      list.add( "latitude" );
      list.add( "wealth" );
      list.add( "educSec" );
      list.add( "age" );
      list.add( "ANC4" );
      list.add( "urban" );
      list.add( "twins" );
      list.add( "unwanted" );
      list.add( "primip" );
```

```
      list.add( "blind" );
      list.add( "illiterate" );
      list.add( "spouseAge" );
      list.add( "risk" );
      list.add( "csPlanned" );
      list.add( "income" );
      result = list.toArray( new String[ list.size() ] );
      _parameterNames_xjal = result;
    }
    return result;
  }
  // Plain Variables

  public
double
 startTime;
  public
double
 travelTime;
  /**
   * beta on wealth for class assignment
   */
  public
double
 bWealth;
  public
int
 provsIndex;
  public
int
 allowedIndex;
  public
double
 travelDistance;
  public
boolean
 distanceFurther;
  public
double
 costPaid;
  /**
   * Destination hospital
   */
  public
Hospital
 destHospital;
  /**
   * Destination hospital type
   */
  public
String
 destType;
  /**
   * Class membership
   */
  public
int
 segment;
  /**
   * difference in utility between optimal and chosen facility
   */
  public
double
```

```
 uDiff;
  /**
   * Destination hospital
   */
  public
Hospital
 optHospital;
  public
double
 selectedBobs;
  public
int
 deadBaby;
  public
boolean
 csPerformed;
  public
int
 catExp;

  // Collection Variables
  public
java.util.ArrayList <
Hospital > provs = new java.util.ArrayList<Hospital>();
  public
ArrayList <
Hospital > allowed = new ArrayList<Hospital>();
  public
ArrayList <
Hospital > allHospitals = new ArrayList<Hospital>();
  @AnyLogicInternalCodegenAPI
  private static Map<String, IElementDescriptor> elementDesciptors_xjal =
createElementDescriptors( Person.class );

  @AnyLogicInternalCodegenAPI
  @Override
  public Map<String, IElementDescriptor> getElementDesciptors() {
    return elementDesciptors_xjal;
  }
  @AnyLogicCustomProposalPriority(type =
AnyLogicCustomProposalPriority.Type.STATIC_ELEMENT)
  public static final Scale scale = new Scale( 10.0 );

  @Override
  public Scale getScale() {
    return scale;
  }





  // Statecharts
  public Statechart<statechart_state> statechart = new Statechart<>( this, (short)1 );

  @Override
  @AnyLogicInternalCodegenAPI
  public String getNameOf( Statechart _s ) {
    if(_s == this.statechart) return "statechart";
    return super.getNameOf( _s );
  }
```

```
@Override
@AnyLogicInternalCodegenAPI
public void executeActionOf( Statechart _s ) {
  if( _s == this.statechart ) {
    enterState( Parameterize, true );
    return;
  }
  super.executeActionOf( _s );
}

 // States of all statecharts

public enum statechart_state implements IStatechartState<Person, statechart_state> {
  Parameterize,
  classAssignment,
  Deciding,
  Traveling,
  atHospital;

  @AnyLogicInternalCodegenAPI
  private Collection<statechart_state> _simpleStatesDeep_xjal;

  @AnyLogicInternalCodegenAPI
  private Set<statechart_state> _fullState_xjal;

  @AnyLogicInternalCodegenAPI
  private Set<statechart_state> _statesInside_xjal;

  @Override
  @AnyLogicInternalCodegenAPI
  public Collection<statechart_state> getSimpleStatesDeep() {
    Collection<statechart_state> result = _simpleStatesDeep_xjal;
    if (result == null) {
      _simpleStatesDeep_xjal = result = calculateAllSimpleStatesDeep();
    }
    return result;
  }

  @Override
  public Set<statechart_state> getFullState() {
    Set<statechart_state> result = _fullState_xjal;
    if (result == null) {
      _fullState_xjal = result = calculateFullState();
    }
    return result;
  }

  @Override
  @AnyLogicInternalCodegenAPI
  public Set<statechart_state> getStatesInside() {
    Set<statechart_state> result = _statesInside_xjal;
    if (result == null) {
      _statesInside_xjal = result = calculateStatesInside();
    }
    return result;
  }

  @Override
  @AnyLogicInternalCodegenAPI
  public Statechart<statechart_state> getStatechart( Person _a ) {
    return _a.statechart;
  }
}
```

```
  @AnyLogicCustomProposalPriority(type =
AnyLogicCustomProposalPriority.Type.STATIC_ELEMENT)
  public static final statechart_state Parameterize = statechart_state.Parameterize;
  @AnyLogicCustomProposalPriority(type =
AnyLogicCustomProposalPriority.Type.STATIC_ELEMENT)
  public static final statechart_state classAssignment =
statechart_state.classAssignment;
  @AnyLogicCustomProposalPriority(type =
AnyLogicCustomProposalPriority.Type.STATIC_ELEMENT)
  public static final statechart_state Deciding = statechart_state.Deciding;
  @AnyLogicCustomProposalPriority(type =
AnyLogicCustomProposalPriority.Type.STATIC_ELEMENT)
  public static final statechart_state Traveling = statechart_state.Traveling;
  @AnyLogicCustomProposalPriority(type =
AnyLogicCustomProposalPriority.Type.STATIC_ELEMENT)
  public static final statechart_state atHospital = statechart_state.atHospital;


  @AnyLogicInternalCodegenAPI
  private void enterState( statechart_state _state, boolean _destination ) {
    switch( _state ) {
      case Parameterize:
          logToDBEnterState(statechart, _state);
        // (Simple state (not composite))
        statechart.setActiveState_xjal( Parameterize );
        {
this.destHospital = null;
this.primip = randomTrue(0.2445) ? 1 : 0;
this.illiterate = randomTrue(0.4049) ? 1 : 0;
this.blind = randomTrue(0.0005628518) ? 1 : 0;

//this.urban = randomTrue(0.1228679) ? 1 : 0;
if(main.Blantyre.contains(this.latitude, this.longitude) ||
main.Lilongwe.contains(this.latitude, this.longitude)) {
	this.urban = randomTrue(0.6) ? 1 : 0;} else {this.urban = 0;}
main.update_urbanPct();

this.educSec = randomTrue(0.1924819) ? 1 : 0;
this.twins = randomTrue(0.02279245) ? 1 : 0;
this.unwanted = randomTrue(0.09888285) ? 1 : 0;
this.ANC4 = randomTrue(0.462858) ? 1 : 0;
this.csPlanned = randomTrue(0.01585624) ? 1 : 0;

double r = Math.random();
if(r < 0.325) {
	this.risk = 0;
} else if(r < 0.787) {
	this.risk = 1;
} else if(r < 0.927) {
	this.risk = 2;
} else if(r < 0.997) {
	this.risk = 3;
} else {
	this.risk = 4;
}



;}
        transition2.start();
        return;
      case classAssignment:
          logToDBEnterState(statechart, _state);
```

```
        // (Simple state (not composite))
        statechart.setActiveState_xjal( classAssignment );
        {

this.bWealth = 0.0;
       switch (this.wealth) {
       case 5:
              this.bWealth = main.bRichest;
              this.circle.setFillColor(new Color(255,0,0, 40));
              this.circle.setLineColor(new Color(255,0,0, 100));
              break;
       case 4:
              this.bWealth = main.bRich;
              this.circle.setFillColor(new Color(192,0,64, 40));
              this.circle.setLineColor(new Color(192,0,64, 100));
              break;
       case 3:
              this.bWealth = main.bMiddle;
              this.circle.setFillColor(new Color(128,0,128, 40));
              this.circle.setLineColor(new Color(128,0,128, 100));
              break;
       case 2:
              this.bWealth = main.bPoor;
              this.circle.setFillColor(new Color(64,0,192, 40));
              this.circle.setLineColor(new Color(64,0,192, 100));
              break;
       case 1:
              this.bWealth = main.bPoorest;
              this.circle.setFillColor(new Color(0,0,255, 40));
              this.circle.setLineColor(new Color(0,0,255, 100));
              break;
       default: throw new IllegalAddException("No one with wealth "+ this.wealth);
       }


//Get betas for class selection

       //Note: wealth is obtained in the prior state
       double bBlind              = main.bBlind;
       double bIlliterate  = main.bIlliterate;
       double bPrimip             = main.bPrimip;
       double bIntercept   = main.bIntercept;
       double bUrban              = main.bUrban;
       double bAge         = main.bAge;
       double bSpouseAge   = main.bSpouseAge;
       double bEducSec            = main.bEducSec;
       double bTwins              = main.bTwins;
       double bUnwanted    = main.bUnwanted;
       double bANC4        = main.bANC4;
       double bRisk        = main.bRisk;
       double bCSPlanned   = main.bCSPlanned;

//Select class
       //double pClass2 = 1.0 / (1.0 + exp(-(bIntercept + bWealth + bBlind *
(double)this.blind + bIlliterate * (double)this.illiterate + bPrimip *
(double)this.primip
       //     + bUrban * (double)this.urban + bAge * (double)this.age + bSpouseAge *
(double)this.spouseAge + bEducSec * (double)this.educSec
       //     + bTwins * (double)this.twins + bUnwanted * (double)this.unwanted + bANC4
* (double)this.ANC4 + bRisk * (double)this.risk + bCSPlanned *
(double)this.csPlanned)));
```

```
	double pClass2 = 1.0 / (1.0 + exp(-(bIntercept + bWealth + bBlind * 
(double)this.blind + bIlliterate * (double)this.illiterate + bPrimip * 
(double)this.primip)));
	this.segment = randomTrue(pClass2) ? 2 : 1;
	main.update_segment2();
	main.update_classAssigned();


/*main.newClassAssignment.setCellValue(this.getId(), "Sheet1", this.getId() - 525, 1);
main.newClassAssignment.setCellValue(this.wealth, 	"Sheet1", this.getId() - 525, 3);
main.newClassAssignment.setCellValue(bWealth, 		"Sheet1", this.getId() - 525, 4);
main.newClassAssignment.setCellValue(this.blind, 	"Sheet1", this.getId() - 525, 5);
main.newClassAssignment.setCellValue(this.primip, 	"Sheet1", this.getId() - 525, 6);
main.newClassAssignment.setCellValue(this.illiterate,"Sheet1", this.getId() - 525, 7);
main.newClassAssignment.setCellValue(this.urban, 	"Sheet1", this.getId() - 525, 8);
main.newClassAssignment.setCellValue(this.age, 			"Sheet1", this.getId() - 
525, 9);
main.newClassAssignment.setCellValue(this.spouseAge,"Sheet1", this.getId() - 525, 10);
main.newClassAssignment.setCellValue(this.educSec, 	"Sheet1", this.getId() - 525, 
11);
main.newClassAssignment.setCellValue(this.twins, 	"Sheet1", this.getId() - 525, 
12);
main.newClassAssignment.setCellValue(this.unwanted, "Sheet1", this.getId() - 525, 13);
main.newClassAssignment.setCellValue(this.ANC4, 	"Sheet1", this.getId() - 525, 
14);
main.newClassAssignment.setCellValue(this.risk, 	"Sheet1", this.getId() - 525, 
15);
main.newClassAssignment.setCellValue(this.csPlanned,"Sheet1", this.getId() - 525, 16);
main.newClassAssignment.setCellValue(pClass2, 		"Sheet1", this.getId() - 525, 
17);
main.newClassAssignment.setCellValue(this.segment, 	"Sheet1", this.getId() - 525, 
18);*/


/* OLD CODE */


/*	for(Hospital h : provs){
		h.distance = h.distanceTo(this, KILOMETER);
	}

	Collections.sort(provs, new Comparator<Hospital>(){
		public int compare(Hospital a1, Hospital a2) {
			return 
Double.valueOf(a1.distance).compareTo(Double.valueOf(a2.distance));
		}
	});*/


/*
//Get betas for hospital selection
	double bDist = main.distance;
	double bQ = main.quality;
	double bBobs = main.bobs;
	double bFees = main.fees;

	double bCentral = main.centralHosp;
	double bDistrict = main.districtHosp;
	double bCommunity = main.communityHosp;
	double bOther = main.otherHosp;
	double bHealthCenter = main.healthCenter;
	double bMaternity = main.maternity;
	double bClinic = main.clinic;
```

```
        double bHome = 0.0;

//Get nearest hospital
        provs.clear();
        provs.addAll(main.hospitalList);

        for(Hospital h : provs){
                h.distance = h.distanceTo(this, KILOMETER);
        }

        Collections.sort(provs, new Comparator<Hospital>(){
                public int compare(Hospital a1, Hospital a2) {
                        return
Double.valueOf(a1.distance).compareTo(Double.valueOf(a2.distance));
                }
        });

        Hospital nearest = provs.get(0);

// Get the nearest 4 hospitals and average their characteristics
        double distAvg = 0.0;
        double feesAvg = 0.0;
        double bobsAvg = 0.0;
        double QAvg = 0.0;

        for(int i = 0; i < 4; i++){
                distAvg += provs.get(i).distance;
                QAvg    += provs.get(i).q;
                bobsAvg += provs.get(i).bobs;  //note that QI is added to the hospital
agent
                feesAvg += (double)provs.get(i).fees;
        }

        distAvg = distAvg / 4.0;
        bobsAvg = bobsAvg / 4.0;
        feesAvg = feesAvg / 4.0;
        QAvg = QAvg / 4.0;


// Upper model (simple logistic model)
        //double pNoCare = 1.0 / (1.0 + exp(bAge * this.age + bChildren *
(double)this.totalChildren + bANC4 * (double)this.ANC4 + bEduc * (double)this.educSec
+ bWealth + bSurvivingChildren * (double)this.survivingChildren + bUseSoap *
(double)this.useSoap + bMarried * (double)this.married + bUrban * (double)this.urban +
bUnintended * (double)this.unintended + bEthnicity + bReligion + bDistNearest *
nearest.distance + bQNearest * min(1.0, nearest.q + main.QI) + bBobsNearest *
nearest.bobs + bFeesNearest * (double)nearest.fees));
        double pNoCare = 1.0 / (1.0 + exp(bAge * this.age + bChildren *
(double)this.totalChildren + bANC4 * (double)this.ANC4 + bEduc * (double)this.educSec
+ bWealth + bSurvivingChildren * (double)this.survivingChildren + bUseSoap *
(double)this.useSoap + bMarried * (double)this.married + bUrban * (double)this.urban +
bUnintended * (double)this.unintended + bEthnicity + bReligion + bDistNearest *
distAvg + bQNearest * QAvg + bBobsNearest * bobsAvg + bFeesNearest * feesAvg));

        //System.out.println("Age\ttotalChildren\tANC4\tedSec\tWealth\tsurvivingChildre
n\tuseSoap\tmarried\turban\tunintended\tethnicity\treligion\tdist.avg\tq.avg\tbobs.avg
\tfees.avg");
        //System.out.println(this.age + "\t" + this.totalChildren + "\t" + this.ANC4 +
"\t" + this.educSec + "\t" + this.wealth  + "\t" + this.survivingChildren + "\t" +
this.useSoap + "\t" + this.married + "\t" + this.urban + "\t" + this.unintended + "\t"
+ this.ethnicity + "\t" + this.religion + "\t" + distAvg + "\t" + QAvg + "\t" +
bobsAvg + "\t" + feesAvg  + "\t" + pNoCare);
```

```
	//Deliver at home or choose a hospital?
	if(randomTrue(pNoCare)){
		this.destHospital = null;
		this.delHome = true;
		this.circle.setFillColor(Color.yellow);
		main.update_delHomeTotal();
		main.update_delHomePoorest();
		main.update_delHomePoor();
		main.update_delHomeMiddle();
		main.update_delHomeRich();
		main.update_delHomeRichest();
	} else {

// Lower model (conditional logistic)
		double denom = 0;
		for(Hospital h : provs){
			double U	= exp(h.bType + bDist * h.distanceTo(this, KILOMETER)
+ bQ * h.q + bBobs * h.bobs + bFees * (double)h.fees);  //note that QI is added to the
hospital agent
			h.u		= U;
			denom	+= U;
		}

		for(Hospital h : provs) {
			h.p = h.u / denom;
		}

		double r = Math.random();
		double cdf = 0.0;
		for(Hospital h : provs){
			cdf += h.p;
			if(r <= cdf){
				this.destHospital = h;
				break;
			}
		}
	}
*/
/*
// Calculate pNoCare
	double denom = 1.0; // because exp(0) = 1, included because home is one of the
options.
// This calculation is done based on the Malawi data, which only allows choices among
the three nearest hospitals
	for(Hospital h : topThree){
		double bType = 0.0;
		switch (h.type) {
			case "Central hospital":
				bType = main.centralHosp;
				break;
			case "District hospital":
				bType = main.districtHosp;
				break;
			case "Community hospital":
				bType = main.communityHosp;
				break;
			case "Other hospital":
				bType = main.otherHosp;
				break;
			case "Health center":
				bType = main.healthCenter;
				break;
			case "Maternity":
```

```
                        bType = main.maternity;
                        break;
                case "Clinic":
                        bType = main.clinic;
                        break;
                default: bType = 0.0;
            }
            double U      = exp(bAge * this.age + bChildren *
(double)this.totalChildren + bEduc * (double)this.educSec + bWealth + bType + bDist *
h.distanceTo(this, KILOMETER) + bQ * h.q + bBobs * h.bobs + bFees * (double)h.fees);
            h.u                = U;
            denom        += U;

        }

        if(randomFalse(1/denom)) { // remember: 1/denom = pHome
            denom = 0.0; //0 now, because home is out of the picture
            for(Hospital h : provs){
                double bType = 0.0;
                switch (h.type) {
                    case "Central hospital":
                        bType = main.centralHosp;
                        break;
                    case "District hospital":
                        bType = main.districtHosp;
                        break;
                    case "Community hospital":
                        bType = main.communityHosp;
                        break;
                    case "Other hospital":
                        bType = main.otherHosp;
                        break;
                    case "Health center":
                        bType = main.healthCenter;
                        break;
                    case "Maternity":
                        bType = main.maternity;
                        break;
                    case "Clinic":
                        bType = main.clinic;
                        break;
                    default: bType = 0.0;
                }
                double U      = exp(bAge * this.age + bChildren *
(double)this.totalChildren + bEduc * (double)this.educSec + bWealth + bType + bDist *
h.distanceTo(this, KILOMETER) + bQ * h.q + bBobs * h.bobs + bFees * (double)h.fees);
                h.u                = U;
                denom        += U;
            }

            for(Hospital h : provs) {
                h.p = h.u / denom;
            }

            double r = Math.random();
            double cdf = 0.0;
            for(Hospital h : provs){
                cdf += h.p;
                if(r <= cdf){
                    this.destHospital = h;
                    break;
                }
            }
```

```
        } else {
              this.destHospital = null;
              this.delHome = true;
              this.circle.setFillColor(Color.yellow);
              main.update_delHomeTotal();
              main.update_delHomePoorest();
              main.update_delHomePoor();
              main.update_delHomeMiddle();
              main.update_delHomeRich();
              main.update_delHomeRichest();
        }

 */

        //double bDistNearest = main.distanceNearest;
        //double bQNearest = main.qualityNearest;
        //double bBobsNearest = main.bobsNearest;
        //double bFeesNearest = main.feesNearest;

        //double bChildren = main.totalChildren;
        //double bSurvivingChildren = main.survivingChildren;
        //double bUseSoap = main.useSoap;
        //double bMarried = main.married;
        //double bUnintended = main.unintended;
        /*
        double bEthnicity = 0.0;
        switch(this.ethnicity) {
        case "Tumbuka":
              bEthnicity = main.tumbuka;
              break;
        case "Lomwe":
              bEthnicity = main.lomwe;
              break;
        case "Tonga":
              bEthnicity = main.tonga;
              break;
        case "Yao":
              bEthnicity = main.yao;
              break;
        case "Sena":
              bEthnicity = main.sena;
              break;
        case "Nkhonde":
              bEthnicity = main.nkhonde;
              break;
        case "Ngoni":
              bEthnicity = main.ngoni;
              break;
        case "Other":
              bEthnicity = main.otherRace;
              break;
        case "Missing":
              bEthnicity = main.missingRace;
              break;
        case "Chewa":
              bEthnicity = main.chewa;
              break;
        default:
              throw new IllegalArgumentException("Invalid race: " + this.ethnicity);
        }

        double bReligion = 0.0;
        switch(this.religion) {
```

```
        case "CCAP":
                bReligion = main.ccap;
                break;
        case "Anglican":
                bReligion = main.anglican;
                break;
        case "Seventh Day adventist":
                bReligion = main.seventhDay;
                break;
        case "Other Christian":
                bReligion = main.otherChristian;
                break;
        case "Muslim":
                bReligion = main.muslim;
                break;
        case "No religion":
                bReligion = main.noReligion;
                break;
        case "Other religion":
                bReligion = main.otherReligion;
                break;
        case "Catholic":
                bReligion = main.catholic;
                break;
        default:
                throw new IllegalArgumentException("Invalid religion: " + this.religion);
        }*/
;}
         transition3.start();
         return;
       case Deciding:
            logToDBEnterState(statechart, _state);
         // (Simple state (not composite))
         statechart.setActiveState_xjal( Deciding );
         {
//Add hospitals
        provs.clear();
        allowed.clear();
        allHospitals.clear();

        allHospitals.addAll(main.hospitalList);
        //allowed.addAll(main.allowedList);

        for(Hospital h : allHospitals){
                h.distance = h.distanceTo(this, KILOMETER);
                if(h.distance <= 100.0)
                {
                        provs.add(h);
                        if(h.doIExist)
                        {
                                allowed.add(h);
                        }
                }
        }

//      for(Hospital h : allowed){
//              h.distance = h.distanceTo(this, KILOMETER);
//      }


//Select hospitals based on class
if(this.segment == 1){
        double bDist = main.bDistance1;
```

```
        double bQ = main.bQuality1;
        double bBobs = main.bBobs1;
        double bFees = main.bFees1;

        double denom = 0.0;
        for(Hospital h : allowed)
        {
               h.u                  = exp(h.bType1 + bDist * ((h.distance - 
main.distCenter)/main.distRMS) + bBobs * h.bobs + bFees * (double)h.fees);  //note 
that QI is added to the hospital agent
               denom        += h.u;
        }

        for(Hospital h : allowed)
        {
               h.p = h.u / denom;
        }

        double r = Math.random();

        double cdf = 0.0;
        for(Hospital h : allowed)
        {
               cdf += h.p;
               //System.out.print(this.getId() + "\t" + this.segment + "\t" + h.getId() 
+ "\t" + h.type + "\t" + h.bType1 + "\t" + h.distanceTo(this, KILOMETER) + "\t" + 
h.bobs + "\t" + h.fees + "\t" + h.q + "\t" + h.u + "\t" + denom + "\t" + h.p + "\t" + 
cdf + "\t" + r + "\t");
               if(r <= cdf){
                      this.destHospital = h;
                      main.update_hospitalAssigned();
                      //System.out.println(this.destHospital.getId());
                      break;
               } //else System.out.println("null");
        }

        if(main.strategy == 0){
               this.optHospital = this.destHospital;
        } else {
               denom = 0.0;
               for(Hospital h : provs)
               {
                      h.u                  = exp(h.bType1 + bDist * ((h.distance - 
main.distCenter)/main.distRMS) + bBobs * h.bobsCounterfactual + bFees * 
(double)h.feesCounterfactual);  //note that QI is added to the hospital agent
                      denom        += h.u;
               }

               for(Hospital h : provs)
               {
                      h.p = h.u / denom;
               }

               r = Math.random();

               cdf = 0.0;
               for(Hospital h : provs)
               {
                      cdf += h.p;
                      if(r <= cdf){
                             this.optHospital = h;
                             break;
                      }
```

```
            }
        }
        this.uDiff = this.optHospital.u - this.destHospital.u;

} else if(this.segment == 2){
        double bDist = main.bDistance2;
        double bQ = main.bQuality2;
        double bBobs = main.bBobs2;
        double bFees = main.bFees2;

        double denom = 0.0;
        for(Hospital h : allowed)
        {
            h.u                 = exp(h.bType2 + bDist * ((h.distance -
main.distCenter)/main.distRMS) + bBobs * h.bobs + bFees * (double)h.fees);  //note
that QI is added to the hospital agent
            denom        += h.u;
        }

        for(Hospital h : allowed)
        {
            h.p = h.u / denom;
        }

        double r = Math.random();

        double cdf = 0.0;
        for(Hospital h : allowed)
        {
            cdf += h.p;
            if(r <= cdf){
                this.destHospital = h;
                main.update_hospitalAssigned();
                break;
            }
        }

        if(main.strategy == 0){
            this.optHospital = this.destHospital;
        } else {
            denom = 0.0;
            for(Hospital h : provs)
            {
                h.u                 = exp(h.bType2 + bDist * ((h.distance -
main.distCenter)/main.distRMS) + bBobs * h.bobsCounterfactual + bFees *
(double)h.feesCounterfactual);  //note that QI is added to the hospital agent
                denom        += h.u;
            }

            for(Hospital h : provs)
            {
                h.p = h.u / denom;
            }

            r = Math.random();

            cdf = 0.0;
            for(Hospital h : provs)
            {
                cdf += h.p;
                if(r <= cdf){
                    this.optHospital = h;
                    break;
```

```
                    }
            }
        }
        this.uDiff = this.optHospital.u - this.destHospital.u;
} else throw new IllegalArgumentException("No class assigned for agent " +
this.agentInfo());


//Neonatal mortality
//this.deadBaby = randomTrue(this.destHospital.mortality + Math.random() * 0.005) ? 1
: 0;
this.deadBaby = randomTrue(this.destHospital.mortality) ? 1 : 0;

//Cost
//calculate the cost they would have paid at their optimal hospital

//Note C/S rate in central hospitals is 9% and 1% in rural hospitals
(https://www.ncbi.nlm.nih.gov/pmc/articles/PMC194081/)
// This paper has a C/S rate of 9.74% in government hospitals and 5.45% in mission
hospitals (8.98% overall in hospitals):
http://citeseerx.ist.psu.edu/viewdoc/download?doi=10.1.1.516.617&rep=rep1&type=pdf

//OOP cost for vaginal delivery in Malawi is $7.69 in hospitals and 0.35 in health
centers
(http://citeseerx.ist.psu.edu/viewdoc/download?doi=10.1.1.516.617&rep=rep1&type=pdf)
//Cost of C/S from Blake's paper = 133.  Conservative, compared with this paper:
http://www.niche1.nl/resources/content/publication_file_189_pub_dennis.pdf (costs =
$164 - $638)

//C section rate in Malawi is 4.6%
(https://www.unicef.org/infobycountry/malawi_statistics.html)
// 65% of births happen in a health center, with an additional 1% at a maternity /
clinic


//I will assume:
// Nobody in a BEMOnC facility gets a C/S
// 8.98% of people in a CEMOnC facility get a C/S
// In BEMOnC facilities, cost will be assigned by whether it's a hospital or not.
// I'll pick the conservative cost for a C/S of $133

//Draw parameters
Random r = new MersenneTwisterRNG();  //from uncommon maths package (found in the
/Mark/Models folder)

if(this.destHospital.cs)
{
        if(randomTrue(0.0974 + 0.05))
        {
                this.costPaid = max(7.69, new GaussianGenerator(133.0, 0.25 * 133.0,
r).nextValue()); //cost of a CS can never be less than the average cost of an SVD
                this.csPerformed = true;
        }
}
else this.costPaid = this.destHospital.hospital ? max(0.0, new GaussianGenerator(7.69,
0.25 * 7.69, r).nextValue()) : max(0.0, new GaussianGenerator(0.35, 0.25 * 0.35,
r).nextValue());

if(this.costPaid >= 0.10 * this.income)
{
        this.catExp = 1;
}
```

```
//System.out.println("Agent " + this.getId() + "\tdestHosp = " + this.destHospital.id
+ "\tuDest = " + this.destHospital.u + "\toptHosp = " + this.optHospital.id + "\tuDest
= " + this.optHospital.u + "\tuDiff = " + this.uDiff);

//System.out.println(this.getId() + "\t" + this.segment + "\t" +
this.destHospital.getId() + "\t" + this.destHospital.distance + "\t" +
this.destHospital.type + "\t" + this.destHospital.bobs + "\t" + this.destHospital.fees
+ "\t" + this.destHospital.q + "\t" + this.destHospital.u + "\t" + this.destHospital.p
+
//      "\t" + this.optHospital.getId() + "\t" + this.optHospital.distance + "\t" +
this.optHospital.type + "\t" + this.optHospital.bobs + "\t" + this.optHospital.fees +
"\t" + this.optHospital.q + "\t" + this.optHospital.u + "\t" + this.optHospital.p +
"\t" + this.uDiff );
this.destType = this.destHospital.type;
main.chart.updateData();
main.update_utilityDifference();
main.uDiff.add(this.uDiff / 100000.0);
main.uDiffHistogram.updateData();
main.travelDistance.add(this.destHospital.distance);
this.travelDistance = this.destHospital.distance;
main.update_meanDistance();
main.update_dist60km();
this.distanceFurther = this.destHospital.distance > this.optHospital.distance;
main.update_distFurther();
main.selectedBobs.add(this.destHospital.bobs);
main.selectedBobsHist.updateData();
main.update_meanBobs();
main.mortChart.updateData();
main.update_neonatalMortality();
main.update_csRate();
main.update_avgOOP();
main.update_catExpTotal();
main.update_catExpPoorest();
main.update_catExpPoor();
main.update_catExpMiddle();
main.update_catExpRich();
main.update_catExpRichest();

/*
main.newClassAssignment.setCellValue(main.strategy,                      "Sheet1",
this.getId() - 526, 1);
main.newClassAssignment.setCellValue(this.getId(),
       "Sheet1", this.getId() - 526, 2);
main.newClassAssignment.setCellValue(this.destHospital.getId(),    "Sheet1",
this.getId() - 526, 3);
main.newClassAssignment.setCellValue(this.destHospital.type,             "Sheet1",
this.getId() - 526, 4);
main.newClassAssignment.setCellValue(this.destHospital.upgradeCS,  "Sheet1",
this.getId() - 526, 5);
main.newClassAssignment.setCellValue(this.destHospital.upgradeBemonc,"Sheet1",
this.getId() - 526, 6);
main.newClassAssignment.setCellValue(this.destHospital.cs,               "Sheet1",
this.getId() - 526, 7);
main.newClassAssignment.setCellValue(this.destHospital.csVar,            "Sheet1",
this.getId() - 526, 8);
main.newClassAssignment.setCellValue(this.destHospital.bemonc,           "Sheet1",
this.getId() - 526, 9);
main.newClassAssignment.setCellValue(this.destHospital.bemoncVar,  "Sheet1",
this.getId() - 526, 10);
main.newClassAssignment.setCellValue(this.destHospital.doIExist,   "Sheet1",
this.getId() - 526, 11);
```

```
main.newClassAssignment.setCellValue(this.destHospital.mortality,  "Sheet1",
this.getId() - 526, 12);
main.newClassAssignment.setCellValue(this.deadBaby,                        "Sheet1",
this.getId() - 526, 13);
*/


/*
main.distanceWTP.setCellValue(this.getId(),                                "Sheet1",
this.getId() - 526, 1);
main.distanceWTP.setCellValue(this.segment,                                "Sheet1",
this.getId() - 526, 2);
main.distanceWTP.setCellValue((this.segment == 1 ? main.bDistance1 : main.bDistance2),
                           "Sheet1", this.getId() - 526, 3);
main.distanceWTP.setCellValue((this.segment == 1 ? main.bBobs1 : main.bBobs2),
                                  "Sheet1", this.getId() - 526, 4);
main.distanceWTP.setCellValue((this.segment == 1 ? main.bFees1 : main.bFees2),
                                  "Sheet1", this.getId() - 526, 5);
main.distanceWTP.setCellValue((this.segment == 1 ? this.destHospital.bType1 :
this.destHospital.bType2),"Sheet1", this.getId() - 526, 6);
main.distanceWTP.setCellValue(this.destHospital.distance,         "Sheet1",
this.getId() - 526, 7);
main.distanceWTP.setCellValue(this.destHospital.bobs,                      "Sheet1",
this.getId() - 526, 8);
main.distanceWTP.setCellValue(this.destHospital.fees,                      "Sheet1",
this.getId() - 526, 9);
main.distanceWTP.setCellValue(this.destHospital.u,                         "Sheet1",
this.getId() - 526, 10);
main.distanceWTP.setCellValue((this.segment == 1 ? this.optHospital.bType1 :
this.optHospital.bType2),  "Sheet1", this.getId() - 526, 11);
main.distanceWTP.setCellValue(this.optHospital.distance,          "Sheet1",
this.getId() - 526, 12);
main.distanceWTP.setCellValue(this.optHospital.bobs,              "Sheet1",
this.getId() - 526, 13);
main.distanceWTP.setCellValue(this.optHospital.fees,              "Sheet1",
this.getId() - 526, 14);
main.distanceWTP.setCellValue(this.optHospital.u,                          "Sheet1",
this.getId() - 526, 15);
main.distanceWTP.setCellValue(this.deadBaby,                               "Sheet1",
this.getId() - 526, 16);
main.distanceWTP.setCellValue(main.strategy,                               "Sheet1",
this.getId() - 526, 17);
*/
;}
        transition.start();
        return;
      case Traveling:
           logToDBEnterState(statechart, _state);
        // (Simple state (not composite))
        statechart.setActiveState_xjal( Traveling );
        {
this.startTime = time(SECOND);
this.moveTo(this.destHospital);

;}
        transition1.start();
        return;
      case atHospital:
           logToDBEnterState(statechart, _state);
        // (Simple state (not composite))
        statechart.setActiveState_xjal( atHospital );
        {
this.travelTime = (time(SECOND) - this.startTime) / 3600.0;
```

```
main.update_travelTime();


;}
        return;
      default:
        return;
    }
  }

  @AnyLogicInternalCodegenAPI
  private void exitState( statechart_state _state, Transition _t, boolean _source ) {
    switch( _state ) {
      case Parameterize:
          logToDBExitState(statechart, _state);
          logToDB(statechart, _t, _state);
      // (Simple state (not composite))
        if ( !_source || _t != transition2) transition2.cancel();
        return;
      case classAssignment:
          logToDBExitState(statechart, _state);
          logToDB(statechart, _t, _state);
      // (Simple state (not composite))
        if ( !_source || _t != transition3) transition3.cancel();
        return;
      case Deciding:
          logToDBExitState(statechart, _state);
          logToDB(statechart, _t, _state);
      // (Simple state (not composite))
        if ( !_source || _t != transition) transition.cancel();
        return;
      case Traveling:
          logToDBExitState(statechart, _state);
          logToDB(statechart, _t, _state);
      // (Simple state (not composite))
        if ( !_source || _t != transition1) transition1.cancel();
        return;
      case atHospital:
          logToDBExitState(statechart, _state);
          logToDB(statechart, _t, _state);
      // (Simple state (not composite))
        return;
      default:
        return;
    }
  }

  @AnyLogicInternalCodegenAPI
  private void exitInnerStates( statechart_state _destination ) {
    statechart_state _state = statechart.getActiveSimpleState();
    while( _state != _destination ) {
                exitState( _state, null, false );
                _state = _state.getContainerState();
          }
      }

  public TransitionTimeout transition2 = new TransitionTimeout( this );
  public TransitionTimeout transition3 = new TransitionTimeout( this );

  @Override
  @AnyLogicInternalCodegenAPI
  public String getNameOf( TransitionTimeout _t ) {
    if ( _t == transition2 ) return "transition2";
```

```
    if ( _t == transition3 ) return "transition3";
    return super.getNameOf( _t );
  }

  @Override
  @AnyLogicInternalCodegenAPI
  public Statechart getStatechartOf( TransitionTimeout _t ) {
    if ( _t == transition2 ) return statechart;
    if ( _t == transition3 ) return statechart;
    return super.getStatechartOf( _t );
  }

  @Override
  @AnyLogicInternalCodegenAPI
  public void executeActionOf( TransitionTimeout _t ) {
    if ( _t == transition2 ) {
      exitState( Parameterize, _t, true );
          enterState( classAssignment, true );
      return;
    }
    if ( _t == transition3 ) {
      exitState( classAssignment, _t, true );
          enterState( Deciding, true );
      return;
    }
    super.executeActionOf( _t );
  }
  @Override
  @AnyLogicInternalCodegenAPI
  public double evaluateTimeoutOf( TransitionTimeout _t ) {
    double _value;
    if ( _t == transition2 ) {
      _value =
1
;
      _value = toModelTime( _value, SECOND );
      return _value;
    }
    if ( _t == transition3 ) {
      _value =
1
;
      _value = toModelTime( _value, SECOND );
      return _value;
    }
    return super.evaluateTimeoutOf( _t );
  }

  public TransitionCondition transition = new TransitionCondition( this );

  @Override
  @AnyLogicInternalCodegenAPI
  public String getNameOf( TransitionCondition _t ) {
    if ( _t == transition ) return "transition";
    return super.getNameOf( _t );
  }

  @Override
  @AnyLogicInternalCodegenAPI
  public Statechart getStatechartOf( TransitionCondition _t ) {
    if ( _t == transition ) return statechart;
    return super.getStatechartOf( _t );
  }
```

```
  @Override
  @AnyLogicInternalCodegenAPI
  public void executeActionOf( TransitionCondition _t ) {
    if ( _t == transition ) {
      exitState( Deciding, _t, true );
          enterState( Traveling, true );
      return;
    }
    super.executeActionOf( _t );
  }
  @Override
  @AnyLogicInternalCodegenAPI
  public boolean testConditionOf( TransitionCondition _t ) {
    if ( _t == transition ) return
//this.destHospital != null
false
;
    return super.testConditionOf( _t );
  }


  public TransitionMessage transition1 = new TransitionMessage( this );

  @Override
  @AnyLogicInternalCodegenAPI
  public String getNameOf( TransitionMessage _t ) {
    if ( _t == transition1 ) return "transition1";
    return super.getNameOf( _t );
  }

  @Override
  @AnyLogicInternalCodegenAPI
  public Statechart getStatechartOf( TransitionMessage _t ) {
    if ( _t == transition1 ) return statechart;
    return super.getStatechartOf( _t );
  }

  @Override
  @AnyLogicInternalCodegenAPI
  public void executeActionOf( TransitionMessage _t, Object _msg ) {
    if ( _t == transition1 ) {
      exitState( Traveling, _t, true );
          enterState( atHospital, true );
      return;
    }
    super.executeActionOf( _t, _msg );
  }
  @Override
  @AnyLogicInternalCodegenAPI
  public boolean testMessageOf( TransitionMessage _t, Object _msg ) {
    if ( _t == transition1 ) {
      return _msg == _ARRIVAL_message_xjal;
    }
    return super.testMessageOf( _t, _msg );
  }
  // View areas
  public ViewArea _origin_VA = new ViewArea( this, "[Origin]", 0, 0, 1550.0, 890.0 );
  @Override
  @AnyLogicInternalCodegenAPI
  public int getViewAreas(Map<String, ViewArea> _output) {
    if ( _output != null ) {
      _output.put( "_origin_VA", this._origin_VA );
```

```
    }
    return 1 + super.getViewAreas( _output );
  }
  protected static final Color _circle_Fill_Color = new Color( 0x1DFF00FF, true );
  @AnyLogicInternalCodegenAPI
  protected static final int _circle = 1;

  /** Internal constant, shouldn't be accessed by user */
  @AnyLogicInternalCodegenAPI
  protected static final int _SHAPE_NEXT_ID_xjal = 2;


  /**
   * Top-level presentation group id
   */
  @AnyLogicInternalCodegenAPI
  protected static final int _presentation = 0;

  @AnyLogicInternalCodegenAPI
  public boolean isPublicPresentationDefined() {
    return true;
  }

  @AnyLogicInternalCodegenAPI
  public boolean isEmbeddedAgentPresentationVisible( Agent _a ) {
    return super.isEmbeddedAgentPresentationVisible( _a );
  }
  /**
   * Top-level icon group id
   */
  @AnyLogicInternalCodegenAPI
  protected static final int _icon = -1;


  @Override
  @AnyLogicInternalCodegenAPI
  public boolean onShapeClick( int _shape, int index, double clickx, double clicky ){
    switch( _shape ){
      case _circle:
        if (true) {
          ShapeOval self = this.circle;

main.vAgentID = this.getLatitude();
        }
        break;
      default: return super.onShapeClick( _shape, index, clickx, clicky );
       }
       return false;
  }

  protected ShapeOval circle;
  @AnyLogicInternalCodegenAPI
  private void _createPersistentElementsBP0_xjal() {
    circle = new ShapeOval(
       SHAPE_DRAW_2D3D, true,0.0, 0.0, 0.0, 0.0,
       purple, _circle_Fill_Color,
          2.0, 2.0, 0.0, 1.0, LINE_STYLE_SOLID ) {

      @Override
      @AnyLogicInternalCodegenAPI
      public boolean onClick( double clickx, double clicky ) {
        return onShapeClick( _circle, 0, clickx, clicky );
      }
```

```
    };

  }

  @AnyLogicInternalCodegenAPI
  private void _createPersistentElementsAP0_xjal() {
  }


  // Static initialization of persistent elements
  {
    _createPersistentElementsBP0_xjal();
  }
  protected ShapeTopLevelPresentationGroup presentation;
  protected ShapeModelElementsGroup icon;

  @Override
  @AnyLogicInternalCodegenAPI
  public ShapeTopLevelPresentationGroup getPresentationShape() {
    return presentation;
  }

  @Override
  @AnyLogicInternalCodegenAPI
  public ShapeModelElementsGroup getModelElementsShape() {
    return icon;
  }

  @Override
  @AnyLogicInternalCodegenAPI
  public Object getPersistentShape( int _shape ) {
    switch (_shape) {
      case _presentation: return presentation;
      case _icon: return icon;
      case _circle: return circle;
      default: return super.getPersistentShape( _shape );
    }
  }

  @Override
  @AnyLogicInternalCodegenAPI
  public String getNameOfShape_xjal( Object _shape ) {
    try {
      if ( _shape == null ) return null;
      String _name_xjal;
      _name_xjal = checkNameOfShape_xjal( _shape, presentation, "presentation" ); if
(_name_xjal != null) return _name_xjal;
      _name_xjal = checkNameOfShape_xjal( _shape, icon, "icon" ); if (_name_xjal !=
null) return _name_xjal;
      _name_xjal = checkNameOfShape_xjal( _shape, circle, "circle" ); if (_name_xjal
!= null) return _name_xjal;
    } catch (Exception e) {
      return null;
    }
    return super.getNameOfShape_xjal( _shape );
  }
  @AnyLogicInternalCodegenAPI
  protected static final int[] _transition2_pointsX_xjal = {310, 310, };
  @AnyLogicInternalCodegenAPI
  protected static final int[] _transition2_pointsY_xjal = {90, 140, };

  @AnyLogicInternalCodegenAPI
  protected static final int[] _transition3_pointsX_xjal = {370, 440, };
```

```
  @AnyLogicInternalCodegenAPI
  protected static final int[] _transition3_pointsY_xjal = {150, 180, };

  @AnyLogicInternalCodegenAPI
  protected static final int[] _transition_pointsX_xjal = {440, 360, };
  @AnyLogicInternalCodegenAPI
  protected static final int[] _transition_pointsY_xjal = {210, 240, };

  @AnyLogicInternalCodegenAPI
  protected static final int[] _transition1_pointsX_xjal = {310, 310, };
  @AnyLogicInternalCodegenAPI
  protected static final int[] _transition1_pointsY_xjal = {250, 300, };


  @AnyLogicInternalCodegenAPI
  private void drawModelElements_Statecharts_xjal(Panel _panel, Graphics2D _g, boolean
_publicOnly, boolean _isSuperClass ) {
    if (!_publicOnly) {
      drawState( _panel, _g, 260, 140, 110, 30, 10, 10, "classAssignment", GOLD,
classAssignment, statechart );
    }
    if (!_publicOnly) {
      drawState( _panel, _g, 260, 220, 100, 30, 10, 10, "Traveling", GOLD, Traveling,
statechart );
    }
    if (!_publicOnly) {
      drawState( _panel, _g, 260, 300, 100, 30, 10, 10, "atHospital", GOLD,
atHospital, statechart );
    }
    if (!_publicOnly) {
      drawState( _panel, _g, 260, 60, 100, 30, 10, 10, "Parameterize", GOLD,
Parameterize, statechart );
    }
    if (!_publicOnly) {
      drawState( _panel, _g, 390, 180, 110, 30, 10, 10, "Deciding", GOLD, Deciding,
statechart );
    }
    if (!_publicOnly) {
      drawStatechartEntryPoint( _panel, _g, 310, 20, 310, 60, 320, 20, "statechart",
statechart );
    }
    if (!_publicOnly) {
      drawTransition(_panel, _g, _transition_pointsX_xjal, _transition_pointsY_xjal,
450, 210, null, transition, TransitionIcon.CONDITION, 421, 217 );
    }
    if (!_publicOnly) {
      drawTransition(_panel, _g, _transition1_pointsX_xjal, _transition1_pointsY_xjal,
320, 250, null, transition1, TransitionIcon.ARRIVAL, 310, 270 );
    }
    if (!_publicOnly) {
      drawTransition(_panel, _g, _transition2_pointsX_xjal, _transition2_pointsY_xjal,
320, 90, null, transition2, TransitionIcon.TIMEOUT, 310, 110 );
    }
    if (!_publicOnly) {
      drawTransition(_panel, _g, _transition3_pointsX_xjal, _transition3_pointsY_xjal,
380, 150, null, transition3, TransitionIcon.TIMEOUT, 388, 157 );
    }
  }

  @AnyLogicInternalCodegenAPI
  private void drawModelElements_Parameters_xjal(Panel _panel, Graphics2D _g, boolean
_publicOnly, boolean _isSuperClass ) {
    if (!_publicOnly) {
```

```
      drawParameter( _panel, _g, 50, 50, 10, 0, "longitude", longitude, 0 );
    }
    if (!_publicOnly) {
      drawParameter( _panel, _g, 50, 100, 10, 0, "latitude", latitude, 0 );
    }
    if (!_publicOnly) {
      drawParameter( _panel, _g, 50, 150, 10, 0, "wealth", wealth, 0 );
    }
    if (!_publicOnly) {
      drawParameter( _panel, _g, 50, 500, 10, 0, "educSec", educSec, 0 );
    }
    if (!_publicOnly) {
      drawParameter( _panel, _g, 50, 400, 10, 0, "age", age, 0 );
    }
    if (!_publicOnly) {
      drawParameter( _panel, _g, 50, 650, 10, 0, "ANC4", ANC4, 0 );
    }
    if (!_publicOnly) {
      drawParameter( _panel, _g, 50, 350, 10, 0, "urban", urban, 0 );
    }
    if (!_publicOnly) {
      drawParameter( _panel, _g, 50, 550, 10, 0, "twins", twins, 0 );
    }
    if (!_publicOnly) {
      drawParameter( _panel, _g, 50, 600, 10, 0, "unwanted", unwanted, 0 );
    }
    if (!_publicOnly) {
      drawParameter( _panel, _g, 50, 200, 10, 0, "primip", primip, 0 );
    }
    if (!_publicOnly) {
      drawParameter( _panel, _g, 50, 250, 10, 0, "blind", blind, 0 );
    }
    if (!_publicOnly) {
      drawParameter( _panel, _g, 50, 300, 10, 0, "illiterate", illiterate, 0 );
    }
    if (!_publicOnly) {
      drawParameter( _panel, _g, 50, 450, 10, 0, "spouseAge", spouseAge, 0 );
    }
    if (!_publicOnly) {
      drawParameter( _panel, _g, 50, 700, 10, 0, "risk", risk, 0 );
    }
    if (!_publicOnly) {
      drawParameter( _panel, _g, 50, 750, 10, 0, "csPlanned", csPlanned, 0 );
    }
    if (!_publicOnly) {
      drawParameter( _panel, _g, 50, 800, 10, 0, "income", income, 0 );
    }
  }

  @AnyLogicInternalCodegenAPI
  private void drawModelElements_PlainVariables_xjal(Panel _panel, Graphics2D _g,
boolean _publicOnly, boolean _isSuperClass ) {
    if (!_publicOnly) {
      drawPlainVariable( _panel, _g, 150, 150, 10, 0, "startTime", startTime, false );
    }
    if (!_publicOnly) {
      drawPlainVariable( _panel, _g, 150, 200, 10, 0, "travelTime", travelTime, false
);
    }
    if (!_publicOnly) {
      drawPlainVariable( _panel, _g, 150, 300, 10, 0, "bWealth", bWealth, false );
    }
    if (!_publicOnly) {
```

```
      drawPlainVariable( _panel, _g, 150, 430, 10, 0, "provsIndex", provsIndex, false
);
    }
    if (!_publicOnly) {
      drawPlainVariable( _panel, _g, 150, 460, 10, 0, "allowedIndex", allowedIndex,
false );
    }
    if (!_publicOnly) {
      drawPlainVariable( _panel, _g, 150, 600, 10, 0, "travelDistance",
travelDistance, false );
    }
    if (!_publicOnly) {
      drawPlainVariable( _panel, _g, 150, 630, 10, 0, "distanceFurther",
distanceFurther, false );
    }
    if (!_publicOnly) {
      drawPlainVariable( _panel, _g, 300, 400, 10, 0, "costPaid", costPaid, false );
    }
    if (!_publicOnly) {
      drawPlainVariable( _panel, _g, 150, 50, 10, 0, "destHospital", destHospital,
false );
    }
    if (!_publicOnly) {
      drawPlainVariable( _panel, _g, 150, 100, 10, 0, "destType", destType, false );
    }
    if (!_publicOnly) {
      drawPlainVariable( _panel, _g, 150, 250, 10, 0, "segment", segment, false );
    }
    if (!_publicOnly) {
      drawPlainVariable( _panel, _g, 150, 350, 10, 0, "uDiff", uDiff, false );
    }
    if (!_publicOnly) {
      drawPlainVariable( _panel, _g, 150, 400, 10, 0, "optHospital", optHospital,
false );
    }
    if (!_publicOnly) {
      drawPlainVariable( _panel, _g, 150, 660, 10, 0, "selectedBobs", selectedBobs,
false );
    }
    if (!_publicOnly) {
      drawPlainVariable( _panel, _g, 150, 700, 10, 0, "deadBaby", deadBaby, false );
    }
    if (!_publicOnly) {
      drawPlainVariable( _panel, _g, 300, 450, 10, 0, "csPerformed", csPerformed,
false );
    }
    if (!_publicOnly) {
      drawPlainVariable( _panel, _g, 300, 500, 10, 0, "catExp", catExp, false );
    }
  }

  @AnyLogicInternalCodegenAPI
  private void drawModelElements_CollectionVariables_xjal(Panel _panel, Graphics2D _g,
boolean _publicOnly, boolean _isSuperClass ) {
    if (!_publicOnly) {
      drawCollection( _panel, _g, 150, 500, 10, 0, "provs", provs );
    }
    if (!_publicOnly) {
      drawCollection( _panel, _g, 150, 530, 10, 0, "allowed", allowed );
    }
    if (!_publicOnly) {
      drawCollection( _panel, _g, 150, 560, 10, 0, "allHospitals", allHospitals );
    }
```

```
  }

  @AnyLogicInternalCodegenAPI
  private void drawModelElements_AgentLinks_xjal(Panel _panel, Graphics2D _g, boolean
_publicOnly, boolean _isSuperClass ) {
    if (_publicOnly) { return; }
      drawLinkToContainer( _panel, _g, 50, -100, 10, 0, "main", main );
      drawLinkToAgent( _panel, _g, 50, -50, 15, 0, "connections", true, connections );
  }

  @Override
  @AnyLogicInternalCodegenAPI
  public void drawModelElements( Panel _panel, Graphics2D _g, boolean _publicOnly,
boolean _isSuperClass ) {
       super.drawModelElements( _panel, _g, _publicOnly, true );
    drawModelElements_Statecharts_xjal( _panel, _g, _publicOnly, _isSuperClass );
    drawModelElements_Parameters_xjal( _panel, _g, _publicOnly, _isSuperClass );
    drawModelElements_PlainVariables_xjal( _panel, _g, _publicOnly, _isSuperClass );
    drawModelElements_CollectionVariables_xjal( _panel, _g, _publicOnly, _isSuperClass
);
    drawModelElements_AgentLinks_xjal( _panel, _g, _publicOnly, _isSuperClass );
  }

  @AnyLogicInternalCodegenAPI
  private boolean onClickModelAt_AgentLinks_xjal( Panel _panel, double _x, double _y,
int _clickCount, boolean _publicOnly, boolean _isSuperClass ) {
    if ( modelElementContains(_x, _y, 50, -100) ) {
      if ( _clickCount == 2 ) {
        _panel.browseAgent_xjal( 50, -100, this, "main" );
      } else {
        _panel.addInspect( 50, -100, this, "main" );
      }
      return true;
    }
    if ( modelElementContains(_x, _y, 50, -50) ) {
        _panel.addInspect_xjal( 50, -50, this, "connections",
Panel.INSPECT_CONNECTIONS_xjal );
      return true;
    }
    return false;
  }


  @AnyLogicInternalCodegenAPI
  private boolean onClickModelAt_Parameters_xjal( Panel _panel, double _x, double _y,
int _clickCount, boolean _publicOnly, boolean _isSuperClass ) {
    if( !_publicOnly && modelElementContains(_x, _y, 50, 50) ) {
      _panel.addInspect( 50, 50, this, "longitude" );
      return true;
    }
    if( !_publicOnly && modelElementContains(_x, _y, 50, 100) ) {
      _panel.addInspect( 50, 100, this, "latitude" );
      return true;
    }
    if( !_publicOnly && modelElementContains(_x, _y, 50, 150) ) {
      _panel.addInspect( 50, 150, this, "wealth" );
      return true;
    }
    if( !_publicOnly && modelElementContains(_x, _y, 50, 500) ) {
      _panel.addInspect( 50, 500, this, "educSec" );
      return true;
    }
    if( !_publicOnly && modelElementContains(_x, _y, 50, 400) ) {
```

```
      _panel.addInspect( 50, 400, this, "age" );
      return true;
    }
    if( !_publicOnly && modelElementContains(_x, _y, 50, 650) ) {
      _panel.addInspect( 50, 650, this, "ANC4" );
      return true;
    }
    if( !_publicOnly && modelElementContains(_x, _y, 50, 350) ) {
      _panel.addInspect( 50, 350, this, "urban" );
      return true;
    }
    if( !_publicOnly && modelElementContains(_x, _y, 50, 550) ) {
      _panel.addInspect( 50, 550, this, "twins" );
      return true;
    }
    if( !_publicOnly && modelElementContains(_x, _y, 50, 600) ) {
      _panel.addInspect( 50, 600, this, "unwanted" );
      return true;
    }
    if( !_publicOnly && modelElementContains(_x, _y, 50, 200) ) {
      _panel.addInspect( 50, 200, this, "primip" );
      return true;
    }
    if( !_publicOnly && modelElementContains(_x, _y, 50, 250) ) {
      _panel.addInspect( 50, 250, this, "blind" );
      return true;
    }
    if( !_publicOnly && modelElementContains(_x, _y, 50, 300) ) {
      _panel.addInspect( 50, 300, this, "illiterate" );
      return true;
    }
    if( !_publicOnly && modelElementContains(_x, _y, 50, 450) ) {
      _panel.addInspect( 50, 450, this, "spouseAge" );
      return true;
    }
    if( !_publicOnly && modelElementContains(_x, _y, 50, 700) ) {
      _panel.addInspect( 50, 700, this, "risk" );
      return true;
    }
    if( !_publicOnly && modelElementContains(_x, _y, 50, 750) ) {
      _panel.addInspect( 50, 750, this, "csPlanned" );
      return true;
    }
    if( !_publicOnly && modelElementContains(_x, _y, 50, 800) ) {
      _panel.addInspect( 50, 800, this, "income" );
      return true;
    }
    return false;
  }

  @AnyLogicInternalCodegenAPI
  private boolean onClickModelAt_PlainVariables_xjal( Panel _panel, double _x, double
_y, int _clickCount, boolean _publicOnly, boolean _isSuperClass ) {
    if( !_publicOnly && modelElementContains(_x, _y, 150, 150) ) {
      _panel.addInspect( 150, 150, this, "startTime" );
      return true;
    }
    if( !_publicOnly && modelElementContains(_x, _y, 150, 200) ) {
      _panel.addInspect( 150, 200, this, "travelTime" );
      return true;
    }
    if( !_publicOnly && modelElementContains(_x, _y, 150, 300) ) {
      _panel.addInspect( 150, 300, this, "bWealth" );
```

```
    return true;
  }
  if( !_publicOnly && modelElementContains(_x, _y, 150, 430) ) {
    _panel.addInspect( 150, 430, this, "provsIndex" );
    return true;
  }
  if( !_publicOnly && modelElementContains(_x, _y, 150, 460) ) {
    _panel.addInspect( 150, 460, this, "allowedIndex" );
    return true;
  }
  if( !_publicOnly && modelElementContains(_x, _y, 150, 600) ) {
    _panel.addInspect( 150, 600, this, "travelDistance" );
    return true;
  }
  if( !_publicOnly && modelElementContains(_x, _y, 150, 630) ) {
    _panel.addInspect( 150, 630, this, "distanceFurther" );
    return true;
  }
  if( !_publicOnly && modelElementContains(_x, _y, 300, 400) ) {
    _panel.addInspect( 300, 400, this, "costPaid" );
    return true;
  }
  if( !_publicOnly && modelElementContains(_x, _y, 150, 50) ) {
    _panel.addInspect( 150, 50, this, "destHospital" );
    return true;
  }
  if( !_publicOnly && modelElementContains(_x, _y, 150, 100) ) {
    _panel.addInspect( 150, 100, this, "destType" );
    return true;
  }
  if( !_publicOnly && modelElementContains(_x, _y, 150, 250) ) {
    _panel.addInspect( 150, 250, this, "segment" );
    return true;
  }
  if( !_publicOnly && modelElementContains(_x, _y, 150, 350) ) {
    _panel.addInspect( 150, 350, this, "uDiff" );
    return true;
  }
  if( !_publicOnly && modelElementContains(_x, _y, 150, 400) ) {
    _panel.addInspect( 150, 400, this, "optHospital" );
    return true;
  }
  if( !_publicOnly && modelElementContains(_x, _y, 150, 660) ) {
    _panel.addInspect( 150, 660, this, "selectedBobs" );
    return true;
  }
  if( !_publicOnly && modelElementContains(_x, _y, 150, 700) ) {
    _panel.addInspect( 150, 700, this, "deadBaby" );
    return true;
  }
  if( !_publicOnly && modelElementContains(_x, _y, 300, 450) ) {
    _panel.addInspect( 300, 450, this, "csPerformed" );
    return true;
  }
  if( !_publicOnly && modelElementContains(_x, _y, 300, 500) ) {
    _panel.addInspect( 300, 500, this, "catExp" );
    return true;
  }
  return false;
}

@AnyLogicInternalCodegenAPI
```

```
  private boolean onClickModelAt_CollectionVariables_xjal( Panel _panel, double _x,
double _y, int _clickCount, boolean _publicOnly, boolean _isSuperClass ) {
    if( !_publicOnly && modelElementContains(_x, _y, 150, 500) ) {
      _panel.addInspect( 150, 500, this, "provs" );
      return true;
    }
    if( !_publicOnly && modelElementContains(_x, _y, 150, 530) ) {
      _panel.addInspect( 150, 530, this, "allowed" );
      return true;
    }
    if( !_publicOnly && modelElementContains(_x, _y, 150, 560) ) {
      _panel.addInspect( 150, 560, this, "allHospitals" );
      return true;
    }
    return false;
  }

  @Override
  @AnyLogicInternalCodegenAPI
  public boolean onClickModelAt( Panel _panel, double _x, double _y, int _clickCount,
boolean _publicOnly, boolean _isSuperClass ) {
    if ( onClickModelAt_AgentLinks_xjal( _panel, _x, _y, _clickCount, _publicOnly,
_isSuperClass ) ) { return true; }
    if ( onClickModelAt_Parameters_xjal( _panel, _x, _y, _clickCount, _publicOnly,
_isSuperClass ) ) { return true; }
    if ( onClickModelAt_PlainVariables_xjal( _panel, _x, _y, _clickCount, _publicOnly,
_isSuperClass ) ) { return true; }
    if ( onClickModelAt_CollectionVariables_xjal( _panel, _x, _y, _clickCount,
_publicOnly, _isSuperClass ) ) { return true; }
    return super.onClickModelAt( _panel, _x, _y, _clickCount, _publicOnly, true );
  }



  @Override
  @AnyLogicInternalCodegenAPI
  public void onArrival() {
    super.onArrival();
    statechart.fireEvent( _ARRIVAL_message_xjal );
  }

  /**
   * Constructor
   */
  public Person( Engine engine, Agent owner, AgentList<? extends Person>
ownerPopulation ) {
    super( engine, owner, ownerPopulation );
    instantiateBaseStructureThis_xjal();
  }

  @AnyLogicInternalCodegenAPI
  public void onOwnerChanged_xjal() {
    super.onOwnerChanged_xjal();
    setupReferences_xjal();
  }

  @AnyLogicInternalCodegenAPI
  public void instantiateBaseStructure_xjal() {
    super.instantiateBaseStructure_xjal();
    instantiateBaseStructureThis_xjal();
  }

  @AnyLogicInternalCodegenAPI
```

```
  private void instantiateBaseStructureThis_xjal() {
    setupReferences_xjal();
  }

  @AnyLogicInternalCodegenAPI
  private void setupReferences_xjal() {
    main = get_Main();
  }

  /**
   * Simple constructor. Please add created agent to some population by calling
goToPopulation() function
   */
  public Person() {
  }

  /**
   * Simple constructor. Please add created agent to some population by calling
goToPopulation() function
   */
  public Person( double longitude, double latitude, int wealth, int educSec, int age,
int ANC4, int urban, int twins, int unwanted, int primip, int blind, int illiterate,
int spouseAge, int risk, int csPlanned, double income ) {
    markParametersAreSet();
    this.longitude = longitude;
    this.latitude = latitude;
    this.wealth = wealth;
    this.educSec = educSec;
    this.age = age;
    this.ANC4 = ANC4;
    this.urban = urban;
    this.twins = twins;
    this.unwanted = unwanted;
    this.primip = primip;
    this.blind = blind;
    this.illiterate = illiterate;
    this.spouseAge = spouseAge;
    this.risk = risk;
    this.csPlanned = csPlanned;
    this.income = income;
  }

  @Override
  @AnyLogicInternalCodegenAPI
  public void doCreate() {
    super.doCreate();
    // Assigning initial values for plain variables
    setupPlainVariables_Person_xjal();
    // Dynamic initialization of persistent elements
    _createPersistentElementsAP0_xjal();
    presentation = new ShapeTopLevelPresentationGroup( Person.this, true, 0, 0, 0, 0 ,
circle );
    icon = new ShapeModelElementsGroup( Person.this, getElementProperty(
"malawi_recentralization_model_2017_06_04.Person.icon",
IElementDescriptor.MODEL_ELEMENT_DESCRIPTORS )  );
    icon.setIconOffsets( 0.0, 0.0 );
        // Port connectors with non-replicated objects
    // Creating replicated embedded objects
    setupInitialConditions_xjal( Person.class );
  }

  @AnyLogicInternalCodegenAPI
  public void setupExt_xjal(AgentExtension _ext) {
```

```
    // Agent properties setup
    if ( _ext instanceof ExtAgentWithSpatialMetrics && _ext instanceof
ExtWithSpaceType ) {
      double _value;
      _value =
30
;
      ((ExtAgentWithSpatialMetrics) _ext).setSpeed( _value, KPH );
    }
  }

  @Override
  @AnyLogicInternalCodegenAPI
  public void doStart() {
    super.doStart();
    statechart.start();
  }


  /**
   * Assigning initial values for plain variables<br>
   * <em>This method isn't designed to be called by user and may be removed in future
releases.</em>
   */
  @AnyLogicInternalCodegenAPI
  public void setupPlainVariables_xjal() {
    setupPlainVariables_Person_xjal();
  }

  /**
   * Assigning initial values for plain variables<br>
   * <em>This method isn't designed to be called by user and may be removed in future
releases.</em>
   */
  @AnyLogicInternalCodegenAPI
  private void setupPlainVariables_Person_xjal() {
    destHospital =
null
;
    destType =
""
;
    segment =
0
;
    uDiff =
0.0
;
    optHospital =
null
;
    selectedBobs =
0.0
;
    deadBaby =
0
;
    csPerformed =
false
;
    catExp =
0
;
```

```
  }

  // User API -----------------------------------------------------
  public Main get_Main() {
    {
      Agent owner = getOwner();
      if ( owner instanceof Main ) return (Main) owner;
    }
    return null;
  }

  /**
   * Read-only variable. <em>Shouldn't be modified by user.</em>
   */
  @AnyLogicCustomSerialization(AnyLogicCustomSerializationMode.REFERENCE)
  public transient malawi_recentralization_model_2017_06_04.Main main;

  @AnyLogicInternalCodegenAPI
  static LinkToAgentAnimationSettings _connections_commonAnimationSettings_xjal = new
LinkToAgentAnimationSettingsImpl( false, black, 1.0, LINE_STYLE_SOLID, ARROW_NONE, 0.0
);

  public LinkToAgentCollection<Agent, Agent> connections = new
LinkToAgentStandardImpl<Agent, Agent>(this,
_connections_commonAnimationSettings_xjal);
  @Override
  public LinkToAgentCollection<? extends Agent, ? extends Agent>
getLinkToAgentStandard_xjal() {
    return connections;
  }
  @Override
  @AnyLogicInternalCodegenAPI
  public void onReceive( Object _msg_xjal, Agent _sender_xjal ) {
    super.onReceive( _msg_xjal, _sender_xjal );
    statechart.fireEvent( _msg_xjal );
  }



  @AnyLogicInternalCodegenAPI
  public void drawLinksToAgents(boolean _underAgents_xjal, LinkToAgentAnimator
_animator_xjal) {
    super.drawLinksToAgents(_underAgents_xjal, _animator_xjal);
    if ( _underAgents_xjal ) {
      _animator_xjal.drawLink( this, connections, true, true );
    }
  }

  public AgentList<? extends Person> getPopulation() {
    return (AgentList<? extends Person>) super.getPopulation();
  }

  public List<? extends Person> agentsInRange( double distance ) {
    return (List<? extends Person>) super.agentsInRange( distance );
  }

  // Reaction on changes -------------------------------------
  public void onChange() {
    super.onChange();
    statechart.onChange();
  }

  @AnyLogicInternalCodegenAPI
```

```java
  public void onDestroy() {
    statechart.onDestroy();
    super.onDestroy();
  }



}



package malawi_recentralization_model_2017_06_04;

import malawi_recentralization_model_2017_06_04.tables_al.*;
import com.anylogic.engine.*;
import com.anylogic.engine.database.*;
import com.anylogic.engine.connectivity.*;

import java.util.Arrays;
import java.io.File;
import java.sql.Connection;

/**
 * Descriptors for built-in database tables
 */
@AnyLogicInternalCodegenAPI
public class DBDescriptor implements DBInfo, TableDataImporter, TableDataExporter {

  /**
   * Descriptor for database table 'mortality'.<br>
   * To be used in functions like
   * {@linkplain Utilities#selectFrom(com.mysema.query.types.Expression) selectFrom()}
   */
  public static final Qmortality mortality = Qmortality.INSTANCE;

  /**
   * Descriptor for database table 'parameters'.<br>
   * To be used in functions like
   * {@linkplain Utilities#selectFrom(com.mysema.query.types.Expression) selectFrom()}
   */
  public static final Qparameters parameters = Qparameters.INSTANCE;

  /**
   * Descriptor for database table 'hospitals'.<br>
   * To be used in functions like
   * {@linkplain Utilities#selectFrom(com.mysema.query.types.Expression) selectFrom()}
   */
  public static final Qhospitals hospitals = Qhospitals.INSTANCE;

  /**
   * Descriptor for database table 'pop_rand'.<br>
   * To be used in functions like
   * {@linkplain Utilities#selectFrom(com.mysema.query.types.Expression) selectFrom()}
   */
  public static final Qpop_rand pop_rand = Qpop_rand.INSTANCE;

  @AnyLogicInternalCodegenAPI
  public DBDescriptor() {
  }

  @Override
  public DatabaseCustomType[] getCustomTypes() {
       return new DatabaseCustomType[] {
```

```
      };
  }

  @AnyLogicInternalAPI
  public void importTableDataOnStartup(java.sql.Connection internalDatabaseConnection)
throws Exception {
    try (DatabaseDescriptorRegistry r = new DatabaseDescriptorRegistry()) {
      java.sql.Connection cachedSourceConnection;
      }
  }

  @AnyLogicInternalAPI
  public void exportTableDataOnFinish(java.sql.Connection internalDatabaseConnection)
throws Exception {
  }
}
```

```
package malawi_recentralization_model_2017_06_04;

import java.io.Serializable;
import java.sql.Connection;
import java.sql.SQLException;
import java.util.ArrayDeque;
import java.util.ArrayList;
import java.util.Arrays;
import java.util.Calendar;
import java.util.Collection;
import java.util.Collections;
import java.util.Comparator;
import java.util.Currency;
import java.util.Date;
import java.util.Enumeration;
import java.util.HashMap;
import java.util.HashSet;
import java.util.Hashtable;
import java.util.Iterator;
import java.util.LinkedHashMap;
import java.util.LinkedHashSet;
import java.util.LinkedList;
import java.util.List;
import java.util.ListIterator;
import java.util.Locale;
import java.util.Map;
import java.util.PriorityQueue;
import java.util.Random;
import java.util.Set;
import java.util.SortedMap;
import java.util.SortedSet;
import java.util.Stack;
import java.util.Timer;
import java.util.TreeMap;
import java.util.TreeSet;
import java.util.Vector;
import java.awt.Color;
import java.awt.Font;
import java.awt.Graphics2D;
import java.awt.geom.AffineTransform;
import com.anylogic.engine.connectivity.ResultSet;
import com.anylogic.engine.connectivity.Statement;
import com.anylogic.engine.elements.*;
import com.anylogic.engine.markup.Network;
```

```
import com.anylogic.engine.Position;
import com.anylogic.engine.markup.PedFlowStatistics;
import com.anylogic.engine.markup.DensityMap;


import static java.lang.Math.*;
import static com.anylogic.engine.UtilitiesArray.*;
import static com.anylogic.engine.UtilitiesCollection.*;
import static com.anylogic.engine.presentation.UtilitiesColor.*;
import static com.anylogic.engine.HyperArray.*;

import com.anylogic.engine.*;
import com.anylogic.engine.analysis.*;
import com.anylogic.engine.connectivity.*;
import com.anylogic.engine.database.*;
import com.anylogic.engine.gis.*;
import com.anylogic.engine.markup.*;
import com.anylogic.engine.presentation.*;

import com.mysema.query.Tuple;
import com.mysema.query.sql.SQLBindings;
import static malawi_recentralization_model_2017_06_04.DBDescriptor.*;

import javax.swing.JApplet;



public class SimulationNewStrategies extends ExperimentSimulation<Main> {
  @AnyLogicInternalCodegenAPI
  public static String[] COMMAND_LINE_ARGUMENTS_xjal = new String[0];
  {
    setCommandLineArguments_xjal( COMMAND_LINE_ARGUMENTS_xjal );
  }
  @AnyLogicInternalCodegenAPI
  private static Map<String, IElementDescriptor> elementDesciptors_xjal =
createElementDescriptors( SimulationNewStrategies.class );

  @AnyLogicInternalCodegenAPI
  @Override
  public Map<String, IElementDescriptor> getElementDesciptors() {
    return elementDesciptors_xjal;
  }
  // View areas
  @AnyLogicInternalCodegenAPI
  protected static final Font _button_Font = new Font("Dialog", 0, 11 );
  @AnyLogicInternalCodegenAPI
  protected static final Font _allowFees_Font = new Font("arial", 0, 12 );
  @AnyLogicInternalCodegenAPI
  protected static final Font _nonHospitals_Font = new Font("Arial", 0, 12 );
  @AnyLogicInternalCodegenAPI
  protected static final Font _strategies_Font = _button_Font;
  @AnyLogicInternalCodegenAPI
  protected static final Font _text_Font = new Font("SansSerif", 0, 24 );
  @AnyLogicInternalCodegenAPI
  protected static final Font _allowList_Font = new Font("Times New Roman", 0, 18 );
  @AnyLogicInternalCodegenAPI
  protected static final Font _QIText_Font = _allowList_Font;
  @AnyLogicInternalCodegenAPI
  protected static final Font _QIText2_Font = _nonHospitals_Font;
  @AnyLogicInternalCodegenAPI
  protected static final Font _text1_Font = new Font("SansSerif", 0, 10 );
  @AnyLogicInternalCodegenAPI
  protected static final Font _text2_Font = _text1_Font;
```

```
  @AnyLogicInternalCodegenAPI
  protected static final Font _text3_Font = _text1_Font;
  @AnyLogicInternalCodegenAPI
  protected static final Font _allowList1_Font = _allowList_Font;
  @AnyLogicInternalCodegenAPI
  protected static final int _button = 1;
  @AnyLogicInternalCodegenAPI
  protected static final int _allowFees = 2;
  @AnyLogicInternalCodegenAPI
  protected static final int _nonHospitals = 3;
  @AnyLogicInternalCodegenAPI
  protected static final int _QIscore = 4;
  @AnyLogicInternalCodegenAPI
  protected static final int _strategies = 5;
  @AnyLogicInternalCodegenAPI
  protected static final int _text = 6;
  @AnyLogicInternalCodegenAPI
  protected static final int _allowList = 7;
  @AnyLogicInternalCodegenAPI
  protected static final int _QIText = 8;
  @AnyLogicInternalCodegenAPI
  protected static final int _QIText2 = 9;
  @AnyLogicInternalCodegenAPI
  protected static final int _text1 = 10;
  @AnyLogicInternalCodegenAPI
  protected static final int _text2 = 11;
  @AnyLogicInternalCodegenAPI
  protected static final int _text3 = 12;
  @AnyLogicInternalCodegenAPI
  protected static final int _allowList1 = 13;

  /** Internal constant, shouldn't be accessed by user */
  @AnyLogicInternalCodegenAPI
  protected static final int _SHAPE_NEXT_ID_xjal = 14;


  /**
   * Top-level presentation group id
   */
  @AnyLogicInternalCodegenAPI
  protected static final int _presentation = 0;

  /**
   * Top-level icon group id
   */
  @AnyLogicInternalCodegenAPI
  protected static final int _icon = -1;


  @Override
  @AnyLogicInternalCodegenAPI
  public void executeShapeControlAction( int _shape, int index ) {
    switch( _shape ) {
      case _button: {
          ShapeButton self = this.button;
if ( getState() == IDLE )
       run();
getPresentation().setPresentable( getEngine().getRoot() );
;}
        break;
      default:
        super.executeShapeControlAction( _shape, index );
        break;
```

```
    }
  }

  @Override
  @AnyLogicInternalCodegenAPI
  public double getShapeControlMinimum( int _shape, int index ) {
    switch( _shape ) {
      case _QIscore: return
0
;
      default: return super.getShapeControlMinimum( _shape, index );
    }
  }

  @Override
  @AnyLogicInternalCodegenAPI
  public double getShapeControlMaximum( int _shape, int index ) {
    switch( _shape ) {
      case _QIscore: return
1
;
      default: return super.getShapeControlMaximum( _shape, index );
    }
  }

  @Override
  @AnyLogicInternalCodegenAPI
  public boolean getShapeControlDefaultValueBoolean( int _shape, int index ) {
    switch(_shape) {
      case _allowFees: return
true
;
      case _nonHospitals: return
true
;
      default: return super.getShapeControlDefaultValueBoolean( _shape, index );
    }
  }

  @Override
  @AnyLogicInternalCodegenAPI
  public double getShapeControlDefaultValueDouble( int _shape, int index ) {
    switch(_shape) {
      case _QIscore: return
0
;
      default: return super.getShapeControlDefaultValueDouble( _shape, index );
    }
  }


  /**
   * <i>This method should not be called by user</i>
   */
  @AnyLogicInternalCodegenAPI
  private void _button_SetDynamicParams_xjal( ShapeButton shape ) {
    shape.setText(
getState() == IDLE ?
       "Run" :
       "Top level agent"
);
  }
```

```
  protected ShapeButton button;
  protected ShapeCheckBox allowFees;
  protected ShapeCheckBox nonHospitals;

  /**
   * <i>This method should not be called by user</i>
   */
  @AnyLogicInternalCodegenAPI
  private void _QIscore_SetDynamicParams_xjal( ShapeSlider shape ) {
    shape.setRange( getShapeControlMinimum( _QIscore ), getShapeControlMaximum(
_QIscore ) );
  }

  protected ShapeSlider QIscore;
  protected ShapeRadioButtonGroup strategies;
  protected ShapeText text;
  protected ShapeText allowList;
  protected ShapeText QIText;
  protected ShapeText QIText2;

  /**
   * <i>This method should not be called by user</i>
   */
  @AnyLogicInternalCodegenAPI
  private void _text1_SetDynamicParams_xjal( ShapeText shape ) {
    shape.setText(
QIscore.getMin()
);
  }

  protected ShapeText text1;

  /**
   * <i>This method should not be called by user</i>
   */
  @AnyLogicInternalCodegenAPI
  private void _text2_SetDynamicParams_xjal( ShapeText shape ) {
    shape.setText(
QIscore.getMax()
);
  }

  protected ShapeText text2;

  /**
   * <i>This method should not be called by user</i>
   */
  @AnyLogicInternalCodegenAPI
  private void _text3_SetDynamicParams_xjal( ShapeText shape ) {
    shape.setText(
QIscore.getValue()
);
  }

  protected ShapeText text3;
  protected ShapeText allowList1;
  @AnyLogicInternalCodegenAPI
  private void _createPersistentElementsBP0_xjal() {
    button = new ShapeButton(
                SimulationNewStrategies.this, true, 60.0, 380.0,
                100.0, 30.0,
        controlDefault, controlDefault, true,
        _button_Font,
```

```
                    "Run" ) {
      @Override
      public void updateDynamicProperties(boolean publicOnly) {
        _button_SetDynamicParams_xjal( this );
        super.updateDynamicProperties(publicOnly);
      }

      @Override
      @AnyLogicInternalCodegenAPI
      public void action(){
        executeShapeControlAction( _button, 0 );
      }
    };

    allowFees = new ShapeCheckBox(
              SimulationNewStrategies.this,true,260.0, 150.0,
              140.0, 30.0,
            transparent, controlDefault, true,
            _allowFees_Font,
                    "Allow fees" ) {

      @Override
      public void setValueToDefault() {
              setSelected( getShapeControlDefaultValueBoolean( _allowFees, 0 ) );
      }
    };

    nonHospitals = new ShapeCheckBox(
              SimulationNewStrategies.this,true,260.0, 120.0,
              190.0, 30.0,
            transparent, controlDefault, true,
            _nonHospitals_Font,
                    "Allow non-hospital facilities" ) {

      @Override
      public void setValueToDefault() {
              setSelected( getShapeControlDefaultValueBoolean( _nonHospitals, 0 ) );
      }
    };

    QIscore = new ShapeSlider(
                SimulationNewStrategies.this, true, 270.0, 290.0,
                  220.0, 30.0,
            transparent, true,
            false, getShapeControlMinimum( _QIscore ), getShapeControlMaximum(
_QIscore ), ShapeControl.TYPE_DOUBLE ) {
      @Override
      public void updateDynamicProperties(boolean publicOnly) {
        _QIscore_SetDynamicParams_xjal( this );
        super.updateDynamicProperties(publicOnly);
      }

      @Override
      public void setValueToDefault() {
              setValue( limit( getMin(), getShapeControlDefaultValueDouble( _QIscore, 0
), getMax() ) );
      }
    };

    strategies = new ShapeRadioButtonGroup(
                SimulationNewStrategies.this, true, 50.0, 110.0,
                  150.0, 150.0,
            transparent, controlDefault, true,
```

```
            _strategies_Font, true,
            new String[]{"Status quo", "Only CS-capable", "Upgrade CS", "Only BEMC",
"Upgrade BEMC", } );

    text = new ShapeText(
        SHAPE_DRAW_2D, true,40.0, 30.0, 0.0, 0.0,
        royalBlue,"Malawi Recentralization Model (2018-05-11 update)",
        _text_Font, ALIGNMENT_LEFT );

    allowList = new ShapeText(
        SHAPE_DRAW_2D, true,50.0, 80.0, 0.0, 0.0,
        black,"Strategies",
        _allowList_Font, ALIGNMENT_LEFT );

    QIText = new ShapeText(
        SHAPE_DRAW_2D, true,260.0, 190.0, 0.0, 0.0,
        black,"Quality improvement",
        _QIText_Font, ALIGNMENT_LEFT );

    QIText2 = new ShapeText(
        SHAPE_DRAW_2D, true,280.0, 210.0, 0.0, 0.0,
        black,"(This value will be added to the \nbasic obstetric readiness score
of\neach hospital when delivery is \nrestricted to hospitals only):",
        _QIText2_Font, ALIGNMENT_LEFT );

    text1 = new ShapeText(
        SHAPE_DRAW_2D, true,270.0, 320.0, 0.0, 0.0,
        black,"min",
        _text1_Font, ALIGNMENT_LEFT ) {
      @Override
      public void updateDynamicProperties(boolean publicOnly) {
        _text1_SetDynamicParams_xjal( this );
        super.updateDynamicProperties(publicOnly);
      }
    };

    text2 = new ShapeText(
        SHAPE_DRAW_2D, true,490.0, 320.0, 0.0, 0.0,
        black,"max",
        _text2_Font, ALIGNMENT_RIGHT ) {
      @Override
      public void updateDynamicProperties(boolean publicOnly) {
        _text2_SetDynamicParams_xjal( this );
        super.updateDynamicProperties(publicOnly);
      }
    };

    text3 = new ShapeText(
        SHAPE_DRAW_2D, true,380.0, 320.0, 0.0, 0.0,
        black,"value",
        _text3_Font, ALIGNMENT_CENTER ) {
      @Override
      public void updateDynamicProperties(boolean publicOnly) {
        _text3_SetDynamicParams_xjal( this );
        super.updateDynamicProperties(publicOnly);
      }
    };

    allowList1 = new ShapeText(
        SHAPE_DRAW_2D, true,260.0, 80.0, 0.0, 0.0,
        black,"Old strategies \n(prior to MEK's revamp of the question)",
        _allowList1_Font, ALIGNMENT_LEFT );
```

```
  }

  @AnyLogicInternalCodegenAPI
  private void _createPersistentElementsAP0_xjal() {
  }


  protected ShapeTopLevelPresentationGroup presentation;
  protected ShapeModelElementsGroup icon;

  @Override
  @AnyLogicInternalCodegenAPI
  public ShapeTopLevelPresentationGroup getPresentationShape() {
    return presentation;
  }

  @Override
  @AnyLogicInternalCodegenAPI
  public ShapeModelElementsGroup getModelElementsShape() {
    return icon;
  }

  @Override
  @AnyLogicInternalCodegenAPI
  public Object getPersistentShape( int _shape ) {
    switch (_shape) {
      case _presentation: return presentation;
      case _icon: return icon;
      case _button: return button;
      case _allowFees: return allowFees;
      case _nonHospitals: return nonHospitals;
      case _QIscore: return QIscore;
      case _strategies: return strategies;
      case _text: return text;
      case _allowList: return allowList;
      case _QIText: return QIText;
      case _QIText2: return QIText2;
      case _text1: return text1;
      case _text2: return text2;
      case _text3: return text3;
      case _allowList1: return allowList1;
      default: return super.getPersistentShape( _shape );
    }
  }

  @Override
  @AnyLogicInternalCodegenAPI
  public String getNameOfShape_xjal( Object _shape ) {
    try {
      if ( _shape == null ) return null;
      String _name_xjal;
      _name_xjal = checkNameOfShape_xjal( _shape, presentation, "presentation" ); if
(_name_xjal != null) return _name_xjal;
      _name_xjal = checkNameOfShape_xjal( _shape, icon, "icon" ); if (_name_xjal !=
null) return _name_xjal;
      _name_xjal = checkNameOfShape_xjal( _shape, button, "button" ); if (_name_xjal
!= null) return _name_xjal;
      _name_xjal = checkNameOfShape_xjal( _shape, allowFees, "allowFees" ); if
(_name_xjal != null) return _name_xjal;
      _name_xjal = checkNameOfShape_xjal( _shape, nonHospitals, "nonHospitals" ); if
(_name_xjal != null) return _name_xjal;
      _name_xjal = checkNameOfShape_xjal( _shape, QIscore, "QIscore" ); if (_name_xjal
!= null) return _name_xjal;
```

```
      _name_xjal = checkNameOfShape_xjal( _shape, strategies, "strategies" ); if
(_name_xjal != null) return _name_xjal;
      _name_xjal = checkNameOfShape_xjal( _shape, text, "text" ); if (_name_xjal !=
null) return _name_xjal;
      _name_xjal = checkNameOfShape_xjal( _shape, allowList, "allowList" ); if
(_name_xjal != null) return _name_xjal;
      _name_xjal = checkNameOfShape_xjal( _shape, QIText, "QIText" ); if (_name_xjal
!= null) return _name_xjal;
      _name_xjal = checkNameOfShape_xjal( _shape, QIText2, "QIText2" ); if (_name_xjal
!= null) return _name_xjal;
      _name_xjal = checkNameOfShape_xjal( _shape, text1, "text1" ); if (_name_xjal !=
null) return _name_xjal;
      _name_xjal = checkNameOfShape_xjal( _shape, text2, "text2" ); if (_name_xjal !=
null) return _name_xjal;
      _name_xjal = checkNameOfShape_xjal( _shape, text3, "text3" ); if (_name_xjal !=
null) return _name_xjal;
      _name_xjal = checkNameOfShape_xjal( _shape, allowList1, "allowList1" ); if
(_name_xjal != null) return _name_xjal;
    } catch (Exception e) {
      return null;
    }
    return super.getNameOfShape_xjal( _shape );
  }

  @Override
  public int getWindowWidth() {
    return 1550;
  }

  @Override
  public int getWindowHeight() {
    return 890;
  }

  @Override
  @AnyLogicInternalCodegenAPI
  public void onDestroy_xjal() {
    super.onDestroy_xjal();
  }

  /**
   * Applet class to run experiment as java applet
   */
  @AnyLogicInternalCodegenAPI
  public static class Applet extends JApplet {

  @AnyLogicInternalCodegenAPI
    SimulationNewStrategies ex;

    @Override
    public void init() {
      ex = new SimulationNewStrategies();
      ex.setup( this );
    }

    @Override
    public void destroy() {
      ex.close();
    }

  }

  @Override
```

```
  @AnyLogicInternalCodegenAPI
  public void initDefaultRandomNumberGenerator(Engine _e) {
    _e.getDefaultRandomGenerator().setSeed( 1 );
  }

  @Override
  @AnyLogicInternalCodegenAPI
  public Main createRoot( Engine engine ) {
    // Create the root object
    return new Main( engine, null, null );
  }

  @Override
  @AnyLogicInternalCodegenAPI
  public void setupRootParameters( final Main self, boolean callOnChangeActions ) {
    final Main root = self; // for compatibility
    boolean allowFeesParam_xjal;
    allowFeesParam_xjal =
allowFees.isSelected()
;
    if (callOnChangeActions) {
      self.set_allowFeesParam( allowFeesParam_xjal );
    } else {
      self.allowFeesParam = allowFeesParam_xjal;
    }
    double centralHosp1_xjal;
    centralHosp1_xjal = self._centralHosp1_DefaultValue_xjal();
    if (callOnChangeActions) {
      self.set_centralHosp1( centralHosp1_xjal );
    } else {
      self.centralHosp1 = centralHosp1_xjal;
    }
    double distance1_xjal;
    distance1_xjal = self._distance1_DefaultValue_xjal();
    if (callOnChangeActions) {
      self.set_distance1( distance1_xjal );
    } else {
      self.distance1 = distance1_xjal;
    }
    double clinic1_xjal;
    clinic1_xjal = self._clinic1_DefaultValue_xjal();
    if (callOnChangeActions) {
      self.set_clinic1( clinic1_xjal );
    } else {
      self.clinic1 = clinic1_xjal;
    }
    double maternity1_xjal;
    maternity1_xjal = self._maternity1_DefaultValue_xjal();
    if (callOnChangeActions) {
      self.set_maternity1( maternity1_xjal );
    } else {
      self.maternity1 = maternity1_xjal;
    }
    double healthCenter1_xjal;
    healthCenter1_xjal = self._healthCenter1_DefaultValue_xjal();
    if (callOnChangeActions) {
      self.set_healthCenter1( healthCenter1_xjal );
    } else {
      self.healthCenter1 = healthCenter1_xjal;
    }
    double otherHosp1_xjal;
    otherHosp1_xjal = self._otherHosp1_DefaultValue_xjal();
    if (callOnChangeActions) {
```

```
      self.set_otherHosp1( otherHosp1_xjal );
    } else {
      self.otherHosp1 = otherHosp1_xjal;
    }
    double communityHosp1_xjal;
    communityHosp1_xjal = self._communityHosp1_DefaultValue_xjal();
    if (callOnChangeActions) {
      self.set_communityHosp1( communityHosp1_xjal );
    } else {
      self.communityHosp1 = communityHosp1_xjal;
    }
    double districtHosp1_xjal;
    districtHosp1_xjal = self._districtHosp1_DefaultValue_xjal();
    if (callOnChangeActions) {
      self.set_districtHosp1( districtHosp1_xjal );
    } else {
      self.districtHosp1 = districtHosp1_xjal;
    }
    double bobs1_xjal;
    bobs1_xjal = self._bobs1_DefaultValue_xjal();
    if (callOnChangeActions) {
      self.set_bobs1( bobs1_xjal );
    } else {
      self.bobs1 = bobs1_xjal;
    }
    double fees1_xjal;
    fees1_xjal = self._fees1_DefaultValue_xjal();
    if (callOnChangeActions) {
      self.set_fees1( fees1_xjal );
    } else {
      self.fees1 = fees1_xjal;
    }
    boolean allowAllParam_xjal;
    allowAllParam_xjal =
nonHospitals.isSelected()
;
    if (callOnChangeActions) {
      self.set_allowAllParam( allowAllParam_xjal );
    } else {
      self.allowAllParam = allowAllParam_xjal;
    }
    double intercept_xjal;
    intercept_xjal = self._intercept_DefaultValue_xjal();
    if (callOnChangeActions) {
      self.set_intercept( intercept_xjal );
    } else {
      self.intercept = intercept_xjal;
    }
    double centralHosp2_xjal;
    centralHosp2_xjal = self._centralHosp2_DefaultValue_xjal();
    if (callOnChangeActions) {
      self.set_centralHosp2( centralHosp2_xjal );
    } else {
      self.centralHosp2 = centralHosp2_xjal;
    }
    double distance2_xjal;
    distance2_xjal = self._distance2_DefaultValue_xjal();
    if (callOnChangeActions) {
      self.set_distance2( distance2_xjal );
    } else {
      self.distance2 = distance2_xjal;
    }
    double clinic2_xjal;
```

```
clinic2_xjal = self._clinic2_DefaultValue_xjal();
if (callOnChangeActions) {
  self.set_clinic2( clinic2_xjal );
} else {
  self.clinic2 = clinic2_xjal;
}
double maternity2_xjal;
maternity2_xjal = self._maternity2_DefaultValue_xjal();
if (callOnChangeActions) {
  self.set_maternity2( maternity2_xjal );
} else {
  self.maternity2 = maternity2_xjal;
}
double healthCenter2_xjal;
healthCenter2_xjal = self._healthCenter2_DefaultValue_xjal();
if (callOnChangeActions) {
  self.set_healthCenter2( healthCenter2_xjal );
} else {
  self.healthCenter2 = healthCenter2_xjal;
}
double otherHosp2_xjal;
otherHosp2_xjal = self._otherHosp2_DefaultValue_xjal();
if (callOnChangeActions) {
  self.set_otherHosp2( otherHosp2_xjal );
} else {
  self.otherHosp2 = otherHosp2_xjal;
}
double communityHosp2_xjal;
communityHosp2_xjal = self._communityHosp2_DefaultValue_xjal();
if (callOnChangeActions) {
  self.set_communityHosp2( communityHosp2_xjal );
} else {
  self.communityHosp2 = communityHosp2_xjal;
}
double districtHosp2_xjal;
districtHosp2_xjal = self._districtHosp2_DefaultValue_xjal();
if (callOnChangeActions) {
  self.set_districtHosp2( districtHosp2_xjal );
} else {
  self.districtHosp2 = districtHosp2_xjal;
}
double bobs2_xjal;
bobs2_xjal = self._bobs2_DefaultValue_xjal();
if (callOnChangeActions) {
  self.set_bobs2( bobs2_xjal );
} else {
  self.bobs2 = bobs2_xjal;
}
double fees2_xjal;
fees2_xjal = self._fees2_DefaultValue_xjal();
if (callOnChangeActions) {
  self.set_fees2( fees2_xjal );
} else {
  self.fees2 = fees2_xjal;
}
double distCenter_xjal;
distCenter_xjal = self._distCenter_DefaultValue_xjal();
if (callOnChangeActions) {
  self.set_distCenter( distCenter_xjal );
} else {
  self.distCenter = distCenter_xjal;
}
double distRMS_xjal;
```

```
distRMS_xjal = self._distRMS_DefaultValue_xjal();
if (callOnChangeActions) {
  self.set_distRMS( distRMS_xjal );
} else {
  self.distRMS = distRMS_xjal;
}
double centralHosp1SE_xjal;
centralHosp1SE_xjal = self._centralHosp1SE_DefaultValue_xjal();
if (callOnChangeActions) {
  self.set_centralHosp1SE( centralHosp1SE_xjal );
} else {
  self.centralHosp1SE = centralHosp1SE_xjal;
}
double distance1SE_xjal;
distance1SE_xjal = self._distance1SE_DefaultValue_xjal();
if (callOnChangeActions) {
  self.set_distance1SE( distance1SE_xjal );
} else {
  self.distance1SE = distance1SE_xjal;
}
double clinic1SE_xjal;
clinic1SE_xjal = self._clinic1SE_DefaultValue_xjal();
if (callOnChangeActions) {
  self.set_clinic1SE( clinic1SE_xjal );
} else {
  self.clinic1SE = clinic1SE_xjal;
}
double maternity1SE_xjal;
maternity1SE_xjal = self._maternity1SE_DefaultValue_xjal();
if (callOnChangeActions) {
  self.set_maternity1SE( maternity1SE_xjal );
} else {
  self.maternity1SE = maternity1SE_xjal;
}
double healthCenter1SE_xjal;
healthCenter1SE_xjal = self._healthCenter1SE_DefaultValue_xjal();
if (callOnChangeActions) {
  self.set_healthCenter1SE( healthCenter1SE_xjal );
} else {
  self.healthCenter1SE = healthCenter1SE_xjal;
}
double otherHosp1SE_xjal;
otherHosp1SE_xjal = self._otherHosp1SE_DefaultValue_xjal();
if (callOnChangeActions) {
  self.set_otherHosp1SE( otherHosp1SE_xjal );
} else {
  self.otherHosp1SE = otherHosp1SE_xjal;
}
double communityHosp1SE_xjal;
communityHosp1SE_xjal = self._communityHosp1SE_DefaultValue_xjal();
if (callOnChangeActions) {
  self.set_communityHosp1SE( communityHosp1SE_xjal );
} else {
  self.communityHosp1SE = communityHosp1SE_xjal;
}
double districtHosp1SE_xjal;
districtHosp1SE_xjal = self._districtHosp1SE_DefaultValue_xjal();
if (callOnChangeActions) {
  self.set_districtHosp1SE( districtHosp1SE_xjal );
} else {
  self.districtHosp1SE = districtHosp1SE_xjal;
}
double bobs1SE_xjal;
```

```
bobs1SE_xjal = self._bobs1SE_DefaultValue_xjal();
if (callOnChangeActions) {
  self.set_bobs1SE( bobs1SE_xjal );
} else {
  self.bobs1SE = bobs1SE_xjal;
}
double fees1SE_xjal;
fees1SE_xjal = self._fees1SE_DefaultValue_xjal();
if (callOnChangeActions) {
  self.set_fees1SE( fees1SE_xjal );
} else {
  self.fees1SE = fees1SE_xjal;
}
double interceptSE_xjal;
interceptSE_xjal = self._interceptSE_DefaultValue_xjal();
if (callOnChangeActions) {
  self.set_interceptSE( interceptSE_xjal );
} else {
  self.interceptSE = interceptSE_xjal;
}
double centralHosp2SE_xjal;
centralHosp2SE_xjal = self._centralHosp2SE_DefaultValue_xjal();
if (callOnChangeActions) {
  self.set_centralHosp2SE( centralHosp2SE_xjal );
} else {
  self.centralHosp2SE = centralHosp2SE_xjal;
}
double distance2SE_xjal;
distance2SE_xjal = self._distance2SE_DefaultValue_xjal();
if (callOnChangeActions) {
  self.set_distance2SE( distance2SE_xjal );
} else {
  self.distance2SE = distance2SE_xjal;
}
double clinic2SE_xjal;
clinic2SE_xjal = self._clinic2SE_DefaultValue_xjal();
if (callOnChangeActions) {
  self.set_clinic2SE( clinic2SE_xjal );
} else {
  self.clinic2SE = clinic2SE_xjal;
}
double maternity2SE_xjal;
maternity2SE_xjal = self._maternity2SE_DefaultValue_xjal();
if (callOnChangeActions) {
  self.set_maternity2SE( maternity2SE_xjal );
} else {
  self.maternity2SE = maternity2SE_xjal;
}
double healthCenter2SE_xjal;
healthCenter2SE_xjal = self._healthCenter2SE_DefaultValue_xjal();
if (callOnChangeActions) {
  self.set_healthCenter2SE( healthCenter2SE_xjal );
} else {
  self.healthCenter2SE = healthCenter2SE_xjal;
}
double otherHosp2SE_xjal;
otherHosp2SE_xjal = self._otherHosp2SE_DefaultValue_xjal();
if (callOnChangeActions) {
  self.set_otherHosp2SE( otherHosp2SE_xjal );
} else {
  self.otherHosp2SE = otherHosp2SE_xjal;
}
double communityHosp2SE_xjal;
```

```
communityHosp2SE_xjal = self._communityHosp2SE_DefaultValue_xjal();
if (callOnChangeActions) {
  self.set_communityHosp2SE( communityHosp2SE_xjal );
} else {
  self.communityHosp2SE = communityHosp2SE_xjal;
}
double districtHosp2SE_xjal;
districtHosp2SE_xjal = self._districtHosp2SE_DefaultValue_xjal();
if (callOnChangeActions) {
  self.set_districtHosp2SE( districtHosp2SE_xjal );
} else {
  self.districtHosp2SE = districtHosp2SE_xjal;
}
double bobs2SE_xjal;
bobs2SE_xjal = self._bobs2SE_DefaultValue_xjal();
if (callOnChangeActions) {
  self.set_bobs2SE( bobs2SE_xjal );
} else {
  self.bobs2SE = bobs2SE_xjal;
}
double fees2SE_xjal;
fees2SE_xjal = self._fees2SE_DefaultValue_xjal();
if (callOnChangeActions) {
  self.set_fees2SE( fees2SE_xjal );
} else {
  self.fees2SE = fees2SE_xjal;
}
double centralCS_xjal;
centralCS_xjal = self._centralCS_DefaultValue_xjal();
if (callOnChangeActions) {
  self.set_centralCS( centralCS_xjal );
} else {
  self.centralCS = centralCS_xjal;
}
double clinicCS_xjal;
clinicCS_xjal = self._clinicCS_DefaultValue_xjal();
if (callOnChangeActions) {
  self.set_clinicCS( clinicCS_xjal );
} else {
  self.clinicCS = clinicCS_xjal;
}
double maternityCS_xjal;
maternityCS_xjal = self._maternityCS_DefaultValue_xjal();
if (callOnChangeActions) {
  self.set_maternityCS( maternityCS_xjal );
} else {
  self.maternityCS = maternityCS_xjal;
}
double healthCenterCS_xjal;
healthCenterCS_xjal = self._healthCenterCS_DefaultValue_xjal();
if (callOnChangeActions) {
  self.set_healthCenterCS( healthCenterCS_xjal );
} else {
  self.healthCenterCS = healthCenterCS_xjal;
}
double otherCS_xjal;
otherCS_xjal = self._otherCS_DefaultValue_xjal();
if (callOnChangeActions) {
  self.set_otherCS( otherCS_xjal );
} else {
  self.otherCS = otherCS_xjal;
}
double communityCS_xjal;
```

```
communityCS_xjal = self._communityCS_DefaultValue_xjal();
if (callOnChangeActions) {
  self.set_communityCS( communityCS_xjal );
} else {
  self.communityCS = communityCS_xjal;
}
double districtCS_xjal;
districtCS_xjal = self._districtCS_DefaultValue_xjal();
if (callOnChangeActions) {
  self.set_districtCS( districtCS_xjal );
} else {
  self.districtCS = districtCS_xjal;
}
double centralCSSE_xjal;
centralCSSE_xjal = self._centralCSSE_DefaultValue_xjal();
if (callOnChangeActions) {
  self.set_centralCSSE( centralCSSE_xjal );
} else {
  self.centralCSSE = centralCSSE_xjal;
}
double clinicCSSE_xjal;
clinicCSSE_xjal = self._clinicCSSE_DefaultValue_xjal();
if (callOnChangeActions) {
  self.set_clinicCSSE( clinicCSSE_xjal );
} else {
  self.clinicCSSE = clinicCSSE_xjal;
}
double maternityCSSE_xjal;
maternityCSSE_xjal = self._maternityCSSE_DefaultValue_xjal();
if (callOnChangeActions) {
  self.set_maternityCSSE( maternityCSSE_xjal );
} else {
  self.maternityCSSE = maternityCSSE_xjal;
}
double healthCenterCSSE_xjal;
healthCenterCSSE_xjal = self._healthCenterCSSE_DefaultValue_xjal();
if (callOnChangeActions) {
  self.set_healthCenterCSSE( healthCenterCSSE_xjal );
} else {
  self.healthCenterCSSE = healthCenterCSSE_xjal;
}
double otherCSSE_xjal;
otherCSSE_xjal = self._otherCSSE_DefaultValue_xjal();
if (callOnChangeActions) {
  self.set_otherCSSE( otherCSSE_xjal );
} else {
  self.otherCSSE = otherCSSE_xjal;
}
double communityCSSE_xjal;
communityCSSE_xjal = self._communityCSSE_DefaultValue_xjal();
if (callOnChangeActions) {
  self.set_communityCSSE( communityCSSE_xjal );
} else {
  self.communityCSSE = communityCSSE_xjal;
}
double districtCSSE_xjal;
districtCSSE_xjal = self._districtCSSE_DefaultValue_xjal();
if (callOnChangeActions) {
  self.set_districtCSSE( districtCSSE_xjal );
} else {
  self.districtCSSE = districtCSSE_xjal;
}
double centralNo_xjal;
```

```
centralNo_xjal = self._centralNo_DefaultValue_xjal();
if (callOnChangeActions) {
  self.set_centralNo( centralNo_xjal );
} else {
  self.centralNo = centralNo_xjal;
}
double clinicNo_xjal;
clinicNo_xjal = self._clinicNo_DefaultValue_xjal();
if (callOnChangeActions) {
  self.set_clinicNo( clinicNo_xjal );
} else {
  self.clinicNo = clinicNo_xjal;
}
double maternityNo_xjal;
maternityNo_xjal = self._maternityNo_DefaultValue_xjal();
if (callOnChangeActions) {
  self.set_maternityNo( maternityNo_xjal );
} else {
  self.maternityNo = maternityNo_xjal;
}
double healthCenterNo_xjal;
healthCenterNo_xjal = self._healthCenterNo_DefaultValue_xjal();
if (callOnChangeActions) {
  self.set_healthCenterNo( healthCenterNo_xjal );
} else {
  self.healthCenterNo = healthCenterNo_xjal;
}
double otherNo_xjal;
otherNo_xjal = self._otherNo_DefaultValue_xjal();
if (callOnChangeActions) {
  self.set_otherNo( otherNo_xjal );
} else {
  self.otherNo = otherNo_xjal;
}
double communityNo_xjal;
communityNo_xjal = self._communityNo_DefaultValue_xjal();
if (callOnChangeActions) {
  self.set_communityNo( communityNo_xjal );
} else {
  self.communityNo = communityNo_xjal;
}
double districtNo_xjal;
districtNo_xjal = self._districtNo_DefaultValue_xjal();
if (callOnChangeActions) {
  self.set_districtNo( districtNo_xjal );
} else {
  self.districtNo = districtNo_xjal;
}
double centralNoSE_xjal;
centralNoSE_xjal = self._centralNoSE_DefaultValue_xjal();
if (callOnChangeActions) {
  self.set_centralNoSE( centralNoSE_xjal );
} else {
  self.centralNoSE = centralNoSE_xjal;
}
double clinicNoSE_xjal;
clinicNoSE_xjal = self._clinicNoSE_DefaultValue_xjal();
if (callOnChangeActions) {
  self.set_clinicNoSE( clinicNoSE_xjal );
} else {
  self.clinicNoSE = clinicNoSE_xjal;
}
double maternityNoSE_xjal;
```

```
maternityNoSE_xjal = self._maternityNoSE_DefaultValue_xjal();
if (callOnChangeActions) {
  self.set_maternityNoSE( maternityNoSE_xjal );
} else {
  self.maternityNoSE = maternityNoSE_xjal;
}
double healthCenterNoSE_xjal;
healthCenterNoSE_xjal = self._healthCenterNoSE_DefaultValue_xjal();
if (callOnChangeActions) {
  self.set_healthCenterNoSE( healthCenterNoSE_xjal );
} else {
  self.healthCenterNoSE = healthCenterNoSE_xjal;
}
double otherNoSE_xjal;
otherNoSE_xjal = self._otherNoSE_DefaultValue_xjal();
if (callOnChangeActions) {
  self.set_otherNoSE( otherNoSE_xjal );
} else {
  self.otherNoSE = otherNoSE_xjal;
}
double communityNoSE_xjal;
communityNoSE_xjal = self._communityNoSE_DefaultValue_xjal();
if (callOnChangeActions) {
  self.set_communityNoSE( communityNoSE_xjal );
} else {
  self.communityNoSE = communityNoSE_xjal;
}
double districtNoSE_xjal;
districtNoSE_xjal = self._districtNoSE_DefaultValue_xjal();
if (callOnChangeActions) {
  self.set_districtNoSE( districtNoSE_xjal );
} else {
  self.districtNoSE = districtNoSE_xjal;
}
double central5_xjal;
central5_xjal = self._central5_DefaultValue_xjal();
if (callOnChangeActions) {
  self.set_central5( central5_xjal );
} else {
  self.central5 = central5_xjal;
}
double clinic5_xjal;
clinic5_xjal = self._clinic5_DefaultValue_xjal();
if (callOnChangeActions) {
  self.set_clinic5( clinic5_xjal );
} else {
  self.clinic5 = clinic5_xjal;
}
double maternity5_xjal;
maternity5_xjal = self._maternity5_DefaultValue_xjal();
if (callOnChangeActions) {
  self.set_maternity5( maternity5_xjal );
} else {
  self.maternity5 = maternity5_xjal;
}
double healthCenter5_xjal;
healthCenter5_xjal = self._healthCenter5_DefaultValue_xjal();
if (callOnChangeActions) {
  self.set_healthCenter5( healthCenter5_xjal );
} else {
  self.healthCenter5 = healthCenter5_xjal;
}
double other5_xjal;
```

```
other5_xjal = self._other5_DefaultValue_xjal();
if (callOnChangeActions) {
  self.set_other5( other5_xjal );
} else {
  self.other5 = other5_xjal;
}
double community5_xjal;
community5_xjal = self._community5_DefaultValue_xjal();
if (callOnChangeActions) {
  self.set_community5( community5_xjal );
} else {
  self.community5 = community5_xjal;
}
double district5_xjal;
district5_xjal = self._district5_DefaultValue_xjal();
if (callOnChangeActions) {
  self.set_district5( district5_xjal );
} else {
  self.district5 = district5_xjal;
}
double central5SE_xjal;
central5SE_xjal = self._central5SE_DefaultValue_xjal();
if (callOnChangeActions) {
  self.set_central5SE( central5SE_xjal );
} else {
  self.central5SE = central5SE_xjal;
}
double clinic5SE_xjal;
clinic5SE_xjal = self._clinic5SE_DefaultValue_xjal();
if (callOnChangeActions) {
  self.set_clinic5SE( clinic5SE_xjal );
} else {
  self.clinic5SE = clinic5SE_xjal;
}
double maternity5SE_xjal;
maternity5SE_xjal = self._maternity5SE_DefaultValue_xjal();
if (callOnChangeActions) {
  self.set_maternity5SE( maternity5SE_xjal );
} else {
  self.maternity5SE = maternity5SE_xjal;
}
double healthCenter5SE_xjal;
healthCenter5SE_xjal = self._healthCenter5SE_DefaultValue_xjal();
if (callOnChangeActions) {
  self.set_healthCenter5SE( healthCenter5SE_xjal );
} else {
  self.healthCenter5SE = healthCenter5SE_xjal;
}
double other5SE_xjal;
other5SE_xjal = self._other5SE_DefaultValue_xjal();
if (callOnChangeActions) {
  self.set_other5SE( other5SE_xjal );
} else {
  self.other5SE = other5SE_xjal;
}
double community5SE_xjal;
community5SE_xjal = self._community5SE_DefaultValue_xjal();
if (callOnChangeActions) {
  self.set_community5SE( community5SE_xjal );
} else {
  self.community5SE = community5SE_xjal;
}
double district5SE_xjal;
```

```
district5SE_xjal = self._district5SE_DefaultValue_xjal();
if (callOnChangeActions) {
  self.set_district5SE( district5SE_xjal );
} else {
  self.district5SE = district5SE_xjal;
}
double centralNo5_xjal;
centralNo5_xjal = self._centralNo5_DefaultValue_xjal();
if (callOnChangeActions) {
  self.set_centralNo5( centralNo5_xjal );
} else {
  self.centralNo5 = centralNo5_xjal;
}
double clinicNo5_xjal;
clinicNo5_xjal = self._clinicNo5_DefaultValue_xjal();
if (callOnChangeActions) {
  self.set_clinicNo5( clinicNo5_xjal );
} else {
  self.clinicNo5 = clinicNo5_xjal;
}
double maternityNo5_xjal;
maternityNo5_xjal = self._maternityNo5_DefaultValue_xjal();
if (callOnChangeActions) {
  self.set_maternityNo5( maternityNo5_xjal );
} else {
  self.maternityNo5 = maternityNo5_xjal;
}
double healthCenterNo5_xjal;
healthCenterNo5_xjal = self._healthCenterNo5_DefaultValue_xjal();
if (callOnChangeActions) {
  self.set_healthCenterNo5( healthCenterNo5_xjal );
} else {
  self.healthCenterNo5 = healthCenterNo5_xjal;
}
double otherNo5_xjal;
otherNo5_xjal = self._otherNo5_DefaultValue_xjal();
if (callOnChangeActions) {
  self.set_otherNo5( otherNo5_xjal );
} else {
  self.otherNo5 = otherNo5_xjal;
}
double communityNo5_xjal;
communityNo5_xjal = self._communityNo5_DefaultValue_xjal();
if (callOnChangeActions) {
  self.set_communityNo5( communityNo5_xjal );
} else {
  self.communityNo5 = communityNo5_xjal;
}
double districtNo5_xjal;
districtNo5_xjal = self._districtNo5_DefaultValue_xjal();
if (callOnChangeActions) {
  self.set_districtNo5( districtNo5_xjal );
} else {
  self.districtNo5 = districtNo5_xjal;
}
double centralNo5SE_xjal;
centralNo5SE_xjal = self._centralNo5SE_DefaultValue_xjal();
if (callOnChangeActions) {
  self.set_centralNo5SE( centralNo5SE_xjal );
} else {
  self.centralNo5SE = centralNo5SE_xjal;
}
double clinicNo5SE_xjal;
```

```
clinicNo5SE_xjal = self._clinicNo5SE_DefaultValue_xjal();
if (callOnChangeActions) {
  self.set_clinicNo5SE( clinicNo5SE_xjal );
} else {
  self.clinicNo5SE = clinicNo5SE_xjal;
}
double maternityNo5SE_xjal;
maternityNo5SE_xjal = self._maternityNo5SE_DefaultValue_xjal();
if (callOnChangeActions) {
  self.set_maternityNo5SE( maternityNo5SE_xjal );
} else {
  self.maternityNo5SE = maternityNo5SE_xjal;
}
double healthCenterNo5SE_xjal;
healthCenterNo5SE_xjal = self._healthCenterNo5SE_DefaultValue_xjal();
if (callOnChangeActions) {
  self.set_healthCenterNo5SE( healthCenterNo5SE_xjal );
} else {
  self.healthCenterNo5SE = healthCenterNo5SE_xjal;
}
double otherNo5SE_xjal;
otherNo5SE_xjal = self._otherNo5SE_DefaultValue_xjal();
if (callOnChangeActions) {
  self.set_otherNo5SE( otherNo5SE_xjal );
} else {
  self.otherNo5SE = otherNo5SE_xjal;
}
double communityNo5SE_xjal;
communityNo5SE_xjal = self._communityNo5SE_DefaultValue_xjal();
if (callOnChangeActions) {
  self.set_communityNo5SE( communityNo5SE_xjal );
} else {
  self.communityNo5SE = communityNo5SE_xjal;
}
double districtNo5SE_xjal;
districtNo5SE_xjal = self._districtNo5SE_DefaultValue_xjal();
if (callOnChangeActions) {
  self.set_districtNo5SE( districtNo5SE_xjal );
} else {
  self.districtNo5SE = districtNo5SE_xjal;
}
double anc4_xjal;
anc4_xjal = self._anc4_DefaultValue_xjal();
if (callOnChangeActions) {
  self.set_anc4( anc4_xjal );
} else {
  self.anc4 = anc4_xjal;
}
double unwanted_xjal;
unwanted_xjal = self._unwanted_DefaultValue_xjal();
if (callOnChangeActions) {
  self.set_unwanted( unwanted_xjal );
} else {
  self.unwanted = unwanted_xjal;
}
double risk_xjal;
risk_xjal = self._risk_DefaultValue_xjal();
if (callOnChangeActions) {
  self.set_risk( risk_xjal );
} else {
  self.risk = risk_xjal;
}
double urban_xjal;
```

```
urban_xjal = self._urban_DefaultValue_xjal();
if (callOnChangeActions) {
  self.set_urban( urban_xjal );
} else {
  self.urban = urban_xjal;
}
double twins_xjal;
twins_xjal = self._twins_DefaultValue_xjal();
if (callOnChangeActions) {
  self.set_twins( twins_xjal );
} else {
  self.twins = twins_xjal;
}
double educSec_xjal;
educSec_xjal = self._educSec_DefaultValue_xjal();
if (callOnChangeActions) {
  self.set_educSec( educSec_xjal );
} else {
  self.educSec = educSec_xjal;
}
double spouseAge_xjal;
spouseAge_xjal = self._spouseAge_DefaultValue_xjal();
if (callOnChangeActions) {
  self.set_spouseAge( spouseAge_xjal );
} else {
  self.spouseAge = spouseAge_xjal;
}
double age_xjal;
age_xjal = self._age_DefaultValue_xjal();
if (callOnChangeActions) {
  self.set_age( age_xjal );
} else {
  self.age = age_xjal;
}
double csPlanned_xjal;
csPlanned_xjal = self._csPlanned_DefaultValue_xjal();
if (callOnChangeActions) {
  self.set_csPlanned( csPlanned_xjal );
} else {
  self.csPlanned = csPlanned_xjal;
}
double anc4SE_xjal;
anc4SE_xjal = self._anc4SE_DefaultValue_xjal();
if (callOnChangeActions) {
  self.set_anc4SE( anc4SE_xjal );
} else {
  self.anc4SE = anc4SE_xjal;
}
double unwantedSE_xjal;
unwantedSE_xjal = self._unwantedSE_DefaultValue_xjal();
if (callOnChangeActions) {
  self.set_unwantedSE( unwantedSE_xjal );
} else {
  self.unwantedSE = unwantedSE_xjal;
}
double riskSE_xjal;
riskSE_xjal = self._riskSE_DefaultValue_xjal();
if (callOnChangeActions) {
  self.set_riskSE( riskSE_xjal );
} else {
  self.riskSE = riskSE_xjal;
}
double urbanSE_xjal;
```

```
    urbanSE_xjal = self._urbanSE_DefaultValue_xjal();
    if (callOnChangeActions) {
      self.set_urbanSE( urbanSE_xjal );
    } else {
      self.urbanSE = urbanSE_xjal;
    }
    double twinsSE_xjal;
    twinsSE_xjal = self._twinsSE_DefaultValue_xjal();
    if (callOnChangeActions) {
      self.set_twinsSE( twinsSE_xjal );
    } else {
      self.twinsSE = twinsSE_xjal;
    }
    double educSecSE_xjal;
    educSecSE_xjal = self._educSecSE_DefaultValue_xjal();
    if (callOnChangeActions) {
      self.set_educSecSE( educSecSE_xjal );
    } else {
      self.educSecSE = educSecSE_xjal;
    }
    double spouseAgeSE_xjal;
    spouseAgeSE_xjal = self._spouseAgeSE_DefaultValue_xjal();
    if (callOnChangeActions) {
      self.set_spouseAgeSE( spouseAgeSE_xjal );
    } else {
      self.spouseAgeSE = spouseAgeSE_xjal;
    }
    double ageSE_xjal;
    ageSE_xjal = self._ageSE_DefaultValue_xjal();
    if (callOnChangeActions) {
      self.set_ageSE( ageSE_xjal );
    } else {
      self.ageSE = ageSE_xjal;
    }
    double csPlannedSE_xjal;
    csPlannedSE_xjal = self._csPlannedSE_DefaultValue_xjal();
    if (callOnChangeActions) {
      self.set_csPlannedSE( csPlannedSE_xjal );
    } else {
      self.csPlannedSE = csPlannedSE_xjal;
    }
    double QIParam_xjal;
    QIParam_xjal = self._QIParam_DefaultValue_xjal();
    if (callOnChangeActions) {
      self.set_QIParam( QIParam_xjal );
    } else {
      self.QIParam = QIParam_xjal;
    }
    int strategy_xjal;
    strategy_xjal = 
strategies.getValue()
;
    if (callOnChangeActions) {
      self.set_strategy( strategy_xjal );
    } else {
      self.strategy = strategy_xjal;
    }
  }


  /**
   * Engine setup
   */
```

```
  @Override
  @AnyLogicInternalCodegenAPI
  public void setupEngine(Engine engine) {
    engine.setATOL( 1.0E-5 );
    engine.setRTOL( 1.0E-5 );
    engine.setTTOL( 1.0E-5 );
    engine.setHTOL( 0.001 );
    engine.setSolverODE( Engine.SOLVER_ODE_EULER );
    engine.setSolverNAE( Engine.SOLVER_NAE_MODIFIED_NEWTON );
    engine.setSolverDAE( Engine.SOLVER_DAE_RK45_NEWTON );
    engine.setVMethods( 427829 );
    engine.setSimultaneousEventsSelectionMode( Engine.EVENT_SELECTION_LIFO );

    engine.setStartTime( 0.0 );
    engine.setTimeUnit( SECOND );
    engine.setStartDate( toDate( 2017, JUNE, 4, 0, 0, 0 ) );
    engine.setStopTime( 3.0 );
    engine.setRealTimeMode( false );
  }

  /**
   * Experiment setup
   */
  @Override
  @AnyLogicInternalCodegenAPI
  public void setup( java.awt.Container container ) {
    setName( "Malawi Recentralization Model 2017-06-04 : Simulation" );
    Presentation _p = new Presentation( this, container instanceof JApplet ?
Presentation.MODE_APPLET :
      container != null ? Presentation.MODE_COMPONENT :
       Presentation.MODE_APPLICATION, container );

    // Static initialization of persistent elements
    _createPersistentElementsBP0_xjal();

    // Dynamic initialization of persistent elements
    _createPersistentElementsAP0_xjal();
    presentation = new ShapeTopLevelPresentationGroup( SimulationNewStrategies.this,
true, 0, 0, 0, 0 , text, allowList, QIText, QIText2, text1, text2, text3, allowList1,
button, allowFees, nonHospitals, QIscore, strategies );
    icon = new ShapeModelElementsGroup( SimulationNewStrategies.this,
getElementProperty(
"malawi_recentralization_model_2017_06_04.SimulationNewStrategies.icon",
IElementDescriptor.MODEL_ELEMENT_DESCRIPTORS )  );
    // Setup presentation
    _p.start();

    Panel _panel = _p.getPanel();
    ToolBar _tb = _p.getToolBar();
    StatusBar _sb = _p.getStatusBar();

    _panel.setFrameManagementBalance( 2.0 );

    _sb.setSectionVisible( StatusBar.DATE, true );
    _sb.setSectionVisible( StatusBar.EPS, false );
    _sb.setSectionVisible( StatusBar.EXPERIMENT, false );
    _sb.setSectionVisible( StatusBar.FPS, false );
    _sb.setSectionVisible( StatusBar.MEMORY, true );
    _sb.setSectionVisible( StatusBar.SECONDS, false );
    _sb.setSectionVisible( StatusBar.SIMULATION, true );
    _sb.setSectionVisible( StatusBar.STATUS, true );
    _sb.setSectionVisible( StatusBar.STEP, false );
    _sb.setSectionVisible( StatusBar.TIME, true );
```

```
    _tb.setSectionVisible( ToolBar.ANIMATION, false );
    _tb.setSectionVisible( ToolBar.EXECUTION, true );
    _tb.setSectionVisible( ToolBar.FILE, false );
    _tb.setSectionVisible( ToolBar.NAVIGATION, true );
    _tb.setSectionVisible( ToolBar.TIME_SCALE, true );
    _tb.setSectionVisible( ToolBar.VIEW, false );
    allowFees.setValueToDefault();
    nonHospitals.setValueToDefault();
    QIscore.setValueToDefault();
  }

}
```

```
package malawi_recentralization_model_2017_06_04;

import java.io.Serializable;
import java.sql.Connection;
import java.sql.SQLException;
import java.util.ArrayDeque;
import java.util.ArrayList;
import java.util.Arrays;
import java.util.Calendar;
import java.util.Collection;
import java.util.Collections;
import java.util.Comparator;
import java.util.Currency;
import java.util.Date;
import java.util.Enumeration;
import java.util.HashMap;
import java.util.HashSet;
import java.util.Hashtable;
import java.util.Iterator;
import java.util.LinkedHashMap;
import java.util.LinkedHashSet;
import java.util.LinkedList;
import java.util.List;
import java.util.ListIterator;
import java.util.Locale;
import java.util.Map;
import java.util.PriorityQueue;
import java.util.Random;
import java.util.Set;
import java.util.SortedMap;
import java.util.SortedSet;
import java.util.Stack;
import java.util.Timer;
import java.util.TreeMap;
import java.util.TreeSet;
import java.util.Vector;
import java.awt.Color;
import java.awt.Font;
import java.awt.Graphics2D;
import java.awt.geom.AffineTransform;
import com.anylogic.engine.connectivity.ResultSet;
import com.anylogic.engine.connectivity.Statement;
import com.anylogic.engine.elements.*;
import com.anylogic.engine.markup.Network;
import com.anylogic.engine.Position;
import com.anylogic.engine.markup.PedFlowStatistics;
import com.anylogic.engine.markup.DensityMap;
```

```
import static java.lang.Math.*;
import static com.anylogic.engine.UtilitiesArray.*;
import static com.anylogic.engine.UtilitiesCollection.*;
import static com.anylogic.engine.presentation.UtilitiesColor.*;
import static com.anylogic.engine.HyperArray.*;

import com.anylogic.engine.*;
import com.anylogic.engine.analysis.*;
import com.anylogic.engine.connectivity.*;
import com.anylogic.engine.database.*;
import com.anylogic.engine.gis.*;
import com.anylogic.engine.markup.*;
import com.anylogic.engine.presentation.*;

import com.mysema.query.Tuple;
import com.mysema.query.sql.SQLBindings;
import static malawi_recentralization_model_2017_06_04.DBDescriptor.*;


import javax.swing.JApplet;



public class ParamVar_NewStrategies extends ExperimentParamVariation<Main> {
  @AnyLogicInternalCodegenAPI
  public static String[] COMMAND_LINE_ARGUMENTS_xjal = new String[0];
  {
    setCommandLineArguments_xjal( COMMAND_LINE_ARGUMENTS_xjal );
  }
  // Excel Files
  public ExcelFile modelOutput = new ExcelFile( ParamVar_NewStrategies.this,
"/malawi_recentralization_model_2017_06_04/", "/Users/Mark/Dropbox/@Papers/HQSS/Model
output/Output template.xlsx", false );

  @AnyLogicInternalCodegenAPI
  private static Map<String, IElementDescriptor> elementDesciptors_xjal =
createElementDescriptors( ParamVar_NewStrategies.class );

  @AnyLogicInternalCodegenAPI
  @Override
  public Map<String, IElementDescriptor> getElementDesciptors() {
    return elementDesciptors_xjal;
  }
  // View areas
  @Override
  @AnyLogicInternalCodegenAPI
  public void drawModelElements(Panel _panel, Graphics2D _g, boolean _publicOnly,
boolean _isSuperClass ) {
    if (!_publicOnly) {
      drawExcelFile( _panel, _g, -200, 50, 10, 0, "modelOutput", modelOutput );
    }
}
  @Override
  @AnyLogicInternalCodegenAPI
  public boolean onClickModelAt( Panel _panel, double _x, double _y, int _clickCount,
boolean _publicOnly, boolean _isSuperClass ) {
    if( !_publicOnly && modelElementContains(_x, _y, -200, 50) ){
      _panel.addInspect( -200, 50, this, "modelOutput" );
      return true;
    }
    return false;
```

```
  }
  @AnyLogicInternalCodegenAPI
  protected static final Font _button_Font = new Font("Dialog", 0, 11 );
  @AnyLogicInternalCodegenAPI
  protected static final Font _text_Font = new Font("SansSerif", 0, 24 );
  @AnyLogicInternalCodegenAPI
  protected static final Font _text1_Font = new Font("SansSerif", 0, 12 );
  @AnyLogicInternalCodegenAPI
  protected static final Font _text2_Font = _text1_Font;
  @AnyLogicInternalCodegenAPI
  protected static final Font _text3_Font = new Font("SansSerif", 1, 12 );
  @AnyLogicInternalCodegenAPI
  protected static final Font _text22_Font = _text1_Font;
  @AnyLogicInternalCodegenAPI
  protected static final Font _text23_Font = _text1_Font;
  @AnyLogicInternalCodegenAPI
  protected static final int _button = 1;
  @AnyLogicInternalCodegenAPI
  protected static final int _text = 2;
  @AnyLogicInternalCodegenAPI
  protected static final int _text1 = 3;
  @AnyLogicInternalCodegenAPI
  protected static final int _text2 = 4;
  @AnyLogicInternalCodegenAPI
  protected static final int _line = 5;
  @AnyLogicInternalCodegenAPI
  protected static final int _text3 = 6;
  @AnyLogicInternalCodegenAPI
  protected static final int _text22 = 7;
  @AnyLogicInternalCodegenAPI
  protected static final int _text23 = 8;

  /** Internal constant, shouldn't be accessed by user */
  @AnyLogicInternalCodegenAPI
  protected static final int _SHAPE_NEXT_ID_xjal = 9;


  /**
   * Top-level presentation group id
   */
  @AnyLogicInternalCodegenAPI
  protected static final int _presentation = 0;

  /**
   * Top-level icon group id
   */
  @AnyLogicInternalCodegenAPI
  protected static final int _icon = -1;


  @Override
  @AnyLogicInternalCodegenAPI
  public void executeShapeControlAction( int _shape, int index ) {
    switch( _shape ) {
      case _button: {
          ShapeButton self = this.button;
run();
;}
        break;
      default:
        super.executeShapeControlAction( _shape, index );
        break;
    }
```

```
  }


  /**
   * <i>This method should not be called by user</i>
   */
  @AnyLogicInternalCodegenAPI
  private void _button_SetDynamicParams_xjal( ShapeButton shape ) {
    shape.setEnabled(
getState() == IDLE
);
  }

  protected ShapeButton button;
  protected ShapeText text;
  protected ShapeText text1;

  /**
   * <i>This method should not be called by user</i>
   */
  @AnyLogicInternalCodegenAPI
  private void _text2_SetDynamicParams_xjal( ShapeText shape ) {
    boolean _visible =
getCurrentIteration() > 0
;
    shape.setVisible( _visible );
      if ( _visible ) {
    shape.setText(
format(getCurrentIteration())
);
      }
  }

  protected ShapeText text2;
  protected ShapeLine line;
  protected ShapeText text3;
  protected ShapeText text22;

  /**
   * <i>This method should not be called by user</i>
   */
  @AnyLogicInternalCodegenAPI
  private void _text23_SetDynamicParams_xjal( ShapeText shape ) {
    boolean _visible =
getCurrentIteration() > 0
;
    shape.setVisible( _visible );
      if ( _visible ) {
    shape.setText(
format( getCurrentIteration() / (getMaximumIterations() / 5) )
);
      }
  }

  protected ShapeText text23;
  @AnyLogicInternalCodegenAPI
  private void _createPersistentElementsBP0_xjal() {
    button = new ShapeButton(
                ParamVar_NewStrategies.this, true, 40.0, 80.0,
                100.0, 30.0,
            controlDefault, controlDefault, true,
            _button_Font,
                "Run" ) {
```

```
      @Override
      public void updateDynamicProperties(boolean publicOnly) {
        _button_SetDynamicParams_xjal( this );
        super.updateDynamicProperties(publicOnly);
      }

      @Override
      @AnyLogicInternalCodegenAPI
      public void action(){
        executeShapeControlAction( _button, 0 );
      }
    };

    text = new ShapeText(
        SHAPE_DRAW_2D, true,40.0, 30.0, 0.0, 0.0,
        royalBlue,"Malawi Recentralization Model stochastic 2017-11-01 :
ParameterVariation",
        _text_Font, ALIGNMENT_LEFT );

    text1 = new ShapeText(
        SHAPE_DRAW_2D, true,50.0, 130.0, 0.0, 0.0,
        black,"Iteration:",
        _text1_Font, ALIGNMENT_LEFT );

    text2 = new ShapeText(
        SHAPE_DRAW_2D, true,270.0, 130.0, 0.0, 0.0,
        darkSlateBlue,"?",
        _text2_Font, ALIGNMENT_RIGHT ) {
      @Override
      public void updateDynamicProperties(boolean publicOnly) {
        _text2_SetDynamicParams_xjal( this );
        super.updateDynamicProperties(publicOnly);
      }
    };

    line = new ShapeLine(
             SHAPE_DRAW_2D, true, 40.0, 150.0, 0.0, black,
             240.0, 0.0, 0.0, 1.0, 10.0, LINE_STYLE_SOLID );

    text3 = new ShapeText(
        SHAPE_DRAW_2D, true,50.0, 160.0, 0.0, 0.0,
        black,"Parameters",
        _text3_Font, ALIGNMENT_LEFT );

    text22 = new ShapeText(
        SHAPE_DRAW_2D, true,50.0, 190.0, 0.0, 0.0,
        black,"strategy",
        _text22_Font, ALIGNMENT_LEFT );

    text23 = new ShapeText(
        SHAPE_DRAW_2D, true,270.0, 190.0, 0.0, 0.0,
        darkSlateBlue,"?",
        _text23_Font, ALIGNMENT_RIGHT ) {
      @Override
      public void updateDynamicProperties(boolean publicOnly) {
        _text23_SetDynamicParams_xjal( this );
        super.updateDynamicProperties(publicOnly);
      }
    };

  }

  @AnyLogicInternalCodegenAPI
```

```
  private void _createPersistentElementsAP0_xjal() {
  }


  protected ShapeTopLevelPresentationGroup presentation;
  protected ShapeModelElementsGroup icon;

  @Override
  @AnyLogicInternalCodegenAPI
  public ShapeTopLevelPresentationGroup getPresentationShape() {
    return presentation;
  }

  @Override
  @AnyLogicInternalCodegenAPI
  public ShapeModelElementsGroup getModelElementsShape() {
    return icon;
  }

  @Override
  @AnyLogicInternalCodegenAPI
  public Object getPersistentShape( int _shape ) {
    switch (_shape) {
      case _presentation: return presentation;
      case _icon: return icon;
      case _button: return button;
      case _text: return text;
      case _text1: return text1;
      case _text2: return text2;
      case _line: return line;
      case _text3: return text3;
      case _text22: return text22;
      case _text23: return text23;
      default: return super.getPersistentShape( _shape );
    }
  }

  @Override
  @AnyLogicInternalCodegenAPI
  public String getNameOfShape_xjal( Object _shape ) {
    try {
      if ( _shape == null ) return null;
      String _name_xjal;
      _name_xjal = checkNameOfShape_xjal( _shape, presentation, "presentation" ); if
(_name_xjal != null) return _name_xjal;
      _name_xjal = checkNameOfShape_xjal( _shape, icon, "icon" ); if (_name_xjal !=
null) return _name_xjal;
      _name_xjal = checkNameOfShape_xjal( _shape, button, "button" ); if (_name_xjal
!= null) return _name_xjal;
      _name_xjal = checkNameOfShape_xjal( _shape, text, "text" ); if (_name_xjal !=
null) return _name_xjal;
      _name_xjal = checkNameOfShape_xjal( _shape, text1, "text1" ); if (_name_xjal !=
null) return _name_xjal;
      _name_xjal = checkNameOfShape_xjal( _shape, text2, "text2" ); if (_name_xjal !=
null) return _name_xjal;
      _name_xjal = checkNameOfShape_xjal( _shape, line, "line" ); if (_name_xjal !=
null) return _name_xjal;
      _name_xjal = checkNameOfShape_xjal( _shape, text3, "text3" ); if (_name_xjal !=
null) return _name_xjal;
      _name_xjal = checkNameOfShape_xjal( _shape, text22, "text22" ); if (_name_xjal
!= null) return _name_xjal;
      _name_xjal = checkNameOfShape_xjal( _shape, text23, "text23" ); if (_name_xjal
!= null) return _name_xjal;
```

```
  } catch (Exception e) {
    return null;
  }
  return super.getNameOfShape_xjal( _shape );
}

@Override
public int getWindowWidth() {
  return 1550;
}

@Override
public int getWindowHeight() {
  return 890;
}

@Override
@AnyLogicInternalCodegenAPI
public void onDestroy_xjal() {
  // Destroy database objects
  modelOutput.writeFile();
  super.onDestroy_xjal();
}

/**
 * Applet class to run experiment as java applet
 */
@AnyLogicInternalCodegenAPI
public static class Applet extends JApplet {

@AnyLogicInternalCodegenAPI
  ParamVar_NewStrategies ex;

  @Override
  public void init() {
    ex = new ParamVar_NewStrategies();
    ex.setup( this );
  }

  @Override
  public void destroy() {
    ex.close();
  }

}

@Override
@AnyLogicInternalCodegenAPI
public void initDefaultRandomNumberGenerator(Engine _e) {
  _e.setDefaultRandomGenerator( new java.util.Random() );
}

// Parameter values (read-only)
public boolean allowFeesParam;
public double centralHosp1;
public double distance1;
public double clinic1;
public double maternity1;
public double healthCenter1;
public double otherHosp1;
public double communityHosp1;
public double districtHosp1;
public double bobs1;
```

```
public double fees1;
public boolean allowAllParam;
public double intercept;
public double centralHosp2;
public double distance2;
public double clinic2;
public double maternity2;
public double healthCenter2;
public double otherHosp2;
public double communityHosp2;
public double districtHosp2;
public double bobs2;
public double fees2;
public double distCenter;
public double distRMS;
public double centralHosp1SE;
public double distance1SE;
public double clinic1SE;
public double maternity1SE;
public double healthCenter1SE;
public double otherHosp1SE;
public double communityHosp1SE;
public double districtHosp1SE;
public double bobs1SE;
public double fees1SE;
public double interceptSE;
public double centralHosp2SE;
public double distance2SE;
public double clinic2SE;
public double maternity2SE;
public double healthCenter2SE;
public double otherHosp2SE;
public double communityHosp2SE;
public double districtHosp2SE;
public double bobs2SE;
public double fees2SE;
public double centralCS;
public double clinicCS;
public double maternityCS;
public double healthCenterCS;
public double otherCS;
public double communityCS;
public double districtCS;
public double centralCSSE;
public double clinicCSSE;
public double maternityCSSE;
public double healthCenterCSSE;
public double otherCSSE;
public double communityCSSE;
public double districtCSSE;
public double centralNo;
public double clinicNo;
public double maternityNo;
public double healthCenterNo;
public double otherNo;
public double communityNo;
public double districtNo;
public double centralNoSE;
public double clinicNoSE;
public double maternityNoSE;
public double healthCenterNoSE;
public double otherNoSE;
public double communityNoSE;
```

```
public double districtNoSE;
public double central5;
public double clinic5;
public double maternity5;
public double healthCenter5;
public double other5;
public double community5;
public double district5;
public double central5SE;
public double clinic5SE;
public double maternity5SE;
public double healthCenter5SE;
public double other5SE;
public double community5SE;
public double district5SE;
public double centralNo5;
public double clinicNo5;
public double maternityNo5;
public double healthCenterNo5;
public double otherNo5;
public double communityNo5;
public double districtNo5;
public double centralNo5SE;
public double clinicNo5SE;
public double maternityNo5SE;
public double healthCenterNo5SE;
public double otherNo5SE;
public double communityNo5SE;
public double districtNo5SE;
public double anc4;
public double unwanted;
public double risk;
public double urban;
public double twins;
public double educSec;
public double spouseAge;
public double age;
public double csPlanned;
public double anc4SE;
public double unwantedSE;
public double riskSE;
public double urbanSE;
public double twinsSE;
public double educSecSE;
public double spouseAgeSE;
public double ageSE;
public double csPlannedSE;
public double QIParam;
public int strategy;

/**
 * Engine setup
 */
@Override
@AnyLogicInternalCodegenAPI
public void setupEngine(Engine engine) {
  engine.setATOL( 1.0E-5 );
  engine.setRTOL( 1.0E-5 );
  engine.setTTOL( 1.0E-5 );
  engine.setHTOL( 0.001 );
  engine.setSolverODE( Engine.SOLVER_ODE_EULER );
  engine.setSolverNAE( Engine.SOLVER_NAE_MODIFIED_NEWTON );
  engine.setSolverDAE( Engine.SOLVER_DAE_RK45_NEWTON );
```

```
    engine.setVMethods( 427829 );
    engine.setSimultaneousEventsSelectionMode( Engine.EVENT_SELECTION_LIFO );

    engine.setStartTime( 0.0 );
    engine.setTimeUnit( SECOND );
    engine.setStartDate( toDate( 2017, JUNE, 4, 0, 0, 0 ) );
    engine.setStopTime( 2.0 );
  }

  /**
   * Experiment setup
   */
  @Override
  @AnyLogicInternalCodegenAPI
  public void setup( java.awt.Container container ) {
    setName( "Malawi Recentralization Model stochastic 2017-11-01 :
ParameterVariation" );
    Presentation _p = new Presentation( this, container instanceof JApplet ?
Presentation.MODE_APPLET :
       container != null ? Presentation.MODE_COMPONENT :
        Presentation.MODE_APPLICATION, container );

    // Static initialization of persistent elements
    _createPersistentElementsBP0_xjal();

    modelOutput.readFile();


    // Dynamic initialization of persistent elements
    _createPersistentElementsAP0_xjal();
    presentation = new ShapeTopLevelPresentationGroup( ParamVar_NewStrategies.this,
true, 0, 0, 0, 0 , text, text1, text2, line, text3, text22, text23, button );
    icon = new ShapeModelElementsGroup( ParamVar_NewStrategies.this,
getElementProperty(
"malawi_recentralization_model_2017_06_04.ParamVar_NewStrategies.icon",
IElementDescriptor.MODEL_ELEMENT_DESCRIPTORS )  );
    // Setup presentation
    _p.start();

    Panel _panel = _p.getPanel();
    ToolBar _tb = _p.getToolBar();
    StatusBar _sb = _p.getStatusBar();

    _panel.setFrameManagementBalance( 2.0 );

    _sb.setSectionVisible( StatusBar.EPS, false );
    _sb.setSectionVisible( StatusBar.EXPERIMENT, true );
    _sb.setSectionVisible( StatusBar.FPS, false );
    _sb.setSectionVisible( StatusBar.MEMORY, true );
    _sb.setSectionVisible( StatusBar.SECONDS, true );
    _sb.setSectionVisible( StatusBar.SIMULATION, true );
    _sb.setSectionVisible( StatusBar.STATUS, true );
    _tb.setSectionVisible( ToolBar.ANIMATION, false );
    _tb.setSectionVisible( ToolBar.EXECUTION, true );
    _tb.setSectionVisible( ToolBar.FILE, false );
    _tb.setSectionEnabled( ToolBar.NAVIGATION, false );
    _tb.setSectionVisible( ToolBar.NAVIGATION, false );
    _tb.setSectionEnabled( ToolBar.TIME_SCALE, false );
    _tb.setSectionVisible( ToolBar.TIME_SCALE, false );
    _tb.setSectionVisible( ToolBar.VIEW, false );
  }

  @Override
```

```
@AnyLogicInternalCodegenAPI
public Main createRoot( Engine engine ) {
  // Create the root object
  return new Main( engine, null, null );
}

@Override
@AnyLogicInternalCodegenAPI
public void onBeforeSimulationRun(Main root) {
  allowFeesParam = root.allowFeesParam;
  centralHosp1 = root.centralHosp1;
  distance1 = root.distance1;
  clinic1 = root.clinic1;
  maternity1 = root.maternity1;
  healthCenter1 = root.healthCenter1;
  otherHosp1 = root.otherHosp1;
  communityHosp1 = root.communityHosp1;
  districtHosp1 = root.districtHosp1;
  bobs1 = root.bobs1;
  fees1 = root.fees1;
  allowAllParam = root.allowAllParam;
  intercept = root.intercept;
  centralHosp2 = root.centralHosp2;
  distance2 = root.distance2;
  clinic2 = root.clinic2;
  maternity2 = root.maternity2;
  healthCenter2 = root.healthCenter2;
  otherHosp2 = root.otherHosp2;
  communityHosp2 = root.communityHosp2;
  districtHosp2 = root.districtHosp2;
  bobs2 = root.bobs2;
  fees2 = root.fees2;
  distCenter = root.distCenter;
  distRMS = root.distRMS;
  centralHosp1SE = root.centralHosp1SE;
  distance1SE = root.distance1SE;
  clinic1SE = root.clinic1SE;
  maternity1SE = root.maternity1SE;
  healthCenter1SE = root.healthCenter1SE;
  otherHosp1SE = root.otherHosp1SE;
  communityHosp1SE = root.communityHosp1SE;
  districtHosp1SE = root.districtHosp1SE;
  bobs1SE = root.bobs1SE;
  fees1SE = root.fees1SE;
  interceptSE = root.interceptSE;
  centralHosp2SE = root.centralHosp2SE;
  distance2SE = root.distance2SE;
  clinic2SE = root.clinic2SE;
  maternity2SE = root.maternity2SE;
  healthCenter2SE = root.healthCenter2SE;
  otherHosp2SE = root.otherHosp2SE;
  communityHosp2SE = root.communityHosp2SE;
  districtHosp2SE = root.districtHosp2SE;
  bobs2SE = root.bobs2SE;
  fees2SE = root.fees2SE;
  centralCS = root.centralCS;
  clinicCS = root.clinicCS;
  maternityCS = root.maternityCS;
  healthCenterCS = root.healthCenterCS;
  otherCS = root.otherCS;
  communityCS = root.communityCS;
  districtCS = root.districtCS;
  centralCSSE = root.centralCSSE;
```

```
clinicCSSE = root.clinicCSSE;
maternityCSSE = root.maternityCSSE;
healthCenterCSSE = root.healthCenterCSSE;
otherCSSE = root.otherCSSE;
communityCSSE = root.communityCSSE;
districtCSSE = root.districtCSSE;
centralNo = root.centralNo;
clinicNo = root.clinicNo;
maternityNo = root.maternityNo;
healthCenterNo = root.healthCenterNo;
otherNo = root.otherNo;
communityNo = root.communityNo;
districtNo = root.districtNo;
centralNoSE = root.centralNoSE;
clinicNoSE = root.clinicNoSE;
maternityNoSE = root.maternityNoSE;
healthCenterNoSE = root.healthCenterNoSE;
otherNoSE = root.otherNoSE;
communityNoSE = root.communityNoSE;
districtNoSE = root.districtNoSE;
central5 = root.central5;
clinic5 = root.clinic5;
maternity5 = root.maternity5;
healthCenter5 = root.healthCenter5;
other5 = root.other5;
community5 = root.community5;
district5 = root.district5;
central5SE = root.central5SE;
clinic5SE = root.clinic5SE;
maternity5SE = root.maternity5SE;
healthCenter5SE = root.healthCenter5SE;
other5SE = root.other5SE;
community5SE = root.community5SE;
district5SE = root.district5SE;
centralNo5 = root.centralNo5;
clinicNo5 = root.clinicNo5;
maternityNo5 = root.maternityNo5;
healthCenterNo5 = root.healthCenterNo5;
otherNo5 = root.otherNo5;
communityNo5 = root.communityNo5;
districtNo5 = root.districtNo5;
centralNo5SE = root.centralNo5SE;
clinicNo5SE = root.clinicNo5SE;
maternityNo5SE = root.maternityNo5SE;
healthCenterNo5SE = root.healthCenterNo5SE;
otherNo5SE = root.otherNo5SE;
communityNo5SE = root.communityNo5SE;
districtNo5SE = root.districtNo5SE;
anc4 = root.anc4;
unwanted = root.unwanted;
risk = root.risk;
urban = root.urban;
twins = root.twins;
educSec = root.educSec;
spouseAge = root.spouseAge;
age = root.age;
csPlanned = root.csPlanned;
anc4SE = root.anc4SE;
unwantedSE = root.unwantedSE;
riskSE = root.riskSE;
urbanSE = root.urbanSE;
twinsSE = root.twinsSE;
educSecSE = root.educSecSE;
```

```
    spouseAgeSE = root.spouseAgeSE;
    ageSE = root.ageSE;
    csPlannedSE = root.csPlannedSE;
    QIParam = root.QIParam;
    strategy = root.strategy;

int nRuns = getMaximumIterations();
int iterNumber = getCurrentIteration();
int split = nRuns / 5;

if(iterNumber <= split){ root.set_strategy(0); }
else if(iterNumber <= split * 2){ root.set_strategy(1); }
else if(iterNumber <= split * 3){ root.set_strategy(2); }
else if(iterNumber <= split * 4){ root.set_strategy(3); }
else { root.set_strategy(4); }

//root.allowAllParam = (iterNumber < 201);
//root.allowFeesParam = ((iterNumber < 401) | (iterNumber >= 601 & iterNumber < 801));
//root.QIParam = (iterNumber > 601) ? 1 : 0;


  }

  @Override
  @AnyLogicInternalCodegenAPI
  public void onEngineFinished() {
    final Main root = (Main) getEngine().getRoot();
    // After simulation run code

int iterNumber = getCurrentIteration();
modelOutput.setCellValue(iterNumber,                              "Sheet1",
iterNumber + 1, 1);
modelOutput.setCellValue(root.strategy,                           "Sheet1",
iterNumber + 1, 2);

modelOutput.setCellValue(root.segment2,                           "Sheet1",
iterNumber + 1, 4);
modelOutput.setCellValue(root.travelDistance.getStatistics().mean(),      "Sheet1",
iterNumber + 1, 5);
modelOutput.setCellValue(root.utilityDifference,            "Sheet1", iterNumber + 1,
6);
modelOutput.setCellValue((double)root.people.nCentral() / (double)root.popSize,
       "Sheet1", iterNumber + 1, 7);
modelOutput.setCellValue((double)root.people.nDistrict() / (double)root.popSize,
       "Sheet1", iterNumber + 1, 8);
modelOutput.setCellValue((double)root.people.nCommunity() / (double)root.popSize,
       "Sheet1", iterNumber + 1, 9);
modelOutput.setCellValue((double)root.people.nOther() / (double)root.popSize,
       "Sheet1", iterNumber + 1, 10);

modelOutput.setCellValue((double)root.people.nHealthCenter() / (double)root.popSize,
       "Sheet1", iterNumber + 1, 11);
modelOutput.setCellValue((double)root.people.nMaternity() / (double)root.popSize,
       "Sheet1", iterNumber + 1, 12);
modelOutput.setCellValue((double)root.people.nClinic() / (double)root.popSize,
       "Sheet1", iterNumber + 1, 13);
modelOutput.setCellValue(root.bCentral1,                          "Sheet1",
iterNumber + 1, 14);
modelOutput.setCellValue(root.bDistrict1,                         "Sheet1",
iterNumber + 1, 15);
modelOutput.setCellValue(root.bCommunity1,                        "Sheet1",
iterNumber + 1, 16);
```

```
modelOutput.setCellValue(root.bOther1,                          "Sheet1",
iterNumber + 1, 17);
modelOutput.setCellValue(root.bHealthCenter1,             "Sheet1", iterNumber + 1,
18);
modelOutput.setCellValue(root.bMaternity1,                      "Sheet1",
iterNumber + 1, 19);
modelOutput.setCellValue(root.bClinic1,                         "Sheet1",
iterNumber + 1, 20);

modelOutput.setCellValue(root.bDistance1,                       "Sheet1",
iterNumber + 1, 21);
modelOutput.setCellValue(root.bQuality1,                        "Sheet1",
iterNumber + 1, 22);
modelOutput.setCellValue(root.bBobs1,                           "Sheet1",
iterNumber + 1, 23);
modelOutput.setCellValue(root.bFees1,                           "Sheet1",
iterNumber + 1, 24);
modelOutput.setCellValue(root.bCentral2,                        "Sheet1",
iterNumber + 1, 25);
modelOutput.setCellValue(root.bDistrict2,                       "Sheet1",
iterNumber + 1, 26);
modelOutput.setCellValue(root.bCommunity2,                      "Sheet1",
iterNumber + 1, 27);
modelOutput.setCellValue(root.bOther2,                          "Sheet1",
iterNumber + 1, 28);
modelOutput.setCellValue(root.bHealthCenter2,             "Sheet1", iterNumber + 1,
29);
modelOutput.setCellValue(root.bMaternity2,                      "Sheet1",
iterNumber + 1, 30);

modelOutput.setCellValue(root.bClinic2,                         "Sheet1",
iterNumber + 1, 31);
modelOutput.setCellValue(root.bDistance2,                       "Sheet1",
iterNumber + 1, 32);
modelOutput.setCellValue(root.bQuality2,                        "Sheet1",
iterNumber + 1, 33);
modelOutput.setCellValue(root.bBobs2,                           "Sheet1",
iterNumber + 1, 34);
modelOutput.setCellValue(root.bFees2,                           "Sheet1",
iterNumber + 1, 35);
modelOutput.setCellValue(root.bPoor,                            "Sheet1",
iterNumber + 1, 36);
modelOutput.setCellValue(root.bMiddle,                          "Sheet1",
iterNumber + 1, 37);
modelOutput.setCellValue(root.bRich,                            "Sheet1",
iterNumber + 1, 38);
modelOutput.setCellValue(root.bRichest,                         "Sheet1",
iterNumber + 1, 39);
modelOutput.setCellValue(root.bPrimip,                          "Sheet1",
iterNumber + 1, 40);

modelOutput.setCellValue(root.bBlind,                           "Sheet1",
iterNumber + 1, 41);
modelOutput.setCellValue(root.bIlliterate,                      "Sheet1",
iterNumber + 1, 42);
modelOutput.setCellValue(root.bIntercept,                       "Sheet1",
iterNumber + 1, 43);
modelOutput.setCellValue(root.popSize,                          "Sheet1",
iterNumber + 1, 44);
modelOutput.setCellValue(root.QI,                                      "Sheet1",
iterNumber + 1, 45);
modelOutput.setCellValue(root.hospitals.hospitalBobs(),    "Sheet1", iterNumber + 1,
46);
```

```
modelOutput.setCellValue(root.hospitals.hospitalFees(),     "Sheet1", iterNumber + 1,
47);
modelOutput.setCellValue(root.people.uDiffPoorest(), "Sheet1", iterNumber + 1, 48);
modelOutput.setCellValue(root.people.uDiffPoor(),           "Sheet1", iterNumber + 1,
49);
modelOutput.setCellValue(root.people.uDiffMiddle(),         "Sheet1", iterNumber + 1,
50);

modelOutput.setCellValue(root.people.uDiffRich(),           "Sheet1", iterNumber + 1,
51);
modelOutput.setCellValue(root.people.uDiffRichest(), "Sheet1", iterNumber + 1, 52);
modelOutput.setCellValue(root.dist60km,                             "Sheet1",
iterNumber + 1, 53);
modelOutput.setCellValue(root.distFurther,                          "Sheet1",
iterNumber + 1, 54);
modelOutput.setCellValue(root.meanBobs,                             "Sheet1",
iterNumber + 1, 55);
modelOutput.setCellValue(root.neonatalMortality,            "Sheet1", iterNumber + 1,
56);

modelOutput.setCellValue((double)root.people.mortPoorest()  /
(double)root.people.nPoorest(),  "Sheet1", iterNumber + 1, 57);
modelOutput.setCellValue((double)root.people.mortPoor()     /
(double)root.people.nPoor(),           "Sheet1", iterNumber + 1, 58);
modelOutput.setCellValue((double)root.people.mortMiddle()   /
(double)root.people.nMiddle(),   "Sheet1", iterNumber + 1, 59);
modelOutput.setCellValue((double)root.people.mortRich()     /
(double)root.people.nRich(),           "Sheet1", iterNumber + 1, 60);
modelOutput.setCellValue((double)root.people.mortRichest()  /
(double)root.people.nRichest(),  "Sheet1", iterNumber + 1, 61);

modelOutput.setCellValue(root.avgOOP,                "Sheet1", iterNumber + 1, 62);
modelOutput.setCellValue(root.csRate,                "Sheet1", iterNumber + 1, 63);

modelOutput.setCellValue(root.catExpTotal,           "Sheet1", iterNumber + 1, 64);
modelOutput.setCellValue(root.catExpPoorest,   "Sheet1", iterNumber + 1, 65);
modelOutput.setCellValue(root.catExpPoor,            "Sheet1", iterNumber + 1, 66);
modelOutput.setCellValue(root.catExpMiddle,          "Sheet1", iterNumber + 1, 67);
modelOutput.setCellValue(root.catExpRich,            "Sheet1", iterNumber + 1, 68);
modelOutput.setCellValue(root.catExpRichest,   "Sheet1", iterNumber + 1, 69);

  }

  @Override
  @AnyLogicInternalCodegenAPI
  public void onAfterExperiment() {
    // After experiment code

modelOutput.evaluateFormulas();
modelOutput.writeFile(true);
  }



  @Override
  @AnyLogicInternalCodegenAPI
  public void reset() {
    // Replications setup
    setUseReplications( false );
  }

  @Override
  public int getMaximumIterations() {
```

```
    return 5000;
  }

  @Override
  @AnyLogicInternalCodegenAPI
  public void setupRootParameters( final Main self, int index, boolean
callOnChangeActions ) {
    final Main root = self; // for compatibility
    boolean allowFeesParam_xjal;
    allowFeesParam_xjal = self._allowFeesParam_DefaultValue_xjal();
    if (callOnChangeActions) {
      self.set_allowFeesParam( allowFeesParam_xjal );
    } else {
      self.allowFeesParam = allowFeesParam_xjal;
    }
    double centralHosp1_xjal;
    centralHosp1_xjal = self._centralHosp1_DefaultValue_xjal();
    if (callOnChangeActions) {
      self.set_centralHosp1( centralHosp1_xjal );
    } else {
      self.centralHosp1 = centralHosp1_xjal;
    }
    double distance1_xjal;
    distance1_xjal = self._distance1_DefaultValue_xjal();
    if (callOnChangeActions) {
      self.set_distance1( distance1_xjal );
    } else {
      self.distance1 = distance1_xjal;
    }
    double clinic1_xjal;
    clinic1_xjal = self._clinic1_DefaultValue_xjal();
    if (callOnChangeActions) {
      self.set_clinic1( clinic1_xjal );
    } else {
      self.clinic1 = clinic1_xjal;
    }
    double maternity1_xjal;
    maternity1_xjal = self._maternity1_DefaultValue_xjal();
    if (callOnChangeActions) {
      self.set_maternity1( maternity1_xjal );
    } else {
      self.maternity1 = maternity1_xjal;
    }
    double healthCenter1_xjal;
    healthCenter1_xjal = self._healthCenter1_DefaultValue_xjal();
    if (callOnChangeActions) {
      self.set_healthCenter1( healthCenter1_xjal );
    } else {
      self.healthCenter1 = healthCenter1_xjal;
    }
    double otherHosp1_xjal;
    otherHosp1_xjal = self._otherHosp1_DefaultValue_xjal();
    if (callOnChangeActions) {
      self.set_otherHosp1( otherHosp1_xjal );
    } else {
      self.otherHosp1 = otherHosp1_xjal;
    }
    double communityHosp1_xjal;
    communityHosp1_xjal = self._communityHosp1_DefaultValue_xjal();
    if (callOnChangeActions) {
      self.set_communityHosp1( communityHosp1_xjal );
    } else {
      self.communityHosp1 = communityHosp1_xjal;
```

```
}
double districtHosp1_xjal;
districtHosp1_xjal = self._districtHosp1_DefaultValue_xjal();
if (callOnChangeActions) {
  self.set_districtHosp1( districtHosp1_xjal );
} else {
  self.districtHosp1 = districtHosp1_xjal;
}
double bobs1_xjal;
bobs1_xjal = self._bobs1_DefaultValue_xjal();
if (callOnChangeActions) {
  self.set_bobs1( bobs1_xjal );
} else {
  self.bobs1 = bobs1_xjal;
}
double fees1_xjal;
fees1_xjal = self._fees1_DefaultValue_xjal();
if (callOnChangeActions) {
  self.set_fees1( fees1_xjal );
} else {
  self.fees1 = fees1_xjal;
}
boolean allowAllParam_xjal;
allowAllParam_xjal = self._allowAllParam_DefaultValue_xjal();
if (callOnChangeActions) {
  self.set_allowAllParam( allowAllParam_xjal );
} else {
  self.allowAllParam = allowAllParam_xjal;
}
double intercept_xjal;
intercept_xjal = self._intercept_DefaultValue_xjal();
if (callOnChangeActions) {
  self.set_intercept( intercept_xjal );
} else {
  self.intercept = intercept_xjal;
}
double centralHosp2_xjal;
centralHosp2_xjal = self._centralHosp2_DefaultValue_xjal();
if (callOnChangeActions) {
  self.set_centralHosp2( centralHosp2_xjal );
} else {
  self.centralHosp2 = centralHosp2_xjal;
}
double distance2_xjal;
distance2_xjal = self._distance2_DefaultValue_xjal();
if (callOnChangeActions) {
  self.set_distance2( distance2_xjal );
} else {
  self.distance2 = distance2_xjal;
}
double clinic2_xjal;
clinic2_xjal = self._clinic2_DefaultValue_xjal();
if (callOnChangeActions) {
  self.set_clinic2( clinic2_xjal );
} else {
  self.clinic2 = clinic2_xjal;
}
double maternity2_xjal;
maternity2_xjal = self._maternity2_DefaultValue_xjal();
if (callOnChangeActions) {
  self.set_maternity2( maternity2_xjal );
} else {
  self.maternity2 = maternity2_xjal;
```

```
}
double healthCenter2_xjal;
healthCenter2_xjal = self._healthCenter2_DefaultValue_xjal();
if (callOnChangeActions) {
  self.set_healthCenter2( healthCenter2_xjal );
} else {
  self.healthCenter2 = healthCenter2_xjal;
}
double otherHosp2_xjal;
otherHosp2_xjal = self._otherHosp2_DefaultValue_xjal();
if (callOnChangeActions) {
  self.set_otherHosp2( otherHosp2_xjal );
} else {
  self.otherHosp2 = otherHosp2_xjal;
}
double communityHosp2_xjal;
communityHosp2_xjal = self._communityHosp2_DefaultValue_xjal();
if (callOnChangeActions) {
  self.set_communityHosp2( communityHosp2_xjal );
} else {
  self.communityHosp2 = communityHosp2_xjal;
}
double districtHosp2_xjal;
districtHosp2_xjal = self._districtHosp2_DefaultValue_xjal();
if (callOnChangeActions) {
  self.set_districtHosp2( districtHosp2_xjal );
} else {
  self.districtHosp2 = districtHosp2_xjal;
}
double bobs2_xjal;
bobs2_xjal = self._bobs2_DefaultValue_xjal();
if (callOnChangeActions) {
  self.set_bobs2( bobs2_xjal );
} else {
  self.bobs2 = bobs2_xjal;
}
double fees2_xjal;
fees2_xjal = self._fees2_DefaultValue_xjal();
if (callOnChangeActions) {
  self.set_fees2( fees2_xjal );
} else {
  self.fees2 = fees2_xjal;
}
double distCenter_xjal;
distCenter_xjal = self._distCenter_DefaultValue_xjal();
if (callOnChangeActions) {
  self.set_distCenter( distCenter_xjal );
} else {
  self.distCenter = distCenter_xjal;
}
double distRMS_xjal;
distRMS_xjal = self._distRMS_DefaultValue_xjal();
if (callOnChangeActions) {
  self.set_distRMS( distRMS_xjal );
} else {
  self.distRMS = distRMS_xjal;
}
double centralHosp1SE_xjal;
centralHosp1SE_xjal = self._centralHosp1SE_DefaultValue_xjal();
if (callOnChangeActions) {
  self.set_centralHosp1SE( centralHosp1SE_xjal );
} else {
  self.centralHosp1SE = centralHosp1SE_xjal;
```

```
}
double distance1SE_xjal;
distance1SE_xjal = self._distance1SE_DefaultValue_xjal();
if (callOnChangeActions) {
  self.set_distance1SE( distance1SE_xjal );
} else {
  self.distance1SE = distance1SE_xjal;
}
double clinic1SE_xjal;
clinic1SE_xjal = self._clinic1SE_DefaultValue_xjal();
if (callOnChangeActions) {
  self.set_clinic1SE( clinic1SE_xjal );
} else {
  self.clinic1SE = clinic1SE_xjal;
}
double maternity1SE_xjal;
maternity1SE_xjal = self._maternity1SE_DefaultValue_xjal();
if (callOnChangeActions) {
  self.set_maternity1SE( maternity1SE_xjal );
} else {
  self.maternity1SE = maternity1SE_xjal;
}
double healthCenter1SE_xjal;
healthCenter1SE_xjal = self._healthCenter1SE_DefaultValue_xjal();
if (callOnChangeActions) {
  self.set_healthCenter1SE( healthCenter1SE_xjal );
} else {
  self.healthCenter1SE = healthCenter1SE_xjal;
}
double otherHosp1SE_xjal;
otherHosp1SE_xjal = self._otherHosp1SE_DefaultValue_xjal();
if (callOnChangeActions) {
  self.set_otherHosp1SE( otherHosp1SE_xjal );
} else {
  self.otherHosp1SE = otherHosp1SE_xjal;
}
double communityHosp1SE_xjal;
communityHosp1SE_xjal = self._communityHosp1SE_DefaultValue_xjal();
if (callOnChangeActions) {
  self.set_communityHosp1SE( communityHosp1SE_xjal );
} else {
  self.communityHosp1SE = communityHosp1SE_xjal;
}
double districtHosp1SE_xjal;
districtHosp1SE_xjal = self._districtHosp1SE_DefaultValue_xjal();
if (callOnChangeActions) {
  self.set_districtHosp1SE( districtHosp1SE_xjal );
} else {
  self.districtHosp1SE = districtHosp1SE_xjal;
}
double bobs1SE_xjal;
bobs1SE_xjal = self._bobs1SE_DefaultValue_xjal();
if (callOnChangeActions) {
  self.set_bobs1SE( bobs1SE_xjal );
} else {
  self.bobs1SE = bobs1SE_xjal;
}
double fees1SE_xjal;
fees1SE_xjal = self._fees1SE_DefaultValue_xjal();
if (callOnChangeActions) {
  self.set_fees1SE( fees1SE_xjal );
} else {
  self.fees1SE = fees1SE_xjal;
```

```
}
double interceptSE_xjal;
interceptSE_xjal = self._interceptSE_DefaultValue_xjal();
if (callOnChangeActions) {
  self.set_interceptSE( interceptSE_xjal );
} else {
  self.interceptSE = interceptSE_xjal;
}
double centralHosp2SE_xjal;
centralHosp2SE_xjal = self._centralHosp2SE_DefaultValue_xjal();
if (callOnChangeActions) {
  self.set_centralHosp2SE( centralHosp2SE_xjal );
} else {
  self.centralHosp2SE = centralHosp2SE_xjal;
}
double distance2SE_xjal;
distance2SE_xjal = self._distance2SE_DefaultValue_xjal();
if (callOnChangeActions) {
  self.set_distance2SE( distance2SE_xjal );
} else {
  self.distance2SE = distance2SE_xjal;
}
double clinic2SE_xjal;
clinic2SE_xjal = self._clinic2SE_DefaultValue_xjal();
if (callOnChangeActions) {
  self.set_clinic2SE( clinic2SE_xjal );
} else {
  self.clinic2SE = clinic2SE_xjal;
}
double maternity2SE_xjal;
maternity2SE_xjal = self._maternity2SE_DefaultValue_xjal();
if (callOnChangeActions) {
  self.set_maternity2SE( maternity2SE_xjal );
} else {
  self.maternity2SE = maternity2SE_xjal;
}
double healthCenter2SE_xjal;
healthCenter2SE_xjal = self._healthCenter2SE_DefaultValue_xjal();
if (callOnChangeActions) {
  self.set_healthCenter2SE( healthCenter2SE_xjal );
} else {
  self.healthCenter2SE = healthCenter2SE_xjal;
}
double otherHosp2SE_xjal;
otherHosp2SE_xjal = self._otherHosp2SE_DefaultValue_xjal();
if (callOnChangeActions) {
  self.set_otherHosp2SE( otherHosp2SE_xjal );
} else {
  self.otherHosp2SE = otherHosp2SE_xjal;
}
double communityHosp2SE_xjal;
communityHosp2SE_xjal = self._communityHosp2SE_DefaultValue_xjal();
if (callOnChangeActions) {
  self.set_communityHosp2SE( communityHosp2SE_xjal );
} else {
  self.communityHosp2SE = communityHosp2SE_xjal;
}
double districtHosp2SE_xjal;
districtHosp2SE_xjal = self._districtHosp2SE_DefaultValue_xjal();
if (callOnChangeActions) {
  self.set_districtHosp2SE( districtHosp2SE_xjal );
} else {
  self.districtHosp2SE = districtHosp2SE_xjal;
```

```
}
double bobs2SE_xjal;
bobs2SE_xjal = self._bobs2SE_DefaultValue_xjal();
if (callOnChangeActions) {
  self.set_bobs2SE( bobs2SE_xjal );
} else {
  self.bobs2SE = bobs2SE_xjal;
}
double fees2SE_xjal;
fees2SE_xjal = self._fees2SE_DefaultValue_xjal();
if (callOnChangeActions) {
  self.set_fees2SE( fees2SE_xjal );
} else {
  self.fees2SE = fees2SE_xjal;
}
double centralCS_xjal;
centralCS_xjal = self._centralCS_DefaultValue_xjal();
if (callOnChangeActions) {
  self.set_centralCS( centralCS_xjal );
} else {
  self.centralCS = centralCS_xjal;
}
double clinicCS_xjal;
clinicCS_xjal = self._clinicCS_DefaultValue_xjal();
if (callOnChangeActions) {
  self.set_clinicCS( clinicCS_xjal );
} else {
  self.clinicCS = clinicCS_xjal;
}
double maternityCS_xjal;
maternityCS_xjal = self._maternityCS_DefaultValue_xjal();
if (callOnChangeActions) {
  self.set_maternityCS( maternityCS_xjal );
} else {
  self.maternityCS = maternityCS_xjal;
}
double healthCenterCS_xjal;
healthCenterCS_xjal = self._healthCenterCS_DefaultValue_xjal();
if (callOnChangeActions) {
  self.set_healthCenterCS( healthCenterCS_xjal );
} else {
  self.healthCenterCS = healthCenterCS_xjal;
}
double otherCS_xjal;
otherCS_xjal = self._otherCS_DefaultValue_xjal();
if (callOnChangeActions) {
  self.set_otherCS( otherCS_xjal );
} else {
  self.otherCS = otherCS_xjal;
}
double communityCS_xjal;
communityCS_xjal = self._communityCS_DefaultValue_xjal();
if (callOnChangeActions) {
  self.set_communityCS( communityCS_xjal );
} else {
  self.communityCS = communityCS_xjal;
}
double districtCS_xjal;
districtCS_xjal = self._districtCS_DefaultValue_xjal();
if (callOnChangeActions) {
  self.set_districtCS( districtCS_xjal );
} else {
  self.districtCS = districtCS_xjal;
```

```
}
double centralCSSE_xjal;
centralCSSE_xjal = self._centralCSSE_DefaultValue_xjal();
if (callOnChangeActions) {
  self.set_centralCSSE( centralCSSE_xjal );
} else {
  self.centralCSSE = centralCSSE_xjal;
}
double clinicCSSE_xjal;
clinicCSSE_xjal = self._clinicCSSE_DefaultValue_xjal();
if (callOnChangeActions) {
  self.set_clinicCSSE( clinicCSSE_xjal );
} else {
  self.clinicCSSE = clinicCSSE_xjal;
}
double maternityCSSE_xjal;
maternityCSSE_xjal = self._maternityCSSE_DefaultValue_xjal();
if (callOnChangeActions) {
  self.set_maternityCSSE( maternityCSSE_xjal );
} else {
  self.maternityCSSE = maternityCSSE_xjal;
}
double healthCenterCSSE_xjal;
healthCenterCSSE_xjal = self._healthCenterCSSE_DefaultValue_xjal();
if (callOnChangeActions) {
  self.set_healthCenterCSSE( healthCenterCSSE_xjal );
} else {
  self.healthCenterCSSE = healthCenterCSSE_xjal;
}
double otherCSSE_xjal;
otherCSSE_xjal = self._otherCSSE_DefaultValue_xjal();
if (callOnChangeActions) {
  self.set_otherCSSE( otherCSSE_xjal );
} else {
  self.otherCSSE = otherCSSE_xjal;
}
double communityCSSE_xjal;
communityCSSE_xjal = self._communityCSSE_DefaultValue_xjal();
if (callOnChangeActions) {
  self.set_communityCSSE( communityCSSE_xjal );
} else {
  self.communityCSSE = communityCSSE_xjal;
}
double districtCSSE_xjal;
districtCSSE_xjal = self._districtCSSE_DefaultValue_xjal();
if (callOnChangeActions) {
  self.set_districtCSSE( districtCSSE_xjal );
} else {
  self.districtCSSE = districtCSSE_xjal;
}
double centralNo_xjal;
centralNo_xjal = self._centralNo_DefaultValue_xjal();
if (callOnChangeActions) {
  self.set_centralNo( centralNo_xjal );
} else {
  self.centralNo = centralNo_xjal;
}
double clinicNo_xjal;
clinicNo_xjal = self._clinicNo_DefaultValue_xjal();
if (callOnChangeActions) {
  self.set_clinicNo( clinicNo_xjal );
} else {
  self.clinicNo = clinicNo_xjal;
```

```
}
double maternityNo_xjal;
maternityNo_xjal = self._maternityNo_DefaultValue_xjal();
if (callOnChangeActions) {
  self.set_maternityNo( maternityNo_xjal );
} else {
  self.maternityNo = maternityNo_xjal;
}
double healthCenterNo_xjal;
healthCenterNo_xjal = self._healthCenterNo_DefaultValue_xjal();
if (callOnChangeActions) {
  self.set_healthCenterNo( healthCenterNo_xjal );
} else {
  self.healthCenterNo = healthCenterNo_xjal;
}
double otherNo_xjal;
otherNo_xjal = self._otherNo_DefaultValue_xjal();
if (callOnChangeActions) {
  self.set_otherNo( otherNo_xjal );
} else {
  self.otherNo = otherNo_xjal;
}
double communityNo_xjal;
communityNo_xjal = self._communityNo_DefaultValue_xjal();
if (callOnChangeActions) {
  self.set_communityNo( communityNo_xjal );
} else {
  self.communityNo = communityNo_xjal;
}
double districtNo_xjal;
districtNo_xjal = self._districtNo_DefaultValue_xjal();
if (callOnChangeActions) {
  self.set_districtNo( districtNo_xjal );
} else {
  self.districtNo = districtNo_xjal;
}
double centralNoSE_xjal;
centralNoSE_xjal = self._centralNoSE_DefaultValue_xjal();
if (callOnChangeActions) {
  self.set_centralNoSE( centralNoSE_xjal );
} else {
  self.centralNoSE = centralNoSE_xjal;
}
double clinicNoSE_xjal;
clinicNoSE_xjal = self._clinicNoSE_DefaultValue_xjal();
if (callOnChangeActions) {
  self.set_clinicNoSE( clinicNoSE_xjal );
} else {
  self.clinicNoSE = clinicNoSE_xjal;
}
double maternityNoSE_xjal;
maternityNoSE_xjal = self._maternityNoSE_DefaultValue_xjal();
if (callOnChangeActions) {
  self.set_maternityNoSE( maternityNoSE_xjal );
} else {
  self.maternityNoSE = maternityNoSE_xjal;
}
double healthCenterNoSE_xjal;
healthCenterNoSE_xjal = self._healthCenterNoSE_DefaultValue_xjal();
if (callOnChangeActions) {
  self.set_healthCenterNoSE( healthCenterNoSE_xjal );
} else {
  self.healthCenterNoSE = healthCenterNoSE_xjal;
```

```
}
double otherNoSE_xjal;
otherNoSE_xjal = self._otherNoSE_DefaultValue_xjal();
if (callOnChangeActions) {
  self.set_otherNoSE( otherNoSE_xjal );
} else {
  self.otherNoSE = otherNoSE_xjal;
}
double communityNoSE_xjal;
communityNoSE_xjal = self._communityNoSE_DefaultValue_xjal();
if (callOnChangeActions) {
  self.set_communityNoSE( communityNoSE_xjal );
} else {
  self.communityNoSE = communityNoSE_xjal;
}
double districtNoSE_xjal;
districtNoSE_xjal = self._districtNoSE_DefaultValue_xjal();
if (callOnChangeActions) {
  self.set_districtNoSE( districtNoSE_xjal );
} else {
  self.districtNoSE = districtNoSE_xjal;
}
double central5_xjal;
central5_xjal = self._central5_DefaultValue_xjal();
if (callOnChangeActions) {
  self.set_central5( central5_xjal );
} else {
  self.central5 = central5_xjal;
}
double clinic5_xjal;
clinic5_xjal = self._clinic5_DefaultValue_xjal();
if (callOnChangeActions) {
  self.set_clinic5( clinic5_xjal );
} else {
  self.clinic5 = clinic5_xjal;
}
double maternity5_xjal;
maternity5_xjal = self._maternity5_DefaultValue_xjal();
if (callOnChangeActions) {
  self.set_maternity5( maternity5_xjal );
} else {
  self.maternity5 = maternity5_xjal;
}
double healthCenter5_xjal;
healthCenter5_xjal = self._healthCenter5_DefaultValue_xjal();
if (callOnChangeActions) {
  self.set_healthCenter5( healthCenter5_xjal );
} else {
  self.healthCenter5 = healthCenter5_xjal;
}
double other5_xjal;
other5_xjal = self._other5_DefaultValue_xjal();
if (callOnChangeActions) {
  self.set_other5( other5_xjal );
} else {
  self.other5 = other5_xjal;
}
double community5_xjal;
community5_xjal = self._community5_DefaultValue_xjal();
if (callOnChangeActions) {
  self.set_community5( community5_xjal );
} else {
  self.community5 = community5_xjal;
```

```
}
double district5_xjal;
district5_xjal = self._district5_DefaultValue_xjal();
if (callOnChangeActions) {
  self.set_district5( district5_xjal );
} else {
  self.district5 = district5_xjal;
}
double central5SE_xjal;
central5SE_xjal = self._central5SE_DefaultValue_xjal();
if (callOnChangeActions) {
  self.set_central5SE( central5SE_xjal );
} else {
  self.central5SE = central5SE_xjal;
}
double clinic5SE_xjal;
clinic5SE_xjal = self._clinic5SE_DefaultValue_xjal();
if (callOnChangeActions) {
  self.set_clinic5SE( clinic5SE_xjal );
} else {
  self.clinic5SE = clinic5SE_xjal;
}
double maternity5SE_xjal;
maternity5SE_xjal = self._maternity5SE_DefaultValue_xjal();
if (callOnChangeActions) {
  self.set_maternity5SE( maternity5SE_xjal );
} else {
  self.maternity5SE = maternity5SE_xjal;
}
double healthCenter5SE_xjal;
healthCenter5SE_xjal = self._healthCenter5SE_DefaultValue_xjal();
if (callOnChangeActions) {
  self.set_healthCenter5SE( healthCenter5SE_xjal );
} else {
  self.healthCenter5SE = healthCenter5SE_xjal;
}
double other5SE_xjal;
other5SE_xjal = self._other5SE_DefaultValue_xjal();
if (callOnChangeActions) {
  self.set_other5SE( other5SE_xjal );
} else {
  self.other5SE = other5SE_xjal;
}
double community5SE_xjal;
community5SE_xjal = self._community5SE_DefaultValue_xjal();
if (callOnChangeActions) {
  self.set_community5SE( community5SE_xjal );
} else {
  self.community5SE = community5SE_xjal;
}
double district5SE_xjal;
district5SE_xjal = self._district5SE_DefaultValue_xjal();
if (callOnChangeActions) {
  self.set_district5SE( district5SE_xjal );
} else {
  self.district5SE = district5SE_xjal;
}
double centralNo5_xjal;
centralNo5_xjal = self._centralNo5_DefaultValue_xjal();
if (callOnChangeActions) {
  self.set_centralNo5( centralNo5_xjal );
} else {
  self.centralNo5 = centralNo5_xjal;
```

```
}
double clinicNo5_xjal;
clinicNo5_xjal = self._clinicNo5_DefaultValue_xjal();
if (callOnChangeActions) {
  self.set_clinicNo5( clinicNo5_xjal );
} else {
  self.clinicNo5 = clinicNo5_xjal;
}
double maternityNo5_xjal;
maternityNo5_xjal = self._maternityNo5_DefaultValue_xjal();
if (callOnChangeActions) {
  self.set_maternityNo5( maternityNo5_xjal );
} else {
  self.maternityNo5 = maternityNo5_xjal;
}
double healthCenterNo5_xjal;
healthCenterNo5_xjal = self._healthCenterNo5_DefaultValue_xjal();
if (callOnChangeActions) {
  self.set_healthCenterNo5( healthCenterNo5_xjal );
} else {
  self.healthCenterNo5 = healthCenterNo5_xjal;
}
double otherNo5_xjal;
otherNo5_xjal = self._otherNo5_DefaultValue_xjal();
if (callOnChangeActions) {
  self.set_otherNo5( otherNo5_xjal );
} else {
  self.otherNo5 = otherNo5_xjal;
}
double communityNo5_xjal;
communityNo5_xjal = self._communityNo5_DefaultValue_xjal();
if (callOnChangeActions) {
  self.set_communityNo5( communityNo5_xjal );
} else {
  self.communityNo5 = communityNo5_xjal;
}
double districtNo5_xjal;
districtNo5_xjal = self._districtNo5_DefaultValue_xjal();
if (callOnChangeActions) {
  self.set_districtNo5( districtNo5_xjal );
} else {
  self.districtNo5 = districtNo5_xjal;
}
double centralNo5SE_xjal;
centralNo5SE_xjal = self._centralNo5SE_DefaultValue_xjal();
if (callOnChangeActions) {
  self.set_centralNo5SE( centralNo5SE_xjal );
} else {
  self.centralNo5SE = centralNo5SE_xjal;
}
double clinicNo5SE_xjal;
clinicNo5SE_xjal = self._clinicNo5SE_DefaultValue_xjal();
if (callOnChangeActions) {
  self.set_clinicNo5SE( clinicNo5SE_xjal );
} else {
  self.clinicNo5SE = clinicNo5SE_xjal;
}
double maternityNo5SE_xjal;
maternityNo5SE_xjal = self._maternityNo5SE_DefaultValue_xjal();
if (callOnChangeActions) {
  self.set_maternityNo5SE( maternityNo5SE_xjal );
} else {
  self.maternityNo5SE = maternityNo5SE_xjal;
```

```
}
double healthCenterNo5SE_xjal;
healthCenterNo5SE_xjal = self._healthCenterNo5SE_DefaultValue_xjal();
if (callOnChangeActions) {
  self.set_healthCenterNo5SE( healthCenterNo5SE_xjal );
} else {
  self.healthCenterNo5SE = healthCenterNo5SE_xjal;
}
double otherNo5SE_xjal;
otherNo5SE_xjal = self._otherNo5SE_DefaultValue_xjal();
if (callOnChangeActions) {
  self.set_otherNo5SE( otherNo5SE_xjal );
} else {
  self.otherNo5SE = otherNo5SE_xjal;
}
double communityNo5SE_xjal;
communityNo5SE_xjal = self._communityNo5SE_DefaultValue_xjal();
if (callOnChangeActions) {
  self.set_communityNo5SE( communityNo5SE_xjal );
} else {
  self.communityNo5SE = communityNo5SE_xjal;
}
double districtNo5SE_xjal;
districtNo5SE_xjal = self._districtNo5SE_DefaultValue_xjal();
if (callOnChangeActions) {
  self.set_districtNo5SE( districtNo5SE_xjal );
} else {
  self.districtNo5SE = districtNo5SE_xjal;
}
double anc4_xjal;
anc4_xjal = self._anc4_DefaultValue_xjal();
if (callOnChangeActions) {
  self.set_anc4( anc4_xjal );
} else {
  self.anc4 = anc4_xjal;
}
double unwanted_xjal;
unwanted_xjal = self._unwanted_DefaultValue_xjal();
if (callOnChangeActions) {
  self.set_unwanted( unwanted_xjal );
} else {
  self.unwanted = unwanted_xjal;
}
double risk_xjal;
risk_xjal = self._risk_DefaultValue_xjal();
if (callOnChangeActions) {
  self.set_risk( risk_xjal );
} else {
  self.risk = risk_xjal;
}
double urban_xjal;
urban_xjal = self._urban_DefaultValue_xjal();
if (callOnChangeActions) {
  self.set_urban( urban_xjal );
} else {
  self.urban = urban_xjal;
}
double twins_xjal;
twins_xjal = self._twins_DefaultValue_xjal();
if (callOnChangeActions) {
  self.set_twins( twins_xjal );
} else {
  self.twins = twins_xjal;
```

```
}
double educSec_xjal;
educSec_xjal = self._educSec_DefaultValue_xjal();
if (callOnChangeActions) {
  self.set_educSec( educSec_xjal );
} else {
  self.educSec = educSec_xjal;
}
double spouseAge_xjal;
spouseAge_xjal = self._spouseAge_DefaultValue_xjal();
if (callOnChangeActions) {
  self.set_spouseAge( spouseAge_xjal );
} else {
  self.spouseAge = spouseAge_xjal;
}
double age_xjal;
age_xjal = self._age_DefaultValue_xjal();
if (callOnChangeActions) {
  self.set_age( age_xjal );
} else {
  self.age = age_xjal;
}
double csPlanned_xjal;
csPlanned_xjal = self._csPlanned_DefaultValue_xjal();
if (callOnChangeActions) {
  self.set_csPlanned( csPlanned_xjal );
} else {
  self.csPlanned = csPlanned_xjal;
}
double anc4SE_xjal;
anc4SE_xjal = self._anc4SE_DefaultValue_xjal();
if (callOnChangeActions) {
  self.set_anc4SE( anc4SE_xjal );
} else {
  self.anc4SE = anc4SE_xjal;
}
double unwantedSE_xjal;
unwantedSE_xjal = self._unwantedSE_DefaultValue_xjal();
if (callOnChangeActions) {
  self.set_unwantedSE( unwantedSE_xjal );
} else {
  self.unwantedSE = unwantedSE_xjal;
}
double riskSE_xjal;
riskSE_xjal = self._riskSE_DefaultValue_xjal();
if (callOnChangeActions) {
  self.set_riskSE( riskSE_xjal );
} else {
  self.riskSE = riskSE_xjal;
}
double urbanSE_xjal;
urbanSE_xjal = self._urbanSE_DefaultValue_xjal();
if (callOnChangeActions) {
  self.set_urbanSE( urbanSE_xjal );
} else {
  self.urbanSE = urbanSE_xjal;
}
double twinsSE_xjal;
twinsSE_xjal = self._twinsSE_DefaultValue_xjal();
if (callOnChangeActions) {
  self.set_twinsSE( twinsSE_xjal );
} else {
  self.twinsSE = twinsSE_xjal;
```

```
    }
    double educSecSE_xjal;
    educSecSE_xjal = self._educSecSE_DefaultValue_xjal();
    if (callOnChangeActions) {
      self.set_educSecSE( educSecSE_xjal );
    } else {
      self.educSecSE = educSecSE_xjal;
    }
    double spouseAgeSE_xjal;
    spouseAgeSE_xjal = self._spouseAgeSE_DefaultValue_xjal();
    if (callOnChangeActions) {
      self.set_spouseAgeSE( spouseAgeSE_xjal );
    } else {
      self.spouseAgeSE = spouseAgeSE_xjal;
    }
    double ageSE_xjal;
    ageSE_xjal = self._ageSE_DefaultValue_xjal();
    if (callOnChangeActions) {
      self.set_ageSE( ageSE_xjal );
    } else {
      self.ageSE = ageSE_xjal;
    }
    double csPlannedSE_xjal;
    csPlannedSE_xjal = self._csPlannedSE_DefaultValue_xjal();
    if (callOnChangeActions) {
      self.set_csPlannedSE( csPlannedSE_xjal );
    } else {
      self.csPlannedSE = csPlannedSE_xjal;
    }
    double QIParam_xjal;
    QIParam_xjal = self._QIParam_DefaultValue_xjal();
    if (callOnChangeActions) {
      self.set_QIParam( QIParam_xjal );
    } else {
      self.QIParam = QIParam_xjal;
    }
    int strategy_xjal;
    strategy_xjal = self._strategy_DefaultValue_xjal();
    if (callOnChangeActions) {
      self.set_strategy( strategy_xjal );
    } else {
      self.strategy = strategy_xjal;
    }
  }

}
```

References

1. Gage AD, Carnes F, Blossom J, Aluvaala J, Amatya A, Mahat K, et al. Improving delivery care quality at scale: Modelling service redesign in six countries. 2018-forthcoming.
2. Tatem AJ. WorldPop, open data for spatial demography. Scientific data. 2017;4:170004–.
3. Tatem AJ, Campbell J, Guerra-Arias M, De Bernis L, Moran A, Matthews Z. Mapping for maternal and newborn health: the distributions of women of childbearing age, pregnancies and births. International journal of health geographics. 2014;13(1):2.
4. Leslie HH, Fink G, Nsona H, Kruk ME. Obstetric Facility Quality and Newborn Mortality in Malawi: A Cross-Sectional Study. PLoS Med. 2016;13(10):e1002151.
